# Different strategies of metabolic regulation in cyanobacteria: from transcriptional to biochemical control

**DOI:** 10.1038/srep33024

**Published:** 2016-09-09

**Authors:** Jiri Jablonsky, Stepan Papacek, Martin Hagemann

**Affiliations:** 1 Institute of Complex Systems, FFPW, University of South Bohemia, Cenakva, Czech Republic; 2 Department of Plant Physiology, University of Rostock, Einsteinstr. 3, D-18059 Rostock, Germany

## Abstract

Cyanobacteria *Synechococcus* sp. PCC 7942 and *Synechocystis* sp. PCC 6803 show similar changes in the metabolic response to changed CO_2_ conditions but exhibit significant differences at the transcriptomic level. This study employs a systems biology approach to investigate the difference in metabolic regulation of *Synechococcus* sp. PCC 7942 and *Synechocystis* sp. PCC 6803. Presented multi-level kinetic model for *Synechocystis* sp. PCC 6803 is a new approach integrating and analysing metabolomic, transcriptomic and fluxomics data obtained under high and ambient CO_2_ levels. Modelling analysis revealed that higher number of different isozymes in *Synechocystis* 6803 improves homeostatic stability of several metabolites, especially 3PGA by 275%, against changes in gene expression, compared to *Synechococcus* sp. PCC 7942. Furthermore, both cyanobacteria have the same amount of phosphoglycerate mutases but *Synechocystis* 6803 exhibits only ~20% differences in their mRNA levels after shifts from high to ambient CO_2_ level, in comparison to ~500% differences in the case of *Synechococcus* sp. PCC 7942. These and other data imply that the biochemical control dominates over transcriptional regulation in *Synechocystis* 6803 to acclimate central carbon metabolism in the environment of variable inorganic carbon availability without extra cost carried by large changes in the proteome.

Cyanobacteria are the only prokaryotes able to perform oxygenic photosynthesis. This process converts inorganic carbon (Ci) into sugars at the expense of light energy. The ability of autotrophic carbon assimilation makes cyanobacteria attractive for biotechnological application to produce bioenergy or chemical feedstock^1^,^2^,^3^. Most of these products branch off central carbon metabolism. CO_2_ fixation via the Calvin-Benson cycle is closely associated with photorespiratory 2-phosphoglycolate (2PG) metabolism^4^, glycolysis, the oxidative pentose phosphate (OPP) cycle, and the tricarboxylic acid (TCA) cycle, which mainly serves to produce 2-oxoglutarate (2OG) as a precursor for ammonia assimilation. Excess organic carbon that is not immediately used for biosynthesis or growth can be stored as glycogen as the main carbon storage, which is consumed during the night^5^,^6^.

Whereas principal carbon assimilation was thoroughly studied in recent decades, we lack knowledge regarding how cellular functions are connected and regulated in photoautotrophic cells. For example, metabolomics was applied to analyse global changes in the central carbon and nitrogen assimilation in model cyanobacteria such as *Synechocystis* sp. strain PCC 6803 (hereafter referred to as *Synechocystis* 6803)^7^,^8^ and *Synechococcus elongatus* strain PCC 7942 (hereafter referred to as *Synechococcus* 7942)^9^ in cells shifted from high CO_2_ atmosphere (supplemented with 5% CO_2_, HC) to the ambient CO_2_ level of ambient air (0.04% CO_2_, AC). These studies demonstrated that a high proportion of carbon is incorporated into glycogen under HC, whereas AC conditions induce a carbon drain from the Calvin-Benson cycle via glycolysis and the TCA cycle to sustain amino acids synthesis and activate the photorespiratory 2PG metabolism. These changes are accompanied by a transient accumulation of photorespiratory intermediates, the so-called photorespiratory burst^10^, as well as stable accumulation of 2-phosphoglycerate (2PGA) and phosphoenolpyruvate (PEP). Recent labelling experiments supported the conclusions regarding altered carbon allocation in cells exposed to different carbon levels^11^,^12^.

However, the handling of large data sets from “omics” experiments, such as transcriptomics, remains challenging. In the frame of systems biology, attempts were initiated to integrate those data into mathematical models^13^,^14^,^15^. These approaches not only support the handling of complex data sets but also allow analysis of those data sets to reveal metabolic and regulatory networks.

The current standard method for mathematical analysis of metabolism, functions of particular pathways or the effects of specific gene knock outs is stoichiometric modelling^16^. This method is suitable for large metabolic networks and is based on enforcing constraints on the metabolic fluxes, such as CO_2_ fixation and glycogen synthesis, allowing to predict the flux distribution. Common method employed within stoichiometric modelling is flux balance analysis (FBA)^17^, proved to be valuable for the evaluation of the metabolism of *Synechocystis* 6803^5^,^18^,^19^,^20^,^21^. However, FBA is a static approach and its advanced derivatives, e.g., regulatory FBA (rFBA)^22^, provide only limited information regarding dynamics and system regulation. Alternatively, kinetic models^23^,^24^,^25^ can be used for the description of cellular functions in photosynthetic organisms. These models require more knowledge and data input than the stoichiometric models, but they allow the description and analysis of different cellular states.

We have extended the traditional kinetic model, based on metabolic data from one steady state, to multi-level kinetic model (various omics data and several steady states) and applied this approach to analyse the metabolic regulation of cyanobacterium *Synechococcus* 7942^14^,^26^. This multi-level kinetic modelling approach combines enzymatic data from the literature and experimentally obtained metabolic, fluxomic and transcriptomics data from several steady states to describe central carbon metabolism in this organism. Gene expression data served as a proxy to estimate changes in the amount of specific enzyme in cells grown under different CO_2_ conditions^14^. The gene expression analysis revealed that the cyanobacterium *Synechococcus* 7942 exhibits major changes in expression of genes for many enzymes involved in central carbon metabolism after a shift from HC to AC^9^.

Here, we report on a multi-level kinetic model for the cyanobacterium *Synechocystis* 6803 and its application to analyse metabolic alterations in cells shifted from HC (high CO_2_) to AC (ambient CO_2_) conditions. In contrast to *Synechococcus* 7942, *Synechocystis* 6803 cells did not show significant changes in the expression of enzymes involved in central carbon metabolism^27^,^28^, whereas the principal changes in the metabolome were similar in both cyanobacteria during the AC shift response^29^. These findings may indicate that in *Synechocystis* 6803 carbon metabolism is rather regulated at the posttranslational, i.e. biochemical, level during the acclimation to different CO_2_ levels. In this study, we focus on three major topics: 1) differences in the metabolic regulation between *Synechococcus* 7942 and *Synechocystis* 6803, 2) the role of additional isozymes in *Synechocystis* 6803, and 3) the influence of gene expression on metabolic fluxes.

## Results and Discussion

### Comparison of two cyanobacteria revealed the dominant role of isozymes in metabolic regulation in *Synechocystis* 6803

**Figure 1:**
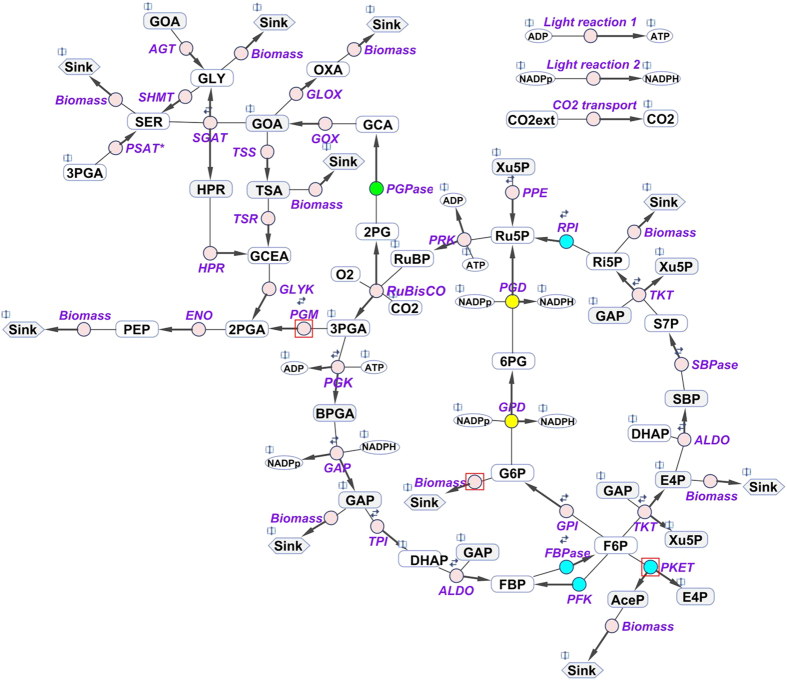
Schematic representation of the central carbon metabolism network, which was implemented in the multi-level kinetic model of *Synechocystis* 6803. Differences in enzymes involved in central carbon metabolism between *Synechococcus* 7942 and *Synechocystis* 6803 were included. **Blue** indicates isozymes in *Synechocystis* 6803 that are missing in *Synechococcus* 7942. **Green** indicates isozyme in *Synechocystis* 6803 with higher amount of isozymes in comparison to *Synechococcus* 7942. **Yellow** indicates the newly added oxidative phase of the oxidative pentose phosphate pathway, which is active also during the day (HC condition) in *Synechocystis* 6803, in contrast to *Synechococcus* 7942. Transaldolase was omitted due to low flux^11^, and phosphogluconolactonase was neglected for simplicity. **White** indicates the metabolites whose concentrations are available in the metabolome data set. The model includes the Calvin-Benson cycle, glycogen synthesis (sink from glucose-6-phosphate), photorespiratory pathways, glycolysis, the oxidative pentose pathway and sink reactions (representing the adjacent pathway and the calculation of biomass production). The reversibility of a particular reaction is indicated by two small arrows. **Red squares** indicate the major carbon reallocation nodes for changing CO_2_ levels. **Purple** indicates the involved enzymes: RuBisCO–ribulose-1,5-bisphosphate carboxylase oxygenase, PGK–phosphoglycerate kinase, GAP–glyceraldehyde-3-phosphate dehydrogenase, TPI–triose-phosphate isomerase, ALDO–aldolase, FBPase–fructose-1,6 bisphosphatase, PFK–phosphofructokinase, TKT–transketolase, SBPase–sedoheptulose-1,7 bisphosphatase, RPI –phosphopentose isomerase, PPE–phosphopentose epimerase, PRK –phosphoribulokinase, GPI–glucose-6-phosphate isomerase, G6PD–glucose-6-phosphate dehydrogenase, PGD–phosphogluconate dehydrogenase, PGPase–phosphoglycolate phosphatase, PKET–phosphoketolase, GOX –glycolate oxidase, SGAT–serineglyoxylate transaminase, HPR –hydroxypyruvate reductase, GLYK –glycerate kinase, AGT–alanine-glyoxylate transaminase, TSS –tartronatesemialdehyde synthase, TSR–tartronatesemialdehyde reductase, SHMT–serine hydroxymethyltransferase, GLOX –glyoxylate oxidase, PSAT*–phosphoserine transaminase (3-phosphoglycerate dehydrogenase is, for simplicity, not implemented), PGM–phosphoglycerate mutase, ENO–enolase. **Open book symbol** indicates an involvement of metabolite in other reaction(s).

The model of *Synechocystis* 6803 was used to compare central carbon metabolism and its acclimation to varying Ci conditions with that of *Synechococcus* 7942. These two unicellular cyanobacterial strains are often used as models to investigate molecular aspects of carbon acclimation^30^,^31^. Compared with *Synechocystis* 6803, *Synechococcus* 7942 has an approximately 40% smaller genome (http://genome.microbedb.jp/cyanobase) and is strictly photoautotrophic. Hence, the metabolism of *Synechocystis* 6803 is more complex and flexible, thus making it more difficult to elucidate. The different genomic capacity also resulted in significant differences within the metabolic network between these cyanobacteria. In particular, *Synechocystis* 6803 runs an active OPP cycle under phototrophic conditions^11^ and harbours more isozymes of fructose-1,6-bisphosphatase, phosphofructokinase, ribose-5-phosphate isomerase, phosphoglycolate phosphatase and phosphoketolase^32^. With the exception of ribose-5-phosphate isomerase and phosphoglycolate phosphatase, the position of these additional isozymes in the metabolic network potentially allows them to regulate the flux of fructose-6-phosphate among the Calvin-Benson cycle, glycolysis, OPP and/or glycolytic bypass, see Fig. 1. However, *Synechocystis* 6803 also lacks some enzymes compared with *Synechococcus* 7942, such as the glycerate 3-phosphate kinase in the photorespiratory pathway, which is replaced by a glycerate 2-phosphate kinase^33^, and one of the glycolytic glyceraldehyde-3-phosphate dehydrogenases.

As previously mentioned, *Synechocystis* 6803 cells did not show significant changes in the expression of genes for enzymes of central carbon metabolism triggered by changing CO_2_ conditions^27^,^28^, whereas in *Synechococcus* 7942 those changes mainly explained the observed changes in the carbon metabolism in HC compared to AC cells^9^,^26^. It is notable that the mean value of transcriptional changes of the enzymes implemented into the model is 1.01 between HC and AC cells, whereas it was 1.36 in *Synechococcus* 7942. Despite the unchanged expression, *Synechocystis* 6803 exhibited marked differences in metabolites under different Ci conditions (see Table 1), which are comparable to those reported from *Synechococcus* 7942^9^,^26^. Hence, the major difference between *Synechocystis* 6803 and *Synechococcus* 7942 seems to be based on different ranges of transcriptional regulation and the amount of isozymes for crucial metabolic nodes. This raises the question if isozymes play a key role in the metabolic regulation of *Synechocystis* 6803.

To test this hypothesis, we assessed the role of transcriptional control over metabolism. To do that, measured changes in the transcriptome of *Synechocystis* 6803 cells shifted from HC to AC were obtained from the literature^29^ and then shuffled and randomly redistributed as weight factors of enzymatic activities. Randomized distribution of transcriptome data was employed in other studies for different reasons, for instance, to elucidate the role of particular metabolic pathway in a genomic model^34^. In our case, the varying transcriptional changes were applied to the complete model with all isozymes occurring in *Synechocystis* 6803 and to a model, in which all the isozymes that are missing in *Synechococcus* 7942 were not considered (see Fig. 1, blue colour labels these enzymes). We note that the deletion of isozymes, i.e., keeping only one isozyme per reaction in the model, would dramatically affect the results of the simulations obtained with and without isozymes, because the kinetic parameters of all isozymes, including the remaining isozyme, were initially estimated for two or more isozymes. Therefore, the kinetic parameters of the remaining single isozymes (Fig. 1, blue color) were rebalanced to obtain a good match with experimental data for cells grown in HC and AC. The manual rebalancing of kinetic parameters of the each remaining single isozyme started with shifting the k_M_single_ value to the mean of k_M_ values or original isozymes followed by adjusting the V_max_single_ accordingly to match the measured metabolic levels. More information about the parameter space for k_M_ of isozymes can be found in our previous study^14^. After the rebalancing procedure, we repeated the simulation, based on shuffled and each time randomly redistributed weight factors, of metabolic changes in Ci-shifted cells 30-times for both scenarios.

**Figure 2:**
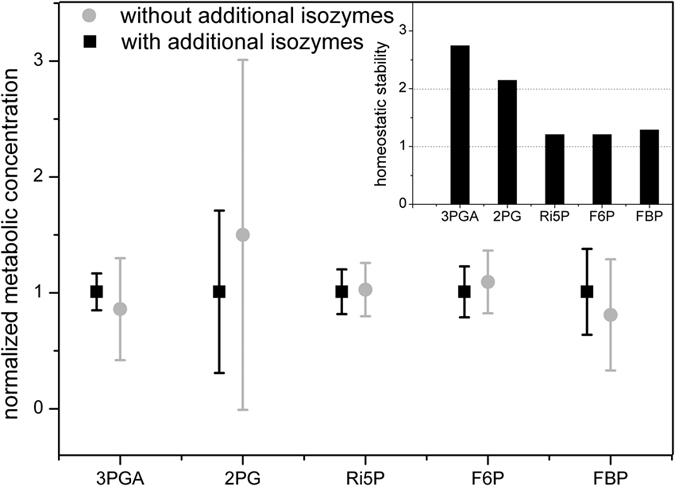
Impact of the random redistribution of transcriptional changes on the metabolic level of *Synechocystis* 6803 at ambient CO_2_ using in multi-level kinetic models with or without including isozymes missing in *Synechococcus* 7942 but present in *Synechocystis* 6803. **Black squares** indicate the mean value of 30 simulations with randomly redistribution of transcriptional changes on the metabolite levels in the model with all isozymes. **Grey circles** indicate the mean value of 30 simulations with random redistribution of transcriptional changes on the metabolite levels in the model without the aforementioned isozymes. **Black columns** indicate the homeostatic stability for particular metabolite defined as a ratio of standard deviations from simulated concentrations between results without and with additional isozymes. The error bars represent the standard deviation of the simulated data.

This analysis of the metabolic levels in AC-grown cells revealed that the higher number of isozymes present in *Synechocystis* 6803 (but absent in *Synechococcus* 7942) affect the behaviour of many metabolites. This effect is particularly visible in the homeostatic stability of 3PGA and 2PG, which increased by 275% and 215%, respectively (Fig. 2). Similar positive impact on homeostatic stability of F6P, FBP and Ri5P (substrates of additional isozymes) is approximately 20% (Fig. 2), but was lower in the case of other metabolites, e.g., +2.1% for DHAP or +2.5% for E4P. The homeostatic stability for these metabolic concentrations is here defined as a ratio of standard deviations from series of simulations with randomly redistributed measured transcriptomic changes (see above) between results without and with additional isozymes. In other words, homeostatic stability for particular metabolite describes the precision of maintaining aimed steady state concentrations in changing environments such as different CO_2_ levels. The high sensitivity of 2PG towards changes in the expression of corresponding genes (Fig. 2) correlates with the existence of additional 2PG phosphatase in *Synechocystis* to maintain the concentration of this toxic intermediate in changing environment, in contrast to the situation in *Synechococcus* 7942 with only two genes encoding for 2PG phosphatases^32^. In addition to the lowered homeostatic stability of 2PG in the case without additional isozymes, we observed also 50% higher mean concentration of 2PG which may inhibits certain enzymes of the Calvin-Benson cycle^12^; thus further explaining the existence of third phosphoglycolate phosphatase in *Synechocystis* 6803.

Another interesting finding of the simulations was an increased homeostatic stability of 3PGA by 275% due to more isozymes in *Synechocystis* (Fig. 2). 3PGA is neither substrate nor product of these isozymes and phosphoglycerate mutase isozymes (PGMs) are present in the same number in *Synechococcus* 7942 and *Synechocystis* 6803^32^. 3PGA acts on the crucial metabolic crossroad between the Calvin-Benson cycle and glycolysis, hence its concentration seems to have a significant impact on growth^26^. We recently showed that primary regulatory role of isozymes not necessarily aims at the catalysed reaction^26^, which seems be also the case of fructose-1,6-bisphosphatases, phosphofructokinases, ribose-5-phosphate isomerases and phosphoketolases. Finally, it is evident that comparable metabolic regulation of 3PGA levels can be achieved either with additional isozymes and almost unchanged expression of 3 PGMs (approximately 20% differences in mRNA levels) in *Synechocystis* after shifts from HC to AC^29^ or lower number of isozymes and high expression changes of PGMs (approximately 500% differences in mRNA levels) in *Synechococcus* after HC to AC shifts^9^. Finally, we have shown before that an extra PGM does not improve the homeostatic stability of 3PGA^14^; incorrect annotation of fourth PGM in *Synechococcus* 7942 and *Synechocystis* 6803 was identified by our multi-level kinetic modelling approach^14^ and recently experimentally confirmed as phosphoserine phosphatase in *Synechocystis* 6803^35^.

These results support the notion that the biochemical control, i.e., control provided by posttranslational level and isozymes, dominates over transcriptional regulation to acclimate central carbon metabolism from HC toward AC conditions in *Synechocystis* 6803 compared to *Synechococcus* 7942. Furthermore, the simulations showed that it is possible to describe the metabolic levels under different CO_2_ conditions using only one set of kinetic parameters, implying a minor role of posttranslational modifications. Finally, this finding may also explain why *Synechocystis* 6803 with its higher enzyme coding capacity does not strongly depend on the regulation of gene expression. Regulation via increased number of specific isozymes is sufficient to maintain the robust homeostasis at metabolic level without extra cost carried by large changes in the transcriptome and thus in the proteome, even in the environment of variable inorganic carbon availability.

Is carbon metabolism scalable in response to a changing environment? At this point, we have analysed two major inputs to our model: metabolic and transcriptomic data. However, estimations of the steady state levels of metabolites cannot be directly translated into carbon flux. This gap can be filled when data from ^13^C-labelling experiments, which directly estimate the metabolic fluxes, are considered as alternative input data^11^,^12^. Fluxes in cyanobacteria are commonly analysed by stoichiometric modelling, which is based on FBA (flux balance analysis) via genome-scale reconstruction of metabolic networks^5^,^18^,^19^,^20^,^21^. However, fluxes can also be calculated within kinetic models, e.g., the rates of reactions based on Michaelis-Menten kinetics are equal to fluxes in units of mmol s^−1 ^36. Such data can then be normalized to the measured flux, typically provided in units of mmol h^−1^g DW^−1 ^11. Moreover, the use of fluxes as input data provides additional constraints for parameter fitting in kinetic modelling.

Accordingly, we compared the calculated fluxes from our multi-level kinetic model of *Synechocystis* 6803 with the normalized estimated fluxes from a labelling experiment performed at HC conditions^11^ and those obtained from published calculations based on FBA^20^. This analysis reveals a good match between the fluxes calculated via our model and those measured in the labelling experiment (Table 2). This match was better than that between the fluxes calculated via our multi-level kinetic model here and stoichiometric FBA model^20^. In the latter case, we observed two major discrepancies, namely a 2.9-fold difference in glycogen synthesis (considered as G6P sink, see Fig. 1) and an 8.3-fold difference in phosphoketolase flux in cells grown in HC (Table 2). Comparing the HC fluxes between phosphoketolase and G6PD (Table 2) indicates that stoichiometric model opted for more beneficial flux via phosphoketolase that preserves carbon molecules in comparison to the route via glycolysis, which could explain the 8.3-fold difference. Although we do not have data from ^13^C labeling for the flux through phosphoketolase (PKET), we could calculate an estimate from other fluxes involving F6P. The maximal flux via PKET could not be higher than 5 10^−2^ mmol h^−1^ g DW^−1^. This estimation is based on the mass conversation law of reactions included within the model. In reality the flux could be lower, since the model is not considering reactions such as mannose-6-phosphate or fructose synthesis. To clarify the exact flux via PKET, new labelling experiment would be required.

Hence, these comparisons indicated that our model can also be used for accurate calculation of fluxes from metabolic steady state data in *Synechocystis* 6803 cells. To test whether the model provides conclusive flux calculation under different environmental conditions, we included flux values for cells cultivated in AC. Because there are no other resources to compare with, we rescaled the original values from Knoop stoichiometric model^20^. For this purpose, we considered a ratio of 3.6 based on difference in growth rates and corresponding ribulose synthesis fluxes between HC and AC resulting from our model. Using this rescaling factor, we divided all fluxes from the original Knoop stoichiometric model^20^ by a constant 3.6 to translate the HC values into normalized AC fluxes.

This attempt resulted in a good match for glycogen synthesis and a 4-fold improvement for phosphoketolase-driven flux under AC conditions (Table 2). Furthermore, the comparison of the fluxes based on our multi-level kinetic model and the rescaled values of Knoop model^20^ in AC revealed a better match than the same comparison for HC, see Table 2. These findings imply that the stoichiometric model^20^ describes rather the situation of *Synechocystis* 6803 under AC conditions. On this basis, the majority of fluxes are scalable for cells in changing CO_2_ conditions. We note that the stoichiometric model^20^ was designed for HC allowing to adjust HCO_3_^**−**^ uptake as necessary for optimal growth yield.

However, there are three exceptions to scalable metabolism: phosphoglycerate mutase (and following reactions), glycogen synthesis and phosphoketolase. Phosphoglycerate mutase regulates the crucial flux of carbon between the Calvin-Benson cycle and glycolysis (Fig. 1) and it plays a major role in biomass production^26^. A vast difference is noted in glycogen synthesis between HC and AC. HC grown cells of *Synechocystis* 6803 contain about 20-fold more glycogen than AC cells (9.1 versus 0.4 μg glycogen OD_750_^−1^ ml^−1^; own unpublished results). Furthermore, phosphoketolase allows by passing of glycolysis and thus prevents the loss of CO_2_ due to pyruvate dehydrogenase, a feature not required in HC. These differences suggest that the redistribution of carbon sources at different CO_2_ level exclusively occurs in a few cardinal nodes of metabolic crossroads such as phosphoglycerate mutase, glycogen synthesis and phosphoketolase, see Fig. 1 (red squares), whereas the vast majority of other fluxes is simply maintained at a constant ratio corresponding to changes in overall growth.

Another interesting result was found for the flux via glucose-6-phosphate dehydrogenase (G6PD), see Table 2. This flux is considered zero in published stoichiometric model^20^ because the involvement of G6PD does not allow a maximum efficiency of metabolism. It has been shown that the flux through G6PD leads to approximately 13% loss of total fixed carbon^11^. The flux via G6PD of approximately 16·10^−2^ mmol h^−1^gDW^−1^measured in HC^11^ indicates that this flux is not regulated by changes in gene expression in AC-shifted cells, because the weight factors for enzymatic activity based on the measured changes in the transcriptome are +8% for G6PD and +37% for phosphogluconate dehydrogenase^29^. These transcriptional changes directly translated into G6PD activity would result in simulated flux 10.3·10^−2^ mmol h^−1^gDW^−1^ via G6PD at AC conditions. This hypothetical high flux is very unlikely, since it did not allow to match the observed metabolic levels and it would led to further 34% decrease of the growth rate in AC. To obtain a good match between observed metabolite and growth changes with flux, it was necessary to modify the two values of weight factors from the transcriptional changes by newly estimated values of only 8% of the HC V_max_ for G6PD (i.e., 13.5-fold down-regulation) and 7% for phosphogluconate dehydrogenase (i.e., 19.6-fold down-regulation) (see Table 2) at AC. Because the previous study reported no reduction in enzymatic activities of G6PD or phosphogluconate dehydrogenase under photoautotrophic compared to heterotrophic conditions^37^, our findings support the idea that OPP cycle is primarily regulated by post-translational modifications.

Finally, our model provided significantly lower fluxes via phosphoglycerate mutase isozymes and the following part of glycolysis in AC cells compared with the published stoichiometric model^20^. This difference can be explained by taking into consideration that the stoichiometric model assumes zero flux via OPP cycle under this conditions^20^ (Table 2) and by the different goals of both approaches. Our multi-level kinetic modelling approach aims to match the experimental data, whereas stoichiometric modelling calculates the fluxes to achieve the theoretical maximal growth efficiency of cyanobacterial cells living in a steady state at never changing environment. Nevertheless, the complexity and vast amount of experimental data required for the multi-level kinetic model makes this approach extremely difficult and prone to error. Thus, a comparison with existing stoichiometric models is valuable and future combinations of the multi-level kinetic modelling and stoichiometric modelling should become a standard approach.

### Influence of gene expression on metabolic fluxes

Modelling metabolic levels is sensitive to changes of the kinetic parameters within the model. However, fluxes must also fulfil the mass conservation law–fluxes in linear chains of reactions are the same or lower and split in the branching points in dependence of the kinetic properties of enzymes. Therefore the question arises to what degree are the fluxes influenced by the network topology and how transcriptomic changes influence the kinetic parameters (V_max_). We have addressed this question by testing the sensitivity of fluxes against changes of the transcriptome for *Synechocystis* 6803 and *Synechococcus* 7942. For this purpose, we collected the measured changes in transcriptome^9^,^29^, for enzymes involved in our metabolic network, then shuffling them and randomly redistributing them as weight factors of enzymatic activities. Please note that analysis for *Synechococcus* 7942 was carried out by previous kinetic model^26^ with newly implemented calculation of fluxes as described here for model of *Synechocystis* 6803.

**Figure 3:**
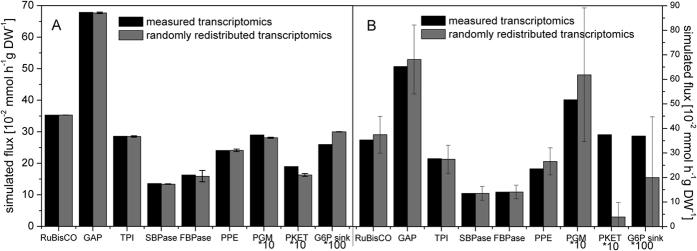
Comparison of the simulated fluxes at ambient CO_2_ based on the measured and randomly generated gene expression of specific enzymes for *Synechocystis* 6803 (**A**) and *Synechococcus* 7942 (**B**). **Black** represents a simulation of fluxes at AC based on the measured changes in gene expression after a shift from HC to AC. **Grey** indicates the mean of the simulated fluxes at AC based on 20 independent runs of randomly redistributed changes of measured gene expression, applied as weight factors for AC instead of measured changes. The error bars represent standard deviation. For better illustration, fluxes through PGM, PKET and the G6P sink are increased by factors of 10, 10 and 100, respectively. FBPase–shows the difference of fluxes between FBPase and PFK. Names of enzymes: RuBisCO– ribulose-1,5-bisphosphate carboxylase oxygenase, GAP–glyceraldehyde 3-phosphate dehydrogenase, TPI–triose-phosphate isomerase, SBPase– sedoheptulose-1,7-bisphosphate aldolase, FBPase–fructose-1,6 bisphosphatase, PFK–phosphofructokinase, PPE –phosphopentose epimerase, PGM–phosphoglycerate mutase, PKET–phosphoketolase, G6P sink–synthesis of carbohydrates.

To this end, our *in silico* experiment for *Synechocystis* 6803 reveals that randomly redistributed changes (20 independent simulations) in gene expression yield almost identical results in fluxes for intermediates within the Calvin-Benson cycle after shifts from HC to AC conditions (Fig. 3A) as found before using the measured changes in gene expression^29^. Moreover, if we focus on the fluxes out of the Calvin-Benson cycle, we observed some deviations among measured and calculated fluxes. In particular, the fluxes via phosphoglycerate mutase and phosphoketolase are affected. They became slightly lower after randomized gene expression changes compared with the fluxes based on measured mRNA levels (Fig. 3A). On the other hand, we can observe an opposite trend for glucose-6-phosphate sink (simplified synthesis of glycogen). All these differences correspond with expected redirection of carbon flux in cells at AC conditions. That means that gene expression changes can contribute to enhance the fluxes via phosphoglycerate mutase or phosphoketolase and to reduce the flux via glucose-6-phosphate sink (Fig. 3A). Taken together, the differences between both scenarios imply a potential minor impact of transcriptional regulation on carbon reallocation and major regulatory role of biochemical control for carbon metabolism in *Synechocystis* 6803.

Finally, the same analysis showed substantially different results for *Synechococcus* 7942. As already discussed above, the changes in transcriptome and thus the impact of their redistribution are significantly broader in *Synechococcus* 7942^9^ in comparison to *Synechocystis* 6803^29^. We observed very high standard deviations of flux rates after assuming random changes in the expression of certain enzymes (Fig. 3B). These deviations were particularly pronounced for the carbon redirection nodes: phosphoglycerate mutase, phosphoketolase and glucose-6-phosphate sink. There we observed 9.6-fold difference for phosphoketolase and 1.8-fold for glucose-6-phosphate sink between the fluxes based on measured and random changes in gene expression, respectively (Fig. 3B). The absence of similar high variability also in phosphoglycerate mutase is probably best explained by the existence of three isozymes of phosphoglycerate mutase in *Synechococcus* 7942^14^. Thus, these isozymes allow a robust biochemical regulation of this step in *Synechococcus* 7942 as was observed for many other reactions in *Synechocystis* 6803, which codes for corresponding more isozymes in its larger genome. Hence, we can conclude that the fluxes in AC on the metabolic crossroads, i.e., on the branching points, are mostly regulated by gene expression changes in the case of *Synechococcus* 7942 and by the action of different isozymes in *Synechocystis* 6803 (compare Fig. 3A,B).

## Materials and Methods

Comparison of transcriptomic changes was performed for genes encoding enzymes involved in central carbon metabolism after HC (5% CO_2_) to AC (0.04% CO_2_) shift from *Synechocystis* 6803 and *Synechococcus* 7942. The data were obtained from^8^ for *Synechococcus* 7942 and^29^ for *Synechocystis* 6803. The cyanobacterial strains were grown under almost identical conditions in the two studies and sampling of cells after the HC to AC shifts were done at exactly the same time points. The relative metabolomic data for *Synechocystis* 6803 cells grown in HC and AC conditions were obtained from^7^,^8^,^38^. The consideration of two environmental conditions was necessary to understand and implement the changes in the transcriptomic level of isozymes in the model and was essential for constraining the model. The measured fluxomics data were obtained from^11^, while simulated fluxes via stoichiometric modelling were obtained from^20^. The flux from the multi-level kinetic model was assumed to be equal to the rate of the particular reaction in the steady state.

The kinetic model approach prefers the absolute concentration of metabolites on the cellular level. Therefore, we recalculated the relative cellular metabolic concentrations given in the literature^7^,^8^,^38^ to absolute values from the ratios to the main CO_2_ fixation product 3PGA. The cellular concentration of 3PGA was calculated as 5 mM. This value was obtained exactly as described before for *Synechococcus* 7942^26^ and is similar to the measured concentration of 6.1 mM reported by^38^. We converted optical density as parameter reflecting biomass into cell volume via our calibration curve and assumed that 60% of the total cyanobacterial cell is represented by osmotic freely accessible cytoplasm^26^. The presented model describes the average population behavior of cyanobacteria and cannot distinguish any heterogeneity.

The multi-level kinetic model of *Synechocystis* 6803 for both high and ambient CO_2_ conditions is available in the Supplementary Model S1 (high CO_2_) and Supplementary Model S2 (ambient CO_2_). A summary of the V_max_ values estimated for *Synechocystis* 6803 cells grown in HC together with weight factors (fold changes in transcriptome after shifting from HC to AC) is provided in the Supplementary Note S3. The list of differential equations and rate equations (required for particular flux calculations and comparison with measured flux data) is available in the Supplementary Figure S4. The model for *Synechococcus* 7942 was previously described^26^.

### General information about the model

The multi-level kinetic model for *Synechocystis* 6803 was developed and simulations were executed using the SimBiology toolbox of MATLAB (The MathWorks, Inc., Natick, Massachusetts, United States of America). The routine for parameter estimation was a hybrid genetic algorithm. The model for both high and ambient CO_2_ conditions is available in the Supplemental Model S1 (high CO_2_) and Supplemental Model S2 (ambient CO_2_) in SBML format L2V4 compatible with MATLAB 2010b and higher. We recommend to open the model either in MATLAB or user friendly COPASI (free academic license).

The final model (Supplemental Model S1 for HC and Supplemental Model S2 for AC) was constrained by metabolic fluxes in HC, growth rate ratio between HC and AC and changes in enzyme quantity using the transcriptomics as well as metabolic levels under HC and AC. The model constraint on energy charge assumed the same ATP · (ADP + ATP)^−1^ ratio in HC and AC cells, which was maintained in a narrow range 0.74–0.78 as previously proposed^39^. The simulated growth ratio between HC and AC was 3.5 which is similar to experimental value 3.4 reported for *Synechocystis* 6803, e.g. ref. 40. As shown in Table 1, the comparisons of the simulated and experimental values correspond with each other. Unfortunately, some metabolic concentrations are only available for HC cells. We included these values to further constrain the parameter estimation.

### Multi-level kinetic model of *Synechocystis* 6803

A multi-level kinetic model of the central carbon metabolism of *Synechocystis* 6803 was generated to analyze the regulatory events and compare these events with *Synechococcus* 7942. The scope of the model includes the main parts of central carbon metabolism, such as the Calvin-Benson cycle, photorespiration, glycolysis, carbohydrate synthesis, and the OPP pathway. These metabolic reactions were coupled to simplified light reactions and Ci uptake as the main input parameters. Biomass production is one of the main output parameters (see Fig. 1), which is estimated as weighted sum of sink reactions. For example, the sink from ribose-5-phosphate for nucleotides synthesis (Fig. 1) contributes with 5 carbon molecules to biomass production. This simplified approach allowed to estimate the CO_2_ fixation under ambient CO_2_ level by comparing to relative changes in growth rate of cyanobacteria between HC and AC. The different growth rates are mean values of published data extracted from the previous experiments on HC to AC acclimations of *Synechocystis* 6803^8^,^28^ or *Synechococcus* 7942^9^. We assume that the biochemical composition of the two cyanobacterial species is rather similar. Moreover, the biomass in the model is supposed to be equal to the amount of fixed carbon, whereas nitrogen was not a limiting nutrient factor in the experimental setups^41^. The definition of biomass in the model is provided by following equation, based on the model content, in which upper index *s* stands for accumulation of respective metabolite in the sink (Fig. 1) and a particular coefficient corresponds to the amount of carbon atoms in the molecule:


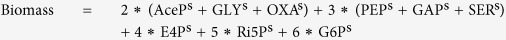


The model consists of 54 reactions, 36 metabolites and 182 kinetic parameters. The enzymatic reactions are described by Michaelis-Menten kinetics, except for the light reactions and Ci uptake, which are described by mass action kinetics. The equations for the Calvin-Benson cycle were obtained from previous studies on plant metabolism^23^,^42^, however, the kinetic parameters were changed (parameter estimation) to the specific conditions (metabolic and flux levels) of the cyanobacterial cell. All reactions, except for the import of external CO_2_, are localized in a single compartment.

The starting point of modelling *Synechocystis* 6803 was our existing model of *Synechococcus* 7942^26^, which was modified and extended because *Synechocystis* 6803 has an approximately 40% larger genome, including some differences in the number and specificity of enzymes for central carbon metabolism (discussed in more detail below). The initial sets of kinetic parameters were obtained from the model of carbon metabolism in C3 plants^42^,^43^ and from our model of *Synechococcus* 7942^26^.

### Kinetic parameters estimation

Initially, we assumed that the principal kinetic properties of the Calvin-Benson cycle are conserved among oxygenic phototrophs. However, the application of kinetic parameters from the previous models^26^,^42^ did not result in an acceptable match (up to 34-fold differences in metabolic level) between the calculated and measured metabolic data for *Synechocystis* 6803 cells in HC conditions^7^,^8^,^38^. Therefore, it was necessary to search for *Synechocystis* 6803-specific kinetic parameters. For this purpose, we employed a routine called parameter estimation requiring the Global optimization and Optimization toolboxes within MATLAB. As a search method for parameter estimation, the genetic algorithm was used, which mimics natural selection by employing processes as inheritance, mutation, selection and crossover within a population of candidate solutions. This process runs numerous iterations to minimize the measure of the distance among the model predictions and the experimental data. However, parameter estimation can yield several kinetic parameter sets, all offering a good match with the experimental data. To substantially constrain the process of parameter estimation, we added metabolic data from *Synechocystis* 6803 cells grown under AC conditions^7^,^8^.

The essential step in identifying the optimized kinetic parameters involves implementation of the connection between both data sets (HC and AC), which, in contrast to traditional kinetic modelling, enabled estimation of one set of kinetic parameters for metabolic reactions describing both HC and AC. To connect the two data sets, we used fold changes in the mRNA levels of genes for metabolic enzymes in the model after shifting from HC to AC^29^. These fold changes were used as weight factors of the enzymatic abundances applied on V_max_ of each reaction, assuming a 1:1 ratio between a change in transcriptome and enzymatic amount. This assumption is supported by several studies with bacterial cells, e.g., *E. coli*^44^ and salt acclimated cells of *Synechocystis* 6803^45^. Further support for this assumption was obtained by our results on glucose-6-phosphate dehydrogenase, which confirmed its post-translational modification (discussed below in the section *Is carbon metabolism scalable in response to a changing environment?*). Because the resulting model describes two environmental conditions (HC and AC) and implements the transcriptome as well as metabolome data, we refer to it as the multi-level kinetic model of *Synechocystis* 6803.

### Is it possible to further constrain the kinetic parameters?

Multi-level kinetic modeling approach represents a significant advancement in parameters constraining in comparison to traditional kinetic modeling. This advancement is based on using a single set of kinetic parameters for more than one system state, e.g., multiple environmental conditions. Moreover, energetic and redox limitations are usually neglected in kinetic modeling, but our approach includes these biological important parameters. Our multi-level kinetic model works with flexible concentrations of ATP and NADPH as these compounds play a crucial role in metabolic regulation within multi-level kinetic modeling^26^. Finally, we assume that prokaryotes evolved toward maximal growth efficiency (in the bounds and costs of adaptive mechanisms towards stress conditions). Thus, the maximal growth rate in HC together with growth rate ratio between HC and AC were used as additional constraining factors for parameter fits. Finally, our current approach includes fluxomic data, which revealed that the parameters estimation procedure itself is flawed in extraordinary cases. In order to match metabolic levels of F6P and G6P, fluxes via futile cycle phosphofructokinase–fructose-1,6-bisphosphatase were several times higher than physiologically possible, but could be corrected after incorporation of flux data. Therefore, including data from additional omics approaches may further improve the accuracy of kinetic parameters. Due to high constraints, we have found only one set of kinetic parameters that allowed matching the experimental data at HC and AC. However, multiple sets of parameter allowed comparable match with experiments on metabolic level after removing the constraint of maximal biomass requirement. Finally, modifications of certain parameters can be compensated to some extent by modifying other one, especially the couple of V_max_ and K_M_ for particular reaction if the substrate and product concentrations do not change significantly in HC and AC. Further model constraints as well as increased knowledge about metabolic regulation can be achieved by implementation of series of measurements based on single mutations (enforcing an adaptation of metabolic network), different light conditions, or other environmental factors affecting the central carbon metabolism.

### Systems biology workflow

The integration of various omics data occurred in several steps. In the first step, metabolic and fluxomics data from cells grown at high CO_2_ and ATP · (ADP + ATP)^−1^ ratio were used for identification of kinetic parameters. The next step included data from the high to ambient CO_2_ shift by applying the mean values of measured transcript changes as weight factors for each V_max_ estimated for HC. Then, the third step simulated a transition to steady state in AC, calculation of biomass and evaluates the match with metabolic data from cells grown at AC and the growth rate ratio between HC and AC states. The deviation between the simulated and experimental metabolic data could reach up to 100% at this stage. This boundary has been empirically chosen to obtain a broader set of results followed by manual curation and tuning of parameter sets. If no match was found after hundreds of parameter estimation runs made in the first step, the model was re-evaluated and missing reaction(s) or regulatory steps, e.g., isoenzyme or OPP, were implemented. Finally, if the match was improved to deviation below 100% (usually around 25%), the final tuning of kinetic parameters was initiated to improve the match between simulated and experimental data. This fine tuning was partially done by automatic parameter estimation routines and partially manually. Manual tuning and curation of kinetic parameters at the different levels of parameter estimation and data integration is an essential part of modelling, because it provides a deeper understanding of system behavior and helps to detect errors in the algorithms, routines and model structure.

## Conclusions

The main goal of this study is to achieve a mathematical description of the response of the central carbon metabolism to different inorganic carbon conditions in *Synechocystis* 6803, which could help to identify major regulatory processes. Our multi-level kinetic model approach allowed employing the same process of parameter estimation for *Synechocystis* 6803 as before for *Synechococcus* 7942 and resulted in a good match on the metabolic level. Furthermore, we demonstrate that the higher number of isozymes present in the larger *Synechocystis* 6803 genome compared with *Synechococcus* 7942 allow higher homeostatic stability on the metabolic level against changes in gene expression suggesting the possibility of a shift in metabolic regulation from genetic control in *Synechococcus* 7942 to enzymatic control in *Synechocystis* 6803. The only exception is G6PD, an enzyme from the OPP cycle that is active in photoautotrophic conditions as well as during the night. In this case, post-translational modifications are necessary to explain the regulation of G6PD.

To support the notion that the two cyanobacteria control central carbon metabolism on different regulatory levels, we analysed the impact of random changes in gene expression on the fluxes. Our simulation revealed that the AC-induced fluxes at the branching points of the metabolic network are highly dependent on transcriptional changes in *Synechococcus* 7942 but not in the case of *Synechocystis* 6803. These findings may be relevant for future attempts to use cyanobacteria for biotechnological purposes. Host strains with greater genomes such as *Synechocystis* 6803 might be more difficult to engineer toward changes in carbon allocation, since the metabolic network is rather stable due to biochemical regulation. Thus, the introduction of single branches or the up- or down-regulation of specific enzymes will have lower impact than in cyanobacterial hosts with lowered genome size such as *Synechococcus* 7942, in which transcriptional changes via different promoter strength or the expression of additional enzymes will have a bigger impact on the cellular carbon allocation. Finally, our results indicate that the multi-level kinetic modelling of cyanobacterial central carbon metabolism can be used to analyse and optimize cellular carbon reallocation towards specific needs in green biotechnology.

## Additional Information

**How to cite this article**: Jablonsky, J. *et al*. Different strategies of metabolic regulation in cyanobacteria: from transcriptional to biochemical control. *Sci. Rep.* **6**, 33024; doi: 10.1038/srep33024 (2016).

## Supplementary Material

Supplementary Information

## Figures and Tables

**Table 1:** Comparison between the simulated and measured metabolic concentrations in *Synechocystis* 6803 cells grown at high and ambient CO_2_.

high CO_2_	3PGA	2PGA	PEP	DHAP	F6P	G6P	FBP	S7P	unit	
experiment	4.99	2.58	2.06	0.08	0.37	0.23	0.08	1.20	mM	
simulation	4.79	3.15	2.58	0.11	0.2	0.15	0.14	1.19	mM	
difference	4.0	22.1	25.2	37.5	45.9	34.8	75.0	0.8	%	
**high CO**_**2**_	**2PG**	**GLY**	**SER**	**GCEA**	**P6G**	**Ri5P**	**Ru5P**	**RuBP**	**unit**	
experiment	1.42	1.30	0.48	0.03	0.30	0.06	0.13	0.09	mM	
simulation	1.17	1.22	0.52	0.026	0.25	0.04	0.08	0.07	mM	
difference	17.6	6.2	7.9	4.0	16.7	33.3	38.5	22.2	%	
**ambient CO**_**2**_	**3PGA**	**2PGA**	**PEP**	**DHAP**	**F6P**	**G6P**	**FBP**	**P6G**	**2PG**	**unit**
experiment	5.86	4.91	4.32	0.06	0.36	0.24	0.02	0.47	3.52	mM
simulation	6.53	4.6	3.95	0.04	0.15	0.12	0.02	0.2	4.18	mM
difference	11.4	6.3	8.6	32.2	58.3	50.0	20.0	57.4	18.8	%

3PGA–3-phosphoglycerate, 2PGA–2-phospohoglycerate, PEP–phosphoenolpyruvate, DHAP–dihydroxyacetone phosphate, F6P–fructose-6-phosphate, G6P–glucose-6-phosphate, FBP–fructose 1,6-bisphosphate, S7P–sedoheptulose-7-phosphate, 2PG–2-phosphoglycolate, GLY–glycine, SER–serine, GCEA –glycerate, P6G–6-phosphogluconate, Ri5P–ribose-5-phosphate, Ru5P–ribulose-5-phosphate, RuBP– ribulose-1,5-bisphosphate. The sources of experimental data: i) 3PGA, 2PGA, PEP, 2PG, GLY, SER^8^, ii) DHAP, F6P, G6P, FBP, P6G^7^, and iii) GCEA, S7P, Ri5P, Ru5P, RuBP^38^. All values were rounded to the nearest second decimal.

**Table 2:** Comparison of the estimated fluxes from ^13^C-labelling experiments, with calculated fluxes using the stoichiometric model and the multi-level kinetic model for *Synechocystis* 6803, respectively.

	RuBisCO	GAPDH	TPI	FBPase	ALDO	PPE	PGM	ENO	G6P sink	PKET	G6PD	Units
High CO_2_												
^13^C labelling	127	228	95	60	36	75.9	23.2	23.6	3	NA	16	10^−2^ mmol h^−1^ gDW^−1^
original FBA	126.9	230.2	98.3	50.1	47.7	81.6	18.4	18.4	1	14.9	0	10^−2^ mmol h^−1^ gDW^−1^
HC model	126.9	230.7	97	64.5	39.3	75.9	22.6	22.6	2.9	1.8	15.5	10^−2^ mmol h^−1^ gDW^−1^
Ambient CO_2_												
rescaled FBA	35.3	64.0	27.3	13.9	13.3	22.7	5.1	5.1	0.3	4.1	0	10^−2^ mmol h^−1^ gDW^−1^
AC model	35.3	67.9	28.6	16.3	13.6	24.1	2.9	2.9	0.27	1.9	1.1	10^−2^ mmol h^−1^ gDW^−1^

The upper part of the Table shows a comparison between the mean values of ^13^C-labelling experiment^11^, stoichiometric model^20^ and our multi-level kinetic model for the high CO_2_ (HC model) condition. For the purpose comparison at the ambient CO_2_ condition, we have scaled the original data from the stoichiometric model, i.e., divided all fluxes for high CO_2_ by a factor of 3.6. The AC model stands for the multi-level kinetic model for high CO_2_. FBPase– PFK shows the difference of fluxes between these two enzymes. Names of enzymes: RuBisCO– ribulose-1,5-bisphosphate carboxylase oxygenase, GAP–glyceraldehyde 3-phosphate dehydrogenase, TPI–triose phosphate isomerase, FBPase– fructose-1,6-bisphosphatase, PFK–phosphofructokinase, ALDO–aldolase, PPE –phosphopentose epimerase, PGM–phosphoglycerate mutase, ENO–enolase, G6P sink– synthesis of glycogen, PKET–phosphoketolase, G6PD–glucose-6-phosphate dehydrogenase.
