# Supplementary material for: Different strategies of metabolic regulation in cyanobacteria: from transcriptional to biochemical control
Source: Sci Rep. 2016 Sep 9;6:33024. doi: 10.1038/srep33024 (PMC5017163; doi:10.1038/srep33024)
Supplement: Supplementary Information [file srep33024-s1.pdf]

Supplementary Info File

Different strategies of metabolic regulation in cyanobacteria: from transcriptional to biochemical control

Jiri Jablonsky[1*], Stepan Papacek[1], Martin Hagemann[2]

1 Institute of Complex Systems, FFPW, University of South Bohemia, CENAKVA, Czech Republic

2 Department of Plant Physiology, University of Rostock, Einsteinstr. 3, D-18059 Rostock, Germany

[*]**Corresponding author**

Zamek 136, 373 33 Nove Hrady, Czech Republic

jiri.jablonsky@gmail.com

+420 607 187 176

Supplementary Note S3

Different strategies of metabolic regulation in cyanobacteria: from transcriptional to biochemical control - Supplementary Note S3

A summary of the V_{max} values estimated for *Synechocystis* 6803 cells grown in HC together with weight factors (fold changes in transcriptome after shifting from HC to LC)

Vm HC indicates the Vmax, estimated for high CO2 condition; this does not apply for the first three reactions which are described by mass action law and thus Vm HC has a meaning of rate constant. WF LC indicates transcriptomic data from cells of Synechocystis 6803 shifted from high to low CO2 atmosphere, obtained from a previous study (Hackenberg et al., 2012), representing the weight factors multiplying Vm for low CO2. RuBP – ribulose 1,5-bisphospate, 3PGA – 3-phosphoglycerate, BPGA – 1,3-bisphosphoglycerate, GAP – glyceraldehyde-3-phosphate, DHAP – dihydroxyacetone-phosphate, FBP – fructose-1,6-bisphosphate, F6P – fructose-6-phosphate, E4P – erythrose-4-phosphate, Xu5P – xylulose-5-phosphate, SBP – sedoheptulose-1,7-bisphosphate, S7P – sedoheptulose-7-phosphate, Ri5P – ribose-5-phosphate, Ru5P – ribulose-5-phosphate, 2PGA – 2-phospohoglycerate, PEP – phosphoenolpyruvate, 2PG – 2-phosphoglycolate, GCA – glycolate, GOA – glyoxylate, SER – serine, HPR – hydroxypyruvate, GLY – glycine, GCEA – glycerate, OXA – oxalate, TSA – tartronate-semialdehyde, G6P – glucose-6-phosphate, P6G – 6-phosphogluconate. NA indicates that the particular gene has not been yet annotated and thus the weight factor was estimated to 1.

model ID	reaction	Vm HC	WF LC
Light_1	ADP + Pi → ATP	0.16	0.25
Light_2	NADPp → NADPH	0.58	0.21
CO2 import	extCO2 → cytCO2	1.9	0.141
CC_1	RuBP + CO2 → 2*3PGA	0.991	0.9606
CC_2	3PGA + ATP → BPGA + ADP	0.6	0.86
CC_3	BPGA + NADPH ↔ GAP + NADPp + Pi	0.53	0.79
CC_4	GAP ↔ DHAP	0.17	0.746
CC_5	GAP + DHAP ↔ FBP	1.61	1.36
CC_6a	FBP → F6P + Pi	0.06	1.19
CC_6b	FBP → F6P + Pi	0.04	0.87
CC_7	F6P + GAP ↔ E4P + Xu5P	0.11	0.93
CC_8	DHAP + E4P ↔ SBP	1.11	1.36
CC_9	SBP → S7P + Pi	0.52	0.875
CC_10	S7P + GAP ↔ Ri5P + Xu5P	0.15	1.19
CC_11a	Ri5P ↔ Ru5P	1.1	0.96
CC_11b	Ri5P ↔ Ru5P	0.4	0.63
CC_12	Xu5P ↔ Ru5P	1.68	0.88
CC_13	Ru5P + ATP → RuBP + ADP	0.43	0.99
GL_1a	F6P → FBP	0.02	0.87
GL_1b	F6P → FBP	0.07	1.11
GL_2	GAP + NADPp + Pi ↔ BPGA + NADPH	1.24	0.79
GL_3a	3PGA ↔ 2PGA	0.24	1.12
GL_3b	3PGA ↔ 2PGA	0.71	1.17
GL_3c	3PGA ↔ 2PGA	0.027	0.78
GL_4	2PGA ↔ PEP	0.82	1.004
PP_1	RuBP + O2 → 2PG + 3PGA	0.009	5.3
PP_2a	2PG → GCA	0.0007	0.84
PP_2b	2PG → GCA	0.0005	0.78
PP_2c	2PG → GCA	0.001	1.003
PP_3	GCA → GOA	0.0042	2
PP_4	GOA + SER ↔ HPR + GLY	0.43	NA
PP_5	2*GLY → SER	0.002	0.82
PP_6	HPR → GCEA	0.004	NA
PP_7	GCEA + ATP → 2PGA + ADP	0.013	NA
OX_1	GOA → OXA	0.12	0.8
GC_1	GOA → TSA	0.01	NA
GC_2	TSA → GCEA	0.1	NA
GSM_1	3PGA → SER	0.0008	NA
GSM_2	GOA ↔ GLY	0.0002	NA
SS_1	F6P ↔ G6P	0.99	0.63
PPP_1	G6P → P6G	0.02	0.08
PPP_2	P6G + NADPp → Ru5P + NADPH	0.01	0.07
PKETa	F6P → E4P + AceP	0.015	1.35
PKETb	F6P → E4P + AceP	0.0005	1.1
Sink_X	DHAP, E4P, Ri5P, PPE, AceP, SER ... → Sink	0 - 0.03	NA

model ID	pathway	enzyme
Light_1	light reactions	ATP synthase
Light_2	light reactions	simplified reaction
CO2 import	CO2 transport	simplified carboxysome
CC_1	Calvin-Benson cycle	RuBisCO
CC_2	Calvin-Benson cycle	phosphoglycerate kinase
CC_3	Calvin-Benson cycle	glyceraldehyde 3-phosphate dehydrogenase
CC_4	Calvin-Benson cycle	triose phosphate isomerase
CC_5	Calvin-Benson cycle	aldolase
CC_6a	Calvin-Benson cycle	fructose-1,6-bisphosphatase
CC_6b	Calvin-Benson cycle	fructose-1,6-bisphosphatase
CC_7	Calvin-Benson cycle	transketolase
CC_8	Calvin-Benson cycle	aldolase
CC_9	Calvin-Benson cycle	sedoheptulose-1,7 bisphosphatase
CC_10	Calvin-Benson cycle	transketolase
CC_11a	Calvin-Benson cycle	phosphopentose isomerase
CC_11b	Calvin-Benson cycle	phosphopentose isomerase
CC_12	Calvin-Benson cycle	phosphopentose epimerase
CC_13	Calvin-Benson cycle	phosphoribulokinase
GL_1a	Glycolysis	phosphofructokinase
GL_1b	Glycolysis	phosphofructokinase
GL_2	Glycolysis	glyceraldehyde 3-phosphate dehydrogenase
GL_3a	Glycolysis	phosphoglycerate mutase
GL_3b	Glycolysis	phosphoglycerate mutase
GL_3c	Glycolysis	phosphoglycerate mutase
GL_4	Glycolysis	enolase
PP_1	Photorespiration	RuBisCO
PP_2a	Photorespiration	phosphoglycolate phosphatase
PP_2b	Photorespiration	phosphoglycolate phosphatase
PP_2c	Photorespiration	phosphoglycolate phosphatase
PP_3	Photorespiration	glycolate oxidase
PP_4	Photorespiration	serineglyoxylate transaminase
PP_5	Photorespiration	serine hydroxymethyltransferase
PP_6	Photorespiration	hydroxypyruvate reductase
PP_7	Photorespiration	glycerate kinase
OX_1	oxalate pathway	glyoxylate oxidase
GC_1	glycerate pathway	tartronate semialdehyde synthase
GC_2	glycerate pathway	tartronate semialdehyde reductase
GSM_1	GLY, SER metabolism	simplified phosphoserine transaminase
GSM_2	GLY, SER metabolism	glycine transaminase
SS_1	carbohydrates synthesis	G6P sink (glycogen synthesis)
PPP_1	pentose phosphate pathway	glucose-6-phosphate dehydrogenase
PPP_2	pentose phosphate pathway	phosphogluconate dehydrogenase
PKETa	bypass of glycolysis	phosphoketolase
PKETb	bypass of glycolysis	phosphoketolase
Sink_X	adjacent pathways for biomass	sum of sinks for biomass production estimate

Supplementary Figure S4

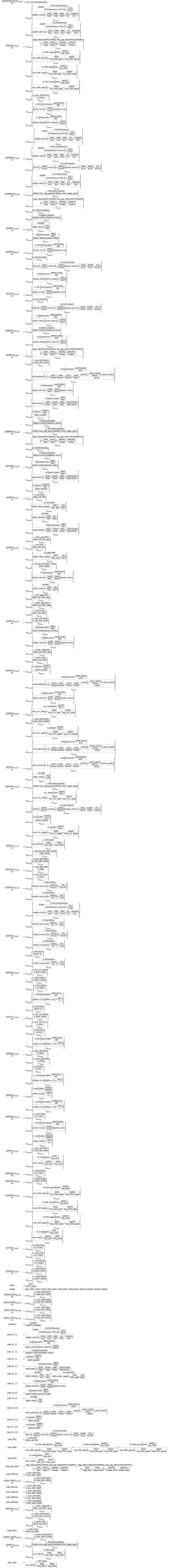

Supplemental File S1 Model


```
<?xml version="1.0" encoding="UTF-8"?>

<sbml xmlns="http://www.sbml.org/sbml/level2" xmlns:html="http://www.w3.org/1999/xhtml"
level="2" version="1">

 <annotation>

Created by The MathWorks, Inc. SimBiology tool, Version 3.3

</annotation>

 <model id="Model_1" name="initial">

  <notes>

   <body xmlns="http://www.w3.org/1999/xhtml"></body>

  </notes>

  <annotation>

   <COPASI xmlns="http://www.copasi.org/static/sbml">

    <rdf:RDF xmlns:dcterms="http://purl.org/dc/terms/"
xmlns:rdf="http://www.w3.org/1999/02/22-rdf-syntax-ns#">

     <rdf:Description rdf:about="#COPASI1">

      <dcterms:created>

       <rdf:Description>

        <dcterms:W3CDTF>2010-04-14T14:47:02Z</dcterms:W3CDTF>

       </rdf:Description>

      </dcterms:created>

     </rdf:Description>

    </rdf:RDF>

   </COPASI>

  </annotation>

  <listOfCompartments>

   <compartment id="mwc7a0146b_dcaf_459b_8aad_1ff6e20a3fb5" name="Stroma" size="1">

    <annotation>

     <COPASI xmlns="http://www.copasi.org/static/sbml">
```

```
<rdf:RDF xmlns:dcterms="http://purl.org/dc/terms/" xmlns:rdf="http://www.w3.org/1999/02/22-rdf-syntax-ns#">
<rdf:Description rdf:about="#COPASI2">
<dcterms:created>
<rdf:Description>
<dcterms:W3CDTF>2010-04-14T14:56:43Z</dcterms:W3CDTF>
</rdf:Description>
</dcterms:created>
</rdf:Description>
</rdf:RDF>
</COPASI>
</annotation>
</compartment>
<compartment id="mw1341fc13_2fe2_46f4_b24f_cd32c24b8dca" name="external" size="1"/>
<compartment id="mw99892ac6_be79_4769_b284_4c503a78023d" name="Stroma Stroma" size="1"/>
</listOfCompartments>
<listOfSpecies>
<species id="species_1" name="CO2" compartment="mwc7a0146b_dcaf_459b_8aad_1ff6e20a3fb5" initialAmount="0.02">
<annotation>
<COPASI xmlns="http://www.copasi.org/static/sbml">
<rdf:RDF xmlns:dcterms="http://purl.org/dc/terms/" xmlns:rdf="http://www.w3.org/1999/02/22-rdf-syntax-ns#">
<rdf:Description rdf:about="#COPASI3">
<dcterms:created>
<rdf:Description>
<dcterms:W3CDTF>2010-04-14T15:46:19Z</dcterms:W3CDTF>
</rdf:Description>
```

```xml
              </dcterms:created>
            </rdf:Description>
          </rdf:RDF>
        </COPASI>
      </annotation>
    </species>
    <species id="species_2" name="PGA"
compartment="mwc7a0146b_dcaf_459b_8aad_1ff6e20a3fb5" initialAmount="4.7">
      <annotation>
        <COPASI xmlns="http://www.copasi.org/static/sbml">
          <rdf:RDF xmlns:dcterms="http://purl.org/dc/terms/"
xmlns:rdf="http://www.w3.org/1999/02/22-rdf-syntax-ns#">
            <rdf:Description rdf:about="#COPASI4">
              <dcterms:created>
                <rdf:Description>
                  <dcterms:W3CDTF>2010-04-14T15:47:21Z</dcterms:W3CDTF>
                </rdf:Description>
              </dcterms:created>
            </rdf:Description>
          </rdf:RDF>
        </COPASI>
      </annotation>
    </species>
    <species id="species_3" name="RuBP"
compartment="mwc7a0146b_dcaf_459b_8aad_1ff6e20a3fb5" initialAmount="1">
      <annotation>
        <COPASI xmlns="http://www.copasi.org/static/sbml">
          <rdf:RDF xmlns:dcterms="http://purl.org/dc/terms/"
xmlns:rdf="http://www.w3.org/1999/02/22-rdf-syntax-ns#">
```

<rdf:Description rdf:about="#COPASI5">
<dcterms:created>
<rdf:Description>
<dcterms:W3CDTF>2010-04-14T15:47:38Z</dcterms:W3CDTF>
</rdf:Description>
</dcterms:created>
</rdf:Description>
</rdf:RDF>
</COPASI>
</annotation>
</species>
<species id="species_5" name="NADPH" compartment="mwc7a0146b_dcaf_459b_8aad_1ff6e20a3fb5" initialAmount="0.148995513447536">
<annotation>
<COPASI xmlns="http://www.copasi.org/static/sbml">
<rdf:RDF xmlns:dcterms="http://purl.org/dc/terms/" xmlns:rdf="http://www.w3.org/1999/02/22-rdf-syntax-ns#">
<rdf:Description rdf:about="#COPASI6">
<dcterms:created>
<rdf:Description>
<dcterms:W3CDTF>2010-04-14T15:47:02Z</dcterms:W3CDTF>
</rdf:Description>
</dcterms:created>
</rdf:Description>
</rdf:RDF>
</COPASI>
</annotation>
</species>

<species id="species_6" name="O2"
compartment="mwc7a0146b_dcaf_459b_8aad_1ff6e20a3fb5"
initialAmount="0.0259999987479538" boundaryCondition="true" constant="true">

<annotation>

<COPASI xmlns="http://www.copasi.org/static/sbml">

<rdf:RDF xmlns:dcterms="http://purl.org/dc/terms/"
xmlns:rdf="http://www.w3.org/1999/02/22-rdf-syntax-ns#">

<rdf:Description rdf:about="#COPASI7">

<dcterms:created>

<rdf:Description>

<dcterms:W3CDTF>2010-04-14T15:47:13Z</dcterms:W3CDTF>

</rdf:Description>

</dcterms:created>

</rdf:Description>

</rdf:RDF>

</COPASI>

</annotation>

</species>

<species id="species_8" name="SBP"
compartment="mwc7a0146b_dcaf_459b_8aad_1ff6e20a3fb5" initialAmount="1.36261887752912">

<annotation>

<COPASI xmlns="http://www.copasi.org/static/sbml">

<rdf:RDF xmlns:dcterms="http://purl.org/dc/terms/"
xmlns:rdf="http://www.w3.org/1999/02/22-rdf-syntax-ns#">

<rdf:Description rdf:about="#COPASI8">

<dcterms:created>

<rdf:Description>

<dcterms:W3CDTF>2010-04-14T15:47:55Z</dcterms:W3CDTF>

</rdf:Description>

</dcterms:created>

</rdf:Description>

</rdf:RDF>

</COPASI>

</annotation>

</species>

<species id="species_4" name="ADP" compartment="mwc7a0146b_dcaf_459b_8aad_1ff6e20a3fb5" initialAmount="0.344798607704336">

<annotation>

<COPASI xmlns="http://www.copasi.org/static/sbml">

<rdf:RDF xmlns:dcterms="http://purl.org/dc/terms/" xmlns:rdf="http://www.w3.org/1999/02/22-rdf-syntax-ns#">

<rdf:Description rdf:about="#COPASI9">

<dcterms:created>

<rdf:Description>

<dcterms:W3CDTF>2010-04-14T16:01:25Z</dcterms:W3CDTF>

</rdf:Description>

</dcterms:created>

</rdf:Description>

</rdf:RDF>

</COPASI>

</annotation>

</species>

<species id="species_7" name="ATP" compartment="mwc7a0146b_dcaf_459b_8aad_1ff6e20a3fb5" initialAmount="1.15520132006222">

<annotation>

<COPASI xmlns="http://www.copasi.org/static/sbml">

<rdf:RDF xmlns:dcterms="http://purl.org/dc/terms/" xmlns:rdf="http://www.w3.org/1999/02/22-rdf-syntax-ns#">

<rdf:Description rdf:about="#COPASI10">

<dcterms:created>

<rdf:Description>

<dcterms:W3CDTF>2010-04-14T16:01:14Z</dcterms:W3CDTF>

</rdf:Description>

</dcterms:created>

</rdf:Description>

</rdf:RDF>

</COPASI>

</annotation>

</species>

<species id="species_9" name="BPGA" compartment="mwc7a0146b_dcaf_459b_8aad_1ff6e20a3fb5" initialAmount="0.00748248557057663">

<annotation>

<COPASI xmlns="http://www.copasi.org/static/sbml">

<rdf:RDF xmlns:dcterms="http://purl.org/dc/terms/" xmlns:rdf="http://www.w3.org/1999/02/22-rdf-syntax-ns#">

<rdf:Description rdf:about="#COPASI11">

<dcterms:created>

<rdf:Description>

<dcterms:W3CDTF>2010-04-14T16:01:08Z</dcterms:W3CDTF>

</rdf:Description>

</dcterms:created>

</rdf:Description>

</rdf:RDF>

</COPASI>

```
</annotation>

</species>

<species id="species_10" name="GAP"
compartment="mwc7a0146b_dcaf_459b_8aad_1ff6e20a3fb5"
initialAmount="0.368362636458716">

<annotation>

<COPASI xmlns="http://www.copasi.org/static/sbml">

<rdf:RDF xmlns:dcterms="http://purl.org/dc/terms/"
xmlns:rdf="http://www.w3.org/1999/02/22-rdf-syntax-ns#">

<rdf:Description rdf:about="#COPASI12">

<dcterms:created>

<rdf:Description>

<dcterms:W3CDTF>2010-06-08T23:46:46Z</dcterms:W3CDTF>

</rdf:Description>

</dcterms:created>

</rdf:Description>

</rdf:RDF>

</COPASI>

</annotation>

</species>

<species id="species_11" name="NADPp"
compartment="mwc7a0146b_dcaf_459b_8aad_1ff6e20a3fb5"
initialAmount="0.271004466327097">

<annotation>

<COPASI xmlns="http://www.copasi.org/static/sbml">

<rdf:RDF xmlns:dcterms="http://purl.org/dc/terms/"
xmlns:rdf="http://www.w3.org/1999/02/22-rdf-syntax-ns#">

<rdf:Description rdf:about="#COPASI13">

<dcterms:created>

<rdf:Description>
```

<dcterms:W3CDTF>2010-06-08T23:43:15Z</dcterms:W3CDTF>

</rdf:Description>

</dcterms:created>

</rdf:Description>

</rdf:RDF>

</COPASI>

</annotation>

</species>

<species id="species_12" name="Pi" compartment="mwc7a0146b_dcaf_459b_8aad_1ff6e20a3fb5" initialAmount="4.99999975922188" boundaryCondition="true" constant="true">

<annotation>

<COPASI xmlns="http://www.copasi.org/static/sbml">

<rdf:RDF xmlns:dcterms="http://purl.org/dc/terms/" xmlns:rdf="http://www.w3.org/1999/02/22-rdf-syntax-ns#">

<rdf:Description rdf:about="#COPASI14">

<dcterms:created>

<rdf:Description>

<dcterms:W3CDTF>2010-05-18T18:10:26Z</dcterms:W3CDTF>

</rdf:Description>

</dcterms:created>

</rdf:Description>

</rdf:RDF>

</COPASI>

</annotation>

</species>

<species id="species_13" name="DHAP" compartment="mwc7a0146b_dcaf_459b_8aad_1ff6e20a3fb5" initialAmount="0.012">

<annotation>

<COPASI xmlns="http://www.copasi.org/static/sbml">
<rdf:RDF xmlns:dcterms="http://purl.org/dc/terms/" xmlns:rdf="http://www.w3.org/1999/02/22-rdf-syntax-ns#">
<rdf:Description rdf:about="#COPASI15">
<dcterms:created>
<rdf:Description>
<dcterms:W3CDTF>2010-06-08T23:43:34Z</dcterms:W3CDTF>
</rdf:Description>
</dcterms:created>
</rdf:Description>
</rdf:RDF>
</COPASI>
</annotation>
</species>
<species id="species_14" name="FBP" compartment="mwc7a0146b_dcaf_459b_8aad_1ff6e20a3fb5" initialAmount="0.02">
<annotation>
<COPASI xmlns="http://www.copasi.org/static/sbml">
<rdf:RDF xmlns:dcterms="http://purl.org/dc/terms/" xmlns:rdf="http://www.w3.org/1999/02/22-rdf-syntax-ns#">
<rdf:Description rdf:about="#COPASI16">
<dcterms:created>
<rdf:Description>
<dcterms:W3CDTF>2010-06-08T23:44:30Z</dcterms:W3CDTF>
</rdf:Description>
</dcterms:created>
</rdf:Description>
</rdf:RDF>
</COPASI>

</annotation>

</species>

<species id="species_15" name="F6P" compartment="mwc7a0146b_dcaf_459b_8aad_1ff6e20a3fb5" initialAmount="2">

<annotation>

<COPASI xmlns="http://www.copasi.org/static/sbml">

<rdf:RDF xmlns:dcterms="http://purl.org/dc/terms/" xmlns:rdf="http://www.w3.org/1999/02/22-rdf-syntax-ns#">

<rdf:Description rdf:about="#COPASI17">

<dcterms:created>

<rdf:Description>

<dcterms:W3CDTF>2010-06-08T23:44:21Z</dcterms:W3CDTF>

</rdf:Description>

</dcterms:created>

</rdf:Description>

</rdf:RDF>

</COPASI>

</annotation>

</species>

<species id="species_16" name="E4P" compartment="mwc7a0146b_dcaf_459b_8aad_1ff6e20a3fb5" initialAmount="0.228820226685508">

<annotation>

<COPASI xmlns="http://www.copasi.org/static/sbml">

<rdf:RDF xmlns:dcterms="http://purl.org/dc/terms/" xmlns:rdf="http://www.w3.org/1999/02/22-rdf-syntax-ns#">

<rdf:Description rdf:about="#COPASI18">

<dcterms:created>

<rdf:Description>

```
              <dcterms:W3CDTF>2010-06-08T23:43:55Z</dcterms:W3CDTF>
            </rdf:Description>
          </dcterms:created>
        </rdf:Description>
      </rdf:RDF>
    </COPASI>
  </annotation>
</species>
<species id="species_17" name="Xu5P"
compartment="mwc7a0146b_dcaf_459b_8aad_1ff6e20a3fb5"
initialAmount="0.0558077870361159">
  <annotation>
    <COPASI xmlns="http://www.copasi.org/static/sbml">
      <rdf:RDF xmlns:dcterms="http://purl.org/dc/terms/"
xmlns:rdf="http://www.w3.org/1999/02/22-rdf-syntax-ns#">
        <rdf:Description rdf:about="#COPASI19">
          <dcterms:created>
            <rdf:Description>
              <dcterms:W3CDTF>2010-06-08T23:41:49Z</dcterms:W3CDTF>
            </rdf:Description>
          </dcterms:created>
        </rdf:Description>
      </rdf:RDF>
    </COPASI>
  </annotation>
</species>
<species id="species_18" name="Ri5P"
compartment="mwc7a0146b_dcaf_459b_8aad_1ff6e20a3fb5"
initialAmount="0.0744045363011372">
```

```
<annotation>
  <COPASI xmlns="http://www.copasi.org/static/sbml">
    <rdf:RDF xmlns:dcterms="http://purl.org/dc/terms/"
xmlns:rdf="http://www.w3.org/1999/02/22-rdf-syntax-ns#">
      <rdf:Description rdf:about="#COPASI20">
        <dcterms:created>
          <rdf:Description>
            <dcterms:W3CDTF>2010-06-08T23:41:08Z</dcterms:W3CDTF>
          </rdf:Description>
        </dcterms:created>
      </rdf:Description>
    </rdf:RDF>
  </COPASI>
</annotation>
</species>
<species id="species_19" name="S7P"
compartment="mwc7a0146b_dcaf_459b_8aad_1ff6e20a3fb5"
initialAmount="0.511319423085088">
<annotation>
  <COPASI xmlns="http://www.copasi.org/static/sbml">
    <rdf:RDF xmlns:dcterms="http://purl.org/dc/terms/"
xmlns:rdf="http://www.w3.org/1999/02/22-rdf-syntax-ns#">
      <rdf:Description rdf:about="#COPASI21">
        <dcterms:created>
          <rdf:Description>
            <dcterms:W3CDTF>2010-06-08T23:47:56Z</dcterms:W3CDTF>
          </rdf:Description>
        </dcterms:created>
      </rdf:Description>
```

</rdf:RDF>

</COPASI>

</annotation>

</species>

<species id="species_20" name="Ru5P" compartment="mwc7a0146b_dcaf_459b_8aad_1ff6e20a3fb5" initialAmount="0.026898222838205">

<annotation>

<COPASI xmlns="http://www.copasi.org/static/sbml">

<rdf:RDF xmlns:dcterms="http://purl.org/dc/terms/" xmlns:rdf="http://www.w3.org/1999/02/22-rdf-syntax-ns#">

<rdf:Description rdf:about="#COPASI22">

<dcterms:created>

<rdf:Description>

<dcterms:W3CDTF>2010-06-08T23:41:17Z</dcterms:W3CDTF>

</rdf:Description>

</dcterms:created>

</rdf:Description>

</rdf:RDF>

</COPASI>

</annotation>

</species>

<species id="species_21" name="G6P" compartment="mwc7a0146b_dcaf_459b_8aad_1ff6e20a3fb5" initialAmount="1.94057630985951">

<annotation>

<COPASI xmlns="http://www.copasi.org/static/sbml">

<rdf:RDF xmlns:dcterms="http://purl.org/dc/terms/" xmlns:rdf="http://www.w3.org/1999/02/22-rdf-syntax-ns#">

<rdf:Description rdf:about="#COPASI23">

```
            <dcterms:created>
              <rdf:Description>
                <dcterms:W3CDTF>2010-06-08T23:46:39Z</dcterms:W3CDTF>
              </rdf:Description>
            </dcterms:created>
          </rdf:Description>
        </rdf:RDF>
      </COPASI>
    </annotation>
  </species>
  <species id="species_23" name="Sink1"
compartment="mwc7a0146b_dcaf_459b_8aad_1ff6e20a3fb5" initialAmount="0">
    <annotation>
      <COPASI xmlns="http://www.copasi.org/static/sbml">
        <rdf:RDF xmlns:dcterms="http://purl.org/dc/terms/"
xmlns:rdf="http://www.w3.org/1999/02/22-rdf-syntax-ns#">
          <rdf:Description rdf:about="#COPASI25">
            <dcterms:created>
              <rdf:Description>
                <dcterms:W3CDTF>2011-05-10T00:14:21Z</dcterms:W3CDTF>
              </rdf:Description>
            </dcterms:created>
          </rdf:Description>
        </rdf:RDF>
      </COPASI>
    </annotation>
  </species>
  <species id="species_24" name="Sink2"
compartment="mwc7a0146b_dcaf_459b_8aad_1ff6e20a3fb5" initialAmount="0">
```

```
<annotation>
<COPASI xmlns="http://www.copasi.org/static/sbml">
<rdf:RDF xmlns:dcterms="http://purl.org/dc/terms/"
xmlns:rdf="http://www.w3.org/1999/02/22-rdf-syntax-ns#">
<rdf:Description rdf:about="#COPASI26">
<dcterms:created>
<rdf:Description>
<dcterms:W3CDTF>2011-04-26T00:48:45Z</dcterms:W3CDTF>
</rdf:Description>
</dcterms:created>
</rdf:Description>
</rdf:RDF>
</COPASI>
</annotation>
</species>
<species id="species_25" name="PGCA"
compartment="mwc7a0146b_dcaf_459b_8aad_1ff6e20a3fb5" initialAmount="0.01">
<annotation>
<COPASI xmlns="http://www.copasi.org/static/sbml">
<rdf:RDF xmlns:dcterms="http://purl.org/dc/terms/"
xmlns:rdf="http://www.w3.org/1999/02/22-rdf-syntax-ns#">
<rdf:Description rdf:about="#COPASI27">
<dcterms:created>
<rdf:Description>
<dcterms:W3CDTF>2011-06-15T16:28:14Z</dcterms:W3CDTF>
</rdf:Description>
</dcterms:created>
</rdf:Description>
</rdf:RDF>
```

</COPASI>

</annotation>

</species>

<species id="species_26" name="GCA" compartment="mwc7a0146b_dcaf_459b_8aad_1ff6e20a3fb5" initialAmount="0.0123593835433703">

<annotation>

<COPASI xmlns="http://www.copasi.org/static/sbml">

<rdf:RDF xmlns:dcterms="http://purl.org/dc/terms/" xmlns:rdf="http://www.w3.org/1999/02/22-rdf-syntax-ns#">

<rdf:Description rdf:about="#COPASI28">

<dcterms:created>

<rdf:Description>

<dcterms:W3CDTF>2011-06-15T16:28:15Z</dcterms:W3CDTF>

</rdf:Description>

</dcterms:created>

</rdf:Description>

</rdf:RDF>

</COPASI>

</annotation>

</species>

<species id="species_27" name="GOA" compartment="mwc7a0146b_dcaf_459b_8aad_1ff6e20a3fb5" initialAmount="0.0327363830028097">

<annotation>

<COPASI xmlns="http://www.copasi.org/static/sbml">

<rdf:RDF xmlns:dcterms="http://purl.org/dc/terms/" xmlns:rdf="http://www.w3.org/1999/02/22-rdf-syntax-ns#">

<rdf:Description rdf:about="#COPASI29">

<dcterms:created>

<rdf:Description>

<dcterms:W3CDTF>2011-06-15T16:28:23Z</dcterms:W3CDTF>

</rdf:Description>

</dcterms:created>

</rdf:Description>

</rdf:RDF>

</COPASI>

</annotation>

</species>

<species id="species_28" name="GLY" compartment="mwc7a0146b_dcaf_459b_8aad_1ff6e20a3fb5" initialAmount="0.8">

<annotation>

<COPASI xmlns="http://www.copasi.org/static/sbml">

<rdf:RDF xmlns:dcterms="http://purl.org/dc/terms/" xmlns:rdf="http://www.w3.org/1999/02/22-rdf-syntax-ns#">

<rdf:Description rdf:about="#COPASI30">

<dcterms:created>

<rdf:Description>

<dcterms:W3CDTF>2011-06-15T16:27:59Z</dcterms:W3CDTF>

</rdf:Description>

</dcterms:created>

</rdf:Description>

</rdf:RDF>

</COPASI>

</annotation>

</species>

<species id="species_29" name="SER" compartment="mwc7a0146b_dcaf_459b_8aad_1ff6e20a3fb5" initialAmount="0.5">

<annotation>

```
        <COPASI xmlns="http://www.copasi.org/static/sbml">
          <rdf:RDF xmlns:dcterms="http://purl.org/dc/terms/" xmlns:rdf="http://www.w3.org/1999/02/22-rdf-syntax-ns#">
            <rdf:Description rdf:about="#COPASI31">
              <dcterms:created>
                <rdf:Description>
                  <dcterms:W3CDTF>2011-06-15T16:28:35Z</dcterms:W3CDTF>
                </rdf:Description>
              </dcterms:created>
            </rdf:Description>
          </rdf:RDF>
        </COPASI>
      </annotation>
    </species>
    <species id="species_30" name="HPR" compartment="mwc7a0146b_dcaf_459b_8aad_1ff6e20a3fb5" initialAmount="0.1">
      <annotation>
        <COPASI xmlns="http://www.copasi.org/static/sbml">
          <rdf:RDF xmlns:dcterms="http://purl.org/dc/terms/" xmlns:rdf="http://www.w3.org/1999/02/22-rdf-syntax-ns#">
            <rdf:Description rdf:about="#COPASI32">
              <dcterms:created>
                <rdf:Description>
                  <dcterms:W3CDTF>2011-06-15T16:38:48Z</dcterms:W3CDTF>
                </rdf:Description>
              </dcterms:created>
            </rdf:Description>
          </rdf:RDF>
        </COPASI>
```

</annotation>

</species>

<species id="species_31" name="GCEA" compartment="mwc7a0146b_dcaf_459b_8aad_1ff6e20a3fb5" initialAmount="0.00400097153227282">

<annotation>

<COPASI xmlns="http://www.copasi.org/static/sbml">

<rdf:RDF xmlns:dcterms="http://purl.org/dc/terms/" xmlns:rdf="http://www.w3.org/1999/02/22-rdf-syntax-ns#">

<rdf:Description rdf:about="#COPASI33">

<dcterms:created>

<rdf:Description>

<dcterms:W3CDTF>2011-06-15T16:30:28Z</dcterms:W3CDTF>

</rdf:Description>

</dcterms:created>

</rdf:Description>

</rdf:RDF>

</COPASI>

</annotation>

</species>

<species id="species_32" name="PEP" compartment="mwc7a0146b_dcaf_459b_8aad_1ff6e20a3fb5" initialAmount="4.19091918967107">

<annotation>

<COPASI xmlns="http://www.copasi.org/static/sbml">

<rdf:RDF xmlns:dcterms="http://purl.org/dc/terms/" xmlns:rdf="http://www.w3.org/1999/02/22-rdf-syntax-ns#">

<rdf:Description rdf:about="#COPASI34">

<dcterms:created>

<rdf:Description>

<dcterms:W3CDTF>2011-05-10T16:27:35Z</dcterms:W3CDTF>

</rdf:Description>

</dcterms:created>

</rdf:Description>

</rdf:RDF>

</COPASI>

</annotation>

</species>

<species id="species_33" name="Sink3" compartment="mwc7a0146b_dcaf_459b_8aad_1ff6e20a3fb5" initialAmount="0">

<annotation>

<COPASI xmlns="http://www.copasi.org/static/sbml">

<rdf:RDF xmlns:dcterms="http://purl.org/dc/terms/" xmlns:rdf="http://www.w3.org/1999/02/22-rdf-syntax-ns#">

<rdf:Description rdf:about="#COPASI35">

<dcterms:created>

<rdf:Description>

<dcterms:W3CDTF>2011-05-10T16:30:50Z</dcterms:W3CDTF>

</rdf:Description>

</dcterms:created>

</rdf:Description>

</rdf:RDF>

</COPASI>

</annotation>

</species>

<species id="species_34" name="Sink4" compartment="mwc7a0146b_dcaf_459b_8aad_1ff6e20a3fb5" initialAmount="0">

<annotation>

<COPASI xmlns="http://www.copasi.org/static/sbml">

<rdf:RDF xmlns:dcterms="http://purl.org/dc/terms/" xmlns:rdf="http://www.w3.org/1999/02/22-rdf-syntax-ns#">

<rdf:Description rdf:about="#COPASI36">

<dcterms:created>

<rdf:Description>

<dcterms:W3CDTF>2011-06-15T16:28:43Z</dcterms:W3CDTF>

</rdf:Description>

</dcterms:created>

</rdf:Description>

</rdf:RDF>

</COPASI>

</annotation>

</species>

<species id="species_35" name="ppGA" compartment="mwc7a0146b_dcaf_459b_8aad_1ff6e20a3fb5" initialAmount="4.19091917967427">

<annotation>

<COPASI xmlns="http://www.copasi.org/static/sbml">

<rdf:RDF xmlns:dcterms="http://purl.org/dc/terms/" xmlns:rdf="http://www.w3.org/1999/02/22-rdf-syntax-ns#">

<rdf:Description rdf:about="#COPASI37">

<dcterms:created>

<rdf:Description>

<dcterms:W3CDTF>2011-06-15T16:30:09Z</dcterms:W3CDTF>

</rdf:Description>

</dcterms:created>

</rdf:Description>

</rdf:RDF>

</COPASI>

</annotation>

</species>

<species id="species_36" name="TSA" compartment="mwc7a0146b_dcaf_459b_8aad_1ff6e20a3fb5" initialAmount="0.0324331443243821">

<annotation>

<COPASI xmlns="http://www.copasi.org/static/sbml">

<rdf:RDF xmlns:dcterms="http://purl.org/dc/terms/" xmlns:rdf="http://www.w3.org/1999/02/22-rdf-syntax-ns#">

<rdf:Description rdf:about="#COPASI38">

<dcterms:created>

<rdf:Description>

<dcterms:W3CDTF>2011-06-15T16:42:10Z</dcterms:W3CDTF>

</rdf:Description>

</dcterms:created>

</rdf:Description>

</rdf:RDF>

</COPASI>

</annotation>

</species>

<species id="species_37" name="OXA" compartment="mwc7a0146b_dcaf_459b_8aad_1ff6e20a3fb5" initialAmount="0.1">

<annotation>

<COPASI xmlns="http://www.copasi.org/static/sbml">

<rdf:RDF xmlns:dcterms="http://purl.org/dc/terms/" xmlns:rdf="http://www.w3.org/1999/02/22-rdf-syntax-ns#">

<rdf:Description rdf:about="#COPASI39">

<dcterms:created>

<rdf:Description>

<dcterms:W3CDTF>2011-06-28T01:15:08Z</dcterms:W3CDTF>

</rdf:Description>

</dcterms:created>

</rdf:Description>

</rdf:RDF>

</COPASI>

</annotation>

</species>

<species id="mw110e8daa_fa0e_409b_af62_9f01d755a8e3" name="ATPr" compartment="mwc7a0146b_dcaf_459b_8aad_1ff6e20a3fb5" initialAmount="0"/>

<species id="mwda7da32a_797a_4dbc_9e6a_c9317b3838b5" name="Mass" compartment="mwc7a0146b_dcaf_459b_8aad_1ff6e20a3fb5" initialAmount="0"/>

<species id="mw8369442e_7e34_45ca_89a9_7f0ab2ab70ee" name="Sink_DHAP" compartment="mwc7a0146b_dcaf_459b_8aad_1ff6e20a3fb5" initialAmount="0"/>

<species id="mw09d7964e_16d4_4c3d_a1f5_d98a7ab66891" name="Sink_E4P" compartment="mwc7a0146b_dcaf_459b_8aad_1ff6e20a3fb5" initialAmount="0"/>

<species id="mw70a9d4b8_2ca3_4692_a159_702d8464b7e7" name="Sink_Ri5P" compartment="mwc7a0146b_dcaf_459b_8aad_1ff6e20a3fb5" initialAmount="0"/>

<species id="mwa8568197_97f2_4c67_8727_f8d9ed6895e3" name="Sink_PEP" compartment="mwc7a0146b_dcaf_459b_8aad_1ff6e20a3fb5" initialAmount="0"/>

<species id="mwaf22c073_9b9c_42b1_bd8e_6e65777ddddc" name="Sink_GAP" compartment="mwc7a0146b_dcaf_459b_8aad_1ff6e20a3fb5" initialAmount="0"/>

<species id="mw7f9b1aa3_a2ab_4207_b81f_4ad133f8ccf8" name="NADPHr" compartment="mwc7a0146b_dcaf_459b_8aad_1ff6e20a3fb5" initialAmount="0"/>

<species id="mw4412eef8_75f9_408b_8ca1_0cd634ded124" name="rate_CC_1" compartment="mwc7a0146b_dcaf_459b_8aad_1ff6e20a3fb5" initialAmount="0"/>

<species id="mw5bf04cb0_8acb_4c37_a547_e939d6416759" name="rate_CC_2" compartment="mwc7a0146b_dcaf_459b_8aad_1ff6e20a3fb5" initialAmount="0"/>

<species id="mwe2947217_6be6_428f_ac4e_28ae24c11d45" name="rate_CC_3" compartment="mwc7a0146b_dcaf_459b_8aad_1ff6e20a3fb5" initialAmount="0"/>

<species id="mwac50212d_9880_4c18_b404_443dcf85e43a" name="rate_CC_4" compartment="mwc7a0146b_dcaf_459b_8aad_1ff6e20a3fb5" initialAmount="0"/>

<species id="mwdad3e574_e8ae_4a2a_bc5b_5d2a8592dffc" name="rate_CC_5" compartment="mwc7a0146b_dcaf_459b_8aad_1ff6e20a3fb5" initialAmount="0"/>

<species id="mwc04a96b8_d70f_4bdc_854b_7367baf678c4" name="rate_CC_6" compartment="mwc7a0146b_dcaf_459b_8aad_1ff6e20a3fb5" initialAmount="0"/>

<species id="mw0ec64103_a8ce_438d_97b6_aecac492a2fb" name="rate_CC_7" compartment="mwc7a0146b_dcaf_459b_8aad_1ff6e20a3fb5" initialAmount="0"/>

<species id="mwc594876f_c9b0_4f4b_a245_d024b6f1931a" name="rate_CC_8" compartment="mwc7a0146b_dcaf_459b_8aad_1ff6e20a3fb5" initialAmount="0"/>

<species id="mwb8fa5238_b6ab_43ba_914d_8d9609a94211" name="rate_CC_9" compartment="mwc7a0146b_dcaf_459b_8aad_1ff6e20a3fb5" initialAmount="0"/>

<species id="mwd13e1918_33bb_4d69_9527_a7db921570eb" name="rate_CC_10" compartment="mwc7a0146b_dcaf_459b_8aad_1ff6e20a3fb5" initialAmount="0"/>

<species id="mwbe2ee4f3_36d2_4a42_8390_f8082ac79296" name="rate_CC_11" compartment="mwc7a0146b_dcaf_459b_8aad_1ff6e20a3fb5" initialAmount="0"/>

<species id="mw60c6f424_4d57_49bf_b130_811bec897e0a" name="rate_CC_12" compartment="mwc7a0146b_dcaf_459b_8aad_1ff6e20a3fb5" initialAmount="0"/>

<species id="mw96deb747_ef33_4a50_b36e_145767b53cf7" name="rate_CC_13" compartment="mwc7a0146b_dcaf_459b_8aad_1ff6e20a3fb5" initialAmount="0"/>

<species id="mw42c5ea06_edf8_46bd_b262_f9db84921975" name="rate_PFK" compartment="mwc7a0146b_dcaf_459b_8aad_1ff6e20a3fb5" initialAmount="0"/>

<species id="mw10231dd9_d13a_4822_a477_0b190284526c" name="rate_PGM" compartment="mwc7a0146b_dcaf_459b_8aad_1ff6e20a3fb5" initialAmount="0"/>

<species id="mw0e384600_a904_40dc_b3a6_de20a7ae04b8" name="rate_ENO" compartment="mwc7a0146b_dcaf_459b_8aad_1ff6e20a3fb5" initialAmount="0"/>

<species id="mwaecebc73_e8e8_4aa8_a5c9_9d2295bf77d4" name="rate_glyc_GAP" compartment="mwc7a0146b_dcaf_459b_8aad_1ff6e20a3fb5" initialAmount="0"/>

<species id="mwd008246b_4ac0_4550_9588_64a1e8664cd1" name="rate_PEPsink" compartment="mwc7a0146b_dcaf_459b_8aad_1ff6e20a3fb5" initialAmount="0"/>

<species id="mw8dc354b7_d1b2_4cc9_bbf9_5d423d6013fb" name="Sink_G6P" compartment="mwc7a0146b_dcaf_459b_8aad_1ff6e20a3fb5" initialAmount="0"/>

<species id="mwf7b66b1f_77b0_4024_99e5_fb37fb0ab929" name="rate_G6Psink" compartment="mwc7a0146b_dcaf_459b_8aad_1ff6e20a3fb5" initialAmount="0"/>

<species id="mw6eb33847_58dd_4306_8ae9_7d68bb2a5612" name="rate_GAPsink" compartment="mwc7a0146b_dcaf_459b_8aad_1ff6e20a3fb5" initialAmount="0"/>

<species id="mw4a42b5e7_485f_4d6b_a4ed_e490759e5f26" name="rate_E4Psink" compartment="mwc7a0146b_dcaf_459b_8aad_1ff6e20a3fb5" initialAmount="0"/>

```xml
<species id="mw3128a56f_9005_484d_92b7_1b5e45b577c6" name="rate_Ri5Psink" compartment="mwc7a0146b_dcaf_459b_8aad_1ff6e20a3fb5" initialAmount="0"/>

<species id="mwfe019cb4_4031_44c9_83f8_9ba5e8de4ed4" name="AceP" compartment="mwc7a0146b_dcaf_459b_8aad_1ff6e20a3fb5" initialAmount="0"/>

<species id="mwca272a09_38a7_4416_a781_d64c8e945cc4" name="rate_PKET" compartment="mwc7a0146b_dcaf_459b_8aad_1ff6e20a3fb5" initialAmount="0"/>

<species id="mwe556cd16_34e2_417a_b945_37061560bfe5" name="Sink_AceP" compartment="mwc7a0146b_dcaf_459b_8aad_1ff6e20a3fb5" initialAmount="0"/>

<species id="mw3447e887_c5e2_4271_9e5f_50c309bc8dc3" name="P6G" compartment="mwc7a0146b_dcaf_459b_8aad_1ff6e20a3fb5" initialAmount="0"/>

<species id="mwf41678a2_15b0_484e_b056_0c8a195fed4d" name="OPP_rate" compartment="mwc7a0146b_dcaf_459b_8aad_1ff6e20a3fb5" initialAmount="0"/>

<species id="mw733b668e_1dba_46ef_9777_e9331b0b590d" name="CO2" compartment="mw1341fc13_2fe2_46f4_b24f_cd32c24b8dca" initialAmount="0.1" boundaryCondition="true" constant="true"/>

</listOfSpecies>

<listOfParameters>

<parameter id="parameter_1" name="kf_ATP" value="0.16">

<annotation>

<COPASI xmlns="http://www.copasi.org/static/sbml">

<rdf:RDF xmlns:dcterms="http://purl.org/dc/terms/" xmlns:rdf="http://www.w3.org/1999/02/22-rdf-syntax-ns#">

<rdf:Description rdf:about="#COPASI47">

<dcterms:created>

<rdf:Description>

<dcterms:W3CDTF>2010-04-14T15:40:36Z</dcterms:W3CDTF>

</rdf:Description>

</dcterms:created>

</rdf:Description>

</rdf:RDF>

</COPASI>
```

```xml
</annotation>

</parameter>

<parameter id="parameter_2" name="kf_NADPH" value="0.58">

<annotation>

<COPASI xmlns="http://www.copasi.org/static/sbml">

<rdf:RDF xmlns:dcterms="http://purl.org/dc/terms/" xmlns:rdf="http://www.w3.org/1999/02/22-rdf-syntax-ns#">

<rdf:Description rdf:about="#COPASI48">

<dcterms:created>

<rdf:Description>

<dcterms:W3CDTF>2010-07-25T02:11:54Z</dcterms:W3CDTF>

</rdf:Description>

</dcterms:created>

</rdf:Description>

</rdf:RDF>

</COPASI>

</annotation>

</parameter>

<parameter id="mw9f919ddc_7006_491f_b827_5b5094d66dd0" name="V_PP1" value="0.009"/>

<parameter id="mwf008a49f_a4ec_49ec_8cdc_30906d544233" name="V1" value="0.991"/>

<parameter id="mwdbdd129f_0428_4a42_8427_cf18a0b5568c" name="V5" value="1.613"/>

<parameter id="mwfe139de1_b141_420e_a4e5_f59c639c6cf0" name="V2" value="0.6"/>

<parameter id="mwc727e6a6_e76e_44d0_89f4_bede015cb04c" name="V3" value="0.536"/>

<parameter id="mw8c8ad41e_6d63_473b_960b_322d5feb6f84" name="V4" value="0.37"/>

<parameter id="mw9dda631f_8096_4e56_a670_fdb559f36d12" name="V6" value="0.06"/>

<parameter id="mwea0f726e_b6ff_4cf0_8dba_9db4cc0827be" name="V7" value="0.11"/>

<parameter id="mw33ab4422_ba35_4d2b_9620_d274168c7e1d" name="V8" value="1.11"/>

<parameter id="mw5c6f658c_7714_4fbb_be83_5ef825b3e08b" name="V9" value="1.2"/>
```

```xml
<parameter id="mw6e8005dd_31e8_483e_902a_8870307bf558" name="V10" value="0.13"/>
<parameter id="mw4fbb318e_c400_4af5_8e3f_e80aec639ee3" name="V11" value="1.1"/>
<parameter id="mw702a7f39_5675_48ad_953a_4bcbbdc8e1a2" name="V12" value="1.28"/>
<parameter id="mwd486ae05_3de1_42aa_9584_2edc458d0c59" name="V13" value="0.43"/>
<parameter id="mw243dc3c0_44b5_4ef5_b8ba_92846b4fee4c" name="V_SS1" value="0.99"/>
<parameter id="mwaeeded85_9203_48b6_89a9_9dbf6c0d38da" name="KE_SS1" value="0.8"/>
<parameter id="mw715a0d4a_6423_4755_a1b5_7d9e5b1852a2" name="V_Sink_E4P" value="0.0009"/>
<parameter id="mw6caa1525_e69b_4ab2_b8c7_2b7deeb5ee58" name="V_Sink_Ri5P" value="0.006"/>
<parameter id="mw3c5b8b60_a85d_4b23_8f30_04a8715af44c" name="V_PP2a" value="0.0007"/>
<parameter id="mw5f743344_e84b_4721_8bd4_3643964b38ea" name="V_PP3" value="0.0042"/>
<parameter id="mw43b7b905_45eb_4193_9d33_c0623544faba" name="V_PP4" value="0.43"/>
<parameter id="mw21878232_3329_413d_9ad8_7dd1cfc2be9c" name="V_PP5" value="0.002"/>
<parameter id="mw9331ee05_e8ae_4da7_be3c_2ae749a0c97a" name="V_PP6" value="0.004"/>
<parameter id="mw4a170042_d461_4f96_b16d_2969db08423c" name="V_PP7" value="0.013"/>
<parameter id="mw5723da7e_7ee9_4b45_b397_c4fad0251fa2" name="V_Sink_PEP" value="0.021"/>
<parameter id="mwa5897908_6cba_4e9b_ae16_669f6cc2215d" name="V_synth_SER" value="0.0008"/>
<parameter id="mwf7e8c4cc_5866_4d9d_80a4_fb1d0db24ecf" name="V_TSA1" value="0.01"/>
<parameter id="mw0f93be3f_8f7b_48cf_b994_7d7d51c0c99d" name="V_TSA2" value="0.023"/>
<parameter id="mw4d57904b_347d_4323_a435_aef6db506bb9" name="V_TSA3" value="0.1"/>
<parameter id="mw7f7c8a85_afe2_49aa_8686_1b3950810d23" name="V_OXA1" value="0.12"/>
<parameter id="mwa562da41_e9ca_4d43_85ba_4372aa40bed8" name="V_OXA2" value="0.0002"/>
<parameter id="mwdde5b27b_4aa1_42aa_b292_6613ea7584a3" name="KE4" value="0.1"/>
<parameter id="mw4ec82ea3_67a6_47ad_971a_52a33f180466" name="KE7" value="0.3"/>
```

```
<parameter id="mwc59d3254_509c_411a_b656_9e80ac0a4d4a" name="KM_PFK" value="0.5"/>

<parameter id="mwfb9d0842_5ad1_42a2_b44c_12cfeaa36962" name="V_PFK" value="0.02"/>

<parameter id="mw701ca18f_a968_4128_8415_d39acae3e377" name="Km112b" value="0.1"/>

<parameter id="mwa378ec6c_6ace_46c3_a515_4be6d27ac144" name="V_PP2b" value="0.0005"/>

<parameter id="mw04118d88_412e_49b2_8ed5_aba0f23d1258" name="Km112a" value="3"/>

<parameter id="mwe806711a_3cbc_4d66_a530_d7ae4e673eea" name="Kms_enol" value="0.525"/>

<parameter id="mwdee0f9ac_28b4_444f_af80_30c299f78093" name="Kmp_enol" value="0.279"/>

<parameter id="mw70b2b9c1_0904_4851_aee4_8526d26a4c7f" name="K_Sink_PEP" value="0.674"/>

<parameter id="mw9c6e9e64_1ad3_462a_b257_61d666a10a15" name="V_Sink_GLY" value="0.0002"/>

<parameter id="mw108b366f_58cf_4f32_9f78_d3a14dd13026" name="V_Sink_SER" value="0.00194"/>

<parameter id="mw245d5cf2_30db_40c3_b1a7_ed389fff8808" name="KE5" value="1.27"/>

<parameter id="mw917c32b0_7a3f_4744_ac14_2e3d15da96f6" name="Keq_PGM" value="0.71"/>

<parameter id="mw231dd6ca_4db0_4cdb_adc8_72349abdba07" name="Kmp_PGM_alpha" value="0.74"/>

<parameter id="mw742fbec5_509e_42be_b7a8_12b9eeb49314" name="Kms_PGM_alpha" value="0.189"/>

<parameter id="mw4f571ae6_7040_40c6_b098_4ac7cf9ee24d" name="Vf_PGM_alpha" value="0.24"/>

<parameter id="mw23fae7dc_7d12_4fb0_98e2_c611e44007e1" name="Kmp_PGM_beta" value="0.005"/>

<parameter id="mwb99ebe97_7d91_406c_8b12_a56b2c939ff4" name="Kms_PGM_beta" value="0.277"/>

<parameter id="mw2ec1703a_729a_4b49_b4dd_4241b0d73907" name="Vf_PGM_beta" value="0.71"/>

<parameter id="mw23f5979c_8188_4521_9629_6469c95e42d6" name="Kmp_PGM_gama" value="0.6"/>
```

```
<parameter id="mw6205c3fd_ecf9_4b2b_98f1_dff68729cd03" name="Kms_PGM_gama" value="2.58"/>
<parameter id="mwa1879047_3c26_45c1_8b2a_137433500cde" name="Vf_PGM_gama" value="0.0278"/>
<parameter id="mwdacf4f8b_f1db_480a_aabf_ff89dc90e087" name="Keq_enol" value="0.866"/>
<parameter id="mw68a26bf2_520b_4b35_90e0_511ec4bbc859" name="Vf_enol" value="0.823"/>
<parameter id="mwaffea60c_8361_4bed_be10_814e0c0f7898" name="kf_CO2" value="1.91"/>
<parameter id="mw39e8b6c4_2ae7_4846_9ac8_e77ee9c7191f" name="KM51" value="0.3"/>
<parameter id="mw54c31bc3_16fc_462a_a55e_a766a97769ed" name="KM52" value="0.4"/>
<parameter id="mwbad28d74_abec_451a_a6bf_a03335045383" name="KM53" value="0.02"/>
<parameter id="mwe110663f_33e9_415c_96f8_04e92e007bb4" name="V_Sink_GAP" value="0.00018"/>
<parameter id="mw395deb93_b80e_4154_b71a_add84953daaf" name="K_Sink_GAP" value="0.1"/>
<parameter id="mw2a519375_b634_461c_a4e7_2205e166b3ce" name="Kgap" value="1"/>
<parameter id="mw02659a3e_942b_4113_928b_885d34341771" name="Knadpp" value="2"/>
<parameter id="mw25dcc228_99f4_4d62_badc_00067a7db23a" name="Kpga" value="0.357"/>
<parameter id="mwd0f35f82_c657_4a53_9ac4_606e79cebb96" name="Vgap_dehyd" value="1.24"/>
<parameter id="mw31fc107e_644a_475e_aeff_2b016202c816" name="knadph" value="0.927"/>
<parameter id="mw1a31f571_e225_4d2d_b672_bb6209080e70" name="Keq_gap_dehyd" value="0.471"/>
<parameter id="mw04a2d768_b6a0_4a14_8c35_7cee82a7a308" name="Kgap_beta" value="1"/>
<parameter id="mwa177ed29_1ff2_4ddf_aa7c_8cc4c08a1a2d" name="Knadpp_beta" value="1"/>
<parameter id="mw18e6c815_3a2d_4abe_8f8c_96b098a1ffe4" name="Kpga_beta" value="1"/>
<parameter id="mw03434a4a_4ed9_491d_bb10_f39e62b3acb4" name="Vgap_dehyd_beta" value="0"/>
<parameter id="mw0d8a79b9_2494_4bbb_8324_70f0b2e27188" name="knadph_beta" value="1"/>
```

```xml
<parameter id="mwe6b619dd_229f_4afa_af14_be3a25b0e262" name="KM61" value="0.033"/>
<parameter id="mwd59eae5a_ff1f_4351_922c_d25f34ca05ac" name="KM72" value="0.1"/>
<parameter id="mwd037be1a_7ea7_4d03_b00e_8f358afc43f0" name="KM73" value="0.1"/>
<parameter id="mw6990314a_e646_4a51_b298_7b53a5a3ca3b" name="KM71" value="0.1"/>
<parameter id="mwd32ff45c_71b0_4916_8ffc_7683019b9311" name="KM74" value="0.1"/>
<parameter id="mwa6f625ba_c9e6_40e4_bf00_d59146ed8f80" name="KM82" value="0.2"/>
<parameter id="mw28855de3_91da_4e09_b2c9_c0eb4b719af3" name="KM81" value="0.4"/>
<parameter id="mwf44df5f6_9e91_4081_a77d_911fe8867849" name="KE8" value="0.83"/>
<parameter id="mw2527d5af_e782_46fb_9b2c_875866d6ecd5" name="KM9" value="0.05"/>
<parameter id="mw075f4d45_ceca_4aad_bc29_89b6915e46f7" name="KE10" value="1.54"/>
<parameter id="mwf8558c32_d374_4352_b837_54ce36f88a5e" name="KM71a" value="0.616"/>
<parameter id="mwf187cf00_32ca_422a_9134_ffd278f81118" name="KM101" value="0.118"/>
<parameter id="mw94fab6bb_d1d8_4344_ac40_d9ea47173826" name="KE11" value="2.35"/>
<parameter id="mw46a0d4e0_a24e_4d84_ac9d_1d098ed93c0b" name="KE12" value="0.32"/>
<parameter id="mwc1d6540f_78d6_40ae_8e94_d6db3d674a6e" name="KM132" value="0.05"/>
<parameter id="mw1862dfa3_5857_4ef7_82ea_209a042715a4" name="K_GOA" value="0.1"/>
<parameter id="mw016a3190_5878_41d4_8022_96365a5cde42" name="V_GLY_syn" value="0.1"/>
<parameter id="mwdec52954_3ec3_416b_8d1c_d8191ee0052a" name="KM11" value="0.0115"/>
<parameter id="mw67c2b3cc_db05_4a74_97af_8d7314b6868b" name="KM12" value="0.222"/>
<parameter id="mwa4c7bb69_e0a6_4881_b8c4_8bb392a17249" name="KI11" value="0.84"/>
<parameter id="mwa496f448_c225_4611_af1e_f7f3dfa7977c" name="KI14" value="0.9"/>
<parameter id="mw49a330a3_2804_4b34_bed5_55979f9f820c" name="KM13" value="0.02"/>
<parameter id="mwaab87003_70c0_4690_a563_726e460f9c64" name="KI12" value="0.04"/>
<parameter id="mw8642dd0a_5a0f_45a5_b0ca_d246e508136b" name="KI13" value="0.075"/>
<parameter id="mw7aeaf567_e744_423f_b2c4_3aeb34386eb6" name="KI15" value="0.07"/>
<parameter id="mw33c77066_cf3c_44c1_9e75_e85e2c99a3fd" name="KM21" value="0.24"/>
```

```xml
<parameter id="mwfd3ba144_51dd_4c75_b0e1_8926ab5477e8" name="KM22" value="0.39"/>
<parameter id="mwb70b9188_7453_4609_ad48_be4bf69f87e4" name="KM23" value="0.23"/>
<parameter id="mwbd9ba0b8_46b5_43a7_894a_1e0a7450c854" name="KE2" value="0.000619"/>
<parameter id="mw701b6fb7_2279_4661_a2d5_e1e2e9f47203" name="KM31" value="0.004"/>
<parameter id="mw9ea5d254_32bc_4a37_aa1f_f4b94da8eadb" name="KM32" value="0.1"/>
<parameter id="mw9f468e68_fa4c_4260_a5af_3b6182abcb92" name="KI62" value="12"/>
<parameter id="mwa90c1c5f_dcc6_48a5_a99f_ce08e7c2c91b" name="KI61" value="0.7"/>
<parameter id="mw118d7663_7b18_410d_8ca7_fc168407ba10" name="KI9" value="12"/>
<parameter id="mwe195dc18_fc3c_4329_905f_5bb2fc3efe19" name="KM104" value="0.54"/>
<parameter id="mwb61a7f15_d0aa_4449_85bb_2cdf4a397051" name="KM103" value="0.09"/>
<parameter id="mwe1915e65_ff53_471a_9d2c_b92b8d75f287" name="KM102" value="0.09"/>
<parameter id="mw7d11f964_6e54_4584_8c91_1808e4e850fd" name="KM72b" value="0.27"/>
<parameter id="mw814665c4_d157_4663_a75e_af6f8f891fb0" name="KI135" value="0.4"/>
<parameter id="mwd947eee9_9a97_4305_a38f_5d0342490834" name="KI131" value="2"/>
<parameter id="mw16745cb2_2a9e_48f6_8cef_80e44ec67119" name="KI134" value="2.5"/>
<parameter id="mw6bb6ce48_f2df_4db7_abe8_95a611a6672b" name="KI133" value="4"/>
<parameter id="mw0b12ed4b_ffff_47bd_a925_140813e42732" name="KI132" value="0.7"/>
<parameter id="mw9c902b95_94d4_4dff_912f_eb93bd74d440" name="KM131" value="0.05"/>
<parameter id="mw52505a99_c3c7_4c4c_8a62_b891bfab4dd8" name="K_Sink_G6P" value="2"/>
<parameter id="mw2fcbcb1c_c103_48bb_86ed_9229a27de6ed" name="V_Sink_G6P" value="0.03"/>
<parameter id="mwa27b7723_ab20_4324_a886_f5c73588ac4a" name="KM_PKET" value="2"/>
<parameter id="mw72f442a2_e01a_44d3_b8bf_9d223245f350" name="V_PKET" value="0.015"/>
<parameter id="mwc1298b32_b9e2_4ab0_8087_3acdea483cdc" name="K_Sink_AceP" value="1"/>
<parameter id="mwd7c8b802_9d0c_4a81_9c65_1a50be7968bb" name="V_Sink_AceP" value="1"/>
```

```xml
<parameter id="mw6f022b45_ad82_4bfc_bbbd_0855ddf10ecf" name="K_OXA1" value="2"/>
<parameter id="mwcd39d210_482b_47b5_b954_07cc17e31155" name="K_OXA2" value="5"/>
<parameter id="mw57949b29_e69c_444c_8d58_1ddda96d13c5" name="KM_PKET_beta" value="0.2"/>
<parameter id="mw86416738_ea27_41b8_adf9_4d3b5a320d97" name="V_PKET_beta" value="0.0005"/>
<parameter id="mw9d451500_8215_4269_83be_e237d42f0aab" name="KM_PFK_beta" value="2.5"/>
<parameter id="mw61b0fed3_e790_4af3_a1e7_65c1646248bf" name="V_PFK_beta" value="0.07"/>
<parameter id="mwd2bb54b2_f070_41d0_a500_9b196833f57a" name="KI61_beta" value="6"/>
<parameter id="mwdb1e1973_5df2_4a29_a277_64c677c902b4" name="KM61_beta" value="0.3"/>
<parameter id="mw13a07b09_9a67_4561_b3db_8e39442e5d53" name="V6_beta" value="0.04"/>
<parameter id="mw9168b04c_6d3a_4f60_8906_5b5293d6a01e" name="Kmp_V11_alpha" value="2"/>
<parameter id="mwf2185be5_b4b0_4bf4_a6d3_0a758e4fbcca" name="Kms_V11_alpha" value="0.1"/>
<parameter id="mw332683df_4959_4366_94ef_2b7108d780dc" name="Kmp_V11_beta" value="0.05"/>
<parameter id="mw8da43b9c_22d4_4c48_bcc7_0bb2a83b77f6" name="Kms_V11_beta" value="2"/>
<parameter id="mw15a7ce92_eb88_4188_966c_aad7e9d8ea7e" name="V11_beta" value="0.4"/>
<parameter id="mw80012d61_bf17_467d_8029_8785e8b66833" name="Kmp_PPP1" value="0.1"/>
<parameter id="mw212994b8_ca26_4fbc_a13e_bb054a312812" name="Kms_PPP1" value="2"/>
<parameter id="mw84e3d5fb_d3f5_4a42_a963_2d0e4de0c9fa" name="V_PPP1" value="0.54"/>
<parameter id="mwbf2d484b_0c51_4597_84e7_a9d72a8ed607" name="Kms_nadpp_ppp2" value="1"/>
<parameter id="mw9aad6b3a_ddc4_4aa7_af1d_9c6f98a72a80" name="Kms_p6g_ppp2" value="1.2"/>
```

```
<parameter id="mwacee8ded_69a8_4141_87f2_2622f06daa29" name="V_PPP2" value="0.3"/>
<parameter id="mw4d3fe418_9692_4308_a1c1_64b6e3b6ad82" name="K_Sink_E4P"
value="0.1"/>
<parameter id="mwe871df2e_a7b7_49c5_8a95_809524380cbd" name="K_Sink_Ri5P"
value="0.1"/>
<parameter id="mw42e7bfb9_db8f_4b73_bd5b_4f46a35e36a0" name="KI1121" value="94"/>
<parameter id="mw43a4fc8c_e159_44c4_aca5_0de4d39f2892" name="KI1122" value="2.55"/>
<parameter id="mw2026f64b_36e7_4da9_8f99_4179a8aa5a0f" name="Km112c" value="0.5"/>
<parameter id="mwe7492e74_1fd5_412c_9bd7_c33659cbb9ae" name="V_PP2c"
value="0.001"/>
</listOfParameters>
<listOfRules>
<assignmentRule metaid="repeatedAssignment_mw48d91844_76d7_4f00_b1f9_dbedf937d63f"
variable="mw110e8daa_fa0e_409b_af62_9f01d755a8e3">
  <math xmlns="http://www.w3.org/1998/Math/MathML">
    <apply>
      <divide/>
      <ci> species_7 </ci>
      <apply>
        <plus/>
        <ci> species_7 </ci>
        <ci> species_4 </ci>
      </apply>
    </apply>
  </math>
</assignmentRule>
<assignmentRule metaid="repeatedAssignment_mw1babcdad_d925_420b_81f6_ebb44bb8be53"
variable="mwda7da32a_797a_4dbc_9e6a_c9317b3838b5">
  <math xmlns="http://www.w3.org/1998/Math/MathML">
```

```
        <apply>
          <plus/>
          <ci> mwa8568197_97f2_4c67_8727_f8d9ed6895e3 </ci>
          <ci> mw8369442e_7e34_45ca_89a9_7f0ab2ab70ee </ci>
          <ci> mw09d7964e_16d4_4c3d_a1f5_d98a7ab66891 </ci>
          <ci> mw70a9d4b8_2ca3_4692_a159_702d8464b7e7 </ci>
          <ci> mwe556cd16_34e2_417a_b945_37061560bfe5 </ci>
          <ci> species_23 </ci>
          <ci> species_24 </ci>
          <ci> species_33 </ci>
          <ci> species_34 </ci>
        </apply>
      </math>
    </assignmentRule>
    <assignmentRule metaid="repeatedAssignment_mw7ce83324_309f_45ce_b8ae_701c6f34d663"
variable="mw7f9b1aa3_a2ab_4207_b81f_4ad133f8ccf8">
      <math xmlns="http://www.w3.org/1998/Math/MathML">
        <apply>
          <divide/>
          <ci> species_5 </ci>
          <apply>
            <plus/>
            <ci> species_5 </ci>
            <ci> species_11 </ci>
          </apply>
        </apply>
      </math>
    </assignmentRule>
```

<assignmentRule metaid="repeatedAssignment_mwca4fd66d_9ddc_4230_9737_ae88453e6d30" variable="mw4412eef8_75f9_408b_8ca1_0cd634ded124">

<math xmlns="http://www.w3.org/1998/Math/MathML">

<apply>

<divide/>

<apply>

<times/>

<ci> species_3 </ci>

<apply>

<divide/>

<apply>

<times/>

<ci> mwf008a49f_a4ec_49ec_8cdc_30906d544233 </ci>

<ci> species_1 </ci>

</apply>

<apply>

<plus/>

<ci> species_1 </ci>

<apply>

<times/>

<ci> mwdec52954_3ec3_416b_8d1c_d8191ee0052a </ci>

<apply>

<plus/>

<cn type="integer"> 1 </cn>

<apply>

<divide/>

<ci> species_6 </ci>

<ci> mw67c2b3cc_db05_4a74_97af_8d7314b6868b </ci>

```
          </apply>
        </apply>
      </apply>
    </apply>
  </apply>
</apply>
<apply>
  <plus/>
  <ci> species_3 </ci>
  <apply>
    <times/>
    <ci> mw49a330a3_2804_4b34_bed5_55979f9f820c </ci>
    <apply>
      <plus/>
      <cn type="integer"> 1 </cn>
      <apply>
        <divide/>
        <ci> species_2 </ci>
        <ci> mwa4c7bb69_e0a6_4881_b8c4_8bb392a17249 </ci>
      </apply>
      <apply>
        <divide/>
        <ci> species_14 </ci>
        <ci> mwaab87003_70c0_4690_a563_726e460f9c64 </ci>
      </apply>
      <apply>
        <divide/>
```

<ci> species_8 </ci>

<ci> mw8642dd0a_5a0f_45a5_b0ca_d246e508136b </ci>

</apply>

<apply>

<divide/>

<ci> species_12 </ci>

<ci> mwa496f448_c225_4611_af1e_f7f3dfa7977c </ci>

</apply>

<apply>

<divide/>

<ci> species_5 </ci>

<ci> mw7aeaf567_e744_423f_b2c4_3aeb34386eb6 </ci>

</apply>

</apply>

</apply>

</apply>

</apply>

</math>

</assignmentRule>

<assignmentRule metaid="repeatedAssignment_mw46513e34_7bce_44fb_bf65_e95c442f2c9c" variable="mw5bf04cb0_8acb_4c37_a547_e939d6416759">

<math xmlns="http://www.w3.org/1998/Math/MathML">

<apply>

<divide/>

<apply>

<times/>

<ci> mwfe139de1_b141_420e_a4e5_f59c639c6cf0 </ci>

<apply>

```
      <minus/>
      <apply>
        <times/>
        <ci> species_2 </ci>
        <ci> species_7 </ci>
      </apply>
      <apply>
        <divide/>
        <apply>
          <times/>
          <ci> species_9 </ci>
          <ci> species_4 </ci>
        </apply>
        <ci> mwbd9ba0b8_46b5_43a7_894a_1e0a7450c854 </ci>
      </apply>
    </apply>
  </apply>
  <apply>
    <times/>
    <apply>
      <plus/>
      <ci> species_2 </ci>
      <ci> mw33c77066_cf3c_44c1_9e75_e85e2c99a3fd </ci>
    </apply>
    <apply>
      <plus/>
      <ci> species_7 </ci>
```

```
              <apply>
                <times/>
                <ci> mwfd3ba144_51dd_4c75_b0e1_8926ab5477e8 </ci>
                <apply>
                  <plus/>
                  <cn type="integer"> 1 </cn>
                  <apply>
                    <divide/>
                    <ci> species_4 </ci>
                    <ci> mwb70b9188_7453_4609_ad48_be4bf69f87e4 </ci>
                  </apply>
                </apply>
              </apply>
            </apply>
          </apply>
        </apply>
      </math>
    </assignmentRule>
    <assignmentRule metaid="repeatedAssignment_mw0d2bcf4d_a89c_4584_b28a_3af29bc061c0"
variable="mwe2947217_6be6_428f_ac4e_28ae24c11d45">
      <math xmlns="http://www.w3.org/1998/Math/MathML">
        <apply>
          <divide/>
          <apply>
            <times/>
            <ci> mwc727e6a6_e76e_44d0_89f4_bede015cb04c </ci>
            <ci> species_9 </ci>
            <ci> species_5 </ci>
```

```
          </apply>
          <apply>
            <times/>
            <apply>
              <plus/>
              <ci> species_9 </ci>
              <ci> mw701b6fb7_2279_4661_a2d5_e1e2e9f47203 </ci>
            </apply>
            <apply>
              <plus/>
              <ci> species_5 </ci>
              <ci> mw9ea5d254_32bc_4a37_aa1f_f4b94da8eadb </ci>
            </apply>
          </apply>
        </apply>
      </math>
    </assignmentRule>
    <assignmentRule metaid="repeatedAssignment_mw91c1bad8_ddb2_4b54_9440_497633fe887c"
variable="mwac50212d_9880_4c18_b404_443dcf85e43a">
      <math xmlns="http://www.w3.org/1998/Math/MathML">
        <apply>
          <divide/>
          <apply>
            <times/>
            <ci> mw8c8ad41e_6d63_473b_960b_322d5feb6f84 </ci>
            <apply>
              <minus/>
              <ci> species_10 </ci>
```

```
          <apply>
            <divide/>
            <ci> species_13 </ci>
            <ci> mwdde5b27b_4aa1_42aa_b292_6613ea7584a3 </ci>
          </apply>
        </apply>
      </apply>
      <apply>
        <plus/>
        <ci> species_10 </ci>
        <ci> species_13 </ci>
      </apply>
    </apply>
  </math>
</assignmentRule>
<assignmentRule metaid="repeatedAssignment_mwf718ba1f_2460_4130_a6f4_199dbcd27a04"
variable="mwdad3e574_e8ae_4a2a_bc5b_5d2a8592dffc">
  <math xmlns="http://www.w3.org/1998/Math/MathML">
    <apply>
      <divide/>
      <apply>
        <times/>
        <ci> mwdbdd129f_0428_4a42_8427_cf18a0b5568c </ci>
        <apply>
          <minus/>
          <apply>
            <times/>
            <ci> species_10 </ci>
```

```
      <ci> species_13 </ci>
    </apply>
    <apply>
      <divide/>
      <ci> species_14 </ci>
      <ci> mw245d5cf2_30db_40c3_b1a7_ed389fff8808 </ci>
    </apply>
  </apply>
</apply>
<apply>
  <times/>
  <ci> mw39e8b6c4_2ae7_4846_9ac8_e77ee9c7191f </ci>
  <ci> mw54c31bc3_16fc_462a_a55e_a766a97769ed </ci>
  <apply>
    <plus/>
    <cn type="integer"> 1 </cn>
    <apply>
      <divide/>
      <ci> species_10 </ci>
      <ci> mw39e8b6c4_2ae7_4846_9ac8_e77ee9c7191f </ci>
    </apply>
    <apply>
      <divide/>
      <ci> species_13 </ci>
      <ci> mw54c31bc3_16fc_462a_a55e_a766a97769ed </ci>
    </apply>
    <apply>
```

```
            <divide/>
            <ci> species_14 </ci>
            <ci> mwbad28d74_abec_451a_a6bf_a03335045383 </ci>
           </apply>
           <apply>
            <divide/>
            <apply>
             <times/>
             <ci> species_10 </ci>
             <ci> species_13 </ci>
            </apply>
            <apply>
             <times/>
             <ci> mw39e8b6c4_2ae7_4846_9ac8_e77ee9c7191f </ci>
             <ci> mw54c31bc3_16fc_462a_a55e_a766a97769ed </ci>
            </apply>
           </apply>
          </apply>
         </apply>
        </apply>
       </math>
      </assignmentRule>
      <assignmentRule metaid="repeatedAssignment_mw78bd7f23_1644_4b90_b0fb_6a4c3079d5c3"
variable="mwc04a96b8_d70f_4bdc_854b_7367baf678c4">
       <math xmlns="http://www.w3.org/1998/Math/MathML">
        <apply>
         <plus/>
         <apply>
```

```
<divide/>
<apply>
  <times/>
  <ci> mw9dda631f_8096_4e56_a670_fdb559f36d12 </ci>
  <ci> species_14 </ci>
</apply>
<apply>
  <plus/>
  <ci> species_14 </ci>
  <apply>
    <times/>
    <ci> mwe6b619dd_229f_4afa_af14_be3a25b0e262 </ci>
    <apply>
      <plus/>
      <cn type="integer"> 1 </cn>
      <apply>
        <divide/>
        <ci> species_15 </ci>
        <ci> mwa90c1c5f_dcc6_48a5_a99f_ce08e7c2c91b </ci>
      </apply>
      <apply>
        <divide/>
        <ci> species_12 </ci>
        <ci> mw9f468e68_fa4c_4260_a5af_3b6182abcb92 </ci>
      </apply>
    </apply>
  </apply>
```

```
  </apply>
</apply>
<apply>
  <divide/>
  <apply>
    <times/>
    <ci> mw13a07b09_9a67_4561_b3db_8e39442e5d53 </ci>
    <ci> species_14 </ci>
  </apply>
  <apply>
    <plus/>
    <ci> species_14 </ci>
    <apply>
      <times/>
      <ci> mwdb1e1973_5df2_4a29_a277_64c677c902b4 </ci>
      <apply>
        <plus/>
        <cn type="integer"> 1 </cn>
        <apply>
          <divide/>
          <ci> species_15 </ci>
          <ci> mwd2bb54b2_f070_41d0_a500_9b196833f57a </ci>
        </apply>
        <apply>
          <divide/>
          <ci> species_12 </ci>
          <ci> mw9f468e68_fa4c_4260_a5af_3b6182abcb92 </ci>
```

```
              </apply>
            </apply>
          </apply>
        </apply>
      </apply>
    </apply>
  </math>
</assignmentRule>
<assignmentRule metaid="repeatedAssignment_mwd0e165e8_210e_4a23_9120_c2a66a94a9bd"
variable="mw0ec64103_a8ce_438d_97b6_aecac492a2fb">
  <math xmlns="http://www.w3.org/1998/Math/MathML">
    <apply>
      <divide/>
      <apply>
        <times/>
        <ci> mwea0f726e_b6ff_4cf0_8dba_9db4cc0827be </ci>
        <apply>
          <minus/>
          <apply>
            <times/>
            <ci> species_15 </ci>
            <ci> species_10 </ci>
          </apply>
          <apply>
            <divide/>
            <apply>
              <times/>
              <ci> species_17 </ci>
```

<ci> species_16 </ci>

</apply>

<ci> mw4ec82ea3_67a6_47ad_971a_52a33f180466 </ci>

</apply>

</apply>

</apply>

<apply>

<times/>

<apply>

<plus/>

<ci> species_15 </ci>

<apply>

<times/>

<ci> mwd037be1a_7ea7_4d03_b00e_8f358afc43f0 </ci>

<apply>

<plus/>

<cn type="integer"> 1 </cn>

<apply>

<divide/>

<ci> species_17 </ci>

<ci> mw6990314a_e646_4a51_b298_7b53a5a3ca3b </ci>

</apply>

<apply>

<divide/>

<ci> species_16 </ci>

<ci> mwd59eae5a_ff1f_4351_922c_d25f34ca05ac </ci>

</apply>

</apply>

</apply>

</apply>

<apply>

<plus/>

<ci> species_10 </ci>

<ci> mwd32ff45c_71b0_4916_8ffc_7683019b9311 </ci>

</apply>

</apply>

</apply>

</math>

</assignmentRule>

<assignmentRule metaid="repeatedAssignment_mwbbdf0263_4ad0_4ab1_8bbd_234f5f14a2a9" variable="mwc594876f_c9b0_4f4b_a245_d024b6f1931a">

<math xmlns="http://www.w3.org/1998/Math/MathML">

<apply>

<divide/>

<apply>

<times/>

<ci> mw33ab4422_ba35_4d2b_9620_d274168c7e1d </ci>

<apply>

<minus/>

<apply>

<times/>

<ci> species_13 </ci>

<ci> species_16 </ci>

</apply>

<apply>

```
            <divide/>
            <ci> species_8 </ci>
            <ci> mwf44df5f6_9e91_4081_a77d_911fe8867849 </ci>
          </apply>
        </apply>
      </apply>
      <apply>
        <times/>
        <apply>
          <plus/>
          <ci> species_16 </ci>
          <ci> mwa6f625ba_c9e6_40e4_bf00_d59146ed8f80 </ci>
        </apply>
        <apply>
          <plus/>
          <ci> species_13 </ci>
          <ci> mw28855de3_91da_4e09_b2c9_c0eb4b719af3 </ci>
        </apply>
      </apply>
    </apply>
  </math>
</assignmentRule>
<assignmentRule metaid="repeatedAssignment_mwfd935303_85b5_4619_8f29_6ce9dd7d5a54"
variable="mwb8fa5238_b6ab_43ba_914d_8d9609a94211">
  <math xmlns="http://www.w3.org/1998/Math/MathML">
    <apply>
      <divide/>
      <apply>
```

```
          <times/>
          <ci> mw5c6f658c_7714_4fbb_be83_5ef825b3e08b </ci>
          <ci> species_8 </ci>
        </apply>
        <apply>
          <plus/>
          <ci> species_8 </ci>
          <apply>
            <times/>
            <ci> mw2527d5af_e782_46fb_9b2c_875866d6ecd5 </ci>
            <apply>
              <plus/>
              <cn type="integer"> 1 </cn>
              <apply>
                <divide/>
                <ci> species_12 </ci>
                <ci> mw118d7663_7b18_410d_8ca7_fc168407ba10 </ci>
              </apply>
            </apply>
          </apply>
        </apply>
      </apply>
    </math>
  </assignmentRule>
  <assignmentRule metaid="repeatedAssignment_mw32890e00_1f5b_4b23_8928_f95a780ca700"
variable="mwd13e1918_33bb_4d69_9527_a7db921570eb">
    <math xmlns="http://www.w3.org/1998/Math/MathML">
      <apply>
```

```
<divide/>
<apply>
  <times/>
  <ci> mw6e8005dd_31e8_483e_902a_8870307bf558 </ci>
  <apply>
    <minus/>
    <apply>
      <times/>
      <ci> species_10 </ci>
      <ci> species_19 </ci>
    </apply>
    <apply>
      <divide/>
      <apply>
        <times/>
        <ci> species_18 </ci>
        <ci> species_17 </ci>
      </apply>
      <ci> mw075f4d45_ceca_4aad_bc29_89b6915e46f7 </ci>
    </apply>
  </apply>
</apply>
<apply>
  <times/>
  <ci> mwf8558c32_d374_4352_b837_54ce36f88a5e </ci>
  <ci> mwf187cf00_32ca_422a_9134_ffd278f81118 </ci>
  <apply>
```

<plus/>

<cn type="integer"> 1 </cn>

<apply>

  <times/>

  <apply>

    <plus/>

    <cn type="integer"> 1 </cn>

    <apply>

      <divide/>

      <ci> species_10 </ci>

      <ci> mw7d11f964_6e54_4584_8c91_1808e4e850fd </ci>

    </apply>

  </apply>

  <apply>

    <plus/>

    <apply>

      <divide/>

      <ci> species_15 </ci>

      <ci> mwe195dc18_fc3c_4329_905f_5bb2fc3efe19 </ci>

    </apply>

    <apply>

      <divide/>

      <ci> species_19 </ci>

      <ci> mwb61a7f15_d0aa_4449_85bb_2cdf4a397051 </ci>

    </apply>

  </apply>

</apply>

```
<apply>
  <divide/>
  <ci> species_10 </ci>
  <ci> mwe1915e65_ff53_471a_9d2c_b92b8d75f287 </ci>
</apply>
<apply>
  <divide/>
  <apply>
    <plus/>
    <apply>
      <times/>
      <ci> species_17 </ci>
      <apply>
        <plus/>
        <cn type="integer"> 1 </cn>
        <apply>
          <divide/>
          <apply>
            <plus/>
            <ci> species_16 </ci>
            <ci> species_18 </ci>
          </apply>
          <ci> mwf8558c32_d374_4352_b837_54ce36f88a5e </ci>
        </apply>
      </apply>
    </apply>
    <ci> species_16 </ci>
```

```
              <ci> species_18 </ci>
            </apply>
            <ci> mwf187cf00_32ca_422a_9134_ffd278f81118 </ci>
          </apply>
        </apply>
      </apply>
    </apply>
  </math>
</assignmentRule>
<assignmentRule metaid="repeatedAssignment_mwce49dbff_e807_40c8_88ca_13e833185c15"
variable="mwbe2ee4f3_36d2_4a42_8390_f8082ac79296">
  <math xmlns="http://www.w3.org/1998/Math/MathML">
    <apply>
      <divide/>
      <apply>
        <times/>
        <ci> mw4fbb318e_c400_4af5_8e3f_e80aec639ee3 </ci>
        <apply>
          <minus/>
          <ci> species_18 </ci>
          <apply>
            <divide/>
            <ci> species_20 </ci>
            <ci> mw94fab6bb_d1d8_4344_ac40_d9ea47173826 </ci>
          </apply>
        </apply>
      </apply>
      <apply>
```

<plus/>

<ci> species_18 </ci>

<ci> species_20 </ci>

</apply>

</apply>

</math>

</assignmentRule>

<assignmentRule metaid="repeatedAssignment_mw3972ea92_33f7_4539_a583_eaec1d4a5e27" variable="mw60c6f424_4d57_49bf_b130_811bec897e0a">

<math xmlns="http://www.w3.org/1998/Math/MathML">

<apply>

<divide/>

<apply>

<times/>

<ci> mw702a7f39_5675_48ad_953a_4bcbbdc8e1a2 </ci>

<apply>

<minus/>

<ci> species_17 </ci>

<apply>

<divide/>

<ci> species_20 </ci>

<ci> mw46a0d4e0_a24e_4d84_ac9d_1d098ed93c0b </ci>

</apply>

</apply>

</apply>

<apply>

<plus/>

<ci> species_17 </ci>

```
          &lt;ci&gt; species_20 &lt;/ci&gt;
        &lt;/apply&gt;
      &lt;/apply&gt;
    &lt;/math&gt;
  &lt;/assignmentRule&gt;
  <assignmentRule metaid="repeatedAssignment_mw445b8e5a_e800_4251_8a9b_4a4f77299cea"
variable="mw96deb747_ef33_4a50_b36e_145767b53cf7">
    &lt;math xmlns="http://www.w3.org/1998/Math/MathML"&gt;
      &lt;apply&gt;
        &lt;divide/&gt;
        &lt;apply&gt;
          &lt;times/&gt;
          &lt;ci&gt; mwd486ae05_3de1_42aa_9584_2edc458d0c59 &lt;/ci&gt;
          &lt;ci&gt; species_7 &lt;/ci&gt;
          &lt;ci&gt; species_20 &lt;/ci&gt;
        &lt;/apply&gt;
        &lt;apply&gt;
          &lt;times/&gt;
          &lt;apply&gt;
            &lt;plus/&gt;
            &lt;apply&gt;
              &lt;times/&gt;
              &lt;ci&gt; species_7 &lt;/ci&gt;
              &lt;apply&gt;
                &lt;plus/&gt;
                &lt;cn type="integer"&gt; 1 &lt;/cn&gt;
                &lt;apply&gt;
                  &lt;divide/&gt;
```

```
        <ci> species_4 </ci>
        <ci> mw16745cb2_2a9e_48f6_8cef_80e44ec67119 </ci>
      </apply>
    </apply>
  </apply>
  <apply>
    <times/>
    <ci> mwc1d6540f_78d6_40ae_8e94_d6db3d674a6e </ci>
    <apply>
      <plus/>
      <cn type="integer"> 1 </cn>
      <apply>
        <divide/>
        <ci> species_4 </ci>
        <ci> mw814665c4_d157_4663_a75e_af6f8f891fb0 </ci>
      </apply>
    </apply>
  </apply>
</apply>
<apply>
  <plus/>
  <ci> species_20 </ci>
  <apply>
    <times/>
    <ci> mw9c902b95_94d4_4dff_912f_eb93bd74d440 </ci>
    <apply>
      <plus/>
```

```
              <cn type="integer"> 1 </cn>
              <apply>
                <divide/>
                <ci> species_2 </ci>
                <ci> mwd947eee9_9a97_4305_a38f_5d0342490834 </ci>
              </apply>
              <apply>
                <divide/>
                <ci> species_3 </ci>
                <ci> mw0b12ed4b_ffff_47bd_a925_140813e42732 </ci>
              </apply>
              <apply>
                <divide/>
                <ci> species_12 </ci>
                <ci> mw6bb6ce48_f2df_4db7_abe8_95a611a6672b </ci>
              </apply>
            </apply>
          </apply>
        </apply>
      </apply>
    </apply>
  </math>
</assignmentRule>
<assignmentRule metaid="repeatedAssignment_mw28551e86_9575_4118_ba85_f632156e900b" variable="mw42c5ea06_edf8_46bd_b262_f9db84921975">
  <math xmlns="http://www.w3.org/1998/Math/MathML">
    <apply>
      <divide/>
```

```
          &lt;apply&gt;
            &lt;times/&gt;
            &lt;ci&gt; mwfb9d0842_5ad1_42a2_b44c_12cfeaa36962 &lt;/ci&gt;
            &lt;ci&gt; species_15 &lt;/ci&gt;
          &lt;/apply&gt;
          &lt;apply&gt;
            &lt;plus/&gt;
            &lt;ci&gt; species_15 &lt;/ci&gt;
            &lt;ci&gt; mwc59d3254_509c_411a_b656_9e80ac0a4d4a &lt;/ci&gt;
          &lt;/apply&gt;
        &lt;/apply&gt;
      &lt;/math&gt;
    &lt;/assignmentRule&gt;
    <assignmentRule metaid="repeatedAssignment_mw46ccbf09_9a03_480b_aa20_e3247fe474ce"
variable="mw10231dd9_d13a_4822_a477_0b190284526c">
      &lt;math xmlns="http://www.w3.org/1998/Math/MathML"&gt;
        &lt;apply&gt;
          &lt;plus/&gt;
          &lt;apply&gt;
            &lt;divide/&gt;
            &lt;apply&gt;
              &lt;times/&gt;
              &lt;ci&gt; mw4f571ae6_7040_40c6_b098_4ac7cf9ee24d &lt;/ci&gt;
              &lt;apply&gt;
                &lt;minus/&gt;
                &lt;ci&gt; species_2 &lt;/ci&gt;
                &lt;apply&gt;
                  &lt;divide/&gt;
```

```
        <ci> species_35 </ci>
        <ci> mw917c32b0_7a3f_4744_ac14_2e3d15da96f6 </ci>
      </apply>
    </apply>
  </apply>
  <apply>
    <times/>
    <ci> mw742fbec5_509e_42be_b7a8_12b9eeb49314 </ci>
    <apply>
      <plus/>
      <cn type="integer"> 1 </cn>
      <apply>
        <divide/>
        <ci> species_2 </ci>
        <ci> mw742fbec5_509e_42be_b7a8_12b9eeb49314 </ci>
      </apply>
      <apply>
        <divide/>
        <ci> species_35 </ci>
        <ci> mw231dd6ca_4db0_4cdb_adc8_72349abdba07 </ci>
      </apply>
    </apply>
  </apply>
</apply>
<apply>
  <divide/>
  <apply>
```

```
  <times/>
  <ci> mw2ec1703a_729a_4b49_b4dd_4241b0d73907 </ci>
  <apply>
    <minus/>
    <ci> species_2 </ci>
    <apply>
      <divide/>
      <ci> species_35 </ci>
      <ci> mw917c32b0_7a3f_4744_ac14_2e3d15da96f6 </ci>
    </apply>
  </apply>
</apply>
<apply>
  <times/>
  <ci> mwb99ebe97_7d91_406c_8b12_a56b2c939ff4 </ci>
  <apply>
    <plus/>
    <cn type="integer"> 1 </cn>
    <apply>
      <divide/>
      <ci> species_2 </ci>
      <ci> mwb99ebe97_7d91_406c_8b12_a56b2c939ff4 </ci>
    </apply>
    <apply>
      <divide/>
      <ci> species_35 </ci>
      <ci> mw23fae7dc_7d12_4fb0_98e2_c611e44007e1 </ci>
```

</apply>
</apply>
</apply>
</apply>
<apply>
<divide/>
<apply>
<times/>
<ci> mwa1879047_3c26_45c1_8b2a_137433500cde </ci>
<apply>
<minus/>
<ci> species_2 </ci>
<apply>
<divide/>
<ci> species_35 </ci>
<ci> mw917c32b0_7a3f_4744_ac14_2e3d15da96f6 </ci>
</apply>
</apply>
</apply>
<apply>
<times/>
<ci> mw6205c3fd_ecf9_4b2b_98f1_dff68729cd03 </ci>
<apply>
<plus/>
<cn type="integer"> 1 </cn>
<apply>
<divide/>

<ci> species_2 </ci>
<ci> mw6205c3fd_ecf9_4b2b_98f1_dff68729cd03 </ci>
</apply>
<apply>
<divide/>
<ci> species_35 </ci>
<ci> mw23f5979c_8188_4521_9629_6469c95e42d6 </ci>
</apply>
</apply>
</apply>
</apply>
</apply>
</math>
</assignmentRule>
<assignmentRule metaid="repeatedAssignment_mwa3220e90_eda8_4345_8304_6f586941e318" variable="mw0e384600_a904_40dc_b3a6_de20a7ae04b8">
<math xmlns="http://www.w3.org/1998/Math/MathML">
<apply>
<divide/>
<apply>
<times/>
<ci> mw68a26bf2_520b_4b35_90e0_511ec4bbc859 </ci>
<apply>
<minus/>
<ci> species_35 </ci>
<apply>
<divide/>
<ci> species_32 </ci>

```
            <ci> mwdacf4f8b_f1db_480a_aabf_ff89dc90e087 </ci>
           </apply>
          </apply>
         </apply>
         <apply>
          <times/>
          <ci> mwe806711a_3cbc_4d66_a530_d7ae4e673eea </ci>
          <apply>
           <plus/>
           <cn type="integer"> 1 </cn>
           <apply>
            <divide/>
            <ci> species_35 </ci>
            <ci> mwe806711a_3cbc_4d66_a530_d7ae4e673eea </ci>
           </apply>
           <apply>
            <divide/>
            <ci> species_32 </ci>
            <ci> mwdee0f9ac_28b4_444f_af80_30c299f78093 </ci>
           </apply>
          </apply>
         </apply>
        </apply>
      </math>
    </assignmentRule>
    <assignmentRule metaid="repeatedAssignment_mw852003bb_7c32_4815_8d4c_3d524e9f72e9" variable="mwaecebc73_e8e8_4aa8_a5c9_9d2295bf77d4">
      <math xmlns="http://www.w3.org/1998/Math/MathML">
```

```
<apply>
  <plus/>
  <apply>
    <divide/>
    <apply>
      <times/>
      <ci> mwd0f35f82_c657_4a53_9ac4_606e79cebb96 </ci>
      <apply>
        <minus/>
        <apply>
          <times/>
          <ci> species_10 </ci>
          <ci> species_11 </ci>
        </apply>
        <apply>
          <times/>
          <ci> mw1a31f571_e225_4d2d_b672_bb6209080e70 </ci>
          <ci> species_2 </ci>
          <ci> species_5 </ci>
        </apply>
      </apply>
    </apply>
    <apply>
      <times/>
      <apply>
        <plus/>
        <cn type="integer"> 1 </cn>
```

```
      <apply>
        <divide/>
        <ci> species_10 </ci>
        <ci> mw2a519375_b634_461c_a4e7_2205e166b3ce </ci>
      </apply>
      <apply>
        <divide/>
        <ci> species_2 </ci>
        <ci> mw25dcc228_99f4_4d62_badc_00067a7db23a </ci>
      </apply>
    </apply>
    <apply>
      <plus/>
      <cn type="integer"> 1 </cn>
      <apply>
        <divide/>
        <ci> species_11 </ci>
        <ci> mw02659a3e_942b_4113_928b_885d34341771 </ci>
      </apply>
      <apply>
        <divide/>
        <ci> species_5 </ci>
        <ci> mw31fc107e_644a_475e_aeff_2b016202c816 </ci>
      </apply>
    </apply>
  </apply>
</apply>
```

```
<apply>
  <divide/>
  <apply>
    <times/>
    <ci> mw03434a4a_4ed9_491d_bb10_f39e62b3acb4 </ci>
    <apply>
      <minus/>
      <apply>
        <times/>
        <ci> species_10 </ci>
        <ci> species_11 </ci>
      </apply>
      <apply>
        <times/>
        <ci> mw1a31f571_e225_4d2d_b672_bb6209080e70 </ci>
        <ci> species_2 </ci>
        <ci> species_5 </ci>
      </apply>
    </apply>
  </apply>
  <apply>
    <times/>
    <apply>
      <plus/>
      <cn type="integer"> 1 </cn>
      <apply>
        <divide/>
```

```
          &lt;ci&gt; species_10 &lt;/ci&gt;
          &lt;ci&gt; mw04a2d768_b6a0_4a14_8c35_7cee82a7a308 &lt;/ci&gt;
        &lt;/apply&gt;
        &lt;apply&gt;
          &lt;divide/&gt;
          &lt;ci&gt; species_2 &lt;/ci&gt;
          &lt;ci&gt; mw18e6c815_3a2d_4abe_8f8c_96b098a1ffe4 &lt;/ci&gt;
        &lt;/apply&gt;
      &lt;/apply&gt;
      &lt;apply&gt;
        &lt;plus/&gt;
        &lt;cn type="integer"&gt; 1 &lt;/cn&gt;
        &lt;apply&gt;
          &lt;divide/&gt;
          &lt;ci&gt; species_11 &lt;/ci&gt;
          &lt;ci&gt; mwa177ed29_1ff2_4ddf_aa7c_8cc4c08a1a2d &lt;/ci&gt;
        &lt;/apply&gt;
        &lt;apply&gt;
          &lt;divide/&gt;
          &lt;ci&gt; species_5 &lt;/ci&gt;
          &lt;ci&gt; mw0d8a79b9_2494_4bbb_8324_70f0b2e27188 &lt;/ci&gt;
        &lt;/apply&gt;
      &lt;/apply&gt;
    &lt;/apply&gt;
  &lt;/apply&gt;
&lt;/apply&gt;
&lt;/math&gt;
```

```
    </assignmentRule>
    <assignmentRule metaid="repeatedAssignment_mwed7d8988_ccb0_4ac6_ad57_3713827b2915"
variable="mwd008246b_4ac0_4550_9588_64a1e8664cd1">
      <math xmlns="http://www.w3.org/1998/Math/MathML">
        <apply>
          <divide/>
          <apply>
            <times/>
            <ci> mw5723da7e_7ee9_4b45_b397_c4fad0251fa2 </ci>
            <ci> species_32 </ci>
          </apply>
          <apply>
            <plus/>
            <ci> mw70b2b9c1_0904_4851_aee4_8526d26a4c7f </ci>
            <ci> species_32 </ci>
          </apply>
        </apply>
      </math>
    </assignmentRule>
    <assignmentRule
metaid="repeatedAssignment_mw8d013e69_5904_4483_a44b_5579e7810e9b"
variable="mwf7b66b1f_77b0_4024_99e5_fb37fb0ab929">
      <math xmlns="http://www.w3.org/1998/Math/MathML">
        <apply>
          <divide/>
          <apply>
            <times/>
            <ci> mw2fcbcb1c_c103_48bb_86ed_9229a27de6ed </ci>
            <ci> species_21 </ci>
```

```
          </apply>
          <apply>
            <plus/>
            <ci> mw52505a99_c3c7_4c4c_8a62_b891bfab4dd8 </ci>
            <ci> species_21 </ci>
          </apply>
        </apply>
      </math>
    </assignmentRule>
    <assignmentRule metaid="repeatedAssignment_mw0393ec44_48a7_474b_9e3a_603d01dd17c3" variable="mw6eb33847_58dd_4306_8ae9_7d68bb2a5612">
      <math xmlns="http://www.w3.org/1998/Math/MathML">
        <apply>
          <divide/>
          <apply>
            <times/>
            <ci> mwe110663f_33e9_415c_96f8_04e92e007bb4 </ci>
            <ci> species_10 </ci>
          </apply>
          <apply>
            <plus/>
            <ci> mw395deb93_b80e_4154_b71a_add84953daaf </ci>
            <ci> species_10 </ci>
          </apply>
        </apply>
      </math>
    </assignmentRule>
```

```
<assignmentRule metaid="repeatedAssignment_mwc9e36a25_0dfd_4cc2_b0de_8637fa3ef59c" variable="mw4a42b5e7_485f_4d6b_a4ed_e490759e5f26">
 <math xmlns="http://www.w3.org/1998/Math/MathML">
  <apply>
   <divide/>
   <apply>
    <times/>
    <ci> mw715a0d4a_6423_4755_a1b5_7d9e5b1852a2 </ci>
    <ci> species_16 </ci>
   </apply>
   <apply>
    <plus/>
    <ci> mw4d3fe418_9692_4308_a1c1_64b6e3b6ad82 </ci>
    <ci> species_16 </ci>
   </apply>
  </apply>
 </math>
</assignmentRule>
<assignmentRule metaid="repeatedAssignment_mwbeb83c0b_9d75_4760_9310_bab51ec60c97" variable="mw3128a56f_9005_484d_92b7_1b5e45b577c6">
 <math xmlns="http://www.w3.org/1998/Math/MathML">
  <apply>
   <divide/>
   <apply>
    <times/>
    <ci> mw6caa1525_e69b_4ab2_b8c7_2b7deeb5ee58 </ci>
    <ci> species_18 </ci>
   </apply>
```

```
          <apply>
            <plus/>
            <ci> mwe871df2e_a7b7_49c5_8a95_809524380cbd </ci>
            <ci> species_18 </ci>
          </apply>
        </apply>
      </math>
    </assignmentRule>
    <assignmentRule metaid="repeatedAssignment_mwb001e092_570f_4ac1_926a_98c5c204f23d" variable="mwca272a09_38a7_4416_a781_d64c8e945cc4">
      <math xmlns="http://www.w3.org/1998/Math/MathML">
        <apply>
          <divide/>
          <apply>
            <times/>
            <ci> mw72f442a2_e01a_44d3_b8bf_9d223245f350 </ci>
            <ci> species_15 </ci>
          </apply>
          <apply>
            <plus/>
            <ci> species_15 </ci>
            <ci> mwa27b7723_ab20_4324_a886_f5c73588ac4a </ci>
          </apply>
        </apply>
      </math>
    </assignmentRule>
    <assignmentRule metaid="repeatedAssignment_mwc6009a2d_977f_4bcd_995b_52321ed9adc5" variable="mwf41678a2_15b0_484e_b056_0c8a195fed4d">
```

```xml
<math xmlns="http://www.w3.org/1998/Math/MathML">
  <apply>
    <divide/>
    <apply>
      <times/>
      <ci> mw84e3d5fb_d3f5_4a42_a963_2d0e4de0c9fa </ci>
      <ci> species_21 </ci>
    </apply>
    <apply>
      <times/>
      <ci> mw212994b8_ca26_4fbc_a13e_bb054a312812 </ci>
      <apply>
        <plus/>
        <cn type="integer"> 1 </cn>
        <apply>
          <divide/>
          <ci> species_21 </ci>
          <ci> mw212994b8_ca26_4fbc_a13e_bb054a312812 </ci>
        </apply>
        <apply>
          <divide/>
          <ci> mw3447e887_c5e2_4271_9e5f_50c309bc8dc3 </ci>
          <ci> mw80012d61_bf17_467d_8029_8785e8b66833 </ci>
        </apply>
      </apply>
    </apply>
  </apply>
```

```
      </math>
     </assignmentRule>
    </listOfRules>
    <listOfReactions>
     <reaction id="reaction_1" name="CC_1 (RuBisCO)" reversible="false" fast="false">
      <annotation>
       <COPASI xmlns="http://www.copasi.org/static/sbml">
        <rdf:RDF xmlns:dcterms="http://purl.org/dc/terms/"
xmlns:rdf="http://www.w3.org/1999/02/22-rdf-syntax-ns#">
         <rdf:Description rdf:about="#COPASI49">
          <dcterms:created>
           <rdf:Description>
            <dcterms:W3CDTF>2010-04-14T14:47:32Z</dcterms:W3CDTF>
           </rdf:Description>
          </dcterms:created>
         </rdf:Description>
        </rdf:RDF>
       </COPASI>
      </annotation>
      <listOfReactants>
       <speciesReference species="species_3"/>
       <speciesReference species="species_1"/>
       <speciesReference species="species_6"/>
       <speciesReference species="species_14"/>
       <speciesReference species="species_8"/>
       <speciesReference species="species_12"/>
       <speciesReference species="species_5"/>
      </listOfReactants>
```

```
<listOfProducts>
  <speciesReference species="species_2" stoichiometry="2"/>
  <speciesReference species="species_6"/>
  <speciesReference species="species_14"/>
  <speciesReference species="species_8"/>
  <speciesReference species="species_12"/>
  <speciesReference species="species_5"/>
</listOfProducts>
<kineticLaw>
  <math xmlns="http://www.w3.org/1998/Math/MathML">
    <apply>
      <divide/>
      <apply>
        <times/>
        <ci> species_3 </ci>
        <apply>
          <divide/>
          <apply>
            <times/>
            <ci> mwf008a49f_a4ec_49ec_8cdc_30906d544233 </ci>
            <ci> species_1 </ci>
          </apply>
          <apply>
            <plus/>
            <ci> species_1 </ci>
            <apply>
              <times/>
```

<ci> mwdec52954_3ec3_416b_8d1c_d8191ee0052a </ci>

<apply>

<plus/>

<cn type="integer"> 1 </cn>

<apply>

<divide/>

<ci> species_6 </ci>

<ci> mw67c2b3cc_db05_4a74_97af_8d7314b6868b </ci>

</apply>

</apply>

</apply>

</apply>

</apply>

</apply>

<apply>

<plus/>

<ci> species_3 </ci>

<apply>

<times/>

<ci> mw49a330a3_2804_4b34_bed5_55979f9f820c </ci>

<apply>

<plus/>

<cn type="integer"> 1 </cn>

<apply>

<divide/>

<ci> species_2 </ci>

<ci> mwa4c7bb69_e0a6_4881_b8c4_8bb392a17249 </ci>

</apply>

<apply>

<divide/>

<ci> species_14 </ci>

<ci> mwaab87003_70c0_4690_a563_726e460f9c64 </ci>

</apply>

<apply>

<divide/>

<ci> species_8 </ci>

<ci> mw8642dd0a_5a0f_45a5_b0ca_d246e508136b </ci>

</apply>

<apply>

<divide/>

<ci> species_12 </ci>

<ci> mwa496f448_c225_4611_af1e_f7f3dfa7977c </ci>

</apply>

<apply>

<divide/>

<ci> species_5 </ci>

<ci> mw7aeaf567_e744_423f_b2c4_3aeb34386eb6 </ci>

</apply>

</apply>

</apply>

</apply>

</apply>

</math>

</kineticLaw>

```
</reaction>
<reaction id="reaction_2" name="CC_2 (phosphoglycerate kinase)" reversible="false" fast="false">
  <notes>
    <body xmlns="http://www.w3.org/1999/xhtml">
      <pre>Vmax sn&#xeD;&#x17e;eno z 10.3 na 2</pre>
    </body>
  </notes>
  <annotation>
    <COPASI xmlns="http://www.copasi.org/static/sbml">
      <rdf:RDF xmlns:dcterms="http://purl.org/dc/terms/" xmlns:rdf="http://www.w3.org/1999/02/22-rdf-syntax-ns#">
        <rdf:Description rdf:about="#COPASI50">
          <dcterms:created>
            <rdf:Description>
              <dcterms:W3CDTF>2010-04-14T15:57:26Z</dcterms:W3CDTF>
            </rdf:Description>
          </dcterms:created>
        </rdf:Description>
      </rdf:RDF>
    </COPASI>
  </annotation>
  <listOfReactants>
    <speciesReference species="species_2"/>
    <speciesReference species="species_7"/>
  </listOfReactants>
  <listOfProducts>
    <speciesReference species="species_9"/>
```

```
  <speciesReference species="species_4"/>
</listOfProducts>
<kineticLaw>
  <math xmlns="http://www.w3.org/1998/Math/MathML">
    <apply>
      <divide/>
      <apply>
        <times/>
        <ci> mwfe139de1_b141_420e_a4e5_f59c639c6cf0 </ci>
        <apply>
          <minus/>
          <apply>
            <times/>
            <ci> species_2 </ci>
            <ci> species_7 </ci>
          </apply>
          <apply>
            <divide/>
            <apply>
              <times/>
              <ci> species_9 </ci>
              <ci> species_4 </ci>
            </apply>
            <ci> mwbd9ba0b8_46b5_43a7_894a_1e0a7450c854 </ci>
          </apply>
        </apply>
      </apply>
```

```
    <apply>
      <times/>
      <apply>
        <plus/>
        <ci> species_2 </ci>
        <ci> mw33c77066_cf3c_44c1_9e75_e85e2c99a3fd </ci>
      </apply>
      <apply>
        <plus/>
        <ci> species_7 </ci>
        <apply>
          <times/>
          <ci> mwfd3ba144_51dd_4c75_b0e1_8926ab5477e8 </ci>
          <apply>
            <plus/>
            <cn type="integer"> 1 </cn>
            <apply>
              <divide/>
              <ci> species_4 </ci>
              <ci> mwb70b9188_7453_4609_ad48_be4bf69f87e4 </ci>
            </apply>
          </apply>
        </apply>
      </apply>
    </apply>
  </apply>
</math>
```

</kineticLaw>

</reaction>

<reaction id="reaction_3" name="CC_3 (glyceraldehyde 3-phosphate dehydrogenase)" reversible="false" fast="false">

<annotation>

<COPASI xmlns="http://www.copasi.org/static/sbml">

<rdf:RDF xmlns:dcterms="http://purl.org/dc/terms/" xmlns:rdf="http://www.w3.org/1999/02/22-rdf-syntax-ns#">

<rdf:Description rdf:about="#COPASI51">

<dcterms:created>

<rdf:Description>

<dcterms:W3CDTF>2010-04-14T16:10:42Z</dcterms:W3CDTF>

</rdf:Description>

</dcterms:created>

</rdf:Description>

</rdf:RDF>

</COPASI>

</annotation>

<listOfReactants>

<speciesReference species="species_9"/>

<speciesReference species="species_5"/>

</listOfReactants>

<listOfProducts>

<speciesReference species="species_10"/>

<speciesReference species="species_11"/>

<speciesReference species="species_12"/>

</listOfProducts>

<kineticLaw>

```
    <math xmlns="http://www.w3.org/1998/Math/MathML">
      <apply>
        <divide/>
        <apply>
          <times/>
          <ci> mwc727e6a6_e76e_44d0_89f4_bede015cb04c </ci>
          <ci> species_9 </ci>
          <ci> species_5 </ci>
        </apply>
        <apply>
          <times/>
          <apply>
            <plus/>
            <ci> species_9 </ci>
            <ci> mw701b6fb7_2279_4661_a2d5_e1e2e9f47203 </ci>
          </apply>
          <apply>
            <plus/>
            <ci> species_5 </ci>
            <ci> mw9ea5d254_32bc_4a37_aa1f_f4b94da8eadb </ci>
          </apply>
        </apply>
      </apply>
    </math>
  </kineticLaw>
</reaction>
<reaction id="reaction_4" name="CC_4 (triose phosphate isomerase)" fast="false">
```

```
      <annotation>
        <COPASI xmlns="http://www.copasi.org/static/sbml">
          <rdf:RDF xmlns:dcterms="http://purl.org/dc/terms/"
xmlns:rdf="http://www.w3.org/1999/02/22-rdf-syntax-ns#">
            <rdf:Description rdf:about="#COPASI52">
              <dcterms:created>
                <rdf:Description>
                  <dcterms:W3CDTF>2010-04-14T16:18:37Z</dcterms:W3CDTF>
                </rdf:Description>
              </dcterms:created>
            </rdf:Description>
          </rdf:RDF>
        </COPASI>
      </annotation>
      <listOfReactants>
        <speciesReference species="species_10"/>
      </listOfReactants>
      <listOfProducts>
        <speciesReference species="species_13"/>
      </listOfProducts>
      <kineticLaw>
        <math xmlns="http://www.w3.org/1998/Math/MathML">
          <apply>
            <divide/>
            <apply>
              <times/>
              <ci> mw8c8ad41e_6d63_473b_960b_322d5feb6f84 </ci>
              <apply>
```

```
        <minus/>
        <ci> species_10 </ci>
        <apply>
          <divide/>
          <ci> species_13 </ci>
          <ci> mwdde5b27b_4aa1_42aa_b292_6613ea7584a3 </ci>
        </apply>
      </apply>
    </apply>
    <apply>
      <plus/>
      <ci> species_10 </ci>
      <ci> species_13 </ci>
    </apply>
  </apply>
</math>
</kineticLaw>
</reaction>
<reaction id="reaction_5" name="CC_5 (aldolase)" fast="false">
  <annotation>
    <COPASI xmlns="http://www.copasi.org/static/sbml">
      <rdf:RDF xmlns:dcterms="http://purl.org/dc/terms/"
xmlns:rdf="http://www.w3.org/1999/02/22-rdf-syntax-ns#">
        <rdf:Description rdf:about="#COPASI53">
          <dcterms:created>
            <rdf:Description>
              <dcterms:W3CDTF>2010-04-14T16:46:06Z</dcterms:W3CDTF>
            </rdf:Description>
```

```
      </dcterms:created>
     </rdf:Description>
    </rdf:RDF>
  </COPASI>
</annotation>
<listOfReactants>
  <speciesReference species="species_10"/>
  <speciesReference species="species_13"/>
</listOfReactants>
<listOfProducts>
  <speciesReference species="species_14"/>
</listOfProducts>
<kineticLaw>
  <math xmlns="http://www.w3.org/1998/Math/MathML">
    <apply>
     <divide/>
     <apply>
      <times/>
      <ci> mwdbdd129f_0428_4a42_8427_cf18a0b5568c </ci>
      <apply>
       <minus/>
       <apply>
        <times/>
        <ci> species_10 </ci>
        <ci> species_13 </ci>
       </apply>
       <apply>
```

```
      <divide/>
      <ci> species_14 </ci>
      <ci> mw245d5cf2_30db_40c3_b1a7_ed389fff8808 </ci>
    </apply>
  </apply>
</apply>
<apply>
  <times/>
  <ci> mw39e8b6c4_2ae7_4846_9ac8_e77ee9c7191f </ci>
  <ci> mw54c31bc3_16fc_462a_a55e_a766a97769ed </ci>
  <apply>
    <plus/>
    <cn type="integer"> 1 </cn>
    <apply>
      <divide/>
      <ci> species_10 </ci>
      <ci> mw39e8b6c4_2ae7_4846_9ac8_e77ee9c7191f </ci>
    </apply>
    <apply>
      <divide/>
      <ci> species_13 </ci>
      <ci> mw54c31bc3_16fc_462a_a55e_a766a97769ed </ci>
    </apply>
    <apply>
      <divide/>
      <ci> species_14 </ci>
      <ci> mwbad28d74_abec_451a_a6bf_a03335045383 </ci>
```

```
              </apply>
              <apply>
                <divide/>
                <apply>
                  <times/>
                  <ci> species_10 </ci>
                  <ci> species_13 </ci>
                </apply>
                <apply>
                  <times/>
                  <ci> mw39e8b6c4_2ae7_4846_9ac8_e77ee9c7191f </ci>
                  <ci> mw54c31bc3_16fc_462a_a55e_a766a97769ed </ci>
                </apply>
              </apply>
            </apply>
          </apply>
        </apply>
      </math>
    </kineticLaw>
  </reaction>
  <reaction id="reaction_6" name="CC_6 alpha (fructose-1,6-bisphosphatase)" reversible="false"
fast="false">
    <annotation>
      <COPASI xmlns="http://www.copasi.org/static/sbml">
        <rdf:RDF xmlns:dcterms="http://purl.org/dc/terms/"
xmlns:rdf="http://www.w3.org/1999/02/22-rdf-syntax-ns#">
          <rdf:Description rdf:about="#COPASI54">
            <dcterms:created>
```

```
        <rdf:Description>
          <dcterms:W3CDTF>2010-04-14T16:52:19Z</dcterms:W3CDTF>
        </rdf:Description>
      </dcterms:created>
    </rdf:Description>
  </rdf:RDF>
</COPASI>
</annotation>
<listOfReactants>
  <speciesReference species="species_14"/>
</listOfReactants>
<listOfProducts>
  <speciesReference species="species_15"/>
  <speciesReference species="species_12"/>
</listOfProducts>
<kineticLaw>
  <math xmlns="http://www.w3.org/1998/Math/MathML">
    <apply>
      <divide/>
      <apply>
        <times/>
        <ci> mw9dda631f_8096_4e56_a670_fdb559f36d12 </ci>
        <ci> species_14 </ci>
      </apply>
      <apply>
        <plus/>
        <ci> species_14 </ci>
```

<apply>
<times/>
<ci> mwe6b619dd_229f_4afa_af14_be3a25b0e262 </ci>
<apply>
<plus/>
<cn type="integer"> 1 </cn>
<apply>
<divide/>
<ci> species_15 </ci>
<ci> mwa90c1c5f_dcc6_48a5_a99f_ce08e7c2c91b </ci>
</apply>
<apply>
<divide/>
<ci> species_12 </ci>
<ci> mw9f468e68_fa4c_4260_a5af_3b6182abcb92 </ci>
</apply>
</apply>
</apply>
</apply>
</apply>
</math>
</kineticLaw>
</reaction>
<reaction id="reaction_7" name="CC_7 (transketolase)" fast="false">
<annotation>
<COPASI xmlns="http://www.copasi.org/static/sbml">
<rdf:RDF xmlns:dcterms="http://purl.org/dc/terms/" xmlns:rdf="http://www.w3.org/1999/02/22-rdf-syntax-ns#">

```
      <rdf:Description rdf:about="#COPASI55">
        <dcterms:created>
          <rdf:Description>
            <dcterms:W3CDTF>2010-04-15T18:06:55Z</dcterms:W3CDTF>
          </rdf:Description>
        </dcterms:created>
      </rdf:Description>
    </rdf:RDF>
  </COPASI>
</annotation>
<listOfReactants>
  <speciesReference species="species_15"/>
  <speciesReference species="species_10"/>
  <speciesReference species="species_19"/>
  <speciesReference species="species_18"/>
</listOfReactants>
<listOfProducts>
  <speciesReference species="species_16"/>
  <speciesReference species="species_17"/>
  <speciesReference species="species_19"/>
  <speciesReference species="species_18"/>
</listOfProducts>
<kineticLaw>
  <math xmlns="http://www.w3.org/1998/Math/MathML">
    <apply>
      <divide/>
      <apply>
```

```
  <times/>
  <ci> mwea0f726e_b6ff_4cf0_8dba_9db4cc0827be </ci>
  <apply>
    <minus/>
    <apply>
      <times/>
      <ci> species_15 </ci>
      <ci> species_10 </ci>
    </apply>
    <apply>
      <divide/>
      <apply>
        <times/>
        <ci> species_17 </ci>
        <ci> species_16 </ci>
      </apply>
      <ci> mw4ec82ea3_67a6_47ad_971a_52a33f180466 </ci>
    </apply>
  </apply>
</apply>
<apply>
  <times/>
  <apply>
    <plus/>
    <ci> species_15 </ci>
    <apply>
      <times/>
```

```xml
          <ci> mwd037be1a_7ea7_4d03_b00e_8f358afc43f0 </ci>
          <apply>
            <plus/>
            <cn type="integer"> 1 </cn>
            <apply>
              <divide/>
              <ci> species_17 </ci>
              <ci> mw6990314a_e646_4a51_b298_7b53a5a3ca3b </ci>
            </apply>
            <apply>
              <divide/>
              <ci> species_16 </ci>
              <ci> mwd59eae5a_ff1f_4351_922c_d25f34ca05ac </ci>
            </apply>
          </apply>
        </apply>
      </apply>
      <apply>
        <plus/>
        <ci> species_10 </ci>
        <ci> mwd32ff45c_71b0_4916_8ffc_7683019b9311 </ci>
      </apply>
    </apply>
  </apply>
</math>
</kineticLaw>
</reaction>
```

```
      <reaction id="reaction_8" name="CC_8 (aldolase)" fast="false">
        <annotation>
          <COPASI xmlns="http://www.copasi.org/static/sbml">
            <rdf:RDF xmlns:dcterms="http://purl.org/dc/terms/"
xmlns:rdf="http://www.w3.org/1999/02/22-rdf-syntax-ns#">
              <rdf:Description rdf:about="#COPASI56">
                <dcterms:created>
                  <rdf:Description>
                    <dcterms:W3CDTF>2010-04-20T11:20:56Z</dcterms:W3CDTF>
                  </rdf:Description>
                </dcterms:created>
              </rdf:Description>
            </rdf:RDF>
          </COPASI>
        </annotation>
        <listOfReactants>
          <speciesReference species="species_13"/>
          <speciesReference species="species_16"/>
        </listOfReactants>
        <listOfProducts>
          <speciesReference species="species_8"/>
        </listOfProducts>
        <kineticLaw>
          <math xmlns="http://www.w3.org/1998/Math/MathML">
            <apply>
              <divide/>
              <apply>
                <times/>
```

```
  <ci> mw33ab4422_ba35_4d2b_9620_d274168c7e1d </ci>
  <apply>
   <minus/>
   <apply>
    <times/>
    <ci> species_13 </ci>
    <ci> species_16 </ci>
   </apply>
   <apply>
    <divide/>
    <ci> species_8 </ci>
    <ci> mwf44df5f6_9e91_4081_a77d_911fe8867849 </ci>
   </apply>
  </apply>
 </apply>
 <apply>
  <times/>
  <apply>
   <plus/>
   <ci> species_16 </ci>
   <ci> mwa6f625ba_c9e6_40e4_bf00_d59146ed8f80 </ci>
  </apply>
  <apply>
   <plus/>
   <ci> species_13 </ci>
   <ci> mw28855de3_91da_4e09_b2c9_c0eb4b719af3 </ci>
  </apply>
```

</apply>

</apply>

</math>

</kineticLaw>

</reaction>

<reaction id="reaction_9" name="CC_9 (sedoheptulose-1,7 bisphosphatase)" reversible="false" fast="false">

<annotation>

<COPASI xmlns="http://www.copasi.org/static/sbml">

<rdf:RDF xmlns:dcterms="http://purl.org/dc/terms/" xmlns:rdf="http://www.w3.org/1999/02/22-rdf-syntax-ns#">

<rdf:Description rdf:about="#COPASI57">

<dcterms:created>

<rdf:Description>

<dcterms:W3CDTF>2010-04-21T02:31:25Z</dcterms:W3CDTF>

</rdf:Description>

</dcterms:created>

</rdf:Description>

</rdf:RDF>

</COPASI>

</annotation>

<listOfReactants>

<speciesReference species="species_8"/>

<speciesReference species="species_12"/>

</listOfReactants>

<listOfProducts>

<speciesReference species="species_19"/>

<speciesReference species="species_12"/>

```
  <speciesReference species="species_12"/>
</listOfProducts>
<kineticLaw>
  <math xmlns="http://www.w3.org/1998/Math/MathML">
    <apply>
      <divide/>
      <apply>
        <times/>
        <ci> mw5c6f658c_7714_4fbb_be83_5ef825b3e08b </ci>
        <ci> species_8 </ci>
      </apply>
      <apply>
        <plus/>
        <ci> species_8 </ci>
        <apply>
          <times/>
          <ci> mw2527d5af_e782_46fb_9b2c_875866d6ecd5 </ci>
          <apply>
            <plus/>
            <cn type="integer"> 1 </cn>
            <apply>
              <divide/>
              <ci> species_12 </ci>
              <ci> mw118d7663_7b18_410d_8ca7_fc168407ba10 </ci>
            </apply>
          </apply>
        </apply>
```

```
          </apply>
        </apply>
      </math>
    </kineticLaw>
  </reaction>
  <reaction id="reaction_10" name="CC_10 (transketolase)" fast="false">
    <annotation>
      <COPASI xmlns="http://www.copasi.org/static/sbml">
        <rdf:RDF xmlns:dcterms="http://purl.org/dc/terms/"
xmlns:rdf="http://www.w3.org/1999/02/22-rdf-syntax-ns#">
          <rdf:Description rdf:about="#COPASI58">
            <dcterms:created>
              <rdf:Description>
                <dcterms:W3CDTF>2010-04-21T03:46:16Z</dcterms:W3CDTF>
              </rdf:Description>
            </dcterms:created>
          </rdf:Description>
        </rdf:RDF>
      </COPASI>
    </annotation>
    <listOfReactants>
      <speciesReference species="species_19"/>
      <speciesReference species="species_10"/>
      <speciesReference species="species_15"/>
      <speciesReference species="species_16"/>
    </listOfReactants>
    <listOfProducts>
      <speciesReference species="species_18"/>
```

```
  <speciesReference species="species_17"/>
  <speciesReference species="species_15"/>
  <speciesReference species="species_16"/>
</listOfProducts>
<kineticLaw>
  <math xmlns="http://www.w3.org/1998/Math/MathML">
    <apply>
      <divide/>
      <apply>
        <times/>
        <ci> mw6e8005dd_31e8_483e_902a_8870307bf558 </ci>
        <apply>
          <minus/>
          <apply>
            <times/>
            <ci> species_10 </ci>
            <ci> species_19 </ci>
          </apply>
          <apply>
            <divide/>
            <apply>
              <times/>
              <ci> species_18 </ci>
              <ci> species_17 </ci>
            </apply>
            <ci> mw075f4d45_ceca_4aad_bc29_89b6915e46f7 </ci>
          </apply>
```

</apply>
</apply>
<apply>
<times/>
<ci> mwf8558c32_d374_4352_b837_54ce36f88a5e </ci>
<ci> mwf187cf00_32ca_422a_9134_ffd278f81118 </ci>
<apply>
<plus/>
<cn type="integer"> 1 </cn>
<apply>
<times/>
<apply>
<plus/>
<cn type="integer"> 1 </cn>
<apply>
<divide/>
<ci> species_10 </ci>
<ci> mw7d11f964_6e54_4584_8c91_1808e4e850fd </ci>
</apply>
</apply>
<apply>
<plus/>
<apply>
<divide/>
<ci> species_15 </ci>
<ci> mwe195dc18_fc3c_4329_905f_5bb2fc3efe19 </ci>
</apply>

```
    <apply>
      <divide/>
      <ci> species_19 </ci>
      <ci> mwb61a7f15_d0aa_4449_85bb_2cdf4a397051 </ci>
    </apply>
  </apply>
</apply>
<apply>
  <divide/>
  <ci> species_10 </ci>
  <ci> mwe1915e65_ff53_471a_9d2c_b92b8d75f287 </ci>
</apply>
<apply>
  <divide/>
  <apply>
    <plus/>
    <apply>
      <times/>
      <ci> species_17 </ci>
      <apply>
        <plus/>
        <cn type="integer"> 1 </cn>
        <apply>
          <divide/>
          <apply>
            <plus/>
            <ci> species_16 </ci>
```

<ci> species_18 </ci>
</apply>
<ci> mwf8558c32_d374_4352_b837_54ce36f88a5e </ci>
</apply>
</apply>
</apply>
<ci> species_16 </ci>
<ci> species_18 </ci>
</apply>
<ci> mwf187cf00_32ca_422a_9134_ffd278f81118 </ci>
</apply>
</apply>
</apply>
</apply>
</math>
</kineticLaw>
</reaction>
<reaction id="reaction_11" name="C_11 (phosphopentose isomerase)" fast="false">
<annotation>
<COPASI xmlns="http://www.copasi.org/static/sbml">
<rdf:RDF xmlns:dcterms="http://purl.org/dc/terms/" xmlns:rdf="http://www.w3.org/1999/02/22-rdf-syntax-ns#">
<rdf:Description rdf:about="#COPASI59">
<dcterms:created>
<rdf:Description>
<dcterms:W3CDTF>2010-04-23T03:43:39Z</dcterms:W3CDTF>
</rdf:Description>
</dcterms:created>

```
      </rdf:Description>
    </rdf:RDF>
  </COPASI>
</annotation>
<listOfReactants>
  <speciesReference species="species_18"/>
</listOfReactants>
<listOfProducts>
  <speciesReference species="species_20"/>
</listOfProducts>
<kineticLaw>
  <math xmlns="http://www.w3.org/1998/Math/MathML">
    <apply>
      <divide/>
      <apply>
        <times/>
        <ci> mw4fbb318e_c400_4af5_8e3f_e80aec639ee3 </ci>
        <apply>
          <minus/>
          <ci> species_18 </ci>
          <apply>
            <divide/>
            <ci> species_20 </ci>
            <ci> mw94fab6bb_d1d8_4344_ac40_d9ea47173826 </ci>
          </apply>
        </apply>
      </apply>
```

```
            <apply>
              <times/>
              <ci> mwf2185be5_b4b0_4bf4_a6d3_0a758e4fbcca </ci>
              <apply>
                <plus/>
                <cn type="integer"> 1 </cn>
                <apply>
                  <divide/>
                  <ci> species_18 </ci>
                  <ci> mwf2185be5_b4b0_4bf4_a6d3_0a758e4fbcca </ci>
                </apply>
                <apply>
                  <divide/>
                  <ci> species_20 </ci>
                  <ci> mw9168b04c_6d3a_4f60_8906_5b5293d6a01e </ci>
                </apply>
              </apply>
            </apply>
          </apply>
        </math>
      </kineticLaw>
    </reaction>
    <reaction id="reaction_12" name="C_12 (phosphopentose epimerase)" fast="false">
      <annotation>
        <COPASI xmlns="http://www.copasi.org/static/sbml">
          <rdf:RDF xmlns:dcterms="http://purl.org/dc/terms/"
xmlns:rdf="http://www.w3.org/1999/02/22-rdf-syntax-ns#">
            <rdf:Description rdf:about="#COPASI60">
```

```
      &lt;dcterms:created&gt;
        &lt;rdf:Description&gt;
          &lt;dcterms:W3CDTF&gt;2010-04-23T03:43:48Z&lt;/dcterms:W3CDTF&gt;
        &lt;/rdf:Description&gt;
      &lt;/dcterms:created&gt;
    &lt;/rdf:Description&gt;
  &lt;/rdf:RDF&gt;
&lt;/COPASI&gt;
&lt;/annotation&gt;
&lt;listOfReactants&gt;
  &lt;speciesReference species="species_17"/&gt;
&lt;/listOfReactants&gt;
&lt;listOfProducts&gt;
  &lt;speciesReference species="species_20"/&gt;
&lt;/listOfProducts&gt;
&lt;kineticLaw&gt;
  &lt;math xmlns="http://www.w3.org/1998/Math/MathML"&gt;
    &lt;apply&gt;
      &lt;divide/&gt;
      &lt;apply&gt;
        &lt;times/&gt;
        &lt;ci&gt; mw702a7f39_5675_48ad_953a_4bcbbdc8e1a2 &lt;/ci&gt;
        &lt;apply&gt;
          &lt;minus/&gt;
          &lt;ci&gt; species_17 &lt;/ci&gt;
          &lt;apply&gt;
            &lt;divide/&gt;
```

<ci> species_20 </ci>

<ci> mw46a0d4e0_a24e_4d84_ac9d_1d098ed93c0b </ci>

</apply>

</apply>

</apply>

<apply>

<plus/>

<ci> species_17 </ci>

<ci> species_20 </ci>

</apply>

</apply>

</math>

</kineticLaw>

</reaction>

<reaction id="reaction_13" name="C_13 (phosphoribulokinase)" reversible="false" fast="false">

<annotation>

<COPASI xmlns="http://www.copasi.org/static/sbml">

<rdf:RDF xmlns:dcterms="http://purl.org/dc/terms/"
xmlns:rdf="http://www.w3.org/1999/02/22-rdf-syntax-ns#">

<rdf:Description rdf:about="#COPASI61">

<dcterms:created>

<rdf:Description>

<dcterms:W3CDTF>2010-04-23T03:48:04Z</dcterms:W3CDTF>

</rdf:Description>

</dcterms:created>

</rdf:Description>

</rdf:RDF>

</COPASI>

```
</annotation>
<listOfReactants>
  <speciesReference species="species_20"/>
  <speciesReference species="species_7"/>
  <speciesReference species="species_12"/>
  <speciesReference species="species_2"/>
</listOfReactants>
<listOfProducts>
  <speciesReference species="species_3"/>
  <speciesReference species="species_4"/>
  <speciesReference species="species_12"/>
  <speciesReference species="species_2"/>
</listOfProducts>
<kineticLaw>
  <math xmlns="http://www.w3.org/1998/Math/MathML">
    <apply>
      <divide/>
      <apply>
        <times/>
        <ci> mwd486ae05_3de1_42aa_9584_2edc458d0c59 </ci>
        <ci> species_7 </ci>
        <ci> species_20 </ci>
      </apply>
      <apply>
        <times/>
        <apply>
          <plus/>
```

```
  <apply>
   <times/>
   <ci> species_7 </ci>
   <apply>
    <plus/>
    <cn type="integer"> 1 </cn>
    <apply>
     <divide/>
     <ci> species_4 </ci>
     <ci> mw16745cb2_2a9e_48f6_8cef_80e44ec67119 </ci>
    </apply>
   </apply>
  </apply>
  <apply>
   <times/>
   <ci> mwc1d6540f_78d6_40ae_8e94_d6db3d674a6e </ci>
   <apply>
    <plus/>
    <cn type="integer"> 1 </cn>
    <apply>
     <divide/>
     <ci> species_4 </ci>
     <ci> mw814665c4_d157_4663_a75e_af6f8f891fb0 </ci>
    </apply>
   </apply>
  </apply>
 </apply>
```

```
<apply>
  <plus/>
  <ci> species_20 </ci>
  <apply>
    <times/>
    <ci> mw9c902b95_94d4_4dff_912f_eb93bd74d440 </ci>
    <apply>
      <plus/>
      <cn type="integer"> 1 </cn>
      <apply>
        <divide/>
        <ci> species_2 </ci>
        <ci> mwd947eee9_9a97_4305_a38f_5d0342490834 </ci>
      </apply>
      <apply>
        <divide/>
        <ci> species_3 </ci>
        <ci> mw0b12ed4b_ffff_47bd_a925_140813e42732 </ci>
      </apply>
      <apply>
        <divide/>
        <ci> species_12 </ci>
        <ci> mw6bb6ce48_f2df_4db7_abe8_95a611a6672b </ci>
      </apply>
    </apply>
  </apply>
</apply>
```

```
          </apply>
        </apply>
      </math>
    </kineticLaw>
  </reaction>
  <reaction id="reaction_14" name="SS_1 (glycogen and sucrose synthesis)" fast="false">
    <annotation>
      <COPASI xmlns="http://www.copasi.org/static/sbml">
        <rdf:RDF xmlns:dcterms="http://purl.org/dc/terms/"
xmlns:rdf="http://www.w3.org/1999/02/22-rdf-syntax-ns#">
          <rdf:Description rdf:about="#COPASI62">
            <dcterms:created>
              <rdf:Description>
                <dcterms:W3CDTF>2010-04-29T13:08:18Z</dcterms:W3CDTF>
              </rdf:Description>
            </dcterms:created>
          </rdf:Description>
        </rdf:RDF>
      </COPASI>
    </annotation>
    <listOfReactants>
      <speciesReference species="species_15"/>
    </listOfReactants>
    <listOfProducts>
      <speciesReference species="species_21"/>
    </listOfProducts>
    <kineticLaw>
      <math xmlns="http://www.w3.org/1998/Math/MathML">
```

```
      <apply>
        <divide/>
        <apply>
          <times/>
          <ci> mw243dc3c0_44b5_4ef5_b8ba_92846b4fee4c </ci>
          <apply>
            <minus/>
            <apply>
              <times/>
              <ci> mwaeeded85_9203_48b6_89a9_9dbf6c0d38da </ci>
              <ci> species_15 </ci>
            </apply>
            <ci> species_21 </ci>
          </apply>
        </apply>
        <apply>
          <plus/>
          <ci> species_15 </ci>
          <ci> species_21 </ci>
        </apply>
      </apply>
    </math>
  </kineticLaw>
</reaction>
<reaction id="reaction_17" name="LIGHT_1 (ATP synthase)" reversible="false" fast="false">
  <annotation>
    <COPASI xmlns="http://www.copasi.org/static/sbml">
```

```
<rdf:RDF xmlns:dcterms="http://purl.org/dc/terms/"
xmlns:rdf="http://www.w3.org/1999/02/22-rdf-syntax-ns#">
  <rdf:Description rdf:about="#COPASI65">
    <dcterms:created>
      <rdf:Description>
        <dcterms:W3CDTF>2010-05-24T02:11:27Z</dcterms:W3CDTF>
      </rdf:Description>
    </dcterms:created>
  </rdf:Description>
</rdf:RDF>
</COPASI>
</annotation>
<listOfReactants>
  <speciesReference species="species_12"/>
  <speciesReference species="species_4"/>
</listOfReactants>
<listOfProducts>
  <speciesReference species="species_7"/>
</listOfProducts>
<kineticLaw>
  <math xmlns="http://www.w3.org/1998/Math/MathML">
    <apply>
      <times/>
      <ci> parameter_1 </ci>
      <ci> species_12 </ci>
      <ci> species_4 </ci>
    </apply>
  </math>
```

```
    </kineticLaw>
   </reaction>
   <reaction id="reaction_18" name="LIGHT_2" reversible="false" fast="false">
    <annotation>
     <COPASI xmlns="http://www.copasi.org/static/sbml">
      <rdf:RDF xmlns:dcterms="http://purl.org/dc/terms/"
xmlns:rdf="http://www.w3.org/1999/02/22-rdf-syntax-ns#">
       <rdf:Description rdf:about="#COPASI66">
        <dcterms:created>
         <rdf:Description>
          <dcterms:W3CDTF>2010-06-09T00:18:40Z</dcterms:W3CDTF>
         </rdf:Description>
        </dcterms:created>
       </rdf:Description>
      </rdf:RDF>
     </COPASI>
    </annotation>
    <listOfReactants>
     <speciesReference species="species_11"/>
    </listOfReactants>
    <listOfProducts>
     <speciesReference species="species_5"/>
    </listOfProducts>
    <kineticLaw>
     <math xmlns="http://www.w3.org/1998/Math/MathML">
      <apply>
       <times/>
       <ci> parameter_2 </ci>
```

```
            <ci> species_11 </ci>
          </apply>
        </math>
      </kineticLaw>
    </reaction>
    <reaction id="reaction_19" name="Sink GAP" reversible="false" fast="false">
      <annotation>
        <COPASI xmlns="http://www.copasi.org/static/sbml">
          <rdf:RDF xmlns:dcterms="http://purl.org/dc/terms/"
xmlns:rdf="http://www.w3.org/1999/02/22-rdf-syntax-ns#">
            <rdf:Description rdf:about="#COPASI67">
              <dcterms:created>
                <rdf:Description>
                  <dcterms:W3CDTF>2011-04-26T00:16:45Z</dcterms:W3CDTF>
                </rdf:Description>
              </dcterms:created>
            </rdf:Description>
          </rdf:RDF>
        </COPASI>
      </annotation>
      <listOfReactants>
        <speciesReference species="species_10"/>
      </listOfReactants>
      <listOfProducts>
        <speciesReference species="mwaf22c073_9b9c_42b1_bd8e_6e65777ddddc"
stoichiometry="3"/>
      </listOfProducts>
      <kineticLaw>
```

```
      <math xmlns="http://www.w3.org/1998/Math/MathML">
        <apply>
          <divide/>
          <apply>
            <times/>
            <ci> mwe110663f_33e9_415c_96f8_04e92e007bb4 </ci>
            <ci> species_10 </ci>
          </apply>
          <apply>
            <plus/>
            <ci> mw395deb93_b80e_4154_b71a_add84953daaf </ci>
            <ci> species_10 </ci>
          </apply>
        </apply>
      </math>
    </kineticLaw>
  </reaction>
  <reaction id="reaction_20" name="Sink E4P" reversible="false" fast="false">
    <annotation>
      <COPASI xmlns="http://www.copasi.org/static/sbml">
        <rdf:RDF xmlns:dcterms="http://purl.org/dc/terms/"
xmlns:rdf="http://www.w3.org/1999/02/22-rdf-syntax-ns#">
          <rdf:Description rdf:about="#COPASI68">
            <dcterms:created>
              <rdf:Description>
                <dcterms:W3CDTF>2011-04-26T00:17:24Z</dcterms:W3CDTF>
              </rdf:Description>
            </dcterms:created>
```

```
          </rdf:Description>
        </rdf:RDF>
      </COPASI>
    </annotation>
    <listOfReactants>
      <speciesReference species="species_16"/>
    </listOfReactants>
    <listOfProducts>
      <speciesReference species="mw09d7964e_16d4_4c3d_a1f5_d98a7ab66891"
stoichiometry="4"/>
    </listOfProducts>
    <kineticLaw>
      <math xmlns="http://www.w3.org/1998/Math/MathML">
        <apply>
          <divide/>
          <apply>
            <times/>
            <ci> mw715a0d4a_6423_4755_a1b5_7d9e5b1852a2 </ci>
            <ci> species_16 </ci>
          </apply>
          <apply>
            <plus/>
            <ci> mw4d3fe418_9692_4308_a1c1_64b6e3b6ad82 </ci>
            <ci> species_16 </ci>
          </apply>
        </apply>
      </math>
    </kineticLaw>
```

</reaction>

<reaction id="reaction_21" name="Sink Ri5P" reversible="false" fast="false">

<annotation>

<COPASI xmlns="http://www.copasi.org/static/sbml">

<rdf:RDF xmlns:dcterms="http://purl.org/dc/terms/" xmlns:rdf="http://www.w3.org/1999/02/22-rdf-syntax-ns#">

<rdf:Description rdf:about="#COPASI69">

<dcterms:created>

<rdf:Description>

<dcterms:W3CDTF>2011-04-26T00:17:46Z</dcterms:W3CDTF>

</rdf:Description>

</dcterms:created>

</rdf:Description>

</rdf:RDF>

</COPASI>

</annotation>

<listOfReactants>

<speciesReference species="species_18"/>

</listOfReactants>

<listOfProducts>

<speciesReference species="mw70a9d4b8_2ca3_4692_a159_702d8464b7e7" stoichiometry="5"/>

</listOfProducts>

<kineticLaw>

<math xmlns="http://www.w3.org/1998/Math/MathML">

<apply>

<divide/>

<apply>

```
              <times/>
              <ci> mw6caa1525_e69b_4ab2_b8c7_2b7deeb5ee58 </ci>
              <ci> species_18 </ci>
            </apply>
            <apply>
              <plus/>
              <ci> mwe871df2e_a7b7_49c5_8a95_809524380cbd </ci>
              <ci> species_18 </ci>
            </apply>
          </apply>
        </math>
      </kineticLaw>
    </reaction>
    <reaction id="reaction_22" name="PP_2a (phosphoglycolate phosphatase)" reversible="false"
fast="false">
      <annotation>
        <COPASI xmlns="http://www.copasi.org/static/sbml">
          <rdf:RDF xmlns:dcterms="http://purl.org/dc/terms/"
xmlns:rdf="http://www.w3.org/1999/02/22-rdf-syntax-ns#">
            <rdf:Description rdf:about="#COPASI70">
              <dcterms:created>
                <rdf:Description>
                  <dcterms:W3CDTF>2011-05-09T23:56:05Z</dcterms:W3CDTF>
                </rdf:Description>
              </dcterms:created>
            </rdf:Description>
          </rdf:RDF>
        </COPASI>
```

```
</annotation>
<listOfReactants>
  <speciesReference species="species_25"/>
</listOfReactants>
<listOfProducts>
  <speciesReference species="species_26"/>
  <speciesReference species="species_12"/>
</listOfProducts>
<kineticLaw>
  <math xmlns="http://www.w3.org/1998/Math/MathML">
    <apply>
      <divide/>
      <apply>
        <times/>
        <ci> mw3c5b8b60_a85d_4b23_8f30_04a8715af44c </ci>
        <ci> species_25 </ci>
      </apply>
      <apply>
        <plus/>
        <ci> species_25 </ci>
        <apply>
          <times/>
          <ci> mw04118d88_412e_49b2_8ed5_aba0f23d1258 </ci>
          <apply>
            <plus/>
            <cn type="integer"> 1 </cn>
            <apply>
```

```
              <divide/>
              <ci> species_26 </ci>
              <ci> KI1121 </ci>
            </apply>
          </apply>
          <apply>
            <plus/>
            <cn type="integer"> 1 </cn>
            <apply>
              <divide/>
              <ci> species_12 </ci>
              <ci> KI1122 </ci>
            </apply>
          </apply>
        </apply>
      </apply>
    </apply>
  </math>
  <listOfParameters>
    <parameter id="KI1121" name="KI1121" value="94"/>
    <parameter id="KI1122" name="KI1122" value="2.55"/>
  </listOfParameters>
</kineticLaw>
</reaction>
<reaction id="reaction_23" name="PP_3 (glycolate oxidase)" reversible="false" fast="false">
  <annotation>
    <COPASI xmlns="http://www.copasi.org/static/sbml">
```

```
<rdf:RDF xmlns:dcterms="http://purl.org/dc/terms/"
xmlns:rdf="http://www.w3.org/1999/02/22-rdf-syntax-ns#">
  <rdf:Description rdf:about="#COPASI71">
    <dcterms:created>
      <rdf:Description>
        <dcterms:W3CDTF>2011-05-10T00:12:50Z</dcterms:W3CDTF>
      </rdf:Description>
    </dcterms:created>
  </rdf:Description>
</rdf:RDF>
</COPASI>
</annotation>
<listOfReactants>
  <speciesReference species="species_26"/>
</listOfReactants>
<listOfProducts>
  <speciesReference species="species_27"/>
</listOfProducts>
<kineticLaw>
  <math xmlns="http://www.w3.org/1998/Math/MathML">
    <apply>
      <divide/>
      <apply>
        <times/>
        <ci> mw5f743344_e84b_4721_8bd4_3643964b38ea </ci>
        <ci> species_26 </ci>
      </apply>
      <apply>
```

```
            <plus/>
            <ci> species_26 </ci>
            <ci> Km121 </ci>
          </apply>
        </apply>
      </math>
      <listOfParameters>
        <parameter id="Km121" name="Km121" value="0.1"/>
      </listOfParameters>
    </kineticLaw>
  </reaction>
  <reaction id="reaction_24" name="PP_4 (serineglyoxylate transaminase)" fast="false">
    <annotation>
      <COPASI xmlns="http://www.copasi.org/static/sbml">
        <rdf:RDF xmlns:dcterms="http://purl.org/dc/terms/"
xmlns:rdf="http://www.w3.org/1999/02/22-rdf-syntax-ns#">
          <rdf:Description rdf:about="#COPASI72">
            <dcterms:created>
              <rdf:Description>
                <dcterms:W3CDTF>2011-05-10T00:16:51Z</dcterms:W3CDTF>
              </rdf:Description>
            </dcterms:created>
          </rdf:Description>
        </rdf:RDF>
      </COPASI>
    </annotation>
    <listOfReactants>
      <speciesReference species="species_27"/>
```

```
  <speciesReference species="species_29"/>
</listOfReactants>
<listOfProducts>
  <speciesReference species="species_30"/>
  <speciesReference species="species_28"/>
</listOfProducts>
<kineticLaw>
  <math xmlns="http://www.w3.org/1998/Math/MathML">
    <apply>
      <divide/>
      <apply>
        <times/>
        <ci> mw43b7b905_45eb_4193_9d33_c0623544faba </ci>
        <apply>
          <minus/>
          <apply>
            <times/>
            <ci> species_27 </ci>
            <ci> species_29 </ci>
          </apply>
          <apply>
            <divide/>
            <apply>
              <times/>
              <ci> species_30 </ci>
              <ci> species_28 </ci>
            </apply>
```

```
      <ci> KE124 </ci>
    </apply>
  </apply>
</apply>
<apply>
  <times/>
  <apply>
    <plus/>
    <ci> species_27 </ci>
    <ci> Km1241 </ci>
  </apply>
  <apply>
    <plus/>
    <ci> species_29 </ci>
    <apply>
      <times/>
      <ci> Km1242 </ci>
      <apply>
        <plus/>
        <cn type="integer"> 1 </cn>
        <apply>
          <divide/>
          <ci> species_28 </ci>
          <ci> KI124 </ci>
        </apply>
      </apply>
    </apply>
```

```xml
            </apply>
          </apply>
        </apply>
      </math>
      <listOfParameters>
        <parameter id="KE124" name="KE124" value="607"/>
        <parameter id="Km1241" name="Km1241" value="0.15"/>
        <parameter id="Km1242" name="Km1242" value="1.7"/>
        <parameter id="KI124" name="KI124" value="2"/>
      </listOfParameters>
    </kineticLaw>
  </reaction>
  <reaction id="reaction_25" name="PP_5 (serine hydroxymethyltransferase)" reversible="false"
fast="false">
    <annotation>
      <COPASI xmlns="http://www.copasi.org/static/sbml">
        <rdf:RDF xmlns:dcterms="http://purl.org/dc/terms/"
xmlns:rdf="http://www.w3.org/1999/02/22-rdf-syntax-ns#">
          <rdf:Description rdf:about="#COPASI73">
            <dcterms:created>
              <rdf:Description>
                <dcterms:W3CDTF>2011-05-10T00:22:17Z</dcterms:W3CDTF>
              </rdf:Description>
            </dcterms:created>
          </rdf:Description>
        </rdf:RDF>
      </COPASI>
    </annotation>
```

```
  <listOfReactants>
    <speciesReference species="species_28" stoichiometry="2"/>
  </listOfReactants>
  <listOfProducts>
    <speciesReference species="species_29"/>
  </listOfProducts>
  <kineticLaw>
    <math xmlns="http://www.w3.org/1998/Math/MathML">
      <apply>
        <divide/>
        <apply>
          <times/>
          <ci> mw21878232_3329_413d_9ad8_7dd1cfc2be9c </ci>
          <ci> species_28 </ci>
        </apply>
        <apply>
          <plus/>
          <ci> mwK13dcce1c1_26b6_4b0e_8782_547ab513fb70 </ci>
          <ci> species_28 </ci>
        </apply>
      </apply>
    </math>
    <listOfParameters>
      <parameter id="mwK13dcce1c1_26b6_4b0e_8782_547ab513fb70" name="K1" value="6"/>
    </listOfParameters>
  </kineticLaw>
</reaction>
```

```
      <reaction id="reaction_26" name="PP_6 (hydroxypyruvate reductase)" reversible="false"
fast="false">
        &lt;annotation&gt;
          &lt;COPASI xmlns="http://www.copasi.org/static/sbml"&gt;
            <rdf:RDF xmlns:dcterms="http://purl.org/dc/terms/"
xmlns:rdf="http://www.w3.org/1999/02/22-rdf-syntax-ns#">
              &lt;rdf:Description rdf:about="#COPASI74"&gt;
                &lt;dcterms:created&gt;
                  &lt;rdf:Description&gt;
                    &lt;dcterms:W3CDTF&gt;2011-05-10T01:04:31Z&lt;/dcterms:W3CDTF&gt;
                  &lt;/rdf:Description&gt;
                &lt;/dcterms:created&gt;
              &lt;/rdf:Description&gt;
            &lt;/rdf:RDF&gt;
          &lt;/COPASI&gt;
        &lt;/annotation&gt;
        &lt;listOfReactants&gt;
          &lt;speciesReference species="species_30"/&gt;
        &lt;/listOfReactants&gt;
        &lt;listOfProducts&gt;
          &lt;speciesReference species="species_31"/&gt;
        &lt;/listOfProducts&gt;
        &lt;kineticLaw&gt;
          &lt;math xmlns="http://www.w3.org/1998/Math/MathML"&gt;
            &lt;apply&gt;
              &lt;divide/&gt;
              &lt;apply&gt;
                &lt;times/&gt;
```

```
<ci> mw9331ee05_e8ae_4da7_be3c_2ae749a0c97a </ci>
<apply>
  <minus/>
  <ci> species_30 </ci>
  <apply>
    <divide/>
    <ci> species_31 </ci>
    <ci> KE123 </ci>
  </apply>
</apply>
</apply>
<apply>
  <plus/>
  <ci> species_30 </ci>
  <apply>
    <times/>
    <ci> Km1231 </ci>
    <apply>
      <plus/>
      <cn type="integer"> 1 </cn>
      <apply>
        <divide/>
        <ci> species_30 </ci>
        <ci> KI123 </ci>
      </apply>
    </apply>
  </apply>
```

```
        </apply>
       </apply>
      </math>
      <listOfParameters>
       <parameter id="KE123" name="KE123" value="250000"/>
       <parameter id="Km1231" name="Km1231" value="0.09"/>
       <parameter id="KI123" name="KI123" value="12"/>
      </listOfParameters>
     </kineticLaw>
    </reaction>
    <reaction id="reaction_27" name="PP_7 (glycerate kinase)" reversible="false" fast="false">
     <annotation>
      <COPASI xmlns="http://www.copasi.org/static/sbml">
       <rdf:RDF xmlns:dcterms="http://purl.org/dc/terms/"
xmlns:rdf="http://www.w3.org/1999/02/22-rdf-syntax-ns#">
        <rdf:Description rdf:about="#COPASI75">
         <dcterms:created>
          <rdf:Description>
           <dcterms:W3CDTF>2011-05-10T01:09:19Z</dcterms:W3CDTF>
          </rdf:Description>
         </dcterms:created>
        </rdf:Description>
       </rdf:RDF>
      </COPASI>
     </annotation>
     <listOfReactants>
      <speciesReference species="species_31"/>
      <speciesReference species="species_7"/>
```

```
</listOfReactants>
<listOfProducts>
  <speciesReference species="species_2"/>
  <speciesReference species="species_4"/>
</listOfProducts>
<kineticLaw>
  <math xmlns="http://www.w3.org/1998/Math/MathML">
    <apply>
      <divide/>
      <apply>
        <times/>
        <ci> mw4a170042_d461_4f96_b16d_2969db08423c </ci>
        <apply>
          <minus/>
          <apply>
            <times/>
            <ci> species_7 </ci>
            <ci> species_31 </ci>
          </apply>
          <apply>
            <divide/>
            <apply>
              <times/>
              <ci> species_4 </ci>
              <ci> species_2 </ci>
            </apply>
            <ci> KE113 </ci>
```

```
    </apply>
  </apply>
</apply>
<apply>
  <times/>
  <apply>
    <plus/>
    <ci> species_7 </ci>
    <apply>
      <times/>
      <ci> Km1131 </ci>
      <apply>
        <plus/>
        <cn type="integer"> 1 </cn>
        <apply>
          <divide/>
          <ci> species_2 </ci>
          <ci> KI113 </ci>
        </apply>
      </apply>
    </apply>
  </apply>
  <apply>
    <plus/>
    <ci> species_31 </ci>
    <ci> Km1132 </ci>
  </apply>
```

```
          </apply>
        </apply>
      </math>
      <listOfParameters>
        <parameter id="KE113" name="KE113" value="300"/>
        <parameter id="Km1131" name="Km1131" value="0.21"/>
        <parameter id="KI113" name="KI113" value="0.36"/>
        <parameter id="Km1132" name="Km1132" value="0.25"/>
      </listOfParameters>
    </kineticLaw>
  </reaction>
  <reaction id="reaction_30" name="GL_4 (enolase)" fast="false">
    <annotation>
      <COPASI xmlns="http://www.copasi.org/static/sbml">
        <rdf:RDF xmlns:dcterms="http://purl.org/dc/terms/"
xmlns:rdf="http://www.w3.org/1999/02/22-rdf-syntax-ns#">
          <rdf:Description rdf:about="#COPASI78">
            <dcterms:created>
              <rdf:Description>
                <dcterms:W3CDTF>2011-05-10T15:12:27Z</dcterms:W3CDTF>
              </rdf:Description>
            </dcterms:created>
          </rdf:Description>
        </rdf:RDF>
      </COPASI>
    </annotation>
    <listOfReactants>
      <speciesReference species="species_35"/>
```

```
</listOfReactants>
<listOfProducts>
  <speciesReference species="species_32"/>
</listOfProducts>
<kineticLaw>
  <math xmlns="http://www.w3.org/1998/Math/MathML">
    <apply>
      <divide/>
      <apply>
        <times/>
        <ci> mw68a26bf2_520b_4b35_90e0_511ec4bbc859 </ci>
        <apply>
          <minus/>
          <ci> species_35 </ci>
          <apply>
            <divide/>
            <ci> species_32 </ci>
            <ci> mwdacf4f8b_f1db_480a_aabf_ff89dc90e087 </ci>
          </apply>
        </apply>
      </apply>
      <apply>
        <times/>
        <ci> mwe806711a_3cbc_4d66_a530_d7ae4e673eea </ci>
        <apply>
          <plus/>
          <cn type="integer"> 1 </cn>
```

```
              <apply>
                <divide/>
                <ci> species_35 </ci>
                <ci> mwe806711a_3cbc_4d66_a530_d7ae4e673eea </ci>
              </apply>
              <apply>
                <divide/>
                <ci> species_32 </ci>
                <ci> mwdee0f9ac_28b4_444f_af80_30c299f78093 </ci>
              </apply>
            </apply>
          </apply>
        </apply>
      </math>
    </kineticLaw>
  </reaction>
  <reaction id="reaction_31" name="Sink PEP" reversible="false" fast="false">
    <annotation>
      <COPASI xmlns="http://www.copasi.org/static/sbml">
        <rdf:RDF xmlns:dcterms="http://purl.org/dc/terms/"
xmlns:rdf="http://www.w3.org/1999/02/22-rdf-syntax-ns#">
          <rdf:Description rdf:about="#COPASI79">
            <dcterms:created>
              <rdf:Description>
                <dcterms:W3CDTF>2011-05-10T16:28:58Z</dcterms:W3CDTF>
              </rdf:Description>
            </dcterms:created>
          </rdf:Description>
```

```
        &lt;/rdf:RDF&gt;
      &lt;/COPASI&gt;
    &lt;/annotation&gt;
    &lt;listOfReactants&gt;
      &lt;speciesReference species="species_32"/&gt;
    &lt;/listOfReactants&gt;
    &lt;listOfProducts&gt;
      <speciesReference species="mwa8568197_97f2_4c67_8727_f8d9ed6895e3"
stoichiometry="3"/>
    &lt;/listOfProducts&gt;
    &lt;kineticLaw&gt;
      &lt;math xmlns="http://www.w3.org/1998/Math/MathML"&gt;
        &lt;apply&gt;
          &lt;divide/&gt;
          &lt;apply&gt;
            &lt;times/&gt;
            &lt;ci&gt; mw5723da7e_7ee9_4b45_b397_c4fad0251fa2 &lt;/ci&gt;
            &lt;ci&gt; species_32 &lt;/ci&gt;
          &lt;/apply&gt;
          &lt;apply&gt;
            &lt;plus/&gt;
            &lt;ci&gt; mw70b2b9c1_0904_4851_aee4_8526d26a4c7f &lt;/ci&gt;
            &lt;ci&gt; species_32 &lt;/ci&gt;
          &lt;/apply&gt;
        &lt;/apply&gt;
      &lt;/math&gt;
    &lt;/kineticLaw&gt;
  &lt;/reaction&gt;
```

```
<reaction id="reaction_33" name="GSM_1 (simplified phosphoserine transaminase)"
reversible="false" fast="false">
  <annotation>
    <COPASI xmlns="http://www.copasi.org/static/sbml">
      <rdf:RDF xmlns:dcterms="http://purl.org/dc/terms/"
xmlns:rdf="http://www.w3.org/1999/02/22-rdf-syntax-ns#">
        <rdf:Description rdf:about="#COPASI81">
          <dcterms:created>
            <rdf:Description>
              <dcterms:W3CDTF>2011-06-15T16:11:22Z</dcterms:W3CDTF>
            </rdf:Description>
          </dcterms:created>
        </rdf:Description>
      </rdf:RDF>
    </COPASI>
  </annotation>
  <listOfReactants>
    <speciesReference species="species_2"/>
  </listOfReactants>
  <listOfProducts>
    <speciesReference species="species_29"/>
  </listOfProducts>
  <kineticLaw>
    <math xmlns="http://www.w3.org/1998/Math/MathML">
      <apply>
        <divide/>
        <apply>
          <times/>
```

```
            <ci> mwa5897908_6cba_4e9b_ae16_669f6cc2215d </ci>
            <ci> species_2 </ci>
          </apply>
          <apply>
            <plus/>
            <ci> mwK17d0edf01_29a7_4ee8_8bbf_a6dd0a6e695e </ci>
            <ci> species_2 </ci>
          </apply>
        </apply>
      </math>
      <listOfParameters>
        <parameter id="mwK17d0edf01_29a7_4ee8_8bbf_a6dd0a6e695e" name="K_synth_SER"
value="2"/>
      </listOfParameters>
    </kineticLaw>
  </reaction>
  <reaction id="reaction_34" name="GC_1 (tartronate semialdehyde synthase)" reversible="false"
fast="false">
    <annotation>
      <COPASI xmlns="http://www.copasi.org/static/sbml">
        <rdf:RDF xmlns:dcterms="http://purl.org/dc/terms/"
xmlns:rdf="http://www.w3.org/1999/02/22-rdf-syntax-ns#">
          <rdf:Description rdf:about="#COPASI82">
            <dcterms:created>
              <rdf:Description>
                <dcterms:W3CDTF>2011-06-15T16:39:15Z</dcterms:W3CDTF>
              </rdf:Description>
            </dcterms:created>
          </rdf:Description>
```

```
          </rdf:RDF>
        </COPASI>
      </annotation>
      <listOfReactants>
        <speciesReference species="species_27" stoichiometry="2"/>
      </listOfReactants>
      <listOfProducts>
        <speciesReference species="species_36"/>
      </listOfProducts>
      <kineticLaw>
        <math xmlns="http://www.w3.org/1998/Math/MathML">
          <apply>
            <divide/>
            <apply>
              <times/>
              <ci> mwf7e8c4cc_5866_4d9d_80a4_fb1d0db24ecf </ci>
              <ci> species_27 </ci>
            </apply>
            <apply>
              <plus/>
              <ci> mwK1a89ce5e5_bfda_43b5_bb20_0b16e57e3632 </ci>
              <ci> species_27 </ci>
            </apply>
          </apply>
        </math>
        <listOfParameters>
          <parameter id="mwK1a89ce5e5_bfda_43b5_bb20_0b16e57e3632" name="K_TSA1"
value="0.1"/>
```

```
        </listOfParameters>
      </kineticLaw>
    </reaction>
    <reaction id="reaction_35" name="Sink TSA" reversible="false" fast="false">
      <annotation>
        <COPASI xmlns="http://www.copasi.org/static/sbml">
          <rdf:RDF xmlns:dcterms="http://purl.org/dc/terms/"
xmlns:rdf="http://www.w3.org/1999/02/22-rdf-syntax-ns#">
            <rdf:Description rdf:about="#COPASI83">
              <dcterms:created>
                <rdf:Description>
                  <dcterms:W3CDTF>2011-06-15T16:40:30Z</dcterms:W3CDTF>
                </rdf:Description>
              </dcterms:created>
            </rdf:Description>
          </rdf:RDF>
        </COPASI>
      </annotation>
      <listOfReactants>
        <speciesReference species="species_36"/>
      </listOfReactants>
      <listOfProducts>
        <speciesReference species="species_33"/>
      </listOfProducts>
      <kineticLaw>
        <math xmlns="http://www.w3.org/1998/Math/MathML">
          <apply>
            <divide/>
```

```xml
<apply>
<times/>
<ci> mw0f93be3f_8f7b_48cf_b994_7d7d51c0c99d </ci>
<ci> species_36 </ci>
</apply>
<apply>
<plus/>
<ci> mwK125774f66_2af6_440c_a4df_5d10c0a54ba0 </ci>
<ci> species_36 </ci>
</apply>
</apply>
</math>
<listOfParameters>
<parameter id="mwK125774f66_2af6_440c_a4df_5d10c0a54ba0" name="K_TSA2" value="0.4"/>
</listOfParameters>
</kineticLaw>
</reaction>
<reaction id="reaction_36" name="GC_2 (tartronate semialdehyde reductase)" reversible="false" fast="false">
<annotation>
<COPASI xmlns="http://www.copasi.org/static/sbml">
<rdf:RDF xmlns:dcterms="http://purl.org/dc/terms/" xmlns:rdf="http://www.w3.org/1999/02/22-rdf-syntax-ns#">
<rdf:Description rdf:about="#COPASI84">
<dcterms:created>
<rdf:Description>
<dcterms:W3CDTF>2011-06-15T16:40:54Z</dcterms:W3CDTF>
</rdf:Description>
```

```
      </dcterms:created>
    </rdf:Description>
  </rdf:RDF>
 </COPASI>
</annotation>
<listOfReactants>
 <speciesReference species="species_36"/>
</listOfReactants>
<listOfProducts>
 <speciesReference species="species_31"/>
</listOfProducts>
<kineticLaw>
 <math xmlns="http://www.w3.org/1998/Math/MathML">
   <apply>
    <divide/>
    <apply>
     <times/>
     <ci> mw4d57904b_347d_4323_a435_aef6db506bb9 </ci>
     <ci> species_36 </ci>
    </apply>
    <apply>
     <plus/>
     <ci> mwK1e14acb7a_4bcf_44cb_aee8_8a181e61c843 </ci>
     <ci> species_36 </ci>
    </apply>
   </apply>
 </math>
```

```xml
<listOfParameters>
<parameter id="mwK1e14acb7a_4bcf_44cb_aee8_8a181e61c843" name="K_TSA3"
value="0.1"/>
</listOfParameters>
</kineticLaw>
</reaction>
<reaction id="reaction_37" name="OX_1 (glyoxylate oxidase)" reversible="false" fast="false">
<annotation>
<COPASI xmlns="http://www.copasi.org/static/sbml">
<rdf:RDF xmlns:dcterms="http://purl.org/dc/terms/"
xmlns:rdf="http://www.w3.org/1999/02/22-rdf-syntax-ns#">
<rdf:Description rdf:about="#COPASI85">
<dcterms:created>
<rdf:Description>
<dcterms:W3CDTF>2011-06-28T01:12:01Z</dcterms:W3CDTF>
</rdf:Description>
</dcterms:created>
</rdf:Description>
</rdf:RDF>
</COPASI>
</annotation>
<listOfReactants>
<speciesReference species="species_27"/>
</listOfReactants>
<listOfProducts>
<speciesReference species="species_37"/>
</listOfProducts>
<kineticLaw>
```

<math xmlns="http://www.w3.org/1998/Math/MathML">
<apply>
<divide/>
<apply>
<times/>
<ci> mw7f7c8a85_afe2_49aa_8686_1b3950810d23 </ci>
<ci> species_27 </ci>
</apply>
<apply>
<plus/>
<ci> mw6f022b45_ad82_4bfc_bbbd_0855ddf10ecf </ci>
<ci> species_27 </ci>
</apply>
</apply>
</math>
</kineticLaw>
</reaction>
<reaction id="reaction_38" name="Sink OXA" reversible="false" fast="false">
<annotation>
<COPASI xmlns="http://www.copasi.org/static/sbml">
<rdf:RDF xmlns:dcterms="http://purl.org/dc/terms/"
xmlns:rdf="http://www.w3.org/1999/02/22-rdf-syntax-ns#">
<rdf:Description rdf:about="#COPASI86">
<dcterms:created>
<rdf:Description>
<dcterms:W3CDTF>2011-06-28T01:12:33Z</dcterms:W3CDTF>
</rdf:Description>
</dcterms:created>

```
      </rdf:Description>
    </rdf:RDF>
  </COPASI>
</annotation>
<listOfReactants>
  <speciesReference species="species_37"/>
</listOfReactants>
<listOfProducts>
  <speciesReference species="species_34"/>
</listOfProducts>
<kineticLaw>
  <math xmlns="http://www.w3.org/1998/Math/MathML">
    <apply>
      <divide/>
      <apply>
        <times/>
        <ci> mwa562da41_e9ca_4d43_85ba_4372aa40bed8 </ci>
        <ci> species_37 </ci>
      </apply>
      <apply>
        <plus/>
        <ci> mwcd39d210_482b_47b5_b954_07cc17e31155 </ci>
        <ci> species_37 </ci>
      </apply>
    </apply>
  </math>
</kineticLaw>
```

```xml
      </reaction>
      <reaction id="mw33f9b969_7c4e_45ca_9a58_1283f92cad68" name="PP_1 (RuBisCO)" reversible="false" fast="false">
        <listOfReactants>
          <speciesReference species="species_3"/>
          <speciesReference species="species_1"/>
          <speciesReference species="species_6"/>
          <speciesReference species="species_2"/>
          <speciesReference species="species_14"/>
          <speciesReference species="species_8"/>
          <speciesReference species="species_12"/>
          <speciesReference species="species_5"/>
        </listOfReactants>
        <listOfProducts>
          <speciesReference species="species_25"/>
          <speciesReference species="species_2"/>
          <speciesReference species="species_6"/>
          <speciesReference species="species_14"/>
          <speciesReference species="species_8"/>
          <speciesReference species="species_12"/>
          <speciesReference species="species_5"/>
        </listOfProducts>
        <kineticLaw>
          <math xmlns="http://www.w3.org/1998/Math/MathML">
            <apply>
              <divide/>
              <apply>
                <times/>
```

```
 <ci> species_3 </ci>
 <apply>
  <divide/>
  <apply>
   <times/>
   <ci> mw9f919ddc_7006_491f_b827_5b5094d66dd0 </ci>
   <ci> species_1 </ci>
  </apply>
  <apply>
   <plus/>
   <ci> species_1 </ci>
   <apply>
    <times/>
    <ci> mwd5269cf8_ccf6_4ec1_8ba7_8e8de3f91fd3 </ci>
    <apply>
     <plus/>
     <cn type="integer"> 1 </cn>
     <apply>
      <divide/>
      <ci> species_6 </ci>
      <ci> mw58e11717_8712_4ffa_b588_c717796bdd73 </ci>
     </apply>
    </apply>
   </apply>
  </apply>
 </apply>
</apply>
```

```
<apply>
  <plus/>
  <ci> species_3 </ci>
  <apply>
    <times/>
    <ci> mwe084ba25_0a71_42ea_8ebb_20a6485bc610 </ci>
    <apply>
      <plus/>
      <cn type="integer"> 1 </cn>
      <apply>
        <divide/>
        <ci> species_2 </ci>
        <ci> mwb7f4d7b4_05fc_4d66_9774_48dd6b62def8 </ci>
      </apply>
      <apply>
        <divide/>
        <ci> species_14 </ci>
        <ci> mwbfaa378d_c0c2_49ba_960c_cb089dfe679a </ci>
      </apply>
      <apply>
        <divide/>
        <ci> species_8 </ci>
        <ci> mw7ce6353b_b8ee_45d4_b210_882da23dc1a6 </ci>
      </apply>
      <apply>
        <divide/>
        <ci> species_12 </ci>
```

```
<ci> mw7c22b6fb_091f_473c_8ff6_62f0470aa853 </ci>
</apply>
<apply>
<divide/>
<ci> species_5 </ci>
<ci> mw6320b291_cd55_41c4_a212_0f802e1bd889 </ci>
</apply>
</apply>
</apply>
</apply>
</apply>
</math>
<listOfParameters>
<parameter id="mwbfaa378d_c0c2_49ba_960c_cb089dfe679a" name="KI12" value="0.08"/>
<parameter id="mwb7f4d7b4_05fc_4d66_9774_48dd6b62def8" name="KI11" value="0.84"/>
<parameter id="mw7c22b6fb_091f_473c_8ff6_62f0470aa853" name="KI14" value="0.9"/>
<parameter id="mw7ce6353b_b8ee_45d4_b210_882da23dc1a6" name="KI13"
value="0.075"/>
<parameter id="mw6320b291_cd55_41c4_a212_0f802e1bd889" name="KI15" value="0.07"/>
<parameter id="mwd5269cf8_ccf6_4ec1_8ba7_8e8de3f91fd3" name="KM11"
value="0.0115"/>
<parameter id="mw58e11717_8712_4ffa_b588_c717796bdd73" name="KM12"
value="0.222"/>
<parameter id="mwe084ba25_0a71_42ea_8ebb_20a6485bc610" name="KM13"
value="0.02"/>
</listOfParameters>
</kineticLaw>
</reaction>
```

```
<reaction id="mwfb52f808_34ac_44be_8317_544bfa615dbc" name="GL_1 alpha (phosphofructokinase)" reversible="false" fast="false">
  <listOfReactants>
    <speciesReference species="species_15"/>
  </listOfReactants>
  <listOfProducts>
    <speciesReference species="species_14"/>
  </listOfProducts>
  <kineticLaw>
    <math xmlns="http://www.w3.org/1998/Math/MathML">
      <apply>
        <divide/>
        <apply>
          <times/>
          <ci> mwfb9d0842_5ad1_42a2_b44c_12cfeaa36962 </ci>
          <ci> species_15 </ci>
        </apply>
        <apply>
          <plus/>
          <ci> species_15 </ci>
          <ci> mwc59d3254_509c_411a_b656_9e80ac0a4d4a </ci>
        </apply>
      </apply>
    </math>
  </kineticLaw>
</reaction>
<reaction id="mw888f46db_cf06_4f12_ad55_d96247e91c02" name="PP_2b (phosphoglycolate phosphatase)" reversible="false" fast="false">
```

```
<listOfReactants>
  <speciesReference species="species_25"/>
</listOfReactants>
<listOfProducts>
  <speciesReference species="species_26"/>
  <speciesReference species="species_12"/>
</listOfProducts>
<kineticLaw>
  <math xmlns="http://www.w3.org/1998/Math/MathML">
    <apply>
      <divide/>
      <apply>
        <times/>
        <ci> mwa378ec6c_6ace_46c3_a515_4be6d27ac144 </ci>
        <ci> species_25 </ci>
      </apply>
      <apply>
        <plus/>
        <ci> species_25 </ci>
        <apply>
          <times/>
          <ci> mw701ca18f_a968_4128_8415_d39acae3e377 </ci>
          <apply>
            <plus/>
            <cn type="integer"> 1 </cn>
            <apply>
              <divide/>
```

```
                <ci> species_26 </ci>
                <ci> mw6cacd0a2_9c0d_4e6c_ac36_efd63ce3bf57 </ci>
              </apply>
            </apply>
            <apply>
              <plus/>
              <cn type="integer"> 1 </cn>
              <apply>
                <divide/>
                <ci> species_12 </ci>
                <ci> mw4ebf27da_6677_470e_a6ec_3d1e15922742 </ci>
              </apply>
            </apply>
          </apply>
        </apply>
      </apply>
    </math>
    <listOfParameters>
      <parameter id="mw6cacd0a2_9c0d_4e6c_ac36_efd63ce3bf57" name="KI1121" value="94"/>
      <parameter id="mw4ebf27da_6677_470e_a6ec_3d1e15922742" name="KI1122"
value="2.55"/>
    </listOfParameters>
  </kineticLaw>
</reaction>
<reaction id="mw8c050f3c_c600_4cba_8a38_619387f62796" name="GL_3a (phosphoglycerate
mutase)" fast="false">
  <listOfReactants>
    <speciesReference species="species_2"/>
```

```
</listOfReactants>
<listOfProducts>
  <speciesReference species="species_35"/>
</listOfProducts>
<kineticLaw>
  <math xmlns="http://www.w3.org/1998/Math/MathML">
    <apply>
      <divide/>
      <apply>
        <times/>
        <ci> mw4f571ae6_7040_40c6_b098_4ac7cf9ee24d </ci>
        <apply>
          <minus/>
          <ci> species_2 </ci>
          <apply>
            <divide/>
            <ci> species_35 </ci>
            <ci> mw917c32b0_7a3f_4744_ac14_2e3d15da96f6 </ci>
          </apply>
        </apply>
      </apply>
      <apply>
        <times/>
        <ci> mw742fbec5_509e_42be_b7a8_12b9eeb49314 </ci>
        <apply>
          <plus/>
          <cn type="integer"> 1 </cn>
```

```
            <apply>
              <divide/>
              <ci> species_2 </ci>
              <ci> mw742fbec5_509e_42be_b7a8_12b9eeb49314 </ci>
            </apply>
            <apply>
              <divide/>
              <ci> species_35 </ci>
              <ci> mw231dd6ca_4db0_4cdb_adc8_72349abdba07 </ci>
            </apply>
          </apply>
        </apply>
      </apply>
    </math>
  </kineticLaw>
</reaction>
<reaction id="mw13e13d10_557f_4f5e_9340_62df61395963" name="Sink GLY"
reversible="false" fast="false">
  <listOfReactants>
    <speciesReference species="species_28"/>
  </listOfReactants>
  <listOfProducts>
    <speciesReference species="species_24"/>
  </listOfProducts>
  <kineticLaw>
    <math xmlns="http://www.w3.org/1998/Math/MathML">
      <apply>
        <divide/>
```

```
          <apply>
            <times/>
            <ci> mw9c6e9e64_1ad3_462a_b257_61d666a10a15 </ci>
            <ci> species_28 </ci>
          </apply>
          <apply>
            <plus/>
            <ci> mw64f1bfc2_b040_4495_98d5_84e5a2e9b1c0 </ci>
            <ci> species_28 </ci>
          </apply>
        </apply>
      </math>
      <listOfParameters>
        <parameter id="mw64f1bfc2_b040_4495_98d5_84e5a2e9b1c0" name="K_Sink_GLY" value="1"/>
      </listOfParameters>
    </kineticLaw>
  </reaction>
  <reaction id="mwc28b2370_81f7_45f3_a71d_18314992330e" name="Sink SER" reversible="false" fast="false">
    <listOfReactants>
      <speciesReference species="species_29"/>
    </listOfReactants>
    <listOfProducts>
      <speciesReference species="species_24"/>
    </listOfProducts>
    <kineticLaw>
      <math xmlns="http://www.w3.org/1998/Math/MathML">
```

<apply>
<divide/>
<apply>
<times/>
<ci> mw108b366f_58cf_4f32_9f78_d3a14dd13026 </ci>
<ci> species_29 </ci>
</apply>
<apply>
<plus/>
<ci> mw7e38db19_97ad_43ac_a0b0_3a88f4184f72 </ci>
<ci> species_29 </ci>
</apply>
</apply>
</math>
<listOfParameters>
<parameter id="mw7e38db19_97ad_43ac_a0b0_3a88f4184f72" name="K_Sink_SER"
value="1"/>
</listOfParameters>
</kineticLaw>
</reaction>
<reaction id="mwe1c72296_a05e_459f_97dc_559333cc5eb6" name="GL_3b (phosphoglycerate mutase)" fast="false">
<listOfReactants>
<speciesReference species="species_2"/>
</listOfReactants>
<listOfProducts>
<speciesReference species="species_35"/>
</listOfProducts>

```
&lt;kineticLaw&gt;
  &lt;math xmlns="http://www.w3.org/1998/Math/MathML"&gt;
    &lt;apply&gt;
      &lt;divide/&gt;
      &lt;apply&gt;
        &lt;times/&gt;
        &lt;ci&gt; mw2ec1703a_729a_4b49_b4dd_4241b0d73907 &lt;/ci&gt;
        &lt;apply&gt;
          &lt;minus/&gt;
          &lt;ci&gt; species_2 &lt;/ci&gt;
          &lt;apply&gt;
            &lt;divide/&gt;
            &lt;ci&gt; species_35 &lt;/ci&gt;
            &lt;ci&gt; mw917c32b0_7a3f_4744_ac14_2e3d15da96f6 &lt;/ci&gt;
          &lt;/apply&gt;
        &lt;/apply&gt;
      &lt;/apply&gt;
      &lt;apply&gt;
        &lt;times/&gt;
        &lt;ci&gt; mwb99ebe97_7d91_406c_8b12_a56b2c939ff4 &lt;/ci&gt;
        &lt;apply&gt;
          &lt;plus/&gt;
          &lt;cn type="integer"&gt; 1 &lt;/cn&gt;
          &lt;apply&gt;
            &lt;divide/&gt;
            &lt;ci&gt; species_2 &lt;/ci&gt;
            &lt;ci&gt; mwb99ebe97_7d91_406c_8b12_a56b2c939ff4 &lt;/ci&gt;
```

</apply>

<apply>

<divide/>

<ci> species_35 </ci>

<ci> mw23fae7dc_7d12_4fb0_98e2_c611e44007e1 </ci>

</apply>

</apply>

</apply>

</apply>

</math>

</kineticLaw>

</reaction>

<reaction id="mw63f09b77_c48f_4b1e_a2a6_7d5f6d48ea38" name="GL_3c (phosphoglycerate mutase)" fast="false">

<listOfReactants>

<speciesReference species="species_2"/>

</listOfReactants>

<listOfProducts>

<speciesReference species="species_35"/>

</listOfProducts>

<kineticLaw>

<math xmlns="http://www.w3.org/1998/Math/MathML">

<apply>

<divide/>

<apply>

<times/>

<ci> mwa1879047_3c26_45c1_8b2a_137433500cde </ci>

<apply>

```
      <minus/>
      <ci> species_2 </ci>
      <apply>
        <divide/>
        <ci> species_35 </ci>
        <ci> mw917c32b0_7a3f_4744_ac14_2e3d15da96f6 </ci>
      </apply>
    </apply>
  </apply>
  <apply>
    <times/>
    <ci> mw6205c3fd_ecf9_4b2b_98f1_dff68729cd03 </ci>
    <apply>
      <plus/>
      <cn type="integer"> 1 </cn>
      <apply>
        <divide/>
        <ci> species_2 </ci>
        <ci> mw6205c3fd_ecf9_4b2b_98f1_dff68729cd03 </ci>
      </apply>
      <apply>
        <divide/>
        <ci> species_35 </ci>
        <ci> mw23f5979c_8188_4521_9629_6469c95e42d6 </ci>
      </apply>
    </apply>
  </apply>
```

```
        </apply>
      </math>
    </kineticLaw>
  </reaction>
  <reaction id="mw153159fa_5578_45d6_8d12_aacee3745c81" name="CO2 import"
reversible="false" fast="false">
    <listOfReactants>
      <speciesReference species="mw733b668e_1dba_46ef_9777_e9331b0b590d"/>
    </listOfReactants>
    <listOfProducts>
      <speciesReference species="species_1"/>
    </listOfProducts>
    <kineticLaw>
      <math xmlns="http://www.w3.org/1998/Math/MathML">
        <apply>
          <times/>
          <ci> mwaffea60c_8361_4bed_be10_814e0c0f7898 </ci>
          <ci> mw733b668e_1dba_46ef_9777_e9331b0b590d </ci>
        </apply>
      </math>
    </kineticLaw>
  </reaction>
  <reaction id="mwc0a63025_cc5a_48e0_8d5e_91b9f8bb5250" name="GL_2 (glyceraldehyde 3-
phosphate dehydrogenase)" fast="false">
    <listOfReactants>
      <speciesReference species="species_10"/>
      <speciesReference species="species_11"/>
    </listOfReactants>
```

```
<listOfProducts>
  <speciesReference species="species_2"/>
  <speciesReference species="species_5"/>
</listOfProducts>
<kineticLaw>
  <math xmlns="http://www.w3.org/1998/Math/MathML">
    <apply>
      <divide/>
      <apply>
        <times/>
        <ci> mwd0f35f82_c657_4a53_9ac4_606e79cebb96 </ci>
        <apply>
          <minus/>
          <apply>
            <times/>
            <ci> species_10 </ci>
            <ci> species_11 </ci>
          </apply>
          <apply>
            <times/>
            <ci> mw1a31f571_e225_4d2d_b672_bb6209080e70 </ci>
            <ci> species_2 </ci>
            <ci> species_5 </ci>
          </apply>
        </apply>
      </apply>
      <apply>
```

<times/>
<apply>
<plus/>
<cn type="integer"> 1 </cn>
<apply>
<divide/>
<ci> species_10 </ci>
<ci> mw2a519375_b634_461c_a4e7_2205e166b3ce </ci>
</apply>
<apply>
<divide/>
<ci> species_2 </ci>
<ci> mw25dcc228_99f4_4d62_badc_00067a7db23a </ci>
</apply>
</apply>
<apply>
<plus/>
<cn type="integer"> 1 </cn>
<apply>
<divide/>
<ci> species_11 </ci>
<ci> mw02659a3e_942b_4113_928b_885d34341771 </ci>
</apply>
<apply>
<divide/>
<ci> species_5 </ci>
<ci> mw31fc107e_644a_475e_aeff_2b016202c816 </ci>

```
              </apply>
            </apply>
          </apply>
        </apply>
      </math>
    </kineticLaw>
  </reaction>
  <reaction id="mwd149e74a_6b61_4bd9_8b74_1d3187a49160" name="GSM_2 (glycine
transaminase)" reversible="false" fast="false">
    <listOfReactants>
      <speciesReference species="species_27"/>
    </listOfReactants>
    <listOfProducts>
      <speciesReference species="species_28"/>
    </listOfProducts>
    <kineticLaw>
      <math xmlns="http://www.w3.org/1998/Math/MathML">
        <apply>
          <divide/>
          <apply>
            <times/>
            <ci> mw016a3190_5878_41d4_8022_96365a5cde42 </ci>
            <ci> species_27 </ci>
          </apply>
          <apply>
            <plus/>
            <ci> mw1862dfa3_5857_4ef7_82ea_209a042715a4 </ci>
            <ci> species_27 </ci>
```

```
        </apply>
      </apply>
    </math>
  </kineticLaw>
</reaction>
<reaction id="mw04b20bf0_57cd_45c3_8316_a103dafa35fe" name="Sink G6P"
reversible="false" fast="false">
  <listOfReactants>
    <speciesReference species="species_21"/>
  </listOfReactants>
  <listOfProducts>
    <speciesReference species="mw8dc354b7_d1b2_4cc9_bbf9_5d423d6013fb"
stoichiometry="6"/>
  </listOfProducts>
  <kineticLaw>
    <math xmlns="http://www.w3.org/1998/Math/MathML">
      <apply>
        <divide/>
        <apply>
          <times/>
          <ci> mw2fcbcb1c_c103_48bb_86ed_9229a27de6ed </ci>
          <ci> species_21 </ci>
        </apply>
        <apply>
          <plus/>
          <ci> mw52505a99_c3c7_4c4c_8a62_b891bfab4dd8 </ci>
          <ci> species_21 </ci>
        </apply>
```

```
        </apply>
       </math>
      </kineticLaw>
     </reaction>
     <reaction id="mw92eee159_bf34_47ce_adc3_21834f561f8e" name="PKET alpha
(phosphoketolase)" reversible="false" fast="false">
      <listOfReactants>
       <speciesReference species="species_15"/>
      </listOfReactants>
      <listOfProducts>
       <speciesReference species="mwfe019cb4_4031_44c9_83f8_9ba5e8de4ed4"/>
       <speciesReference species="species_16"/>
      </listOfProducts>
      <kineticLaw>
       <math xmlns="http://www.w3.org/1998/Math/MathML">
        <apply>
         <divide/>
         <apply>
          <times/>
          <ci> mw72f442a2_e01a_44d3_b8bf_9d223245f350 </ci>
          <ci> species_15 </ci>
         </apply>
         <apply>
          <plus/>
          <ci> species_15 </ci>
          <ci> mwa27b7723_ab20_4324_a886_f5c73588ac4a </ci>
         </apply>
        </apply>
```

```
        </math>
      </kineticLaw>
    </reaction>
    <reaction id="mw0891344d_8739_44c9_a4fb_b36347073110" name="Sink AceP"
reversible="false" fast="false">
      <listOfReactants>
        <speciesReference species="mwfe019cb4_4031_44c9_83f8_9ba5e8de4ed4"/>
      </listOfReactants>
      <listOfProducts>
        <speciesReference species="mwe556cd16_34e2_417a_b945_37061560bfe5"
stoichiometry="2"/>
      </listOfProducts>
      <kineticLaw>
        <math xmlns="http://www.w3.org/1998/Math/MathML">
          <apply>
            <divide/>
            <apply>
              <times/>
              <ci> mwd7c8b802_9d0c_4a81_9c65_1a50be7968bb </ci>
              <ci> mwfe019cb4_4031_44c9_83f8_9ba5e8de4ed4 </ci>
            </apply>
            <apply>
              <plus/>
              <ci> mwc1298b32_b9e2_4ab0_8087_3acdea483cdc </ci>
              <ci> mwfe019cb4_4031_44c9_83f8_9ba5e8de4ed4 </ci>
            </apply>
          </apply>
        </math>
```

```
      &lt;/kineticLaw&gt;
    &lt;/reaction&gt;
    &lt;reaction id="mw26097e16_ab9d_4350_98c3_2e61cdf3ff9f" name="PKET beta (phosphoketolase)" reversible="false" fast="false"&gt;
      &lt;listOfReactants&gt;
        &lt;speciesReference species="species_15"/&gt;
      &lt;/listOfReactants&gt;
      &lt;listOfProducts&gt;
        &lt;speciesReference species="mwfe019cb4_4031_44c9_83f8_9ba5e8de4ed4"/&gt;
        &lt;speciesReference species="species_16"/&gt;
      &lt;/listOfProducts&gt;
      &lt;kineticLaw&gt;
        &lt;math xmlns="http://www.w3.org/1998/Math/MathML"&gt;
          &lt;apply&gt;
            &lt;divide/&gt;
            &lt;apply&gt;
              &lt;times/&gt;
              &lt;ci&gt; mw86416738_ea27_41b8_adf9_4d3b5a320d97 &lt;/ci&gt;
              &lt;ci&gt; species_15 &lt;/ci&gt;
            &lt;/apply&gt;
            &lt;apply&gt;
              &lt;plus/&gt;
              &lt;ci&gt; species_15 &lt;/ci&gt;
              &lt;ci&gt; mw57949b29_e69c_444c_8d58_1ddda96d13c5 &lt;/ci&gt;
            &lt;/apply&gt;
          &lt;/apply&gt;
        &lt;/math&gt;
      &lt;/kineticLaw&gt;
```

```
    </reaction>
    <reaction id="mwcb3cefb7_75fa_446c_9836_44386ea80892" name="GL_1 beta
(phosphofructokinase)" reversible="false" fast="false">
      <listOfReactants>
        <speciesReference species="species_15"/>
      </listOfReactants>
      <listOfProducts>
        <speciesReference species="species_14"/>
      </listOfProducts>
      <kineticLaw>
        <math xmlns="http://www.w3.org/1998/Math/MathML">
          <apply>
            <divide/>
            <apply>
              <times/>
              <ci> mw61b0fed3_e790_4af3_a1e7_65c1646248bf </ci>
              <ci> species_15 </ci>
            </apply>
            <apply>
              <plus/>
              <ci> species_15 </ci>
              <ci> mw9d451500_8215_4269_83be_e237d42f0aab </ci>
            </apply>
          </apply>
        </math>
      </kineticLaw>
    </reaction>
```

<reaction id="mw31133011_39d2_478f_bee1_c84fabc8343e" name="CC_6 beta (fructose-1,6-bisphosphatase)" reversible="false" fast="false">

<listOfReactants>

<speciesReference species="species_14"/>

</listOfReactants>

<listOfProducts>

<speciesReference species="species_15"/>

<speciesReference species="species_12"/>

</listOfProducts>

<kineticLaw>

<math xmlns="http://www.w3.org/1998/Math/MathML">

<apply>

<divide/>

<apply>

<times/>

<ci> mw13a07b09_9a67_4561_b3db_8e39442e5d53 </ci>

<ci> species_14 </ci>

</apply>

<apply>

<plus/>

<ci> species_14 </ci>

<apply>

<times/>

<ci> mwdb1e1973_5df2_4a29_a277_64c677c902b4 </ci>

<apply>

<plus/>

<cn type="integer"> 1 </cn>

<apply>

```
            <divide/>
            <ci> species_15 </ci>
            <ci> mwd2bb54b2_f070_41d0_a500_9b196833f57a </ci>
           </apply>
           <apply>
            <divide/>
            <ci> species_12 </ci>
            <ci> mw9f468e68_fa4c_4260_a5af_3b6182abcb92 </ci>
           </apply>
          </apply>
         </apply>
        </apply>
       </apply>
      </math>
     </kineticLaw>
    </reaction>
    <reaction id="mwe1c332df_bf6c_404f_b207_fa3e8ee321b4" name="C_11 beta (phosphopentose
isomerase)" fast="false">
     <listOfReactants>
      <speciesReference species="species_18"/>
     </listOfReactants>
     <listOfProducts>
      <speciesReference species="species_20"/>
     </listOfProducts>
     <kineticLaw>
      <math xmlns="http://www.w3.org/1998/Math/MathML">
       <apply>
        <divide/>
```

```
<apply>
  <times/>
  <ci> mw15a7ce92_eb88_4188_966c_aad7e9d8ea7e </ci>
  <apply>
    <minus/>
    <ci> species_18 </ci>
    <apply>
      <divide/>
      <ci> species_20 </ci>
      <ci> mw94fab6bb_d1d8_4344_ac40_d9ea47173826 </ci>
    </apply>
  </apply>
</apply>
<apply>
  <times/>
  <ci> mw8da43b9c_22d4_4c48_bcc7_0bb2a83b77f6 </ci>
  <apply>
    <plus/>
    <cn type="integer"> 1 </cn>
    <apply>
      <divide/>
      <ci> species_18 </ci>
      <ci> mw8da43b9c_22d4_4c48_bcc7_0bb2a83b77f6 </ci>
    </apply>
    <apply>
      <divide/>
      <ci> species_20 </ci>
```

```
          <ci> mw332683df_4959_4366_94ef_2b7108d780dc </ci>
         </apply>
        </apply>
       </apply>
      </apply>
     </math>
    </kineticLaw>
   </reaction>
   <reaction id="mw6ca245ce_5769_4e56_893d_f2a8e43f8119" name="PPP_1 (glucose-6-phosphate dehydrogenase)" reversible="false" fast="false">
    <listOfReactants>
     <speciesReference species="species_21"/>
    </listOfReactants>
    <listOfProducts>
     <speciesReference species="mw3447e887_c5e2_4271_9e5f_50c309bc8dc3"/>
    </listOfProducts>
    <kineticLaw>
     <math xmlns="http://www.w3.org/1998/Math/MathML">
      <apply>
       <divide/>
       <apply>
        <times/>
        <ci> mw84e3d5fb_d3f5_4a42_a963_2d0e4de0c9fa </ci>
        <ci> species_21 </ci>
       </apply>
       <apply>
        <times/>
        <ci> mw212994b8_ca26_4fbc_a13e_bb054a312812 </ci>
```

```
            <apply>
              <plus/>
              <cn type="integer"> 1 </cn>
              <apply>
                <divide/>
                <ci> species_21 </ci>
                <ci> mw212994b8_ca26_4fbc_a13e_bb054a312812 </ci>
              </apply>
              <apply>
                <divide/>
                <ci> mw3447e887_c5e2_4271_9e5f_50c309bc8dc3 </ci>
                <ci> mw80012d61_bf17_467d_8029_8785e8b66833 </ci>
              </apply>
            </apply>
          </apply>
        </apply>
      </math>
    </kineticLaw>
  </reaction>
  <reaction id="mw068859c7_1ee9_41f0_aaab_4cb88de606d7" name="PPP_2 (phosphogluconate
dehydrogenase)" reversible="false" fast="false">
    <listOfReactants>
      <speciesReference species="mw3447e887_c5e2_4271_9e5f_50c309bc8dc3"/>
      <speciesReference species="species_11"/>
    </listOfReactants>
    <listOfProducts>
      <speciesReference species="species_20"/>
      <speciesReference species="species_5"/>
```

```
</listOfProducts>
<kineticLaw>
  <math xmlns="http://www.w3.org/1998/Math/MathML">
    <apply>
      <divide/>
      <apply>
        <times/>
        <ci> mwacee8ded_69a8_4141_87f2_2622f06daa29 </ci>
        <ci> mw3447e887_c5e2_4271_9e5f_50c309bc8dc3 </ci>
        <ci> species_11 </ci>
      </apply>
      <apply>
        <times/>
        <apply>
          <plus/>
          <ci> mw3447e887_c5e2_4271_9e5f_50c309bc8dc3 </ci>
          <ci> mw9aad6b3a_ddc4_4aa7_af1d_9c6f98a72a80 </ci>
        </apply>
        <apply>
          <plus/>
          <ci> species_11 </ci>
          <ci> mwbf2d484b_0c51_4597_84e7_a9d72a8ed607 </ci>
        </apply>
      </apply>
    </apply>
  </math>
</kineticLaw>
```

```
      </reaction>
      <reaction id="mw84530e4e_bd49_4c49_96fa_3753a80777df" name="PP_2c (phosphoglycolate phosphatase)" reversible="false" fast="false">
        <listOfReactants>
          <speciesReference species="species_25"/>
        </listOfReactants>
        <listOfProducts>
          <speciesReference species="species_26"/>
          <speciesReference species="species_12"/>
        </listOfProducts>
        <kineticLaw>
          <math xmlns="http://www.w3.org/1998/Math/MathML">
            <apply>
              <divide/>
              <apply>
                <times/>
                <ci> mwe7492e74_1fd5_412c_9bd7_c33659cbb9ae </ci>
                <ci> species_25 </ci>
              </apply>
              <apply>
                <plus/>
                <ci> species_25 </ci>
                <apply>
                  <times/>
                  <ci> mw2026f64b_36e7_4da9_8f99_4179a8aa5a0f </ci>
                  <apply>
                    <plus/>
                    <cn type="integer"> 1 </cn>
```

```
          <apply>
            <divide/>
            <ci> species_26 </ci>
            <ci> mw42e7bfb9_db8f_4b73_bd5b_4f46a35e36a0 </ci>
          </apply>
        </apply>
        <apply>
          <plus/>
          <cn type="integer"> 1 </cn>
          <apply>
            <divide/>
            <ci> species_12 </ci>
            <ci> mw43a4fc8c_e159_44c4_aca5_0de4d39f2892 </ci>
          </apply>
        </apply>
      </apply>
    </apply>
   </apply>
  </math>
 </kineticLaw>
</reaction>
</listOfReactions>
</model>
</sbml>
```

Supplemental File S2 Model


```
<?xml version="1.0" encoding="UTF-8"?>

<sbml xmlns="http://www.sbml.org/sbml/level2" xmlns:html="http://www.w3.org/1999/xhtml"
level="2" version="1">

  <annotation>

Created by The MathWorks, Inc. SimBiology tool, Version 3.3

</annotation>

  <model id="Model_1" name="initial">

    <notes>

      <body xmlns="http://www.w3.org/1999/xhtml"></body>

    </notes>

    <annotation>

      <COPASI xmlns="http://www.copasi.org/static/sbml">

        <rdf:RDF xmlns:dcterms="http://purl.org/dc/terms/"
xmlns:rdf="http://www.w3.org/1999/02/22-rdf-syntax-ns#">

          <rdf:Description rdf:about="#COPASI1">

            <dcterms:created>

              <rdf:Description>

                <dcterms:W3CDTF>2010-04-14T14:47:02Z</dcterms:W3CDTF>

              </rdf:Description>

            </dcterms:created>

          </rdf:Description>

        </rdf:RDF>

      </COPASI>

    </annotation>

    <listOfCompartments>

      <compartment id="mwc7a0146b_dcaf_459b_8aad_1ff6e20a3fb5" name="Stroma" size="1">

        <annotation>

          <COPASI xmlns="http://www.copasi.org/static/sbml">
```

```
<rdf:RDF xmlns:dcterms="http://purl.org/dc/terms/"
xmlns:rdf="http://www.w3.org/1999/02/22-rdf-syntax-ns#">
<rdf:Description rdf:about="#COPASI2">
<dcterms:created>
<rdf:Description>
<dcterms:W3CDTF>2010-04-14T14:56:43Z</dcterms:W3CDTF>
</rdf:Description>
</dcterms:created>
</rdf:Description>
</rdf:RDF>
</COPASI>
</annotation>
</compartment>
<compartment id="mw1341fc13_2fe2_46f4_b24f_cd32c24b8dca" name="external" size="1"/>
<compartment id="mw99892ac6_be79_4769_b284_4c503a78023d" name="Stroma Stroma"
size="1"/>
</listOfCompartments>
<listOfSpecies>
<species id="species_1" name="CO2"
compartment="mwc7a0146b_dcaf_459b_8aad_1ff6e20a3fb5" initialAmount="0.02">
<annotation>
<COPASI xmlns="http://www.copasi.org/static/sbml">
<rdf:RDF xmlns:dcterms="http://purl.org/dc/terms/"
xmlns:rdf="http://www.w3.org/1999/02/22-rdf-syntax-ns#">
<rdf:Description rdf:about="#COPASI3">
<dcterms:created>
<rdf:Description>
<dcterms:W3CDTF>2010-04-14T15:46:19Z</dcterms:W3CDTF>
</rdf:Description>
```

</dcterms:created>

</rdf:Description>

</rdf:RDF>

</COPASI>

</annotation>

</species>

<species id="species_2" name="PGA" compartment="mwc7a0146b_dcaf_459b_8aad_1ff6e20a3fb5" initialAmount="4.7">

<annotation>

<COPASI xmlns="http://www.copasi.org/static/sbml">

<rdf:RDF xmlns:dcterms="http://purl.org/dc/terms/" xmlns:rdf="http://www.w3.org/1999/02/22-rdf-syntax-ns#">

<rdf:Description rdf:about="#COPASI4">

<dcterms:created>

<rdf:Description>

<dcterms:W3CDTF>2010-04-14T15:47:21Z</dcterms:W3CDTF>

</rdf:Description>

</dcterms:created>

</rdf:Description>

</rdf:RDF>

</COPASI>

</annotation>

</species>

<species id="species_3" name="RuBP" compartment="mwc7a0146b_dcaf_459b_8aad_1ff6e20a3fb5" initialAmount="1">

<annotation>

<COPASI xmlns="http://www.copasi.org/static/sbml">

<rdf:RDF xmlns:dcterms="http://purl.org/dc/terms/" xmlns:rdf="http://www.w3.org/1999/02/22-rdf-syntax-ns#">

<rdf:Description rdf:about="#COPASI5">
<dcterms:created>
<rdf:Description>
<dcterms:W3CDTF>2010-04-14T15:47:38Z</dcterms:W3CDTF>
</rdf:Description>
</dcterms:created>
</rdf:Description>
</rdf:RDF>
</COPASI>
</annotation>
</species>
<species id="species_5" name="NADPH" compartment="mwc7a0146b_dcaf_459b_8aad_1ff6e20a3fb5" initialAmount="0.148995513447536">
<annotation>
<COPASI xmlns="http://www.copasi.org/static/sbml">
<rdf:RDF xmlns:dcterms="http://purl.org/dc/terms/" xmlns:rdf="http://www.w3.org/1999/02/22-rdf-syntax-ns#">
<rdf:Description rdf:about="#COPASI6">
<dcterms:created>
<rdf:Description>
<dcterms:W3CDTF>2010-04-14T15:47:02Z</dcterms:W3CDTF>
</rdf:Description>
</dcterms:created>
</rdf:Description>
</rdf:RDF>
</COPASI>
</annotation>
</species>

```
<species id="species_6" name="O2"
compartment="mwc7a0146b_dcaf_459b_8aad_1ff6e20a3fb5"
initialAmount="0.0259999987479538" boundaryCondition="true" constant="true">
  <annotation>
    <COPASI xmlns="http://www.copasi.org/static/sbml">
      <rdf:RDF xmlns:dcterms="http://purl.org/dc/terms/"
xmlns:rdf="http://www.w3.org/1999/02/22-rdf-syntax-ns#">
        <rdf:Description rdf:about="#COPASI7">
          <dcterms:created>
            <rdf:Description>
              <dcterms:W3CDTF>2010-04-14T15:47:13Z</dcterms:W3CDTF>
            </rdf:Description>
          </dcterms:created>
        </rdf:Description>
      </rdf:RDF>
    </COPASI>
  </annotation>
</species>
<species id="species_8" name="SBP"
compartment="mwc7a0146b_dcaf_459b_8aad_1ff6e20a3fb5" initialAmount="1.36261887752912">
  <annotation>
    <COPASI xmlns="http://www.copasi.org/static/sbml">
      <rdf:RDF xmlns:dcterms="http://purl.org/dc/terms/"
xmlns:rdf="http://www.w3.org/1999/02/22-rdf-syntax-ns#">
        <rdf:Description rdf:about="#COPASI8">
          <dcterms:created>
            <rdf:Description>
              <dcterms:W3CDTF>2010-04-14T15:47:55Z</dcterms:W3CDTF>
            </rdf:Description>
```

```
          </dcterms:created>
        </rdf:Description>
      </rdf:RDF>
    </COPASI>
  </annotation>
</species>
<species id="species_4" name="ADP"
compartment="mwc7a0146b_dcaf_459b_8aad_1ff6e20a3fb5"
initialAmount="0.344798607704336">
  <annotation>
    <COPASI xmlns="http://www.copasi.org/static/sbml">
      <rdf:RDF xmlns:dcterms="http://purl.org/dc/terms/"
xmlns:rdf="http://www.w3.org/1999/02/22-rdf-syntax-ns#">
        <rdf:Description rdf:about="#COPASI9">
          <dcterms:created>
            <rdf:Description>
              <dcterms:W3CDTF>2010-04-14T16:01:25Z</dcterms:W3CDTF>
            </rdf:Description>
          </dcterms:created>
        </rdf:Description>
      </rdf:RDF>
    </COPASI>
  </annotation>
</species>
<species id="species_7" name="ATP"
compartment="mwc7a0146b_dcaf_459b_8aad_1ff6e20a3fb5" initialAmount="1.15520132006222">
  <annotation>
    <COPASI xmlns="http://www.copasi.org/static/sbml">
```

<rdf:RDF xmlns:dcterms="http://purl.org/dc/terms/" xmlns:rdf="http://www.w3.org/1999/02/22-rdf-syntax-ns#">

<rdf:Description rdf:about="#COPASI10">

<dcterms:created>

<rdf:Description>

<dcterms:W3CDTF>2010-04-14T16:01:14Z</dcterms:W3CDTF>

</rdf:Description>

</dcterms:created>

</rdf:Description>

</rdf:RDF>

</COPASI>

</annotation>

</species>

<species id="species_9" name="BPGA" compartment="mwc7a0146b_dcaf_459b_8aad_1ff6e20a3fb5" initialAmount="0.00748248557057663">

<annotation>

<COPASI xmlns="http://www.copasi.org/static/sbml">

<rdf:RDF xmlns:dcterms="http://purl.org/dc/terms/" xmlns:rdf="http://www.w3.org/1999/02/22-rdf-syntax-ns#">

<rdf:Description rdf:about="#COPASI11">

<dcterms:created>

<rdf:Description>

<dcterms:W3CDTF>2010-04-14T16:01:08Z</dcterms:W3CDTF>

</rdf:Description>

</dcterms:created>

</rdf:Description>

</rdf:RDF>

</COPASI>

</annotation>
</species>
<species id="species_10" name="GAP" compartment="mwc7a0146b_dcaf_459b_8aad_1ff6e20a3fb5" initialAmount="0.368362636458716">
<annotation>
<COPASI xmlns="http://www.copasi.org/static/sbml">
<rdf:RDF xmlns:dcterms="http://purl.org/dc/terms/" xmlns:rdf="http://www.w3.org/1999/02/22-rdf-syntax-ns#">
<rdf:Description rdf:about="#COPASI12">
<dcterms:created>
<rdf:Description>
<dcterms:W3CDTF>2010-06-08T23:46:46Z</dcterms:W3CDTF>
</rdf:Description>
</dcterms:created>
</rdf:Description>
</rdf:RDF>
</COPASI>
</annotation>
</species>
<species id="species_11" name="NADPp" compartment="mwc7a0146b_dcaf_459b_8aad_1ff6e20a3fb5" initialAmount="0.271004466327097">
<annotation>
<COPASI xmlns="http://www.copasi.org/static/sbml">
<rdf:RDF xmlns:dcterms="http://purl.org/dc/terms/" xmlns:rdf="http://www.w3.org/1999/02/22-rdf-syntax-ns#">
<rdf:Description rdf:about="#COPASI13">
<dcterms:created>
<rdf:Description>

```
<dcterms:W3CDTF>2010-06-08T23:43:15Z</dcterms:W3CDTF>
</rdf:Description>
</dcterms:created>
</rdf:Description>
</rdf:RDF>
</COPASI>
</annotation>
</species>
<species id="species_12" name="Pi"
compartment="mwc7a0146b_dcaf_459b_8aad_1ff6e20a3fb5" initialAmount="4.99999975922188"
boundaryCondition="true" constant="true">
<annotation>
<COPASI xmlns="http://www.copasi.org/static/sbml">
<rdf:RDF xmlns:dcterms="http://purl.org/dc/terms/"
xmlns:rdf="http://www.w3.org/1999/02/22-rdf-syntax-ns#">
<rdf:Description rdf:about="#COPASI14">
<dcterms:created>
<rdf:Description>
<dcterms:W3CDTF>2010-05-18T18:10:26Z</dcterms:W3CDTF>
</rdf:Description>
</dcterms:created>
</rdf:Description>
</rdf:RDF>
</COPASI>
</annotation>
</species>
<species id="species_13" name="DHAP"
compartment="mwc7a0146b_dcaf_459b_8aad_1ff6e20a3fb5" initialAmount="0.012">
<annotation>
```

<COPASI xmlns="http://www.copasi.org/static/sbml">
<rdf:RDF xmlns:dcterms="http://purl.org/dc/terms/" xmlns:rdf="http://www.w3.org/1999/02/22-rdf-syntax-ns#">
<rdf:Description rdf:about="#COPASI15">
<dcterms:created>
<rdf:Description>
<dcterms:W3CDTF>2010-06-08T23:43:34Z</dcterms:W3CDTF>
</rdf:Description>
</dcterms:created>
</rdf:Description>
</rdf:RDF>
</COPASI>
</annotation>
</species>
<species id="species_14" name="FBP" compartment="mwc7a0146b_dcaf_459b_8aad_1ff6e20a3fb5" initialAmount="0.02">
<annotation>
<COPASI xmlns="http://www.copasi.org/static/sbml">
<rdf:RDF xmlns:dcterms="http://purl.org/dc/terms/" xmlns:rdf="http://www.w3.org/1999/02/22-rdf-syntax-ns#">
<rdf:Description rdf:about="#COPASI16">
<dcterms:created>
<rdf:Description>
<dcterms:W3CDTF>2010-06-08T23:44:30Z</dcterms:W3CDTF>
</rdf:Description>
</dcterms:created>
</rdf:Description>
</rdf:RDF>
</COPASI>

</annotation>

</species>

<species id="species_15" name="F6P" compartment="mwc7a0146b_dcaf_459b_8aad_1ff6e20a3fb5" initialAmount="2">

<annotation>

<COPASI xmlns="http://www.copasi.org/static/sbml">

<rdf:RDF xmlns:dcterms="http://purl.org/dc/terms/" xmlns:rdf="http://www.w3.org/1999/02/22-rdf-syntax-ns#">

<rdf:Description rdf:about="#COPASI17">

<dcterms:created>

<rdf:Description>

<dcterms:W3CDTF>2010-06-08T23:44:21Z</dcterms:W3CDTF>

</rdf:Description>

</dcterms:created>

</rdf:Description>

</rdf:RDF>

</COPASI>

</annotation>

</species>

<species id="species_16" name="E4P" compartment="mwc7a0146b_dcaf_459b_8aad_1ff6e20a3fb5" initialAmount="0.228820226685508">

<annotation>

<COPASI xmlns="http://www.copasi.org/static/sbml">

<rdf:RDF xmlns:dcterms="http://purl.org/dc/terms/" xmlns:rdf="http://www.w3.org/1999/02/22-rdf-syntax-ns#">

<rdf:Description rdf:about="#COPASI18">

<dcterms:created>

<rdf:Description>

<dcterms:W3CDTF>2010-06-08T23:43:55Z</dcterms:W3CDTF>

</rdf:Description>

</dcterms:created>

</rdf:Description>

</rdf:RDF>

</COPASI>

</annotation>

</species>

<species id="species_17" name="Xu5P" compartment="mwc7a0146b_dcaf_459b_8aad_1ff6e20a3fb5" initialAmount="0.0558077870361159">

<annotation>

<COPASI xmlns="http://www.copasi.org/static/sbml">

<rdf:RDF xmlns:dcterms="http://purl.org/dc/terms/" xmlns:rdf="http://www.w3.org/1999/02/22-rdf-syntax-ns#">

<rdf:Description rdf:about="#COPASI19">

<dcterms:created>

<rdf:Description>

<dcterms:W3CDTF>2010-06-08T23:41:49Z</dcterms:W3CDTF>

</rdf:Description>

</dcterms:created>

</rdf:Description>

</rdf:RDF>

</COPASI>

</annotation>

</species>

<species id="species_18" name="Ri5P" compartment="mwc7a0146b_dcaf_459b_8aad_1ff6e20a3fb5" initialAmount="0.0744045363011372">

<annotation>

<COPASI xmlns="http://www.copasi.org/static/sbml">

<rdf:RDF xmlns:dcterms="http://purl.org/dc/terms/"
xmlns:rdf="http://www.w3.org/1999/02/22-rdf-syntax-ns#">

<rdf:Description rdf:about="#COPASI20">

<dcterms:created>

<rdf:Description>

<dcterms:W3CDTF>2010-06-08T23:41:08Z</dcterms:W3CDTF>

</rdf:Description>

</dcterms:created>

</rdf:Description>

</rdf:RDF>

</COPASI>

</annotation>

</species>

<species id="species_19" name="S7P"
compartment="mwc7a0146b_dcaf_459b_8aad_1ff6e20a3fb5"
initialAmount="0.511319423085088">

<annotation>

<COPASI xmlns="http://www.copasi.org/static/sbml">

<rdf:RDF xmlns:dcterms="http://purl.org/dc/terms/"
xmlns:rdf="http://www.w3.org/1999/02/22-rdf-syntax-ns#">

<rdf:Description rdf:about="#COPASI21">

<dcterms:created>

<rdf:Description>

<dcterms:W3CDTF>2010-06-08T23:47:56Z</dcterms:W3CDTF>

</rdf:Description>

</dcterms:created>

</rdf:Description>

</rdf:RDF>

</COPASI>

</annotation>

</species>

<species id="species_20" name="Ru5P" compartment="mwc7a0146b_dcaf_459b_8aad_1ff6e20a3fb5" initialAmount="0.026898222838205">

<annotation>

<COPASI xmlns="http://www.copasi.org/static/sbml">

<rdf:RDF xmlns:dcterms="http://purl.org/dc/terms/" xmlns:rdf="http://www.w3.org/1999/02/22-rdf-syntax-ns#">

<rdf:Description rdf:about="#COPASI22">

<dcterms:created>

<rdf:Description>

<dcterms:W3CDTF>2010-06-08T23:41:17Z</dcterms:W3CDTF>

</rdf:Description>

</dcterms:created>

</rdf:Description>

</rdf:RDF>

</COPASI>

</annotation>

</species>

<species id="species_21" name="G6P" compartment="mwc7a0146b_dcaf_459b_8aad_1ff6e20a3fb5" initialAmount="1.94057630985951">

<annotation>

<COPASI xmlns="http://www.copasi.org/static/sbml">

<rdf:RDF xmlns:dcterms="http://purl.org/dc/terms/" xmlns:rdf="http://www.w3.org/1999/02/22-rdf-syntax-ns#">

<rdf:Description rdf:about="#COPASI23">

```
            <dcterms:created>
              <rdf:Description>
                <dcterms:W3CDTF>2010-06-08T23:46:39Z</dcterms:W3CDTF>
              </rdf:Description>
            </dcterms:created>
          </rdf:Description>
        </rdf:RDF>
      </COPASI>
    </annotation>
  </species>
  <species id="species_23" name="Sink1"
compartment="mwc7a0146b_dcaf_459b_8aad_1ff6e20a3fb5" initialAmount="0">
    <annotation>
      <COPASI xmlns="http://www.copasi.org/static/sbml">
        <rdf:RDF xmlns:dcterms="http://purl.org/dc/terms/"
xmlns:rdf="http://www.w3.org/1999/02/22-rdf-syntax-ns#">
          <rdf:Description rdf:about="#COPASI25">
            <dcterms:created>
              <rdf:Description>
                <dcterms:W3CDTF>2011-05-10T00:14:21Z</dcterms:W3CDTF>
              </rdf:Description>
            </dcterms:created>
          </rdf:Description>
        </rdf:RDF>
      </COPASI>
    </annotation>
  </species>
  <species id="species_24" name="Sink2"
compartment="mwc7a0146b_dcaf_459b_8aad_1ff6e20a3fb5" initialAmount="0">
```

<annotation>

<COPASI xmlns="http://www.copasi.org/static/sbml">

<rdf:RDF xmlns:dcterms="http://purl.org/dc/terms/" xmlns:rdf="http://www.w3.org/1999/02/22-rdf-syntax-ns#">

<rdf:Description rdf:about="#COPASI26">

<dcterms:created>

<rdf:Description>

<dcterms:W3CDTF>2011-04-26T00:48:45Z</dcterms:W3CDTF>

</rdf:Description>

</dcterms:created>

</rdf:Description>

</rdf:RDF>

</COPASI>

</annotation>

</species>

<species id="species_25" name="PGCA" compartment="mwc7a0146b_dcaf_459b_8aad_1ff6e20a3fb5" initialAmount="0.01">

<annotation>

<COPASI xmlns="http://www.copasi.org/static/sbml">

<rdf:RDF xmlns:dcterms="http://purl.org/dc/terms/" xmlns:rdf="http://www.w3.org/1999/02/22-rdf-syntax-ns#">

<rdf:Description rdf:about="#COPASI27">

<dcterms:created>

<rdf:Description>

<dcterms:W3CDTF>2011-06-15T16:28:14Z</dcterms:W3CDTF>

</rdf:Description>

</dcterms:created>

</rdf:Description>

</rdf:RDF>

</COPASI>

</annotation>

</species>

<species id="species_26" name="GCA" compartment="mwc7a0146b_dcaf_459b_8aad_1ff6e20a3fb5" initialAmount="0.0123593835433703">

<annotation>

<COPASI xmlns="http://www.copasi.org/static/sbml">

<rdf:RDF xmlns:dcterms="http://purl.org/dc/terms/" xmlns:rdf="http://www.w3.org/1999/02/22-rdf-syntax-ns#">

<rdf:Description rdf:about="#COPASI28">

<dcterms:created>

<rdf:Description>

<dcterms:W3CDTF>2011-06-15T16:28:15Z</dcterms:W3CDTF>

</rdf:Description>

</dcterms:created>

</rdf:Description>

</rdf:RDF>

</COPASI>

</annotation>

</species>

<species id="species_27" name="GOA" compartment="mwc7a0146b_dcaf_459b_8aad_1ff6e20a3fb5" initialAmount="0.0327363830028097">

<annotation>

<COPASI xmlns="http://www.copasi.org/static/sbml">

<rdf:RDF xmlns:dcterms="http://purl.org/dc/terms/" xmlns:rdf="http://www.w3.org/1999/02/22-rdf-syntax-ns#">

<rdf:Description rdf:about="#COPASI29">

<dcterms:created>

<rdf:Description>

<dcterms:W3CDTF>2011-06-15T16:28:23Z</dcterms:W3CDTF>

</rdf:Description>

</dcterms:created>

</rdf:Description>

</rdf:RDF>

</COPASI>

</annotation>

</species>

<species id="species_28" name="GLY" compartment="mwc7a0146b_dcaf_459b_8aad_1ff6e20a3fb5" initialAmount="0.8">

<annotation>

<COPASI xmlns="http://www.copasi.org/static/sbml">

<rdf:RDF xmlns:dcterms="http://purl.org/dc/terms/" xmlns:rdf="http://www.w3.org/1999/02/22-rdf-syntax-ns#">

<rdf:Description rdf:about="#COPASI30">

<dcterms:created>

<rdf:Description>

<dcterms:W3CDTF>2011-06-15T16:27:59Z</dcterms:W3CDTF>

</rdf:Description>

</dcterms:created>

</rdf:Description>

</rdf:RDF>

</COPASI>

</annotation>

</species>

<species id="species_29" name="SER" compartment="mwc7a0146b_dcaf_459b_8aad_1ff6e20a3fb5" initialAmount="0.5">

<annotation>

```
<COPASI xmlns="http://www.copasi.org/static/sbml">
<rdf:RDF xmlns:dcterms="http://purl.org/dc/terms/"
xmlns:rdf="http://www.w3.org/1999/02/22-rdf-syntax-ns#">
<rdf:Description rdf:about="#COPASI31">
<dcterms:created>
<rdf:Description>
<dcterms:W3CDTF>2011-06-15T16:28:35Z</dcterms:W3CDTF>
</rdf:Description>
</dcterms:created>
</rdf:Description>
</rdf:RDF>
</COPASI>
</annotation>
</species>
<species id="species_30" name="HPR"
compartment="mwc7a0146b_dcaf_459b_8aad_1ff6e20a3fb5" initialAmount="0.1">
<annotation>
<COPASI xmlns="http://www.copasi.org/static/sbml">
<rdf:RDF xmlns:dcterms="http://purl.org/dc/terms/"
xmlns:rdf="http://www.w3.org/1999/02/22-rdf-syntax-ns#">
<rdf:Description rdf:about="#COPASI32">
<dcterms:created>
<rdf:Description>
<dcterms:W3CDTF>2011-06-15T16:38:48Z</dcterms:W3CDTF>
</rdf:Description>
</dcterms:created>
</rdf:Description>
</rdf:RDF>
</COPASI>
```

</annotation>

</species>

<species id="species_31" name="GCEA" compartment="mwc7a0146b_dcaf_459b_8aad_1ff6e20a3fb5" initialAmount="0.00400097153227282">

<annotation>

<COPASI xmlns="http://www.copasi.org/static/sbml">

<rdf:RDF xmlns:dcterms="http://purl.org/dc/terms/" xmlns:rdf="http://www.w3.org/1999/02/22-rdf-syntax-ns#">

<rdf:Description rdf:about="#COPASI33">

<dcterms:created>

<rdf:Description>

<dcterms:W3CDTF>2011-06-15T16:30:28Z</dcterms:W3CDTF>

</rdf:Description>

</dcterms:created>

</rdf:Description>

</rdf:RDF>

</COPASI>

</annotation>

</species>

<species id="species_32" name="PEP" compartment="mwc7a0146b_dcaf_459b_8aad_1ff6e20a3fb5" initialAmount="4.19091918967107">

<annotation>

<COPASI xmlns="http://www.copasi.org/static/sbml">

<rdf:RDF xmlns:dcterms="http://purl.org/dc/terms/" xmlns:rdf="http://www.w3.org/1999/02/22-rdf-syntax-ns#">

<rdf:Description rdf:about="#COPASI34">

<dcterms:created>

<rdf:Description>

<dcterms:W3CDTF>2011-05-10T16:27:35Z</dcterms:W3CDTF>

</rdf:Description>

</dcterms:created>

</rdf:Description>

</rdf:RDF>

</COPASI>

</annotation>

</species>

<species id="species_33" name="Sink3" compartment="mwc7a0146b_dcaf_459b_8aad_1ff6e20a3fb5" initialAmount="0">

<annotation>

<COPASI xmlns="http://www.copasi.org/static/sbml">

<rdf:RDF xmlns:dcterms="http://purl.org/dc/terms/" xmlns:rdf="http://www.w3.org/1999/02/22-rdf-syntax-ns#">

<rdf:Description rdf:about="#COPASI35">

<dcterms:created>

<rdf:Description>

<dcterms:W3CDTF>2011-05-10T16:30:50Z</dcterms:W3CDTF>

</rdf:Description>

</dcterms:created>

</rdf:Description>

</rdf:RDF>

</COPASI>

</annotation>

</species>

<species id="species_34" name="Sink4" compartment="mwc7a0146b_dcaf_459b_8aad_1ff6e20a3fb5" initialAmount="0">

<annotation>

<COPASI xmlns="http://www.copasi.org/static/sbml">

```
<rdf:RDF xmlns:dcterms="http://purl.org/dc/terms/"
xmlns:rdf="http://www.w3.org/1999/02/22-rdf-syntax-ns#">
<rdf:Description rdf:about="#COPASI36">
<dcterms:created>
<rdf:Description>
<dcterms:W3CDTF>2011-06-15T16:28:43Z</dcterms:W3CDTF>
</rdf:Description>
</dcterms:created>
</rdf:Description>
</rdf:RDF>
</COPASI>
</annotation>
</species>
<species id="species_35" name="ppGA"
compartment="mwc7a0146b_dcaf_459b_8aad_1ff6e20a3fb5" initialAmount="4.19091917967427">
<annotation>
<COPASI xmlns="http://www.copasi.org/static/sbml">
<rdf:RDF xmlns:dcterms="http://purl.org/dc/terms/"
xmlns:rdf="http://www.w3.org/1999/02/22-rdf-syntax-ns#">
<rdf:Description rdf:about="#COPASI37">
<dcterms:created>
<rdf:Description>
<dcterms:W3CDTF>2011-06-15T16:30:09Z</dcterms:W3CDTF>
</rdf:Description>
</dcterms:created>
</rdf:Description>
</rdf:RDF>
</COPASI>
</annotation>
```

```
</species>
<species id="species_36" name="TSA"
compartment="mwc7a0146b_dcaf_459b_8aad_1ff6e20a3fb5"
initialAmount="0.0324331443243821">
<annotation>
<COPASI xmlns="http://www.copasi.org/static/sbml">
<rdf:RDF xmlns:dcterms="http://purl.org/dc/terms/"
xmlns:rdf="http://www.w3.org/1999/02/22-rdf-syntax-ns#">
<rdf:Description rdf:about="#COPASI38">
<dcterms:created>
<rdf:Description>
<dcterms:W3CDTF>2011-06-15T16:42:10Z</dcterms:W3CDTF>
</rdf:Description>
</dcterms:created>
</rdf:Description>
</rdf:RDF>
</COPASI>
</annotation>
</species>
<species id="species_37" name="OXA"
compartment="mwc7a0146b_dcaf_459b_8aad_1ff6e20a3fb5" initialAmount="0.1">
<annotation>
<COPASI xmlns="http://www.copasi.org/static/sbml">
<rdf:RDF xmlns:dcterms="http://purl.org/dc/terms/"
xmlns:rdf="http://www.w3.org/1999/02/22-rdf-syntax-ns#">
<rdf:Description rdf:about="#COPASI39">
<dcterms:created>
<rdf:Description>
<dcterms:W3CDTF>2011-06-28T01:15:08Z</dcterms:W3CDTF>
```

</rdf:Description>

</dcterms:created>

</rdf:Description>

</rdf:RDF>

</COPASI>

</annotation>

</species>

<species id="mw110e8daa_fa0e_409b_af62_9f01d755a8e3" name="ATPr" compartment="mwc7a0146b_dcaf_459b_8aad_1ff6e20a3fb5" initialAmount="0"/>

<species id="mwda7da32a_797a_4dbc_9e6a_c9317b3838b5" name="Mass" compartment="mwc7a0146b_dcaf_459b_8aad_1ff6e20a3fb5" initialAmount="0"/>

<species id="mw8369442e_7e34_45ca_89a9_7f0ab2ab70ee" name="Sink_DHAP" compartment="mwc7a0146b_dcaf_459b_8aad_1ff6e20a3fb5" initialAmount="0"/>

<species id="mw09d7964e_16d4_4c3d_a1f5_d98a7ab66891" name="Sink_E4P" compartment="mwc7a0146b_dcaf_459b_8aad_1ff6e20a3fb5" initialAmount="0"/>

<species id="mw70a9d4b8_2ca3_4692_a159_702d8464b7e7" name="Sink_Ri5P" compartment="mwc7a0146b_dcaf_459b_8aad_1ff6e20a3fb5" initialAmount="0"/>

<species id="mwa8568197_97f2_4c67_8727_f8d9ed6895e3" name="Sink_PEP" compartment="mwc7a0146b_dcaf_459b_8aad_1ff6e20a3fb5" initialAmount="0"/>

<species id="mwaf22c073_9b9c_42b1_bd8e_6e65777ddddc" name="Sink_GAP" compartment="mwc7a0146b_dcaf_459b_8aad_1ff6e20a3fb5" initialAmount="0"/>

<species id="mw7f9b1aa3_a2ab_4207_b81f_4ad133f8ccf8" name="NADPHr" compartment="mwc7a0146b_dcaf_459b_8aad_1ff6e20a3fb5" initialAmount="0"/>

<species id="mw4412eef8_75f9_408b_8ca1_0cd634ded124" name="rate_CC_1" compartment="mwc7a0146b_dcaf_459b_8aad_1ff6e20a3fb5" initialAmount="0"/>

<species id="mw5bf04cb0_8acb_4c37_a547_e939d6416759" name="rate_CC_2" compartment="mwc7a0146b_dcaf_459b_8aad_1ff6e20a3fb5" initialAmount="0"/>

<species id="mwe2947217_6be6_428f_ac4e_28ae24c11d45" name="rate_CC_3" compartment="mwc7a0146b_dcaf_459b_8aad_1ff6e20a3fb5" initialAmount="0"/>

<species id="mwac50212d_9880_4c18_b404_443dcf85e43a" name="rate_CC_4" compartment="mwc7a0146b_dcaf_459b_8aad_1ff6e20a3fb5" initialAmount="0"/>

<species id="mwdad3e574_e8ae_4a2a_bc5b_5d2a8592dffc" name="rate_CC_5" compartment="mwc7a0146b_dcaf_459b_8aad_1ff6e20a3fb5" initialAmount="0"/>

<species id="mwc04a96b8_d70f_4bdc_854b_7367baf678c4" name="rate_CC_6" compartment="mwc7a0146b_dcaf_459b_8aad_1ff6e20a3fb5" initialAmount="0"/>

<species id="mw0ec64103_a8ce_438d_97b6_aecac492a2fb" name="rate_CC_7" compartment="mwc7a0146b_dcaf_459b_8aad_1ff6e20a3fb5" initialAmount="0"/>

<species id="mwc594876f_c9b0_4f4b_a245_d024b6f1931a" name="rate_CC_8" compartment="mwc7a0146b_dcaf_459b_8aad_1ff6e20a3fb5" initialAmount="0"/>

<species id="mwb8fa5238_b6ab_43ba_914d_8d9609a94211" name="rate_CC_9" compartment="mwc7a0146b_dcaf_459b_8aad_1ff6e20a3fb5" initialAmount="0"/>

<species id="mwd13e1918_33bb_4d69_9527_a7db921570eb" name="rate_CC_10" compartment="mwc7a0146b_dcaf_459b_8aad_1ff6e20a3fb5" initialAmount="0"/>

<species id="mwbe2ee4f3_36d2_4a42_8390_f8082ac79296" name="rate_CC_11" compartment="mwc7a0146b_dcaf_459b_8aad_1ff6e20a3fb5" initialAmount="0"/>

<species id="mw60c6f424_4d57_49bf_b130_811bec897e0a" name="rate_CC_12" compartment="mwc7a0146b_dcaf_459b_8aad_1ff6e20a3fb5" initialAmount="0"/>

<species id="mw96deb747_ef33_4a50_b36e_145767b53cf7" name="rate_CC_13" compartment="mwc7a0146b_dcaf_459b_8aad_1ff6e20a3fb5" initialAmount="0"/>

<species id="mw42c5ea06_edf8_46bd_b262_f9db84921975" name="rate_PFK" compartment="mwc7a0146b_dcaf_459b_8aad_1ff6e20a3fb5" initialAmount="0"/>

<species id="mw10231dd9_d13a_4822_a477_0b190284526c" name="rate_PGM" compartment="mwc7a0146b_dcaf_459b_8aad_1ff6e20a3fb5" initialAmount="0"/>

<species id="mw0e384600_a904_40dc_b3a6_de20a7ae04b8" name="rate_ENO" compartment="mwc7a0146b_dcaf_459b_8aad_1ff6e20a3fb5" initialAmount="0"/>

<species id="mwaecebc73_e8e8_4aa8_a5c9_9d2295bf77d4" name="rate_glyc_GAP" compartment="mwc7a0146b_dcaf_459b_8aad_1ff6e20a3fb5" initialAmount="0"/>

<species id="mwd008246b_4ac0_4550_9588_64a1e8664cd1" name="rate_PEPsink" compartment="mwc7a0146b_dcaf_459b_8aad_1ff6e20a3fb5" initialAmount="0"/>

<species id="mw8dc354b7_d1b2_4cc9_bbf9_5d423d6013fb" name="Sink_G6P" compartment="mwc7a0146b_dcaf_459b_8aad_1ff6e20a3fb5" initialAmount="0"/>

<species id="mwf7b66b1f_77b0_4024_99e5_fb37fb0ab929" name="rate_G6Psink" compartment="mwc7a0146b_dcaf_459b_8aad_1ff6e20a3fb5" initialAmount="0"/>

<species id="mw6eb33847_58dd_4306_8ae9_7d68bb2a5612" name="rate_GAPsink" compartment="mwc7a0146b_dcaf_459b_8aad_1ff6e20a3fb5" initialAmount="0"/>

<species id="mw4a42b5e7_485f_4d6b_a4ed_e490759e5f26" name="rate_E4Psink" compartment="mwc7a0146b_dcaf_459b_8aad_1ff6e20a3fb5" initialAmount="0"/>

```xml
<species id="mw3128a56f_9005_484d_92b7_1b5e45b577c6" name="rate_Ri5Psink" compartment="mwc7a0146b_dcaf_459b_8aad_1ff6e20a3fb5" initialAmount="0"/>

<species id="mwfe019cb4_4031_44c9_83f8_9ba5e8de4ed4" name="AceP" compartment="mwc7a0146b_dcaf_459b_8aad_1ff6e20a3fb5" initialAmount="0"/>

<species id="mwca272a09_38a7_4416_a781_d64c8e945cc4" name="rate_PKET" compartment="mwc7a0146b_dcaf_459b_8aad_1ff6e20a3fb5" initialAmount="0"/>

<species id="mwe556cd16_34e2_417a_b945_37061560bfe5" name="Sink_AceP" compartment="mwc7a0146b_dcaf_459b_8aad_1ff6e20a3fb5" initialAmount="0"/>

<species id="mw3447e887_c5e2_4271_9e5f_50c309bc8dc3" name="P6G" compartment="mwc7a0146b_dcaf_459b_8aad_1ff6e20a3fb5" initialAmount="0"/>

<species id="mwf41678a2_15b0_484e_b056_0c8a195fed4d" name="OPP_rate" compartment="mwc7a0146b_dcaf_459b_8aad_1ff6e20a3fb5" initialAmount="0"/>

<species id="mw733b668e_1dba_46ef_9777_e9331b0b590d" name="CO2" compartment="mw1341fc13_2fe2_46f4_b24f_cd32c24b8dca" initialAmount="0.1" boundaryCondition="true" constant="true"/>

</listOfSpecies>

<listOfParameters>

<parameter id="parameter_1" name="kf_ATP" value="0.16">

<annotation>

<COPASI xmlns="http://www.copasi.org/static/sbml">

<rdf:RDF xmlns:dcterms="http://purl.org/dc/terms/" xmlns:rdf="http://www.w3.org/1999/02/22-rdf-syntax-ns#">

<rdf:Description rdf:about="#COPASI47">

<dcterms:created>

<rdf:Description>

<dcterms:W3CDTF>2010-04-14T15:40:36Z</dcterms:W3CDTF>

</rdf:Description>

</dcterms:created>

</rdf:Description>

</rdf:RDF>

</COPASI>
```

```xml
          </annotation>
        </parameter>
        <parameter id="parameter_2" name="kf_NADPH" value="0.58">
          <annotation>
            <COPASI xmlns="http://www.copasi.org/static/sbml">
              <rdf:RDF xmlns:dcterms="http://purl.org/dc/terms/"
xmlns:rdf="http://www.w3.org/1999/02/22-rdf-syntax-ns#">
                <rdf:Description rdf:about="#COPASI48">
                  <dcterms:created>
                    <rdf:Description>
                      <dcterms:W3CDTF>2010-07-25T02:11:54Z</dcterms:W3CDTF>
                    </rdf:Description>
                  </dcterms:created>
                </rdf:Description>
              </rdf:RDF>
            </COPASI>
          </annotation>
        </parameter>
        <parameter id="mw9f919ddc_7006_491f_b827_5b5094d66dd0" name="V_PP1" value="0.009"/>
        <parameter id="mwf008a49f_a4ec_49ec_8cdc_30906d544233" name="V1" value="0.991"/>
        <parameter id="mwdbdd129f_0428_4a42_8427_cf18a0b5568c" name="V5" value="1.613"/>
        <parameter id="mwfe139de1_b141_420e_a4e5_f59c639c6cf0" name="V2" value="0.6"/>
        <parameter id="mwc727e6a6_e76e_44d0_89f4_bede015cb04c" name="V3" value="0.536"/>
        <parameter id="mw8c8ad41e_6d63_473b_960b_322d5feb6f84" name="V4" value="0.37"/>
        <parameter id="mw9dda631f_8096_4e56_a670_fdb559f36d12" name="V6" value="0.06"/>
        <parameter id="mwea0f726e_b6ff_4cf0_8dba_9db4cc0827be" name="V7" value="0.11"/>
        <parameter id="mw33ab4422_ba35_4d2b_9620_d274168c7e1d" name="V8" value="1.11"/>
        <parameter id="mw5c6f658c_7714_4fbb_be83_5ef825b3e08b" name="V9" value="1.2"/>
```

```xml
<parameter id="mw6e8005dd_31e8_483e_902a_8870307bf558" name="V10" value="0.13"/>
<parameter id="mw4fbb318e_c400_4af5_8e3f_e80aec639ee3" name="V11" value="1.1"/>
<parameter id="mw702a7f39_5675_48ad_953a_4bcbbdc8e1a2" name="V12" value="1.28"/>
<parameter id="mwd486ae05_3de1_42aa_9584_2edc458d0c59" name="V13" value="0.43"/>
<parameter id="mw243dc3c0_44b5_4ef5_b8ba_92846b4fee4c" name="V_SS1" value="0.99"/>
<parameter id="mwaeeded85_9203_48b6_89a9_9dbf6c0d38da" name="KE_SS1" value="0.8"/>
<parameter id="mw715a0d4a_6423_4755_a1b5_7d9e5b1852a2" name="V_Sink_E4P" value="0.00012"/>
<parameter id="mw6caa1525_e69b_4ab2_b8c7_2b7deeb5ee58" name="V_Sink_Ri5P" value="0.005"/>
<parameter id="mw3c5b8b60_a85d_4b23_8f30_04a8715af44c" name="V_PP2a" value="0.0007"/>
<parameter id="mw5f743344_e84b_4721_8bd4_3643964b38ea" name="V_PP3" value="0.0042"/>
<parameter id="mw43b7b905_45eb_4193_9d33_c0623544faba" name="V_PP4" value="0.43"/>
<parameter id="mw21878232_3329_413d_9ad8_7dd1cfc2be9c" name="V_PP5" value="0.002"/>
<parameter id="mw9331ee05_e8ae_4da7_be3c_2ae749a0c97a" name="V_PP6" value="0.004"/>
<parameter id="mw4a170042_d461_4f96_b16d_2969db08423c" name="V_PP7" value="0.013"/>
<parameter id="mw5723da7e_7ee9_4b45_b397_c4fad0251fa2" name="V_Sink_PEP" value="0.021"/>
<parameter id="mwa5897908_6cba_4e9b_ae16_669f6cc2215d" name="V_synth_SER" value="0.0008"/>
<parameter id="mwf7e8c4cc_5866_4d9d_80a4_fb1d0db24ecf" name="V_TSA1" value="0.01"/>
<parameter id="mw0f93be3f_8f7b_48cf_b994_7d7d51c0c99d" name="V_TSA2" value="0.023"/>
<parameter id="mw4d57904b_347d_4323_a435_aef6db506bb9" name="V_TSA3" value="0.1"/>
<parameter id="mw7f7c8a85_afe2_49aa_8686_1b3950810d23" name="V_OXA1" value="0.12"/>
<parameter id="mwa562da41_e9ca_4d43_85ba_4372aa40bed8" name="V_OXA2" value="0.0002"/>
<parameter id="mwdde5b27b_4aa1_42aa_b292_6613ea7584a3" name="KE4" value="0.1"/>
<parameter id="mw4ec82ea3_67a6_47ad_971a_52a33f180466" name="KE7" value="0.3"/>
```

```xml
<parameter id="mwc59d3254_509c_411a_b656_9e80ac0a4d4a" name="KM_PFK" value="0.5"/>
<parameter id="mwfb9d0842_5ad1_42a2_b44c_12cfeaa36962" name="V_PFK" value="0.02"/>
<parameter id="mw701ca18f_a968_4128_8415_d39acae3e377" name="Km112b" value="0.1"/>
<parameter id="mwa378ec6c_6ace_46c3_a515_4be6d27ac144" name="V_PP2b" value="0.0005"/>
<parameter id="mw04118d88_412e_49b2_8ed5_aba0f23d1258" name="Km112a" value="3"/>
<parameter id="mwe806711a_3cbc_4d66_a530_d7ae4e673eea" name="Kms_enol" value="0.525"/>
<parameter id="mwdee0f9ac_28b4_444f_af80_30c299f78093" name="Kmp_enol" value="0.279"/>
<parameter id="mw70b2b9c1_0904_4851_aee4_8526d26a4c7f" name="K_Sink_PEP" value="0.674"/>
<parameter id="mw9c6e9e64_1ad3_462a_b257_61d666a10a15" name="V_Sink_GLY" value="0.0002"/>
<parameter id="mw108b366f_58cf_4f32_9f78_d3a14dd13026" name="V_Sink_SER" value="0.00194"/>
<parameter id="mw245d5cf2_30db_40c3_b1a7_ed389fff8808" name="KE5" value="1.27"/>
<parameter id="mw917c32b0_7a3f_4744_ac14_2e3d15da96f6" name="Keq_PGM" value="0.71"/>
<parameter id="mw231dd6ca_4db0_4cdb_adc8_72349abdba07" name="Kmp_PGM_alpha" value="0.74"/>
<parameter id="mw742fbec5_509e_42be_b7a8_12b9eeb49314" name="Kms_PGM_alpha" value="0.189"/>
<parameter id="mw4f571ae6_7040_40c6_b098_4ac7cf9ee24d" name="Vf_PGM_alpha" value="0.24"/>
<parameter id="mw23fae7dc_7d12_4fb0_98e2_c611e44007e1" name="Kmp_PGM_beta" value="0.005"/>
<parameter id="mwb99ebe97_7d91_406c_8b12_a56b2c939ff4" name="Kms_PGM_beta" value="0.277"/>
<parameter id="mw2ec1703a_729a_4b49_b4dd_4241b0d73907" name="Vf_PGM_beta" value="0.71"/>
<parameter id="mw23f5979c_8188_4521_9629_6469c95e42d6" name="Kmp_PGM_gama" value="0.6"/>
```

```xml
<parameter id="mw6205c3fd_ecf9_4b2b_98f1_dff68729cd03" name="Kms_PGM_gama" value="2.58"/>

<parameter id="mwa1879047_3c26_45c1_8b2a_137433500cde" name="Vf_PGM_gama" value="0.0278"/>

<parameter id="mwdacf4f8b_f1db_480a_aabf_ff89dc90e087" name="Keq_enol" value="0.866"/>

<parameter id="mw68a26bf2_520b_4b35_90e0_511ec4bbc859" name="Vf_enol" value="0.823"/>

<parameter id="mwaffea60c_8361_4bed_be10_814e0c0f7898" name="kf_CO2" value="1.91"/>

<parameter id="mw39e8b6c4_2ae7_4846_9ac8_e77ee9c7191f" name="KM51" value="0.3"/>

<parameter id="mw54c31bc3_16fc_462a_a55e_a766a97769ed" name="KM52" value="0.4"/>

<parameter id="mwbad28d74_abec_451a_a6bf_a03335045383" name="KM53" value="0.02"/>

<parameter id="mwe110663f_33e9_415c_96f8_04e92e007bb4" name="V_Sink_GAP" value="9e-005"/>

<parameter id="mw395deb93_b80e_4154_b71a_add84953daaf" name="K_Sink_GAP" value="0.1"/>

<parameter id="mw2a519375_b634_461c_a4e7_2205e166b3ce" name="Kgap" value="1"/>

<parameter id="mw02659a3e_942b_4113_928b_885d34341771" name="Knadpp" value="2"/>

<parameter id="mw25dcc228_99f4_4d62_badc_00067a7db23a" name="Kpga" value="0.357"/>

<parameter id="mwd0f35f82_c657_4a53_9ac4_606e79cebb96" name="Vgap_dehyd" value="1.24"/>

<parameter id="mw31fc107e_644a_475e_aeff_2b016202c816" name="knadph" value="0.927"/>

<parameter id="mw1a31f571_e225_4d2d_b672_bb6209080e70" name="Keq_gap_dehyd" value="0.471"/>

<parameter id="mw04a2d768_b6a0_4a14_8c35_7cee82a7a308" name="Kgap_beta" value="1"/>

<parameter id="mwa177ed29_1ff2_4ddf_aa7c_8cc4c08a1a2d" name="Knadpp_beta" value="1"/>

<parameter id="mw18e6c815_3a2d_4abe_8f8c_96b098a1ffe4" name="Kpga_beta" value="1"/>

<parameter id="mw03434a4a_4ed9_491d_bb10_f39e62b3acb4" name="Vgap_dehyd_beta" value="0"/>

<parameter id="mw0d8a79b9_2494_4bbb_8324_70f0b2e27188" name="knadph_beta" value="1"/>
```

```
<parameter id="mwe6b619dd_229f_4afa_af14_be3a25b0e262" name="KM61" value="0.033"/>
<parameter id="mwd59eae5a_ff1f_4351_922c_d25f34ca05ac" name="KM72" value="0.1"/>
<parameter id="mwd037be1a_7ea7_4d03_b00e_8f358afc43f0" name="KM73" value="0.1"/>
<parameter id="mw6990314a_e646_4a51_b298_7b53a5a3ca3b" name="KM71" value="0.1"/>
<parameter id="mwd32ff45c_71b0_4916_8ffc_7683019b9311" name="KM74" value="0.1"/>
<parameter id="mwa6f625ba_c9e6_40e4_bf00_d59146ed8f80" name="KM82" value="0.2"/>
<parameter id="mw28855de3_91da_4e09_b2c9_c0eb4b719af3" name="KM81" value="0.4"/>
<parameter id="mwf44df5f6_9e91_4081_a77d_911fe8867849" name="KE8" value="0.83"/>
<parameter id="mw2527d5af_e782_46fb_9b2c_875866d6ecd5" name="KM9" value="0.05"/>
<parameter id="mw075f4d45_ceca_4aad_bc29_89b6915e46f7" name="KE10" value="1.54"/>
<parameter id="mwf8558c32_d374_4352_b837_54ce36f88a5e" name="KM71a" value="0.616"/>
<parameter id="mwf187cf00_32ca_422a_9134_ffd278f81118" name="KM101" value="0.118"/>
<parameter id="mw94fab6bb_d1d8_4344_ac40_d9ea47173826" name="KE11" value="2.35"/>
<parameter id="mw46a0d4e0_a24e_4d84_ac9d_1d098ed93c0b" name="KE12" value="0.32"/>
<parameter id="mwc1d6540f_78d6_40ae_8e94_d6db3d674a6e" name="KM132" value="0.05"/>
<parameter id="mw1862dfa3_5857_4ef7_82ea_209a042715a4" name="K_GOA" value="0.1"/>
<parameter id="mw016a3190_5878_41d4_8022_96365a5cde42" name="V_GLY_syn"
value="0.1"/>
<parameter id="mwdec52954_3ec3_416b_8d1c_d8191ee0052a" name="KM11"
value="0.0115"/>
<parameter id="mw67c2b3cc_db05_4a74_97af_8d7314b6868b" name="KM12" value="0.222"/>
<parameter id="mwa4c7bb69_e0a6_4881_b8c4_8bb392a17249" name="KI11" value="0.84"/>
<parameter id="mwa496f448_c225_4611_af1e_f7f3dfa7977c" name="KI14" value="0.9"/>
<parameter id="mw49a330a3_2804_4b34_bed5_55979f9f820c" name="KM13" value="0.02"/>
<parameter id="mwaab87003_70c0_4690_a563_726e460f9c64" name="KI12" value="0.04"/>
<parameter id="mw8642dd0a_5a0f_45a5_b0ca_d246e508136b" name="KI13" value="0.075"/>
<parameter id="mw7aeaf567_e744_423f_b2c4_3aeb34386eb6" name="KI15" value="0.07"/>
<parameter id="mw33c77066_cf3c_44c1_9e75_e85e2c99a3fd" name="KM21" value="0.24"/>
```

```xml
<parameter id="mwfd3ba144_51dd_4c75_b0e1_8926ab5477e8" name="KM22" value="0.39"/>
<parameter id="mwb70b9188_7453_4609_ad48_be4bf69f87e4" name="KM23" value="0.23"/>
<parameter id="mwbd9ba0b8_46b5_43a7_894a_1e0a7450c854" name="KE2" value="0.000619"/>
<parameter id="mw701b6fb7_2279_4661_a2d5_e1e2e9f47203" name="KM31" value="0.004"/>
<parameter id="mw9ea5d254_32bc_4a37_aa1f_f4b94da8eadb" name="KM32" value="0.1"/>
<parameter id="mw9f468e68_fa4c_4260_a5af_3b6182abcb92" name="KI62" value="12"/>
<parameter id="mwa90c1c5f_dcc6_48a5_a99f_ce08e7c2c91b" name="KI61" value="0.7"/>
<parameter id="mw118d7663_7b18_410d_8ca7_fc168407ba10" name="KI9" value="12"/>
<parameter id="mwe195dc18_fc3c_4329_905f_5bb2fc3efe19" name="KM104" value="0.54"/>
<parameter id="mwb61a7f15_d0aa_4449_85bb_2cdf4a397051" name="KM103" value="0.09"/>
<parameter id="mwe1915e65_ff53_471a_9d2c_b92b8d75f287" name="KM102" value="0.09"/>
<parameter id="mw7d11f964_6e54_4584_8c91_1808e4e850fd" name="KM72b" value="0.27"/>
<parameter id="mw814665c4_d157_4663_a75e_af6f8f891fb0" name="KI135" value="0.4"/>
<parameter id="mwd947eee9_9a97_4305_a38f_5d0342490834" name="KI131" value="2"/>
<parameter id="mw16745cb2_2a9e_48f6_8cef_80e44ec67119" name="KI134" value="2.5"/>
<parameter id="mw6bb6ce48_f2df_4db7_abe8_95a611a6672b" name="KI133" value="4"/>
<parameter id="mw0b12ed4b_ffff_47bd_a925_140813e42732" name="KI132" value="0.7"/>
<parameter id="mw9c902b95_94d4_4dff_912f_eb93bd74d440" name="KM131" value="0.05"/>
<parameter id="mw52505a99_c3c7_4c4c_8a62_b891bfab4dd8" name="K_Sink_G6P" value="2"/>
<parameter id="mw2fcbcb1c_c103_48bb_86ed_9229a27de6ed" name="V_Sink_G6P" value="0.0035"/>
<parameter id="mwa27b7723_ab20_4324_a886_f5c73588ac4a" name="KM_PKET" value="2"/>
<parameter id="mw72f442a2_e01a_44d3_b8bf_9d223245f350" name="V_PKET" value="0.015"/>
<parameter id="mwc1298b32_b9e2_4ab0_8087_3acdea483cdc" name="K_Sink_AceP" value="1"/>
<parameter id="mwd7c8b802_9d0c_4a81_9c65_1a50be7968bb" name="V_Sink_AceP" value="1"/>
```

```xml
<parameter id="mw6f022b45_ad82_4bfc_bbbd_0855ddf10ecf" name="K_OXA1" value="2"/>

<parameter id="mwcd39d210_482b_47b5_b954_07cc17e31155" name="K_OXA2" value="5"/>

<parameter id="mw57949b29_e69c_444c_8d58_1ddda96d13c5" name="KM_PKET_beta" value="0.2"/>

<parameter id="mw86416738_ea27_41b8_adf9_4d3b5a320d97" name="V_PKET_beta" value="0.0005"/>

<parameter id="mw9d451500_8215_4269_83be_e237d42f0aab" name="KM_PFK_beta" value="2.5"/>

<parameter id="mw61b0fed3_e790_4af3_a1e7_65c1646248bf" name="V_PFK_beta" value="0.07"/>

<parameter id="mwd2bb54b2_f070_41d0_a500_9b196833f57a" name="KI61_beta" value="6"/>

<parameter id="mwdb1e1973_5df2_4a29_a277_64c677c902b4" name="KM61_beta" value="0.3"/>

<parameter id="mw13a07b09_9a67_4561_b3db_8e39442e5d53" name="V6_beta" value="0.04"/>

<parameter id="mw9168b04c_6d3a_4f60_8906_5b5293d6a01e" name="Kmp_V11_alpha" value="2"/>

<parameter id="mwf2185be5_b4b0_4bf4_a6d3_0a758e4fbcca" name="Kms_V11_alpha" value="0.1"/>

<parameter id="mw332683df_4959_4366_94ef_2b7108d780dc" name="Kmp_V11_beta" value="0.05"/>

<parameter id="mw8da43b9c_22d4_4c48_bcc7_0bb2a83b77f6" name="Kms_V11_beta" value="2"/>

<parameter id="mw15a7ce92_eb88_4188_966c_aad7e9d8ea7e" name="V11_beta" value="0.4"/>

<parameter id="mw80012d61_bf17_467d_8029_8785e8b66833" name="Kmp_PPP1" value="0.1"/>

<parameter id="mw212994b8_ca26_4fbc_a13e_bb054a312812" name="Kms_PPP1" value="2"/>

<parameter id="mw84e3d5fb_d3f5_4a42_a963_2d0e4de0c9fa" name="V_PPP1" value="0.54"/>

<parameter id="mwbf2d484b_0c51_4597_84e7_a9d72a8ed607" name="Kms_nadpp_ppp2" value="1"/>

<parameter id="mw9aad6b3a_ddc4_4aa7_af1d_9c6f98a72a80" name="Kms_p6g_ppp2" value="1.2"/>
```

```
      <parameter id="mwacee8ded_69a8_4141_87f2_2622f06daa29" name="V_PPP2" value="0.3"/>
      <parameter id="mw4d3fe418_9692_4308_a1c1_64b6e3b6ad82" name="K_Sink_E4P"
value="0.1"/>
      <parameter id="mwe871df2e_a7b7_49c5_8a95_809524380cbd" name="K_Sink_Ri5P"
value="0.1"/>
      <parameter id="mw42e7bfb9_db8f_4b73_bd5b_4f46a35e36a0" name="KI1121" value="94"/>
      <parameter id="mw43a4fc8c_e159_44c4_aca5_0de4d39f2892" name="KI1122" value="2.55"/>
      <parameter id="mw2026f64b_36e7_4da9_8f99_4179a8aa5a0f" name="Km112c" value="0.5"/>
      <parameter id="mwe7492e74_1fd5_412c_9bd7_c33659cbb9ae" name="V_PP2c"
value="0.001"/>
    </listOfParameters>
    <listOfRules>
      <assignmentRule metaid="repeatedAssignment_mw48d91844_76d7_4f00_b1f9_dbedf937d63f"
variable="mw110e8daa_fa0e_409b_af62_9f01d755a8e3">
        <math xmlns="http://www.w3.org/1998/Math/MathML">
          <apply>
            <divide/>
            <ci> species_7 </ci>
            <apply>
              <plus/>
              <ci> species_7 </ci>
              <ci> species_4 </ci>
            </apply>
          </apply>
        </math>
      </assignmentRule>
      <assignmentRule metaid="repeatedAssignment_mw1babcdad_d925_420b_81f6_ebb44bb8be53"
variable="mwda7da32a_797a_4dbc_9e6a_c9317b3838b5">
        <math xmlns="http://www.w3.org/1998/Math/MathML">
```

<apply>

<plus/>

<ci> mwa8568197_97f2_4c67_8727_f8d9ed6895e3 </ci>

<ci> mw8369442e_7e34_45ca_89a9_7f0ab2ab70ee </ci>

<ci> mw09d7964e_16d4_4c3d_a1f5_d98a7ab66891 </ci>

<ci> mw70a9d4b8_2ca3_4692_a159_702d8464b7e7 </ci>

<ci> mwe556cd16_34e2_417a_b945_37061560bfe5 </ci>

<ci> species_23 </ci>

<ci> species_24 </ci>

<ci> species_33 </ci>

<ci> species_34 </ci>

</apply>

</math>

</assignmentRule>

<assignmentRule metaid="repeatedAssignment_mw7ce83324_309f_45ce_b8ae_701c6f34d663" variable="mw7f9b1aa3_a2ab_4207_b81f_4ad133f8ccf8">

<math xmlns="http://www.w3.org/1998/Math/MathML">

<apply>

<divide/>

<ci> species_5 </ci>

<apply>

<plus/>

<ci> species_5 </ci>

<ci> species_11 </ci>

</apply>

</apply>

</math>

</assignmentRule>

```
    <assignmentRule metaid="repeatedAssignment_mwca4fd66d_9ddc_4230_9737_ae88453e6d30"
variable="mw4412eef8_75f9_408b_8ca1_0cd634ded124">
      <math xmlns="http://www.w3.org/1998/Math/MathML">
        <apply>
          <divide/>
          <apply>
            <times/>
            <ci> species_3 </ci>
            <apply>
              <divide/>
              <apply>
                <times/>
                <ci> mwf008a49f_a4ec_49ec_8cdc_30906d544233 </ci>
                <ci> species_1 </ci>
              </apply>
              <apply>
                <plus/>
                <ci> species_1 </ci>
                <apply>
                  <times/>
                  <ci> mwdec52954_3ec3_416b_8d1c_d8191ee0052a </ci>
                  <apply>
                    <plus/>
                    <cn type="integer"> 1 </cn>
                    <apply>
                      <divide/>
                      <ci> species_6 </ci>
                      <ci> mw67c2b3cc_db05_4a74_97af_8d7314b6868b </ci>
```

</apply>

</apply>

</apply>

</apply>

</apply>

</apply>

<apply>

<plus/>

<ci> species_3 </ci>

<apply>

<times/>

<ci> mw49a330a3_2804_4b34_bed5_55979f9f820c </ci>

<apply>

<plus/>

<cn type="integer"> 1 </cn>

<apply>

<divide/>

<ci> species_2 </ci>

<ci> mwa4c7bb69_e0a6_4881_b8c4_8bb392a17249 </ci>

</apply>

<apply>

<divide/>

<ci> species_14 </ci>

<ci> mwaab87003_70c0_4690_a563_726e460f9c64 </ci>

</apply>

<apply>

<divide/>

```
            &lt;ci&gt; species_8 &lt;/ci&gt;
            &lt;ci&gt; mw8642dd0a_5a0f_45a5_b0ca_d246e508136b &lt;/ci&gt;
          &lt;/apply&gt;
          &lt;apply&gt;
            &lt;divide/&gt;
            &lt;ci&gt; species_12 &lt;/ci&gt;
            &lt;ci&gt; mwa496f448_c225_4611_af1e_f7f3dfa7977c &lt;/ci&gt;
          &lt;/apply&gt;
          &lt;apply&gt;
            &lt;divide/&gt;
            &lt;ci&gt; species_5 &lt;/ci&gt;
            &lt;ci&gt; mw7aeaf567_e744_423f_b2c4_3aeb34386eb6 &lt;/ci&gt;
          &lt;/apply&gt;
        &lt;/apply&gt;
      &lt;/apply&gt;
    &lt;/apply&gt;
  &lt;/apply&gt;
&lt;/math&gt;
&lt;/assignmentRule&gt;
&lt;assignmentRule metaid="repeatedAssignment_mw46513e34_7bce_44fb_bf65_e95c442f2c9c" variable="mw5bf04cb0_8acb_4c37_a547_e939d6416759"&gt;
  &lt;math xmlns="http://www.w3.org/1998/Math/MathML"&gt;
    &lt;apply&gt;
      &lt;divide/&gt;
      &lt;apply&gt;
        &lt;times/&gt;
        &lt;ci&gt; mwfe139de1_b141_420e_a4e5_f59c639c6cf0 &lt;/ci&gt;
        &lt;apply&gt;
```

<minus/>
<apply>
<times/>
<ci> species_2 </ci>
<ci> species_7 </ci>
</apply>
<apply>
<divide/>
<apply>
<times/>
<ci> species_9 </ci>
<ci> species_4 </ci>
</apply>
<ci> mwbd9ba0b8_46b5_43a7_894a_1e0a7450c854 </ci>
</apply>
</apply>
</apply>
<apply>
<times/>
<apply>
<plus/>
<ci> species_2 </ci>
<ci> mw33c77066_cf3c_44c1_9e75_e85e2c99a3fd </ci>
</apply>
<apply>
<plus/>
<ci> species_7 </ci>

```
            <apply>
              <times/>
              <ci> mwfd3ba144_51dd_4c75_b0e1_8926ab5477e8 </ci>
              <apply>
                <plus/>
                <cn type="integer"> 1 </cn>
                <apply>
                  <divide/>
                  <ci> species_4 </ci>
                  <ci> mwb70b9188_7453_4609_ad48_be4bf69f87e4 </ci>
                </apply>
              </apply>
            </apply>
          </apply>
        </apply>
      </apply>
    </math>
  </assignmentRule>
  <assignmentRule metaid="repeatedAssignment_mw0d2bcf4d_a89c_4584_b28a_3af29bc061c0"
variable="mwe2947217_6be6_428f_ac4e_28ae24c11d45">
    <math xmlns="http://www.w3.org/1998/Math/MathML">
      <apply>
        <divide/>
        <apply>
          <times/>
          <ci> mwc727e6a6_e76e_44d0_89f4_bede015cb04c </ci>
          <ci> species_9 </ci>
          <ci> species_5 </ci>
```

</apply>
<apply>
<times/>
<apply>
<plus/>
<ci> species_9 </ci>
<ci> mw701b6fb7_2279_4661_a2d5_e1e2e9f47203 </ci>
</apply>
<apply>
<plus/>
<ci> species_5 </ci>
<ci> mw9ea5d254_32bc_4a37_aa1f_f4b94da8eadb </ci>
</apply>
</apply>
</apply>
</math>
</assignmentRule>
<assignmentRule metaid="repeatedAssignment_mw91c1bad8_ddb2_4b54_9440_497633fe887c" variable="mwac50212d_9880_4c18_b404_443dcf85e43a">
<math xmlns="http://www.w3.org/1998/Math/MathML">
<apply>
<divide/>
<apply>
<times/>
<ci> mw8c8ad41e_6d63_473b_960b_322d5feb6f84 </ci>
<apply>
<minus/>
<ci> species_10 </ci>

```
          <apply>
            <divide/>
            <ci> species_13 </ci>
            <ci> mwdde5b27b_4aa1_42aa_b292_6613ea7584a3 </ci>
          </apply>
        </apply>
      </apply>
      <apply>
        <plus/>
        <ci> species_10 </ci>
        <ci> species_13 </ci>
      </apply>
    </apply>
  </math>
</assignmentRule>
<assignmentRule metaid="repeatedAssignment_mwf718ba1f_2460_4130_a6f4_199dbcd27a04" variable="mwdad3e574_e8ae_4a2a_bc5b_5d2a8592dffc">
  <math xmlns="http://www.w3.org/1998/Math/MathML">
    <apply>
      <divide/>
      <apply>
        <times/>
        <ci> mwdbdd129f_0428_4a42_8427_cf18a0b5568c </ci>
        <apply>
          <minus/>
          <apply>
            <times/>
            <ci> species_10 </ci>
```

<ci> species_13 </ci>

</apply>

<apply>

<divide/>

<ci> species_14 </ci>

<ci> mw245d5cf2_30db_40c3_b1a7_ed389fff8808 </ci>

</apply>

</apply>

</apply>

<apply>

<times/>

<ci> mw39e8b6c4_2ae7_4846_9ac8_e77ee9c7191f </ci>

<ci> mw54c31bc3_16fc_462a_a55e_a766a97769ed </ci>

<apply>

<plus/>

<cn type="integer"> 1 </cn>

<apply>

<divide/>

<ci> species_10 </ci>

<ci> mw39e8b6c4_2ae7_4846_9ac8_e77ee9c7191f </ci>

</apply>

<apply>

<divide/>

<ci> species_13 </ci>

<ci> mw54c31bc3_16fc_462a_a55e_a766a97769ed </ci>

</apply>

<apply>

<divide/>

<ci> species_14 </ci>

<ci> mwbad28d74_abec_451a_a6bf_a03335045383 </ci>

</apply>

<apply>

<divide/>

<apply>

<times/>

<ci> species_10 </ci>

<ci> species_13 </ci>

</apply>

<apply>

<times/>

<ci> mw39e8b6c4_2ae7_4846_9ac8_e77ee9c7191f </ci>

<ci> mw54c31bc3_16fc_462a_a55e_a766a97769ed </ci>

</apply>

</apply>

</apply>

</apply>

</apply>

</math>

</assignmentRule>

<assignmentRule metaid="repeatedAssignment_mw78bd7f23_1644_4b90_b0fb_6a4c3079d5c3" variable="mwc04a96b8_d70f_4bdc_854b_7367baf678c4">

<math xmlns="http://www.w3.org/1998/Math/MathML">

<apply>

<plus/>

<apply>

```
<divide/>
<apply>
  <times/>
  <ci> mw9dda631f_8096_4e56_a670_fdb559f36d12 </ci>
  <ci> species_14 </ci>
</apply>
<apply>
  <plus/>
  <ci> species_14 </ci>
  <apply>
    <times/>
    <ci> mwe6b619dd_229f_4afa_af14_be3a25b0e262 </ci>
    <apply>
      <plus/>
      <cn type="integer"> 1 </cn>
      <apply>
        <divide/>
        <ci> species_15 </ci>
        <ci> mwa90c1c5f_dcc6_48a5_a99f_ce08e7c2c91b </ci>
      </apply>
      <apply>
        <divide/>
        <ci> species_12 </ci>
        <ci> mw9f468e68_fa4c_4260_a5af_3b6182abcb92 </ci>
      </apply>
    </apply>
  </apply>
```

```
  </apply>
</apply>
<apply>
  <divide/>
  <apply>
    <times/>
    <ci> mw13a07b09_9a67_4561_b3db_8e39442e5d53 </ci>
    <ci> species_14 </ci>
  </apply>
  <apply>
    <plus/>
    <ci> species_14 </ci>
    <apply>
      <times/>
      <ci> mwdb1e1973_5df2_4a29_a277_64c677c902b4 </ci>
      <apply>
        <plus/>
        <cn type="integer"> 1 </cn>
        <apply>
          <divide/>
          <ci> species_15 </ci>
          <ci> mwd2bb54b2_f070_41d0_a500_9b196833f57a </ci>
        </apply>
        <apply>
          <divide/>
          <ci> species_12 </ci>
          <ci> mw9f468e68_fa4c_4260_a5af_3b6182abcb92 </ci>
```

</apply>

</apply>

</apply>

</apply>

</apply>

</apply>

</math>

</assignmentRule>

<assignmentRule metaid="repeatedAssignment_mwd0e165e8_210e_4a23_9120_c2a66a94a9bd" variable="mw0ec64103_a8ce_438d_97b6_aecac492a2fb">

<math xmlns="http://www.w3.org/1998/Math/MathML">

<apply>

<divide/>

<apply>

<times/>

<ci> mwea0f726e_b6ff_4cf0_8dba_9db4cc0827be </ci>

<apply>

<minus/>

<apply>

<times/>

<ci> species_15 </ci>

<ci> species_10 </ci>

</apply>

<apply>

<divide/>

<apply>

<times/>

<ci> species_17 </ci>

```
    <ci> species_16 </ci>
   </apply>
   <ci> mw4ec82ea3_67a6_47ad_971a_52a33f180466 </ci>
  </apply>
 </apply>
</apply>
<apply>
 <times/>
 <apply>
  <plus/>
  <ci> species_15 </ci>
  <apply>
   <times/>
   <ci> mwd037be1a_7ea7_4d03_b00e_8f358afc43f0 </ci>
   <apply>
    <plus/>
    <cn type="integer"> 1 </cn>
    <apply>
     <divide/>
     <ci> species_17 </ci>
     <ci> mw6990314a_e646_4a51_b298_7b53a5a3ca3b </ci>
    </apply>
    <apply>
     <divide/>
     <ci> species_16 </ci>
     <ci> mwd59eae5a_ff1f_4351_922c_d25f34ca05ac </ci>
    </apply>
```

```
          </apply>
        </apply>
      </apply>
      <apply>
        <plus/>
        <ci> species_10 </ci>
        <ci> mwd32ff45c_71b0_4916_8ffc_7683019b9311 </ci>
      </apply>
    </apply>
  </apply>
</math>
</assignmentRule>
<assignmentRule metaid="repeatedAssignment_mwbbdf0263_4ad0_4ab1_8bbd_234f5f14a2a9"
variable="mwc594876f_c9b0_4f4b_a245_d024b6f1931a">
  <math xmlns="http://www.w3.org/1998/Math/MathML">
    <apply>
      <divide/>
      <apply>
        <times/>
        <ci> mw33ab4422_ba35_4d2b_9620_d274168c7e1d </ci>
        <apply>
          <minus/>
          <apply>
            <times/>
            <ci> species_13 </ci>
            <ci> species_16 </ci>
          </apply>
          <apply>
```

```
            <divide/>
            <ci> species_8 </ci>
            <ci> mwf44df5f6_9e91_4081_a77d_911fe8867849 </ci>
          </apply>
        </apply>
      </apply>
      <apply>
        <times/>
        <apply>
          <plus/>
          <ci> species_16 </ci>
          <ci> mwa6f625ba_c9e6_40e4_bf00_d59146ed8f80 </ci>
        </apply>
        <apply>
          <plus/>
          <ci> species_13 </ci>
          <ci> mw28855de3_91da_4e09_b2c9_c0eb4b719af3 </ci>
        </apply>
      </apply>
    </apply>
  </math>
</assignmentRule>
<assignmentRule metaid="repeatedAssignment_mwfd935303_85b5_4619_8f29_6ce9dd7d5a54"
variable="mwb8fa5238_b6ab_43ba_914d_8d9609a94211">
  <math xmlns="http://www.w3.org/1998/Math/MathML">
    <apply>
      <divide/>
      <apply>
```

```
            <times/>
            <ci> mw5c6f658c_7714_4fbb_be83_5ef825b3e08b </ci>
            <ci> species_8 </ci>
          </apply>
          <apply>
            <plus/>
            <ci> species_8 </ci>
            <apply>
              <times/>
              <ci> mw2527d5af_e782_46fb_9b2c_875866d6ecd5 </ci>
              <apply>
                <plus/>
                <cn type="integer"> 1 </cn>
                <apply>
                  <divide/>
                  <ci> species_12 </ci>
                  <ci> mw118d7663_7b18_410d_8ca7_fc168407ba10 </ci>
                </apply>
              </apply>
            </apply>
          </apply>
        </apply>
      </math>
    </assignmentRule>
    <assignmentRule metaid="repeatedAssignment_mw32890e00_1f5b_4b23_8928_f95a780ca700"
variable="mwd13e1918_33bb_4d69_9527_a7db921570eb">
      <math xmlns="http://www.w3.org/1998/Math/MathML">
        <apply>
```

```
<divide/>
<apply>
 <times/>
 <ci> mw6e8005dd_31e8_483e_902a_8870307bf558 </ci>
 <apply>
  <minus/>
  <apply>
   <times/>
   <ci> species_10 </ci>
   <ci> species_19 </ci>
  </apply>
  <apply>
   <divide/>
   <apply>
    <times/>
    <ci> species_18 </ci>
    <ci> species_17 </ci>
   </apply>
   <ci> mw075f4d45_ceca_4aad_bc29_89b6915e46f7 </ci>
  </apply>
 </apply>
</apply>
<apply>
 <times/>
 <ci> mwf8558c32_d374_4352_b837_54ce36f88a5e </ci>
 <ci> mwf187cf00_32ca_422a_9134_ffd278f81118 </ci>
 <apply>
```

<plus/>
<cn type="integer"> 1 </cn>
<apply>
<times/>
<apply>
<plus/>
<cn type="integer"> 1 </cn>
<apply>
<divide/>
<ci> species_10 </ci>
<ci> mw7d11f964_6e54_4584_8c91_1808e4e850fd </ci>
</apply>
</apply>
<apply>
<plus/>
<apply>
<divide/>
<ci> species_15 </ci>
<ci> mwe195dc18_fc3c_4329_905f_5bb2fc3efe19 </ci>
</apply>
<apply>
<divide/>
<ci> species_19 </ci>
<ci> mwb61a7f15_d0aa_4449_85bb_2cdf4a397051 </ci>
</apply>
</apply>
</apply>

<apply>
<divide/>
<ci> species_10 </ci>
<ci> mwe1915e65_ff53_471a_9d2c_b92b8d75f287 </ci>
</apply>
<apply>
<divide/>
<apply>
<plus/>
<apply>
<times/>
<ci> species_17 </ci>
<apply>
<plus/>
<cn type="integer"> 1 </cn>
<apply>
<divide/>
<apply>
<plus/>
<ci> species_16 </ci>
<ci> species_18 </ci>
</apply>
<ci> mwf8558c32_d374_4352_b837_54ce36f88a5e </ci>
</apply>
</apply>
</apply>
<ci> species_16 </ci>

<ci> species_18 </ci>
</apply>
<ci> mwf187cf00_32ca_422a_9134_ffd278f81118 </ci>
</apply>
</apply>
</apply>
</apply>
</math>
</assignmentRule>
<assignmentRule metaid="repeatedAssignment_mwce49dbff_e807_40c8_88ca_13e833185c15" variable="mwbe2ee4f3_36d2_4a42_8390_f8082ac79296">
<math xmlns="http://www.w3.org/1998/Math/MathML">
<apply>
<divide/>
<apply>
<times/>
<ci> mw4fbb318e_c400_4af5_8e3f_e80aec639ee3 </ci>
<apply>
<minus/>
<ci> species_18 </ci>
<apply>
<divide/>
<ci> species_20 </ci>
<ci> mw94fab6bb_d1d8_4344_ac40_d9ea47173826 </ci>
</apply>
</apply>
</apply>
<apply>

```
          <plus/>
          <ci> species_18 </ci>
          <ci> species_20 </ci>
        </apply>
      </apply>
    </math>
  </assignmentRule>
  <assignmentRule metaid="repeatedAssignment_mw3972ea92_33f7_4539_a583_eaec1d4a5e27"
variable="mw60c6f424_4d57_49bf_b130_811bec897e0a">
    <math xmlns="http://www.w3.org/1998/Math/MathML">
      <apply>
        <divide/>
        <apply>
          <times/>
          <ci> mw702a7f39_5675_48ad_953a_4bcbbdc8e1a2 </ci>
          <apply>
            <minus/>
            <ci> species_17 </ci>
            <apply>
              <divide/>
              <ci> species_20 </ci>
              <ci> mw46a0d4e0_a24e_4d84_ac9d_1d098ed93c0b </ci>
            </apply>
          </apply>
        </apply>
        <apply>
          <plus/>
          <ci> species_17 </ci>
```

```
          <ci> species_20 </ci>
        </apply>
      </apply>
    </math>
  </assignmentRule>
  <assignmentRule metaid="repeatedAssignment_mw445b8e5a_e800_4251_8a9b_4a4f77299cea"
variable="mw96deb747_ef33_4a50_b36e_145767b53cf7">
    <math xmlns="http://www.w3.org/1998/Math/MathML">
      <apply>
        <divide/>
        <apply>
          <times/>
          <ci> mwd486ae05_3de1_42aa_9584_2edc458d0c59 </ci>
          <ci> species_7 </ci>
          <ci> species_20 </ci>
        </apply>
        <apply>
          <times/>
          <apply>
            <plus/>
            <apply>
              <times/>
              <ci> species_7 </ci>
              <apply>
                <plus/>
                <cn type="integer"> 1 </cn>
                <apply>
                  <divide/>
```

<ci> species_4 </ci>
<ci> mw16745cb2_2a9e_48f6_8cef_80e44ec67119 </ci>
</apply>
</apply>
</apply>
<apply>
<times/>
<ci> mwc1d6540f_78d6_40ae_8e94_d6db3d674a6e </ci>
<apply>
<plus/>
<cn type="integer"> 1 </cn>
<apply>
<divide/>
<ci> species_4 </ci>
<ci> mw814665c4_d157_4663_a75e_af6f8f891fb0 </ci>
</apply>
</apply>
</apply>
</apply>
<apply>
<plus/>
<ci> species_20 </ci>
<apply>
<times/>
<ci> mw9c902b95_94d4_4dff_912f_eb93bd74d440 </ci>
<apply>
<plus/>

```
            <cn type="integer"> 1 </cn>
            <apply>
              <divide/>
              <ci> species_2 </ci>
              <ci> mwd947eee9_9a97_4305_a38f_5d0342490834 </ci>
            </apply>
            <apply>
              <divide/>
              <ci> species_3 </ci>
              <ci> mw0b12ed4b_ffff_47bd_a925_140813e42732 </ci>
            </apply>
            <apply>
              <divide/>
              <ci> species_12 </ci>
              <ci> mw6bb6ce48_f2df_4db7_abe8_95a611a6672b </ci>
            </apply>
          </apply>
        </apply>
      </apply>
    </apply>
  </apply>
</math>
</assignmentRule>
<assignmentRule metaid="repeatedAssignment_mw28551e86_9575_4118_ba85_f632156e900b" variable="mw42c5ea06_edf8_46bd_b262_f9db84921975">
  <math xmlns="http://www.w3.org/1998/Math/MathML">
    <apply>
      <divide/>
```

```
          <apply>
            <times/>
            <ci> mwfb9d0842_5ad1_42a2_b44c_12cfeaa36962 </ci>
            <ci> species_15 </ci>
          </apply>
          <apply>
            <plus/>
            <ci> species_15 </ci>
            <ci> mwc59d3254_509c_411a_b656_9e80ac0a4d4a </ci>
          </apply>
        </apply>
      </math>
    </assignmentRule>
    <assignmentRule metaid="repeatedAssignment_mw46ccbf09_9a03_480b_aa20_e3247fe474ce"
variable="mw10231dd9_d13a_4822_a477_0b190284526c">
      <math xmlns="http://www.w3.org/1998/Math/MathML">
        <apply>
          <plus/>
          <apply>
            <divide/>
            <apply>
              <times/>
              <ci> mw4f571ae6_7040_40c6_b098_4ac7cf9ee24d </ci>
              <apply>
                <minus/>
                <ci> species_2 </ci>
                <apply>
                  <divide/>
```

```
        <ci> species_35 </ci>
        <ci> mw917c32b0_7a3f_4744_ac14_2e3d15da96f6 </ci>
      </apply>
    </apply>
  </apply>
  <apply>
    <times/>
    <ci> mw742fbec5_509e_42be_b7a8_12b9eeb49314 </ci>
    <apply>
      <plus/>
      <cn type="integer"> 1 </cn>
      <apply>
        <divide/>
        <ci> species_2 </ci>
        <ci> mw742fbec5_509e_42be_b7a8_12b9eeb49314 </ci>
      </apply>
      <apply>
        <divide/>
        <ci> species_35 </ci>
        <ci> mw231dd6ca_4db0_4cdb_adc8_72349abdba07 </ci>
      </apply>
    </apply>
  </apply>
</apply>
<apply>
  <divide/>
  <apply>
```

```
  <times/>
  <ci> mw2ec1703a_729a_4b49_b4dd_4241b0d73907 </ci>
  <apply>
    <minus/>
    <ci> species_2 </ci>
    <apply>
      <divide/>
      <ci> species_35 </ci>
      <ci> mw917c32b0_7a3f_4744_ac14_2e3d15da96f6 </ci>
    </apply>
  </apply>
</apply>
<apply>
  <times/>
  <ci> mwb99ebe97_7d91_406c_8b12_a56b2c939ff4 </ci>
  <apply>
    <plus/>
    <cn type="integer"> 1 </cn>
    <apply>
      <divide/>
      <ci> species_2 </ci>
      <ci> mwb99ebe97_7d91_406c_8b12_a56b2c939ff4 </ci>
    </apply>
    <apply>
      <divide/>
      <ci> species_35 </ci>
      <ci> mw23fae7dc_7d12_4fb0_98e2_c611e44007e1 </ci>
```

```
      </apply>
    </apply>
  </apply>
</apply>
<apply>
  <divide/>
  <apply>
    <times/>
    <ci> mwa1879047_3c26_45c1_8b2a_137433500cde </ci>
    <apply>
      <minus/>
      <ci> species_2 </ci>
      <apply>
        <divide/>
        <ci> species_35 </ci>
        <ci> mw917c32b0_7a3f_4744_ac14_2e3d15da96f6 </ci>
      </apply>
    </apply>
  </apply>
  <apply>
    <times/>
    <ci> mw6205c3fd_ecf9_4b2b_98f1_dff68729cd03 </ci>
    <apply>
      <plus/>
      <cn type="integer"> 1 </cn>
      <apply>
        <divide/>
```

```
          <ci> species_2 </ci>
          <ci> mw6205c3fd_ecf9_4b2b_98f1_dff68729cd03 </ci>
         </apply>
         <apply>
          <divide/>
          <ci> species_35 </ci>
          <ci> mw23f5979c_8188_4521_9629_6469c95e42d6 </ci>
         </apply>
        </apply>
       </apply>
      </apply>
     </apply>
    </math>
   </assignmentRule>
   <assignmentRule metaid="repeatedAssignment_mwa3220e90_eda8_4345_8304_6f586941e318"
variable="mw0e384600_a904_40dc_b3a6_de20a7ae04b8">
    <math xmlns="http://www.w3.org/1998/Math/MathML">
     <apply>
      <divide/>
      <apply>
       <times/>
       <ci> mw68a26bf2_520b_4b35_90e0_511ec4bbc859 </ci>
       <apply>
        <minus/>
        <ci> species_35 </ci>
        <apply>
         <divide/>
         <ci> species_32 </ci>
```

```
<ci> mwdacf4f8b_f1db_480a_aabf_ff89dc90e087 </ci>
</apply>
</apply>
</apply>
<apply>
<times/>
<ci> mwe806711a_3cbc_4d66_a530_d7ae4e673eea </ci>
<apply>
<plus/>
<cn type="integer"> 1 </cn>
<apply>
<divide/>
<ci> species_35 </ci>
<ci> mwe806711a_3cbc_4d66_a530_d7ae4e673eea </ci>
</apply>
<apply>
<divide/>
<ci> species_32 </ci>
<ci> mwdee0f9ac_28b4_444f_af80_30c299f78093 </ci>
</apply>
</apply>
</apply>
</apply>
</math>
</assignmentRule>
<assignmentRule metaid="repeatedAssignment_mw852003bb_7c32_4815_8d4c_3d524e9f72e9" variable="mwaecebc73_e8e8_4aa8_a5c9_9d2295bf77d4">
<math xmlns="http://www.w3.org/1998/Math/MathML">
```

```
<apply>
 <plus/>
 <apply>
  <divide/>
  <apply>
   <times/>
   <ci> mwd0f35f82_c657_4a53_9ac4_606e79cebb96 </ci>
   <apply>
    <minus/>
    <apply>
     <times/>
     <ci> species_10 </ci>
     <ci> species_11 </ci>
    </apply>
    <apply>
     <times/>
     <ci> mw1a31f571_e225_4d2d_b672_bb6209080e70 </ci>
     <ci> species_2 </ci>
     <ci> species_5 </ci>
    </apply>
   </apply>
  </apply>
  <apply>
   <times/>
   <apply>
    <plus/>
    <cn type="integer"> 1 </cn>
```

```
      <apply>
        <divide/>
        <ci> species_10 </ci>
        <ci> mw2a519375_b634_461c_a4e7_2205e166b3ce </ci>
      </apply>
      <apply>
        <divide/>
        <ci> species_2 </ci>
        <ci> mw25dcc228_99f4_4d62_badc_00067a7db23a </ci>
      </apply>
    </apply>
    <apply>
      <plus/>
      <cn type="integer"> 1 </cn>
      <apply>
        <divide/>
        <ci> species_11 </ci>
        <ci> mw02659a3e_942b_4113_928b_885d34341771 </ci>
      </apply>
      <apply>
        <divide/>
        <ci> species_5 </ci>
        <ci> mw31fc107e_644a_475e_aeff_2b016202c816 </ci>
      </apply>
    </apply>
  </apply>
</apply>
```

```
<apply>
  <divide/>
  <apply>
    <times/>
    <ci> mw03434a4a_4ed9_491d_bb10_f39e62b3acb4 </ci>
    <apply>
      <minus/>
      <apply>
        <times/>
        <ci> species_10 </ci>
        <ci> species_11 </ci>
      </apply>
      <apply>
        <times/>
        <ci> mw1a31f571_e225_4d2d_b672_bb6209080e70 </ci>
        <ci> species_2 </ci>
        <ci> species_5 </ci>
      </apply>
    </apply>
  </apply>
  <apply>
    <times/>
    <apply>
      <plus/>
      <cn type="integer"> 1 </cn>
      <apply>
        <divide/>
```

```
          <ci> species_10 </ci>
          <ci> mw04a2d768_b6a0_4a14_8c35_7cee82a7a308 </ci>
        </apply>
        <apply>
          <divide/>
          <ci> species_2 </ci>
          <ci> mw18e6c815_3a2d_4abe_8f8c_96b098a1ffe4 </ci>
        </apply>
      </apply>
      <apply>
        <plus/>
        <cn type="integer"> 1 </cn>
        <apply>
          <divide/>
          <ci> species_11 </ci>
          <ci> mwa177ed29_1ff2_4ddf_aa7c_8cc4c08a1a2d </ci>
        </apply>
        <apply>
          <divide/>
          <ci> species_5 </ci>
          <ci> mw0d8a79b9_2494_4bbb_8324_70f0b2e27188 </ci>
        </apply>
      </apply>
    </apply>
  </apply>
 </apply>
</math>
```

```
      </assignmentRule>
      <assignmentRule metaid="repeatedAssignment_mwed7d8988_ccb0_4ac6_ad57_3713827b2915"
variable="mwd008246b_4ac0_4550_9588_64a1e8664cd1">
        <math xmlns="http://www.w3.org/1998/Math/MathML">
          <apply>
            <divide/>
            <apply>
              <times/>
              <ci> mw5723da7e_7ee9_4b45_b397_c4fad0251fa2 </ci>
              <ci> species_32 </ci>
            </apply>
            <apply>
              <plus/>
              <ci> mw70b2b9c1_0904_4851_aee4_8526d26a4c7f </ci>
              <ci> species_32 </ci>
            </apply>
          </apply>
        </math>
      </assignmentRule>
      <assignmentRule
metaid="repeatedAssignment_mw8d013e69_5904_4483_a44b_5579e7810e9b"
variable="mwf7b66b1f_77b0_4024_99e5_fb37fb0ab929">
        <math xmlns="http://www.w3.org/1998/Math/MathML">
          <apply>
            <divide/>
            <apply>
              <times/>
              <ci> mw2fcbcb1c_c103_48bb_86ed_9229a27de6ed </ci>
              <ci> species_21 </ci>
```

```
        &lt;/apply&gt;
        &lt;apply&gt;
          &lt;plus/&gt;
          &lt;ci&gt; mw52505a99_c3c7_4c4c_8a62_b891bfab4dd8 &lt;/ci&gt;
          &lt;ci&gt; species_21 &lt;/ci&gt;
        &lt;/apply&gt;
      &lt;/apply&gt;
    &lt;/math&gt;
  &lt;/assignmentRule&gt;
  &lt;assignmentRule metaid="repeatedAssignment_mw0393ec44_48a7_474b_9e3a_603d01dd17c3" variable="mw6eb33847_58dd_4306_8ae9_7d68bb2a5612"&gt;
    &lt;math xmlns="http://www.w3.org/1998/Math/MathML"&gt;
      &lt;apply&gt;
        &lt;divide/&gt;
        &lt;apply&gt;
          &lt;times/&gt;
          &lt;ci&gt; mwe110663f_33e9_415c_96f8_04e92e007bb4 &lt;/ci&gt;
          &lt;ci&gt; species_10 &lt;/ci&gt;
        &lt;/apply&gt;
        &lt;apply&gt;
          &lt;plus/&gt;
          &lt;ci&gt; mw395deb93_b80e_4154_b71a_add84953daaf &lt;/ci&gt;
          &lt;ci&gt; species_10 &lt;/ci&gt;
        &lt;/apply&gt;
      &lt;/apply&gt;
    &lt;/math&gt;
  &lt;/assignmentRule&gt;
```

```
<assignmentRule metaid="repeatedAssignment_mwc9e36a25_0dfd_4cc2_b0de_8637fa3ef59c" variable="mw4a42b5e7_485f_4d6b_a4ed_e490759e5f26">
 <math xmlns="http://www.w3.org/1998/Math/MathML">
  <apply>
   <divide/>
   <apply>
    <times/>
    <ci> mw715a0d4a_6423_4755_a1b5_7d9e5b1852a2 </ci>
    <ci> species_16 </ci>
   </apply>
   <apply>
    <plus/>
    <ci> mw4d3fe418_9692_4308_a1c1_64b6e3b6ad82 </ci>
    <ci> species_16 </ci>
   </apply>
  </apply>
 </math>
</assignmentRule>
<assignmentRule metaid="repeatedAssignment_mwbeb83c0b_9d75_4760_9310_bab51ec60c97" variable="mw3128a56f_9005_484d_92b7_1b5e45b577c6">
 <math xmlns="http://www.w3.org/1998/Math/MathML">
  <apply>
   <divide/>
   <apply>
    <times/>
    <ci> mw6caa1525_e69b_4ab2_b8c7_2b7deeb5ee58 </ci>
    <ci> species_18 </ci>
   </apply>
```

```
          <apply>
            <plus/>
            <ci> mwe871df2e_a7b7_49c5_8a95_809524380cbd </ci>
            <ci> species_18 </ci>
          </apply>
        </apply>
      </math>
    </assignmentRule>
    <assignmentRule metaid="repeatedAssignment_mwb001e092_570f_4ac1_926a_98c5c204f23d" variable="mwca272a09_38a7_4416_a781_d64c8e945cc4">
      <math xmlns="http://www.w3.org/1998/Math/MathML">
        <apply>
          <divide/>
          <apply>
            <times/>
            <ci> mw72f442a2_e01a_44d3_b8bf_9d223245f350 </ci>
            <ci> species_15 </ci>
          </apply>
          <apply>
            <plus/>
            <ci> species_15 </ci>
            <ci> mwa27b7723_ab20_4324_a886_f5c73588ac4a </ci>
          </apply>
        </apply>
      </math>
    </assignmentRule>
    <assignmentRule metaid="repeatedAssignment_mwc6009a2d_977f_4bcd_995b_52321ed9adc5" variable="mwf41678a2_15b0_484e_b056_0c8a195fed4d">
```

```
<math xmlns="http://www.w3.org/1998/Math/MathML">
  <apply>
    <divide/>
    <apply>
      <times/>
      <ci> mw84e3d5fb_d3f5_4a42_a963_2d0e4de0c9fa </ci>
      <ci> species_21 </ci>
    </apply>
    <apply>
      <times/>
      <ci> mw212994b8_ca26_4fbc_a13e_bb054a312812 </ci>
      <apply>
        <plus/>
        <cn type="integer"> 1 </cn>
        <apply>
          <divide/>
          <ci> species_21 </ci>
          <ci> mw212994b8_ca26_4fbc_a13e_bb054a312812 </ci>
        </apply>
        <apply>
          <divide/>
          <ci> mw3447e887_c5e2_4271_9e5f_50c309bc8dc3 </ci>
          <ci> mw80012d61_bf17_467d_8029_8785e8b66833 </ci>
        </apply>
      </apply>
    </apply>
  </apply>
```

```
      &lt;/math&gt;
     &lt;/assignmentRule&gt;
    &lt;/listOfRules&gt;
    &lt;listOfReactions&gt;
     &lt;reaction id="reaction_1" name="CC_1 (RuBisCO)" reversible="false" fast="false"&gt;
      &lt;annotation&gt;
       &lt;COPASI xmlns="http://www.copasi.org/static/sbml"&gt;
        <rdf:RDF xmlns:dcterms="http://purl.org/dc/terms/"
xmlns:rdf="http://www.w3.org/1999/02/22-rdf-syntax-ns#">
         &lt;rdf:Description rdf:about="#COPASI49"&gt;
          &lt;dcterms:created&gt;
           &lt;rdf:Description&gt;
            &lt;dcterms:W3CDTF&gt;2010-04-14T14:47:32Z&lt;/dcterms:W3CDTF&gt;
           &lt;/rdf:Description&gt;
          &lt;/dcterms:created&gt;
         &lt;/rdf:Description&gt;
        &lt;/rdf:RDF&gt;
       &lt;/COPASI&gt;
      &lt;/annotation&gt;
      &lt;listOfReactants&gt;
       &lt;speciesReference species="species_3"/&gt;
       &lt;speciesReference species="species_1"/&gt;
       &lt;speciesReference species="species_6"/&gt;
       &lt;speciesReference species="species_14"/&gt;
       &lt;speciesReference species="species_8"/&gt;
       &lt;speciesReference species="species_12"/&gt;
       &lt;speciesReference species="species_5"/&gt;
      &lt;/listOfReactants&gt;
```

```
<listOfProducts>
  <speciesReference species="species_2" stoichiometry="2"/>
  <speciesReference species="species_6"/>
  <speciesReference species="species_14"/>
  <speciesReference species="species_8"/>
  <speciesReference species="species_12"/>
  <speciesReference species="species_5"/>
</listOfProducts>
<kineticLaw>
  <math xmlns="http://www.w3.org/1998/Math/MathML">
    <apply>
      <divide/>
      <apply>
        <times/>
        <ci> species_3 </ci>
        <apply>
          <divide/>
          <apply>
            <times/>
            <ci> mwf008a49f_a4ec_49ec_8cdc_30906d544233 </ci>
            <cn> 0.9606 </cn>
            <ci> species_1 </ci>
          </apply>
          <apply>
            <plus/>
            <ci> species_1 </ci>
            <apply>
```

```
        <times/>
        <ci> mwdec52954_3ec3_416b_8d1c_d8191ee0052a </ci>
        <apply>
          <plus/>
          <cn type="integer"> 1 </cn>
          <apply>
            <divide/>
            <ci> species_6 </ci>
            <ci> mw67c2b3cc_db05_4a74_97af_8d7314b6868b </ci>
          </apply>
        </apply>
      </apply>
    </apply>
  </apply>
</apply>
<apply>
  <plus/>
  <ci> species_3 </ci>
  <apply>
    <times/>
    <ci> mw49a330a3_2804_4b34_bed5_55979f9f820c </ci>
    <apply>
      <plus/>
      <cn type="integer"> 1 </cn>
      <apply>
        <divide/>
        <ci> species_2 </ci>
```

<ci> mwa4c7bb69_e0a6_4881_b8c4_8bb392a17249 </ci>
</apply>
<apply>
<divide/>
<ci> species_14 </ci>
<ci> mwaab87003_70c0_4690_a563_726e460f9c64 </ci>
</apply>
<apply>
<divide/>
<ci> species_8 </ci>
<ci> mw8642dd0a_5a0f_45a5_b0ca_d246e508136b </ci>
</apply>
<apply>
<divide/>
<ci> species_12 </ci>
<ci> mwa496f448_c225_4611_af1e_f7f3dfa7977c </ci>
</apply>
<apply>
<divide/>
<ci> species_5 </ci>
<ci> mw7aeaf567_e744_423f_b2c4_3aeb34386eb6 </ci>
</apply>
</apply>
</apply>
</apply>
</apply>
</math>

```
        </kineticLaw>
      </reaction>
      <reaction id="reaction_2" name="CC_2 (phosphoglycerate kinase)" reversible="false" fast="false">
        <notes>
          <body xmlns="http://www.w3.org/1999/xhtml">
            <pre>Vmax sn&#xeD;&#x17e;eno z 10.3 na 2</pre>
          </body>
        </notes>
        <annotation>
          <COPASI xmlns="http://www.copasi.org/static/sbml">
            <rdf:RDF xmlns:dcterms="http://purl.org/dc/terms/" xmlns:rdf="http://www.w3.org/1999/02/22-rdf-syntax-ns#">
              <rdf:Description rdf:about="#COPASI50">
                <dcterms:created>
                  <rdf:Description>
                    <dcterms:W3CDTF>2010-04-14T15:57:26Z</dcterms:W3CDTF>
                  </rdf:Description>
                </dcterms:created>
              </rdf:Description>
            </rdf:RDF>
          </COPASI>
        </annotation>
        <listOfReactants>
          <speciesReference species="species_2"/>
          <speciesReference species="species_7"/>
        </listOfReactants>
        <listOfProducts>
```

```xml
  <speciesReference species="species_9"/>
  <speciesReference species="species_4"/>
</listOfProducts>
<kineticLaw>
  <math xmlns="http://www.w3.org/1998/Math/MathML">
    <apply>
      <divide/>
      <apply>
        <times/>
        <ci> mwfe139de1_b141_420e_a4e5_f59c639c6cf0 </ci>
        <cn> 0.86 </cn>
        <apply>
          <minus/>
          <apply>
            <times/>
            <ci> species_2 </ci>
            <ci> species_7 </ci>
          </apply>
          <apply>
            <divide/>
            <apply>
              <times/>
              <ci> species_9 </ci>
              <ci> species_4 </ci>
            </apply>
            <ci> mwbd9ba0b8_46b5_43a7_894a_1e0a7450c854 </ci>
          </apply>
```

```
  </apply>
</apply>
<apply>
  <times/>
  <apply>
    <plus/>
    <ci> species_2 </ci>
    <ci> mw33c77066_cf3c_44c1_9e75_e85e2c99a3fd </ci>
  </apply>
  <apply>
    <plus/>
    <ci> species_7 </ci>
    <apply>
      <times/>
      <ci> mwfd3ba144_51dd_4c75_b0e1_8926ab5477e8 </ci>
      <apply>
        <plus/>
        <cn type="integer"> 1 </cn>
        <apply>
          <divide/>
          <ci> species_4 </ci>
          <ci> mwb70b9188_7453_4609_ad48_be4bf69f87e4 </ci>
        </apply>
      </apply>
    </apply>
  </apply>
</apply>
```

```
          </apply>
        </math>
      </kineticLaw>
    </reaction>
    <reaction id="reaction_3" name="CC_3 (glyceraldehyde 3-phosphate dehydrogenase)"
reversible="false" fast="false">
      <annotation>
        <COPASI xmlns="http://www.copasi.org/static/sbml">
          <rdf:RDF xmlns:dcterms="http://purl.org/dc/terms/"
xmlns:rdf="http://www.w3.org/1999/02/22-rdf-syntax-ns#">
            <rdf:Description rdf:about="#COPASI51">
              <dcterms:created>
                <rdf:Description>
                  <dcterms:W3CDTF>2010-04-14T16:10:42Z</dcterms:W3CDTF>
                </rdf:Description>
              </dcterms:created>
            </rdf:Description>
          </rdf:RDF>
        </COPASI>
      </annotation>
      <listOfReactants>
        <speciesReference species="species_9"/>
        <speciesReference species="species_5"/>
      </listOfReactants>
      <listOfProducts>
        <speciesReference species="species_10"/>
        <speciesReference species="species_11"/>
        <speciesReference species="species_12"/>
```

```
</listOfProducts>
<kineticLaw>
  <math xmlns="http://www.w3.org/1998/Math/MathML">
    <apply>
      <divide/>
      <apply>
        <times/>
        <ci> mwc727e6a6_e76e_44d0_89f4_bede015cb04c </ci>
        <cn> 0.79 </cn>
        <ci> species_9 </ci>
        <ci> species_5 </ci>
      </apply>
      <apply>
        <times/>
        <apply>
          <plus/>
          <ci> species_9 </ci>
          <ci> mw701b6fb7_2279_4661_a2d5_e1e2e9f47203 </ci>
        </apply>
        <apply>
          <plus/>
          <ci> species_5 </ci>
          <ci> mw9ea5d254_32bc_4a37_aa1f_f4b94da8eadb </ci>
        </apply>
      </apply>
    </apply>
  </math>
```

```
        </kineticLaw>
      </reaction>
      <reaction id="reaction_4" name="CC_4 (triose phosphate isomerase)" fast="false">
        <annotation>
          <COPASI xmlns="http://www.copasi.org/static/sbml">
            <rdf:RDF xmlns:dcterms="http://purl.org/dc/terms/"
xmlns:rdf="http://www.w3.org/1999/02/22-rdf-syntax-ns#">
              <rdf:Description rdf:about="#COPASI52">
                <dcterms:created>
                  <rdf:Description>
                    <dcterms:W3CDTF>2010-04-14T16:18:37Z</dcterms:W3CDTF>
                  </rdf:Description>
                </dcterms:created>
              </rdf:Description>
            </rdf:RDF>
          </COPASI>
        </annotation>
        <listOfReactants>
          <speciesReference species="species_10"/>
        </listOfReactants>
        <listOfProducts>
          <speciesReference species="species_13"/>
        </listOfProducts>
        <kineticLaw>
          <math xmlns="http://www.w3.org/1998/Math/MathML">
            <apply>
              <divide/>
              <apply>
```

<times/>
<ci> mw8c8ad41e_6d63_473b_960b_322d5feb6f84 </ci>
<cn> 0.746 </cn>
<apply>
<minus/>
<ci> species_10 </ci>
<apply>
<divide/>
<ci> species_13 </ci>
<ci> mwdde5b27b_4aa1_42aa_b292_6613ea7584a3 </ci>
</apply>
</apply>
</apply>
<apply>
<plus/>
<ci> species_10 </ci>
<ci> species_13 </ci>
</apply>
</apply>
</math>
</kineticLaw>
</reaction>
<reaction id="reaction_5" name="CC_5 (aldolase)" fast="false">
<annotation>
<COPASI xmlns="http://www.copasi.org/static/sbml">
<rdf:RDF xmlns:dcterms="http://purl.org/dc/terms/" xmlns:rdf="http://www.w3.org/1999/02/22-rdf-syntax-ns#">
<rdf:Description rdf:about="#COPASI53">

```
        <dcterms:created>
          <rdf:Description>
            <dcterms:W3CDTF>2010-04-14T16:46:06Z</dcterms:W3CDTF>
          </rdf:Description>
        </dcterms:created>
      </rdf:Description>
    </rdf:RDF>
  </COPASI>
</annotation>
<listOfReactants>
  <speciesReference species="species_10"/>
  <speciesReference species="species_13"/>
</listOfReactants>
<listOfProducts>
  <speciesReference species="species_14"/>
</listOfProducts>
<kineticLaw>
  <math xmlns="http://www.w3.org/1998/Math/MathML">
    <apply>
      <divide/>
      <apply>
        <times/>
        <ci> mwdbdd129f_0428_4a42_8427_cf18a0b5568c </ci>
        <cn> 1.36 </cn>
        <apply>
          <minus/>
          <apply>
```

      <times/>
      <ci> species_10 </ci>
      <ci> species_13 </ci>
    </apply>
    <apply>
      <divide/>
      <ci> species_14 </ci>
      <ci> mw245d5cf2_30db_40c3_b1a7_ed389fff8808 </ci>
    </apply>
  </apply>
</apply>
<apply>
  <times/>
  <ci> mw39e8b6c4_2ae7_4846_9ac8_e77ee9c7191f </ci>
  <ci> mw54c31bc3_16fc_462a_a55e_a766a97769ed </ci>
  <apply>
    <plus/>
    <cn type="integer"> 1 </cn>
    <apply>
      <divide/>
      <ci> species_10 </ci>
      <ci> mw39e8b6c4_2ae7_4846_9ac8_e77ee9c7191f </ci>
    </apply>
    <apply>
      <divide/>
      <ci> species_13 </ci>
      <ci> mw54c31bc3_16fc_462a_a55e_a766a97769ed </ci>

</apply>
<apply>
<divide/>
<ci> species_14 </ci>
<ci> mwbad28d74_abec_451a_a6bf_a03335045383 </ci>
</apply>
<apply>
<divide/>
<apply>
<times/>
<ci> species_10 </ci>
<ci> species_13 </ci>
</apply>
<apply>
<times/>
<ci> mw39e8b6c4_2ae7_4846_9ac8_e77ee9c7191f </ci>
<ci> mw54c31bc3_16fc_462a_a55e_a766a97769ed </ci>
</apply>
</apply>
</apply>
</apply>
</apply>
</math>
</kineticLaw>
</reaction>
<reaction id="reaction_6" name="CC_6 alpha (fructose-1,6-bisphosphatase)" reversible="false" fast="false">
<annotation>

```
          <COPASI xmlns="http://www.copasi.org/static/sbml">
            <rdf:RDF xmlns:dcterms="http://purl.org/dc/terms/" xmlns:rdf="http://www.w3.org/1999/02/22-rdf-syntax-ns#">
              <rdf:Description rdf:about="#COPASI54">
                <dcterms:created>
                  <rdf:Description>
                    <dcterms:W3CDTF>2010-04-14T16:52:19Z</dcterms:W3CDTF>
                  </rdf:Description>
                </dcterms:created>
              </rdf:Description>
            </rdf:RDF>
          </COPASI>
        </annotation>
        <listOfReactants>
          <speciesReference species="species_14"/>
        </listOfReactants>
        <listOfProducts>
          <speciesReference species="species_15"/>
          <speciesReference species="species_12"/>
        </listOfProducts>
        <kineticLaw>
          <math xmlns="http://www.w3.org/1998/Math/MathML">
            <apply>
              <divide/>
              <apply>
                <times/>
                <ci> mw9dda631f_8096_4e56_a670_fdb559f36d12 </ci>
                <cn> 1.19 </cn>
```

```
        <ci> species_14 </ci>
      </apply>
      <apply>
        <plus/>
        <ci> species_14 </ci>
        <apply>
          <times/>
          <ci> mwe6b619dd_229f_4afa_af14_be3a25b0e262 </ci>
          <apply>
            <plus/>
            <cn type="integer"> 1 </cn>
            <apply>
              <divide/>
              <ci> species_15 </ci>
              <ci> mwa90c1c5f_dcc6_48a5_a99f_ce08e7c2c91b </ci>
            </apply>
            <apply>
              <divide/>
              <ci> species_12 </ci>
              <ci> mw9f468e68_fa4c_4260_a5af_3b6182abcb92 </ci>
            </apply>
          </apply>
        </apply>
      </apply>
    </apply>
  </math>
</kineticLaw>
```

```
</reaction>
<reaction id="reaction_7" name="CC_7 (transketolase)" fast="false">
  <annotation>
    <COPASI xmlns="http://www.copasi.org/static/sbml">
      <rdf:RDF xmlns:dcterms="http://purl.org/dc/terms/"
xmlns:rdf="http://www.w3.org/1999/02/22-rdf-syntax-ns#">
        <rdf:Description rdf:about="#COPASI55">
          <dcterms:created>
            <rdf:Description>
              <dcterms:W3CDTF>2010-04-15T18:06:55Z</dcterms:W3CDTF>
            </rdf:Description>
          </dcterms:created>
        </rdf:Description>
      </rdf:RDF>
    </COPASI>
  </annotation>
  <listOfReactants>
    <speciesReference species="species_15"/>
    <speciesReference species="species_10"/>
    <speciesReference species="species_19"/>
    <speciesReference species="species_18"/>
  </listOfReactants>
  <listOfProducts>
    <speciesReference species="species_16"/>
    <speciesReference species="species_17"/>
    <speciesReference species="species_19"/>
    <speciesReference species="species_18"/>
  </listOfProducts>
```

```
<kineticLaw>
  <math xmlns="http://www.w3.org/1998/Math/MathML">
    <apply>
      <divide/>
      <apply>
        <times/>
        <ci> mwea0f726e_b6ff_4cf0_8dba_9db4cc0827be </ci>
        <cn> 0.93 </cn>
        <apply>
          <minus/>
          <apply>
            <times/>
            <ci> species_15 </ci>
            <ci> species_10 </ci>
          </apply>
          <apply>
            <divide/>
            <apply>
              <times/>
              <ci> species_17 </ci>
              <ci> species_16 </ci>
            </apply>
            <ci> mw4ec82ea3_67a6_47ad_971a_52a33f180466 </ci>
          </apply>
        </apply>
      </apply>
      <apply>
```

<times/>
<apply>
<plus/>
<ci> species_15 </ci>
<apply>
<times/>
<ci> mwd037be1a_7ea7_4d03_b00e_8f358afc43f0 </ci>
<apply>
<plus/>
<cn type="integer"> 1 </cn>
<apply>
<divide/>
<ci> species_17 </ci>
<ci> mw6990314a_e646_4a51_b298_7b53a5a3ca3b </ci>
</apply>
<apply>
<divide/>
<ci> species_16 </ci>
<ci> mwd59eae5a_ff1f_4351_922c_d25f34ca05ac </ci>
</apply>
</apply>
</apply>
</apply>
<apply>
<plus/>
<ci> species_10 </ci>
<ci> mwd32ff45c_71b0_4916_8ffc_7683019b9311 </ci>

</apply>
</apply>
</apply>
</math>
</kineticLaw>
</reaction>
<reaction id="reaction_8" name="CC_8 (aldolase)" fast="false">
<annotation>
<COPASI xmlns="http://www.copasi.org/static/sbml">
<rdf:RDF xmlns:dcterms="http://purl.org/dc/terms/"
xmlns:rdf="http://www.w3.org/1999/02/22-rdf-syntax-ns#">
<rdf:Description rdf:about="#COPASI56">
<dcterms:created>
<rdf:Description>
<dcterms:W3CDTF>2010-04-20T11:20:56Z</dcterms:W3CDTF>
</rdf:Description>
</dcterms:created>
</rdf:Description>
</rdf:RDF>
</COPASI>
</annotation>
<listOfReactants>
<speciesReference species="species_13"/>
<speciesReference species="species_16"/>
</listOfReactants>
<listOfProducts>
<speciesReference species="species_8"/>
</listOfProducts>

```
<kineticLaw>
  <math xmlns="http://www.w3.org/1998/Math/MathML">
    <apply>
      <divide/>
      <apply>
        <times/>
        <ci> mw33ab4422_ba35_4d2b_9620_d274168c7e1d </ci>
        <cn> 1.36 </cn>
        <apply>
          <minus/>
          <apply>
            <times/>
            <ci> species_13 </ci>
            <ci> species_16 </ci>
          </apply>
          <apply>
            <divide/>
            <ci> species_8 </ci>
            <ci> mwf44df5f6_9e91_4081_a77d_911fe8867849 </ci>
          </apply>
        </apply>
      </apply>
      <apply>
        <times/>
        <apply>
          <plus/>
          <ci> species_16 </ci>
```

```xml
              <ci> mwa6f625ba_c9e6_40e4_bf00_d59146ed8f80 </ci>
            </apply>
            <apply>
              <plus/>
              <ci> species_13 </ci>
              <ci> mw28855de3_91da_4e09_b2c9_c0eb4b719af3 </ci>
            </apply>
          </apply>
        </apply>
      </math>
    </kineticLaw>
  </reaction>
  <reaction id="reaction_9" name="CC_9 (sedoheptulose-1,7 bisphosphatase)" reversible="false"
fast="false">
    <annotation>
      <COPASI xmlns="http://www.copasi.org/static/sbml">
        <rdf:RDF xmlns:dcterms="http://purl.org/dc/terms/"
xmlns:rdf="http://www.w3.org/1999/02/22-rdf-syntax-ns#">
          <rdf:Description rdf:about="#COPASI57">
            <dcterms:created>
              <rdf:Description>
                <dcterms:W3CDTF>2010-04-21T02:31:25Z</dcterms:W3CDTF>
              </rdf:Description>
            </dcterms:created>
          </rdf:Description>
        </rdf:RDF>
      </COPASI>
    </annotation>
```

```
&lt;listOfReactants&gt;
  &lt;speciesReference species="species_8"/&gt;
  &lt;speciesReference species="species_12"/&gt;
&lt;/listOfReactants&gt;
&lt;listOfProducts&gt;
  &lt;speciesReference species="species_19"/&gt;
  &lt;speciesReference species="species_12"/&gt;
  &lt;speciesReference species="species_12"/&gt;
&lt;/listOfProducts&gt;
&lt;kineticLaw&gt;
  &lt;math xmlns="http://www.w3.org/1998/Math/MathML"&gt;
    &lt;apply&gt;
      &lt;divide/&gt;
      &lt;apply&gt;
        &lt;times/&gt;
        &lt;ci&gt; mw5c6f658c_7714_4fbb_be83_5ef825b3e08b &lt;/ci&gt;
        &lt;cn&gt; 0.875 &lt;/cn&gt;
        &lt;ci&gt; species_8 &lt;/ci&gt;
      &lt;/apply&gt;
      &lt;apply&gt;
        &lt;plus/&gt;
        &lt;ci&gt; species_8 &lt;/ci&gt;
        &lt;apply&gt;
          &lt;times/&gt;
          &lt;ci&gt; mw2527d5af_e782_46fb_9b2c_875866d6ecd5 &lt;/ci&gt;
          &lt;apply&gt;
            &lt;plus/&gt;
```

```
&lt;cn type="integer"&gt; 1 &lt;/cn&gt;
&lt;apply&gt;
  &lt;divide/&gt;
  &lt;ci&gt; species_12 &lt;/ci&gt;
  &lt;ci&gt; mw118d7663_7b18_410d_8ca7_fc168407ba10 &lt;/ci&gt;
&lt;/apply&gt;
&lt;/apply&gt;
&lt;/apply&gt;
&lt;/apply&gt;
&lt;/apply&gt;
&lt;/math&gt;
&lt;/kineticLaw&gt;
&lt;/reaction&gt;
&lt;reaction id="reaction_10" name="CC_10 (transketolase)" fast="false"&gt;
&lt;annotation&gt;
&lt;COPASI xmlns="http://www.copasi.org/static/sbml"&gt;
<rdf:RDF xmlns:dcterms="http://purl.org/dc/terms/"
xmlns:rdf="http://www.w3.org/1999/02/22-rdf-syntax-ns#">
&lt;rdf:Description rdf:about="#COPASI58"&gt;
&lt;dcterms:created&gt;
&lt;rdf:Description&gt;
&lt;dcterms:W3CDTF&gt;2010-04-21T03:46:16Z&lt;/dcterms:W3CDTF&gt;
&lt;/rdf:Description&gt;
&lt;/dcterms:created&gt;
&lt;/rdf:Description&gt;
&lt;/rdf:RDF&gt;
&lt;/COPASI&gt;
&lt;/annotation&gt;
```

```
<listOfReactants>
  <speciesReference species="species_19"/>
  <speciesReference species="species_10"/>
  <speciesReference species="species_15"/>
  <speciesReference species="species_16"/>
</listOfReactants>
<listOfProducts>
  <speciesReference species="species_18"/>
  <speciesReference species="species_17"/>
  <speciesReference species="species_15"/>
  <speciesReference species="species_16"/>
</listOfProducts>
<kineticLaw>
  <math xmlns="http://www.w3.org/1998/Math/MathML">
    <apply>
      <divide/>
      <apply>
        <times/>
        <ci> mw6e8005dd_31e8_483e_902a_8870307bf558 </ci>
        <cn> 1.19 </cn>
        <apply>
          <minus/>
          <apply>
            <times/>
            <ci> species_10 </ci>
            <ci> species_19 </ci>
          </apply>
```

```
    <apply>
      <divide/>
      <apply>
        <times/>
        <ci> species_18 </ci>
        <ci> species_17 </ci>
      </apply>
      <ci> mw075f4d45_ceca_4aad_bc29_89b6915e46f7 </ci>
    </apply>
  </apply>
</apply>
<apply>
  <times/>
  <ci> mwf8558c32_d374_4352_b837_54ce36f88a5e </ci>
  <ci> mwf187cf00_32ca_422a_9134_ffd278f81118 </ci>
  <apply>
    <plus/>
    <cn type="integer"> 1 </cn>
    <apply>
      <times/>
      <apply>
        <plus/>
        <cn type="integer"> 1 </cn>
        <apply>
          <divide/>
          <ci> species_10 </ci>
          <ci> mw7d11f964_6e54_4584_8c91_1808e4e850fd </ci>
```

```
    </apply>
  </apply>
  <apply>
    <plus/>
    <apply>
      <divide/>
      <ci> species_15 </ci>
      <ci> mwe195dc18_fc3c_4329_905f_5bb2fc3efe19 </ci>
    </apply>
    <apply>
      <divide/>
      <ci> species_19 </ci>
      <ci> mwb61a7f15_d0aa_4449_85bb_2cdf4a397051 </ci>
    </apply>
  </apply>
</apply>
<apply>
  <divide/>
  <ci> species_10 </ci>
  <ci> mwe1915e65_ff53_471a_9d2c_b92b8d75f287 </ci>
</apply>
<apply>
  <divide/>
  <apply>
    <plus/>
    <apply>
      <times/>
```

```
              <ci> species_17 </ci>
              <apply>
                <plus/>
                <cn type="integer"> 1 </cn>
                <apply>
                  <divide/>
                  <apply>
                    <plus/>
                    <ci> species_16 </ci>
                    <ci> species_18 </ci>
                  </apply>
                  <ci> mwf8558c32_d374_4352_b837_54ce36f88a5e </ci>
                </apply>
              </apply>
            </apply>
            <ci> species_16 </ci>
            <ci> species_18 </ci>
          </apply>
          <ci> mwf187cf00_32ca_422a_9134_ffd278f81118 </ci>
        </apply>
      </apply>
    </apply>
  </apply>
 </math>
</kineticLaw>
</reaction>
<reaction id="reaction_11" name="C_11 (phosphopentose isomerase)" fast="false">
```

```
<annotation>
  <COPASI xmlns="http://www.copasi.org/static/sbml">
    <rdf:RDF xmlns:dcterms="http://purl.org/dc/terms/"
xmlns:rdf="http://www.w3.org/1999/02/22-rdf-syntax-ns#">
      <rdf:Description rdf:about="#COPASI59">
        <dcterms:created>
          <rdf:Description>
            <dcterms:W3CDTF>2010-04-23T03:43:39Z</dcterms:W3CDTF>
          </rdf:Description>
        </dcterms:created>
      </rdf:Description>
    </rdf:RDF>
  </COPASI>
</annotation>
<listOfReactants>
  <speciesReference species="species_18"/>
</listOfReactants>
<listOfProducts>
  <speciesReference species="species_20"/>
</listOfProducts>
<kineticLaw>
  <math xmlns="http://www.w3.org/1998/Math/MathML">
    <apply>
      <divide/>
      <apply>
        <times/>
        <ci> mw4fbb318e_c400_4af5_8e3f_e80aec639ee3 </ci>
        <cn> 0.96 </cn>
```

```
  <apply>
    <minus/>
    <ci> species_18 </ci>
    <apply>
      <divide/>
      <ci> species_20 </ci>
      <ci> mw94fab6bb_d1d8_4344_ac40_d9ea47173826 </ci>
    </apply>
  </apply>
</apply>
<apply>
  <times/>
  <ci> mwf2185be5_b4b0_4bf4_a6d3_0a758e4fbcca </ci>
  <apply>
    <plus/>
    <cn type="integer"> 1 </cn>
    <apply>
      <divide/>
      <ci> species_18 </ci>
      <ci> mwf2185be5_b4b0_4bf4_a6d3_0a758e4fbcca </ci>
    </apply>
    <apply>
      <divide/>
      <ci> species_20 </ci>
      <ci> mw9168b04c_6d3a_4f60_8906_5b5293d6a01e </ci>
    </apply>
  </apply>
```

```
          </apply>
        </apply>
      </math>
    </kineticLaw>
  </reaction>
  <reaction id="reaction_12" name="C_12 (phosphopentose epimerase)" fast="false">
    <annotation>
      <COPASI xmlns="http://www.copasi.org/static/sbml">
        <rdf:RDF xmlns:dcterms="http://purl.org/dc/terms/"
xmlns:rdf="http://www.w3.org/1999/02/22-rdf-syntax-ns#">
          <rdf:Description rdf:about="#COPASI60">
            <dcterms:created>
              <rdf:Description>
                <dcterms:W3CDTF>2010-04-23T03:43:48Z</dcterms:W3CDTF>
              </rdf:Description>
            </dcterms:created>
          </rdf:Description>
        </rdf:RDF>
      </COPASI>
    </annotation>
    <listOfReactants>
      <speciesReference species="species_17"/>
    </listOfReactants>
    <listOfProducts>
      <speciesReference species="species_20"/>
    </listOfProducts>
    <kineticLaw>
      <math xmlns="http://www.w3.org/1998/Math/MathML">
```

```
      <apply>
        <divide/>
        <apply>
          <times/>
          <ci> mw702a7f39_5675_48ad_953a_4bcbbdc8e1a2 </ci>
          <cn> 0.88 </cn>
          <apply>
            <minus/>
            <ci> species_17 </ci>
            <apply>
              <divide/>
              <ci> species_20 </ci>
              <ci> mw46a0d4e0_a24e_4d84_ac9d_1d098ed93c0b </ci>
            </apply>
          </apply>
        </apply>
        <apply>
          <plus/>
          <ci> species_17 </ci>
          <ci> species_20 </ci>
        </apply>
      </apply>
    </math>
  </kineticLaw>
</reaction>
<reaction id="reaction_13" name="C_13 (phosphoribulokinase)" reversible="false" fast="false">
  <annotation>
```

```
          <COPASI xmlns="http://www.copasi.org/static/sbml">
            <rdf:RDF xmlns:dcterms="http://purl.org/dc/terms/"
xmlns:rdf="http://www.w3.org/1999/02/22-rdf-syntax-ns#">
              <rdf:Description rdf:about="#COPASI61">
                <dcterms:created>
                  <rdf:Description>
                    <dcterms:W3CDTF>2010-04-23T03:48:04Z</dcterms:W3CDTF>
                  </rdf:Description>
                </dcterms:created>
              </rdf:Description>
            </rdf:RDF>
          </COPASI>
        </annotation>
        <listOfReactants>
          <speciesReference species="species_20"/>
          <speciesReference species="species_7"/>
          <speciesReference species="species_12"/>
          <speciesReference species="species_2"/>
        </listOfReactants>
        <listOfProducts>
          <speciesReference species="species_3"/>
          <speciesReference species="species_4"/>
          <speciesReference species="species_12"/>
          <speciesReference species="species_2"/>
        </listOfProducts>
        <kineticLaw>
          <math xmlns="http://www.w3.org/1998/Math/MathML">
            <apply>
```

```
<divide/>
<apply>
 <times/>
 <ci> mwd486ae05_3de1_42aa_9584_2edc458d0c59 </ci>
 <cn> 0.99 </cn>
 <ci> species_7 </ci>
 <ci> species_20 </ci>
</apply>
<apply>
 <times/>
 <apply>
  <plus/>
  <apply>
   <times/>
   <ci> species_7 </ci>
   <apply>
    <plus/>
    <cn type="integer"> 1 </cn>
    <apply>
     <divide/>
     <ci> species_4 </ci>
     <ci> mw16745cb2_2a9e_48f6_8cef_80e44ec67119 </ci>
    </apply>
   </apply>
  </apply>
  <apply>
   <times/>
```

<ci> mwc1d6540f_78d6_40ae_8e94_d6db3d674a6e </ci>
<apply>
<plus/>
<cn type="integer"> 1 </cn>
<apply>
<divide/>
<ci> species_4 </ci>
<ci> mw814665c4_d157_4663_a75e_af6f8f891fb0 </ci>
</apply>
</apply>
</apply>
</apply>
<apply>
<plus/>
<ci> species_20 </ci>
<apply>
<times/>
<ci> mw9c902b95_94d4_4dff_912f_eb93bd74d440 </ci>
<apply>
<plus/>
<cn type="integer"> 1 </cn>
<apply>
<divide/>
<ci> species_2 </ci>
<ci> mwd947eee9_9a97_4305_a38f_5d0342490834 </ci>
</apply>
<apply>

```
                <divide/>
                <ci> species_3 </ci>
                <ci> mw0b12ed4b_ffff_47bd_a925_140813e42732 </ci>
              </apply>
              <apply>
                <divide/>
                <ci> species_12 </ci>
                <ci> mw6bb6ce48_f2df_4db7_abe8_95a611a6672b </ci>
              </apply>
            </apply>
          </apply>
        </apply>
      </apply>
    </apply>
  </math>
</kineticLaw>
</reaction>
<reaction id="reaction_14" name="SS_1 (glycogen and sucrose synthesis)" fast="false">
  <annotation>
    <COPASI xmlns="http://www.copasi.org/static/sbml">
      <rdf:RDF xmlns:dcterms="http://purl.org/dc/terms/"
xmlns:rdf="http://www.w3.org/1999/02/22-rdf-syntax-ns#">
        <rdf:Description rdf:about="#COPASI62">
          <dcterms:created>
            <rdf:Description>
              <dcterms:W3CDTF>2010-04-29T13:08:18Z</dcterms:W3CDTF>
            </rdf:Description>
          </dcterms:created>
```

```
      </rdf:Description>
    </rdf:RDF>
  </COPASI>
</annotation>
<listOfReactants>
  <speciesReference species="species_15"/>
</listOfReactants>
<listOfProducts>
  <speciesReference species="species_21"/>
</listOfProducts>
<kineticLaw>
  <math xmlns="http://www.w3.org/1998/Math/MathML">
    <apply>
      <divide/>
      <apply>
        <times/>
        <ci> mw243dc3c0_44b5_4ef5_b8ba_92846b4fee4c </ci>
        <cn> 0.63 </cn>
        <apply>
          <minus/>
          <apply>
            <times/>
            <ci> mwaeeded85_9203_48b6_89a9_9dbf6c0d38da </ci>
            <ci> species_15 </ci>
          </apply>
          <ci> species_21 </ci>
        </apply>
```

```
          &lt;/apply&gt;
          &lt;apply&gt;
            &lt;plus/&gt;
            &lt;ci&gt; species_15 &lt;/ci&gt;
            &lt;ci&gt; species_21 &lt;/ci&gt;
          &lt;/apply&gt;
        &lt;/apply&gt;
      &lt;/math&gt;
    &lt;/kineticLaw&gt;
  &lt;/reaction&gt;
  &lt;reaction id="reaction_17" name="LIGHT_1 (ATP synthase)" reversible="false" fast="false"&gt;
    &lt;annotation&gt;
      &lt;COPASI xmlns="http://www.copasi.org/static/sbml"&gt;
        <rdf:RDF xmlns:dcterms="http://purl.org/dc/terms/"
xmlns:rdf="http://www.w3.org/1999/02/22-rdf-syntax-ns#">
          &lt;rdf:Description rdf:about="#COPASI65"&gt;
            &lt;dcterms:created&gt;
              &lt;rdf:Description&gt;
                &lt;dcterms:W3CDTF&gt;2010-05-24T02:11:27Z&lt;/dcterms:W3CDTF&gt;
              &lt;/rdf:Description&gt;
            &lt;/dcterms:created&gt;
          &lt;/rdf:Description&gt;
        &lt;/rdf:RDF&gt;
      &lt;/COPASI&gt;
    &lt;/annotation&gt;
    &lt;listOfReactants&gt;
      &lt;speciesReference species="species_12"/&gt;
      &lt;speciesReference species="species_4"/&gt;
```

```
      </listOfReactants>
      <listOfProducts>
        <speciesReference species="species_7"/>
      </listOfProducts>
      <kineticLaw>
        <math xmlns="http://www.w3.org/1998/Math/MathML">
          <apply>
            <times/>
            <ci> parameter_1 </ci>
            <cn> 0.25 </cn>
            <ci> species_12 </ci>
            <ci> species_4 </ci>
          </apply>
        </math>
      </kineticLaw>
    </reaction>
    <reaction id="reaction_18" name="LIGHT_2" reversible="false" fast="false">
      <annotation>
        <COPASI xmlns="http://www.copasi.org/static/sbml">
          <rdf:RDF xmlns:dcterms="http://purl.org/dc/terms/"
xmlns:rdf="http://www.w3.org/1999/02/22-rdf-syntax-ns#">
            <rdf:Description rdf:about="#COPASI66">
              <dcterms:created>
                <rdf:Description>
                  <dcterms:W3CDTF>2010-06-09T00:18:40Z</dcterms:W3CDTF>
                </rdf:Description>
              </dcterms:created>
            </rdf:Description>
```

```
          &lt;/rdf:RDF&gt;
        &lt;/COPASI&gt;
      &lt;/annotation&gt;
      &lt;listOfReactants&gt;
        &lt;speciesReference species="species_11"/&gt;
      &lt;/listOfReactants&gt;
      &lt;listOfProducts&gt;
        &lt;speciesReference species="species_5"/&gt;
      &lt;/listOfProducts&gt;
      &lt;kineticLaw&gt;
        &lt;math xmlns="http://www.w3.org/1998/Math/MathML"&gt;
          &lt;apply&gt;
            &lt;times/&gt;
            &lt;ci&gt; parameter_2 &lt;/ci&gt;
            &lt;cn&gt; 0.21 &lt;/cn&gt;
            &lt;ci&gt; species_11 &lt;/ci&gt;
          &lt;/apply&gt;
        &lt;/math&gt;
      &lt;/kineticLaw&gt;
    &lt;/reaction&gt;
    &lt;reaction id="reaction_19" name="Sink GAP" reversible="false" fast="false"&gt;
      &lt;annotation&gt;
        &lt;COPASI xmlns="http://www.copasi.org/static/sbml"&gt;
          <rdf:RDF xmlns:dcterms="http://purl.org/dc/terms/"
xmlns:rdf="http://www.w3.org/1999/02/22-rdf-syntax-ns#">
            &lt;rdf:Description rdf:about="#COPASI67"&gt;
              &lt;dcterms:created&gt;
                &lt;rdf:Description&gt;
```

```
          <dcterms:W3CDTF>2011-04-26T00:16:45Z</dcterms:W3CDTF>
        </rdf:Description>
      </dcterms:created>
    </rdf:Description>
  </rdf:RDF>
</COPASI>
</annotation>
<listOfReactants>
  <speciesReference species="species_10"/>
</listOfReactants>
<listOfProducts>
  <speciesReference species="mwaf22c073_9b9c_42b1_bd8e_6e65777ddddc"
stoichiometry="3"/>
</listOfProducts>
<kineticLaw>
  <math xmlns="http://www.w3.org/1998/Math/MathML">
    <apply>
      <divide/>
      <apply>
        <times/>
        <ci> mwe110663f_33e9_415c_96f8_04e92e007bb4 </ci>
        <ci> species_10 </ci>
      </apply>
      <apply>
        <plus/>
        <ci> mw395deb93_b80e_4154_b71a_add84953daaf </ci>
        <ci> species_10 </ci>
      </apply>
```

```
        </apply>
      </math>
    </kineticLaw>
  </reaction>
  <reaction id="reaction_20" name="Sink E4P" reversible="false" fast="false">
    <annotation>
      <COPASI xmlns="http://www.copasi.org/static/sbml">
        <rdf:RDF xmlns:dcterms="http://purl.org/dc/terms/"
xmlns:rdf="http://www.w3.org/1999/02/22-rdf-syntax-ns#">
          <rdf:Description rdf:about="#COPASI68">
            <dcterms:created>
              <rdf:Description>
                <dcterms:W3CDTF>2011-04-26T00:17:24Z</dcterms:W3CDTF>
              </rdf:Description>
            </dcterms:created>
          </rdf:Description>
        </rdf:RDF>
      </COPASI>
    </annotation>
    <listOfReactants>
      <speciesReference species="species_16"/>
    </listOfReactants>
    <listOfProducts>
      <speciesReference species="mw09d7964e_16d4_4c3d_a1f5_d98a7ab66891"
stoichiometry="4"/>
    </listOfProducts>
    <kineticLaw>
      <math xmlns="http://www.w3.org/1998/Math/MathML">
```

```
            <apply>
              <divide/>
              <apply>
                <times/>
                <ci> mw715a0d4a_6423_4755_a1b5_7d9e5b1852a2 </ci>
                <ci> species_16 </ci>
              </apply>
              <apply>
                <plus/>
                <ci> mw4d3fe418_9692_4308_a1c1_64b6e3b6ad82 </ci>
                <ci> species_16 </ci>
              </apply>
            </apply>
          </math>
        </kineticLaw>
      </reaction>
      <reaction id="reaction_21" name="Sink Ri5P" reversible="false" fast="false">
        <annotation>
          <COPASI xmlns="http://www.copasi.org/static/sbml">
            <rdf:RDF xmlns:dcterms="http://purl.org/dc/terms/"
xmlns:rdf="http://www.w3.org/1999/02/22-rdf-syntax-ns#">
              <rdf:Description rdf:about="#COPASI69">
                <dcterms:created>
                  <rdf:Description>
                    <dcterms:W3CDTF>2011-04-26T00:17:46Z</dcterms:W3CDTF>
                  </rdf:Description>
                </dcterms:created>
              </rdf:Description>
```

```
          </rdf:RDF>
        </COPASI>
      </annotation>
      <listOfReactants>
        <speciesReference species="species_18"/>
      </listOfReactants>
      <listOfProducts>
        <speciesReference species="mw70a9d4b8_2ca3_4692_a159_702d8464b7e7"
stoichiometry="5"/>
      </listOfProducts>
      <kineticLaw>
        <math xmlns="http://www.w3.org/1998/Math/MathML">
          <apply>
            <divide/>
            <apply>
              <times/>
              <ci> mw6caa1525_e69b_4ab2_b8c7_2b7deeb5ee58 </ci>
              <ci> species_18 </ci>
            </apply>
            <apply>
              <plus/>
              <ci> mwe871df2e_a7b7_49c5_8a95_809524380cbd </ci>
              <ci> species_18 </ci>
            </apply>
          </apply>
        </math>
      </kineticLaw>
    </reaction>
```

```
      <reaction id="reaction_22" name="PP_2a (phosphoglycolate phosphatase)" reversible="false"
fast="false">
        &lt;annotation&gt;
          &lt;COPASI xmlns="http://www.copasi.org/static/sbml"&gt;
            <rdf:RDF xmlns:dcterms="http://purl.org/dc/terms/"
xmlns:rdf="http://www.w3.org/1999/02/22-rdf-syntax-ns#">
              &lt;rdf:Description rdf:about="#COPASI70"&gt;
                &lt;dcterms:created&gt;
                  &lt;rdf:Description&gt;
                    &lt;dcterms:W3CDTF&gt;2011-05-09T23:56:05Z&lt;/dcterms:W3CDTF&gt;
                  &lt;/rdf:Description&gt;
                &lt;/dcterms:created&gt;
              &lt;/rdf:Description&gt;
            &lt;/rdf:RDF&gt;
          &lt;/COPASI&gt;
        &lt;/annotation&gt;
        &lt;listOfReactants&gt;
          &lt;speciesReference species="species_25"/&gt;
        &lt;/listOfReactants&gt;
        &lt;listOfProducts&gt;
          &lt;speciesReference species="species_26"/&gt;
          &lt;speciesReference species="species_12"/&gt;
        &lt;/listOfProducts&gt;
        &lt;kineticLaw&gt;
          &lt;math xmlns="http://www.w3.org/1998/Math/MathML"&gt;
            &lt;apply&gt;
              &lt;divide/&gt;
              &lt;apply&gt;
```

```
  <times/>
  <ci> mw3c5b8b60_a85d_4b23_8f30_04a8715af44c </ci>
  <cn> 0.84 </cn>
  <ci> species_25 </ci>
</apply>
<apply>
  <plus/>
  <ci> species_25 </ci>
  <apply>
    <times/>
    <ci> mw04118d88_412e_49b2_8ed5_aba0f23d1258 </ci>
    <apply>
      <plus/>
      <cn type="integer"> 1 </cn>
      <apply>
        <divide/>
        <ci> species_26 </ci>
        <ci> KI1121 </ci>
      </apply>
    </apply>
    <apply>
      <plus/>
      <cn type="integer"> 1 </cn>
      <apply>
        <divide/>
        <ci> species_12 </ci>
        <ci> KI1122 </ci>
```

```
              </apply>
            </apply>
          </apply>
        </apply>
      </apply>
    </math>
    <listOfParameters>
      <parameter id="KI1121" name="KI1121" value="94"/>
      <parameter id="KI1122" name="KI1122" value="2.55"/>
    </listOfParameters>
  </kineticLaw>
</reaction>
<reaction id="reaction_23" name="PP_3 (glycolate oxidase)" reversible="false" fast="false">
  <annotation>
    <COPASI xmlns="http://www.copasi.org/static/sbml">
      <rdf:RDF xmlns:dcterms="http://purl.org/dc/terms/"
xmlns:rdf="http://www.w3.org/1999/02/22-rdf-syntax-ns#">
        <rdf:Description rdf:about="#COPASI71">
          <dcterms:created>
            <rdf:Description>
              <dcterms:W3CDTF>2011-05-10T00:12:50Z</dcterms:W3CDTF>
            </rdf:Description>
          </dcterms:created>
        </rdf:Description>
      </rdf:RDF>
    </COPASI>
  </annotation>
  <listOfReactants>
```

```
    <speciesReference species="species_26"/>
  </listOfReactants>
  <listOfProducts>
    <speciesReference species="species_27"/>
  </listOfProducts>
  <kineticLaw>
    <math xmlns="http://www.w3.org/1998/Math/MathML">
      <apply>
        <divide/>
        <apply>
          <times/>
          <ci> mw5f743344_e84b_4721_8bd4_3643964b38ea </ci>
          <cn type="integer"> 2 </cn>
          <ci> species_26 </ci>
        </apply>
        <apply>
          <plus/>
          <ci> species_26 </ci>
          <ci> Km121 </ci>
        </apply>
      </apply>
    </math>
    <listOfParameters>
      <parameter id="Km121" name="Km121" value="0.1"/>
    </listOfParameters>
  </kineticLaw>
</reaction>
```

```
<reaction id="reaction_24" name="PP_4 (serineglyoxylate transaminase)" fast="false">
 <annotation>
  <COPASI xmlns="http://www.copasi.org/static/sbml">
   <rdf:RDF xmlns:dcterms="http://purl.org/dc/terms/"
xmlns:rdf="http://www.w3.org/1999/02/22-rdf-syntax-ns#">
    <rdf:Description rdf:about="#COPASI72">
     <dcterms:created>
      <rdf:Description>
       <dcterms:W3CDTF>2011-05-10T00:16:51Z</dcterms:W3CDTF>
      </rdf:Description>
     </dcterms:created>
    </rdf:Description>
   </rdf:RDF>
  </COPASI>
 </annotation>
 <listOfReactants>
  <speciesReference species="species_27"/>
  <speciesReference species="species_29"/>
 </listOfReactants>
 <listOfProducts>
  <speciesReference species="species_30"/>
  <speciesReference species="species_28"/>
 </listOfProducts>
 <kineticLaw>
  <math xmlns="http://www.w3.org/1998/Math/MathML">
   <apply>
    <divide/>
    <apply>
```

```
  <times/>
  <ci> mw43b7b905_45eb_4193_9d33_c0623544faba </ci>
  <cn> 0.42 </cn>
  <apply>
   <minus/>
   <apply>
    <times/>
    <ci> species_27 </ci>
    <ci> species_29 </ci>
   </apply>
   <apply>
    <divide/>
    <apply>
     <times/>
     <ci> species_30 </ci>
     <ci> species_28 </ci>
    </apply>
    <ci> KE124 </ci>
   </apply>
  </apply>
 </apply>
 <apply>
  <times/>
  <apply>
   <plus/>
   <ci> species_27 </ci>
   <ci> Km1241 </ci>
```

```
      </apply>
      <apply>
        <plus/>
        <ci> species_29 </ci>
        <apply>
          <times/>
          <ci> Km1242 </ci>
          <apply>
            <plus/>
            <cn type="integer"> 1 </cn>
            <apply>
              <divide/>
              <ci> species_28 </ci>
              <ci> KI124 </ci>
            </apply>
          </apply>
        </apply>
      </apply>
    </apply>
  </apply>
</math>
<listOfParameters>
  <parameter id="KE124" name="KE124" value="607"/>
  <parameter id="Km1241" name="Km1241" value="0.15"/>
  <parameter id="Km1242" name="Km1242" value="1.7"/>
  <parameter id="KI124" name="KI124" value="2"/>
</listOfParameters>
```

</kineticLaw>

</reaction>

<reaction id="reaction_25" name="PP_5 (serine hydroxymethyltransferase)" reversible="false" fast="false">

<annotation>

<COPASI xmlns="http://www.copasi.org/static/sbml">

<rdf:RDF xmlns:dcterms="http://purl.org/dc/terms/" xmlns:rdf="http://www.w3.org/1999/02/22-rdf-syntax-ns#">

<rdf:Description rdf:about="#COPASI73">

<dcterms:created>

<rdf:Description>

<dcterms:W3CDTF>2011-05-10T00:22:17Z</dcterms:W3CDTF>

</rdf:Description>

</dcterms:created>

</rdf:Description>

</rdf:RDF>

</COPASI>

</annotation>

<listOfReactants>

<speciesReference species="species_28" stoichiometry="2"/>

</listOfReactants>

<listOfProducts>

<speciesReference species="species_29"/>

</listOfProducts>

<kineticLaw>

<math xmlns="http://www.w3.org/1998/Math/MathML">

<apply>

<divide/>

```
            <apply>
              <times/>
              <ci> mw21878232_3329_413d_9ad8_7dd1cfc2be9c </ci>
              <ci> species_28 </ci>
            </apply>
            <apply>
              <plus/>
              <ci> mwK13dcce1c1_26b6_4b0e_8782_547ab513fb70 </ci>
              <ci> species_28 </ci>
            </apply>
          </apply>
        </math>
        <listOfParameters>
          <parameter id="mwK13dcce1c1_26b6_4b0e_8782_547ab513fb70" name="K1" value="6"/>
        </listOfParameters>
      </kineticLaw>
    </reaction>
    <reaction id="reaction_26" name="PP_6 (hydroxypyruvate reductase)" reversible="false"
fast="false">
      <annotation>
        <COPASI xmlns="http://www.copasi.org/static/sbml">
          <rdf:RDF xmlns:dcterms="http://purl.org/dc/terms/"
xmlns:rdf="http://www.w3.org/1999/02/22-rdf-syntax-ns#">
            <rdf:Description rdf:about="#COPASI74">
              <dcterms:created>
                <rdf:Description>
                  <dcterms:W3CDTF>2011-05-10T01:04:31Z</dcterms:W3CDTF>
                </rdf:Description>
```

```
        &lt;/dcterms:created&gt;
      &lt;/rdf:Description&gt;
    &lt;/rdf:RDF&gt;
  &lt;/COPASI&gt;
&lt;/annotation&gt;
&lt;listOfReactants&gt;
  &lt;speciesReference species="species_30"/&gt;
&lt;/listOfReactants&gt;
&lt;listOfProducts&gt;
  &lt;speciesReference species="species_31"/&gt;
&lt;/listOfProducts&gt;
&lt;kineticLaw&gt;
  &lt;math xmlns="http://www.w3.org/1998/Math/MathML"&gt;
    &lt;apply&gt;
      &lt;divide/&gt;
      &lt;apply&gt;
        &lt;times/&gt;
        &lt;ci&gt; mw9331ee05_e8ae_4da7_be3c_2ae749a0c97a &lt;/ci&gt;
        &lt;apply&gt;
          &lt;minus/&gt;
          &lt;ci&gt; species_30 &lt;/ci&gt;
          &lt;apply&gt;
            &lt;divide/&gt;
            &lt;ci&gt; species_31 &lt;/ci&gt;
            &lt;ci&gt; KE123 &lt;/ci&gt;
          &lt;/apply&gt;
        &lt;/apply&gt;
```

```
        </apply>
        <apply>
          <plus/>
          <ci> species_30 </ci>
          <apply>
            <times/>
            <ci> Km1231 </ci>
            <apply>
              <plus/>
              <cn type="integer"> 1 </cn>
              <apply>
                <divide/>
                <ci> species_30 </ci>
                <ci> KI123 </ci>
              </apply>
            </apply>
          </apply>
        </apply>
      </apply>
    </math>
    <listOfParameters>
      <parameter id="KE123" name="KE123" value="250000"/>
      <parameter id="Km1231" name="Km1231" value="0.09"/>
      <parameter id="KI123" name="KI123" value="12"/>
    </listOfParameters>
  </kineticLaw>
</reaction>
```

```
      <reaction id="reaction_27" name="PP_7 (glycerate kinase)" reversible="false" fast="false">
        <annotation>
          <COPASI xmlns="http://www.copasi.org/static/sbml">
            <rdf:RDF xmlns:dcterms="http://purl.org/dc/terms/"
xmlns:rdf="http://www.w3.org/1999/02/22-rdf-syntax-ns#">
              <rdf:Description rdf:about="#COPASI75">
                <dcterms:created>
                  <rdf:Description>
                    <dcterms:W3CDTF>2011-05-10T01:09:19Z</dcterms:W3CDTF>
                  </rdf:Description>
                </dcterms:created>
              </rdf:Description>
            </rdf:RDF>
          </COPASI>
        </annotation>
        <listOfReactants>
          <speciesReference species="species_31"/>
          <speciesReference species="species_7"/>
        </listOfReactants>
        <listOfProducts>
          <speciesReference species="species_2"/>
          <speciesReference species="species_4"/>
        </listOfProducts>
        <kineticLaw>
          <math xmlns="http://www.w3.org/1998/Math/MathML">
            <apply>
              <divide/>
              <apply>
```

<times/>
<ci> mw4a170042_d461_4f96_b16d_2969db08423c </ci>
<apply>
<minus/>
<apply>
<times/>
<ci> species_7 </ci>
<ci> species_31 </ci>
</apply>
<apply>
<divide/>
<apply>
<times/>
<ci> species_4 </ci>
<ci> species_2 </ci>
</apply>
<ci> KE113 </ci>
</apply>
</apply>
</apply>
<apply>
<times/>
<apply>
<plus/>
<ci> species_7 </ci>
<apply>
<times/>

```
        <ci> Km1131 </ci>
        <apply>
          <plus/>
          <cn type="integer"> 1 </cn>
          <apply>
            <divide/>
            <ci> species_2 </ci>
            <ci> KI113 </ci>
          </apply>
        </apply>
      </apply>
    </apply>
    <apply>
      <plus/>
      <ci> species_31 </ci>
      <ci> Km1132 </ci>
    </apply>
  </apply>
 </apply>
</math>
<listOfParameters>
  <parameter id="KE113" name="KE113" value="300"/>
  <parameter id="Km1131" name="Km1131" value="0.21"/>
  <parameter id="KI113" name="KI113" value="0.36"/>
  <parameter id="Km1132" name="Km1132" value="0.25"/>
</listOfParameters>
</kineticLaw>
```

</reaction>

<reaction id="reaction_30" name="GL_4 (enolase)" fast="false">

<annotation>

<COPASI xmlns="http://www.copasi.org/static/sbml">

<rdf:RDF xmlns:dcterms="http://purl.org/dc/terms/" xmlns:rdf="http://www.w3.org/1999/02/22-rdf-syntax-ns#">

<rdf:Description rdf:about="#COPASI78">

<dcterms:created>

<rdf:Description>

<dcterms:W3CDTF>2011-05-10T15:12:27Z</dcterms:W3CDTF>

</rdf:Description>

</dcterms:created>

</rdf:Description>

</rdf:RDF>

</COPASI>

</annotation>

<listOfReactants>

<speciesReference species="species_35"/>

</listOfReactants>

<listOfProducts>

<speciesReference species="species_32"/>

</listOfProducts>

<kineticLaw>

<math xmlns="http://www.w3.org/1998/Math/MathML">

<apply>

<divide/>

<apply>

<times/>

<ci> mw68a26bf2_520b_4b35_90e0_511ec4bbc859 </ci>

<cn> 1.004 </cn>

<apply>

<minus/>

<ci> species_35 </ci>

<apply>

<divide/>

<ci> species_32 </ci>

<ci> mwdacf4f8b_f1db_480a_aabf_ff89dc90e087 </ci>

</apply>

</apply>

</apply>

<apply>

<times/>

<ci> mwe806711a_3cbc_4d66_a530_d7ae4e673eea </ci>

<apply>

<plus/>

<cn type="integer"> 1 </cn>

<apply>

<divide/>

<ci> species_35 </ci>

<ci> mwe806711a_3cbc_4d66_a530_d7ae4e673eea </ci>

</apply>

<apply>

<divide/>

<ci> species_32 </ci>

<ci> mwdee0f9ac_28b4_444f_af80_30c299f78093 </ci>

```
              </apply>
            </apply>
          </apply>
        </apply>
      </math>
    </kineticLaw>
  </reaction>
  <reaction id="reaction_31" name="Sink PEP" reversible="false" fast="false">
    <annotation>
      <COPASI xmlns="http://www.copasi.org/static/sbml">
        <rdf:RDF xmlns:dcterms="http://purl.org/dc/terms/"
xmlns:rdf="http://www.w3.org/1999/02/22-rdf-syntax-ns#">
          <rdf:Description rdf:about="#COPASI79">
            <dcterms:created>
              <rdf:Description>
                <dcterms:W3CDTF>2011-05-10T16:28:58Z</dcterms:W3CDTF>
              </rdf:Description>
            </dcterms:created>
          </rdf:Description>
        </rdf:RDF>
      </COPASI>
    </annotation>
    <listOfReactants>
      <speciesReference species="species_32"/>
    </listOfReactants>
    <listOfProducts>
      <speciesReference species="mwa8568197_97f2_4c67_8727_f8d9ed6895e3"
stoichiometry="3"/>
```

```
        </listOfProducts>
        <kineticLaw>
          <math xmlns="http://www.w3.org/1998/Math/MathML">
            <apply>
              <divide/>
              <apply>
                <times/>
                <ci> mw5723da7e_7ee9_4b45_b397_c4fad0251fa2 </ci>
                <cn> 0.12 </cn>
                <ci> species_32 </ci>
              </apply>
              <apply>
                <plus/>
                <ci> mw70b2b9c1_0904_4851_aee4_8526d26a4c7f </ci>
                <ci> species_32 </ci>
              </apply>
            </apply>
          </math>
        </kineticLaw>
      </reaction>
      <reaction id="reaction_33" name="GSM_1 (simplified phosphoserine transaminase)"
reversible="false" fast="false">
        <annotation>
          <COPASI xmlns="http://www.copasi.org/static/sbml">
            <rdf:RDF xmlns:dcterms="http://purl.org/dc/terms/"
xmlns:rdf="http://www.w3.org/1999/02/22-rdf-syntax-ns#">
              <rdf:Description rdf:about="#COPASI81">
                <dcterms:created>
```

```
          <rdf:Description>
            <dcterms:W3CDTF>2011-06-15T16:11:22Z</dcterms:W3CDTF>
          </rdf:Description>
        </dcterms:created>
      </rdf:Description>
    </rdf:RDF>
  </COPASI>
</annotation>
<listOfReactants>
  <speciesReference species="species_2"/>
</listOfReactants>
<listOfProducts>
  <speciesReference species="species_29"/>
</listOfProducts>
<kineticLaw>
  <math xmlns="http://www.w3.org/1998/Math/MathML">
    <apply>
      <divide/>
      <apply>
        <times/>
        <ci> mwa5897908_6cba_4e9b_ae16_669f6cc2215d </ci>
        <cn> 0.3 </cn>
        <ci> species_2 </ci>
      </apply>
      <apply>
        <plus/>
        <ci> mwK17d0edf01_29a7_4ee8_8bbf_a6dd0a6e695e </ci>
```

```
            <ci> species_2 </ci>
          </apply>
        </apply>
      </math>
      <listOfParameters>
        <parameter id="mwK17d0edf01_29a7_4ee8_8bbf_a6dd0a6e695e" name="K_synth_SER" value="2"/>
      </listOfParameters>
    </kineticLaw>
  </reaction>
  <reaction id="reaction_34" name="GC_1 (tartronate semialdehyde synthase)" reversible="false" fast="false">
    <annotation>
      <COPASI xmlns="http://www.copasi.org/static/sbml">
        <rdf:RDF xmlns:dcterms="http://purl.org/dc/terms/" xmlns:rdf="http://www.w3.org/1999/02/22-rdf-syntax-ns#">
          <rdf:Description rdf:about="#COPASI82">
            <dcterms:created>
              <rdf:Description>
                <dcterms:W3CDTF>2011-06-15T16:39:15Z</dcterms:W3CDTF>
              </rdf:Description>
            </dcterms:created>
          </rdf:Description>
        </rdf:RDF>
      </COPASI>
    </annotation>
    <listOfReactants>
      <speciesReference species="species_27" stoichiometry="2"/>
    </listOfReactants>
```

```
        <listOfProducts>
          <speciesReference species="species_36"/>
        </listOfProducts>
        <kineticLaw>
          <math xmlns="http://www.w3.org/1998/Math/MathML">
            <apply>
              <divide/>
              <apply>
                <times/>
                <ci> mwf7e8c4cc_5866_4d9d_80a4_fb1d0db24ecf </ci>
                <ci> species_27 </ci>
              </apply>
              <apply>
                <plus/>
                <ci> mwK1a89ce5e5_bfda_43b5_bb20_0b16e57e3632 </ci>
                <ci> species_27 </ci>
              </apply>
            </apply>
          </math>
          <listOfParameters>
            <parameter id="mwK1a89ce5e5_bfda_43b5_bb20_0b16e57e3632" name="K_TSA1"
value="0.1"/>
          </listOfParameters>
        </kineticLaw>
      </reaction>
      <reaction id="reaction_35" name="Sink TSA" reversible="false" fast="false">
        <annotation>
          <COPASI xmlns="http://www.copasi.org/static/sbml">
```

```
          <rdf:RDF xmlns:dcterms="http://purl.org/dc/terms/"
xmlns:rdf="http://www.w3.org/1999/02/22-rdf-syntax-ns#">
            <rdf:Description rdf:about="#COPASI83">
              <dcterms:created>
                <rdf:Description>
                  <dcterms:W3CDTF>2011-06-15T16:40:30Z</dcterms:W3CDTF>
                </rdf:Description>
              </dcterms:created>
            </rdf:Description>
          </rdf:RDF>
        </COPASI>
      </annotation>
      <listOfReactants>
        <speciesReference species="species_36"/>
      </listOfReactants>
      <listOfProducts>
        <speciesReference species="species_33"/>
      </listOfProducts>
      <kineticLaw>
        <math xmlns="http://www.w3.org/1998/Math/MathML">
          <apply>
            <divide/>
            <apply>
              <times/>
              <ci> mw0f93be3f_8f7b_48cf_b994_7d7d51c0c99d </ci>
              <ci> species_36 </ci>
            </apply>
            <apply>
```

```
            <plus/>
            <ci> mwK125774f66_2af6_440c_a4df_5d10c0a54ba0 </ci>
            <ci> species_36 </ci>
          </apply>
        </apply>
      </math>
      <listOfParameters>
        <parameter id="mwK125774f66_2af6_440c_a4df_5d10c0a54ba0" name="K_TSA2"
value="0.4"/>
      </listOfParameters>
    </kineticLaw>
  </reaction>
  <reaction id="reaction_36" name="GC_2 (tartronate semialdehyde reductase)" reversible="false"
fast="false">
    <annotation>
      <COPASI xmlns="http://www.copasi.org/static/sbml">
        <rdf:RDF xmlns:dcterms="http://purl.org/dc/terms/"
xmlns:rdf="http://www.w3.org/1999/02/22-rdf-syntax-ns#">
          <rdf:Description rdf:about="#COPASI84">
            <dcterms:created>
              <rdf:Description>
                <dcterms:W3CDTF>2011-06-15T16:40:54Z</dcterms:W3CDTF>
              </rdf:Description>
            </dcterms:created>
          </rdf:Description>
        </rdf:RDF>
      </COPASI>
    </annotation>
    <listOfReactants>
```

```
        <speciesReference species="species_36"/>
      </listOfReactants>
      <listOfProducts>
        <speciesReference species="species_31"/>
      </listOfProducts>
      <kineticLaw>
        <math xmlns="http://www.w3.org/1998/Math/MathML">
          <apply>
            <divide/>
            <apply>
              <times/>
              <ci> mw4d57904b_347d_4323_a435_aef6db506bb9 </ci>
              <ci> species_36 </ci>
            </apply>
            <apply>
              <plus/>
              <ci> mwK1e14acb7a_4bcf_44cb_aee8_8a181e61c843 </ci>
              <ci> species_36 </ci>
            </apply>
          </apply>
        </math>
        <listOfParameters>
          <parameter id="mwK1e14acb7a_4bcf_44cb_aee8_8a181e61c843" name="K_TSA3"
value="0.1"/>
        </listOfParameters>
      </kineticLaw>
    </reaction>
    <reaction id="reaction_37" name="OX_1 (glyoxylate oxidase)" reversible="false" fast="false">
```

```
        <annotation>
          <COPASI xmlns="http://www.copasi.org/static/sbml">
            <rdf:RDF xmlns:dcterms="http://purl.org/dc/terms/" xmlns:rdf="http://www.w3.org/1999/02/22-rdf-syntax-ns#">
              <rdf:Description rdf:about="#COPASI85">
                <dcterms:created>
                  <rdf:Description>
                    <dcterms:W3CDTF>2011-06-28T01:12:01Z</dcterms:W3CDTF>
                  </rdf:Description>
                </dcterms:created>
              </rdf:Description>
            </rdf:RDF>
          </COPASI>
        </annotation>
        <listOfReactants>
          <speciesReference species="species_27"/>
        </listOfReactants>
        <listOfProducts>
          <speciesReference species="species_37"/>
        </listOfProducts>
        <kineticLaw>
          <math xmlns="http://www.w3.org/1998/Math/MathML">
            <apply>
              <divide/>
              <apply>
                <times/>
                <ci> mw7f7c8a85_afe2_49aa_8686_1b3950810d23 </ci>
                <cn> 0.8 </cn>
```

```
<ci> species_27 </ci>
</apply>
<apply>
<plus/>
<ci> mw6f022b45_ad82_4bfc_bbbd_0855ddf10ecf </ci>
<ci> species_27 </ci>
</apply>
</apply>
</math>
</kineticLaw>
</reaction>
<reaction id="reaction_38" name="Sink OXA" reversible="false" fast="false">
<annotation>
<COPASI xmlns="http://www.copasi.org/static/sbml">
<rdf:RDF xmlns:dcterms="http://purl.org/dc/terms/"
xmlns:rdf="http://www.w3.org/1999/02/22-rdf-syntax-ns#">
<rdf:Description rdf:about="#COPASI86">
<dcterms:created>
<rdf:Description>
<dcterms:W3CDTF>2011-06-28T01:12:33Z</dcterms:W3CDTF>
</rdf:Description>
</dcterms:created>
</rdf:Description>
</rdf:RDF>
</COPASI>
</annotation>
<listOfReactants>
<speciesReference species="species_37"/>
```

```
        </listOfReactants>
        <listOfProducts>
          <speciesReference species="species_34"/>
        </listOfProducts>
        <kineticLaw>
          <math xmlns="http://www.w3.org/1998/Math/MathML">
            <apply>
              <divide/>
              <apply>
                <times/>
                <ci> mwa562da41_e9ca_4d43_85ba_4372aa40bed8 </ci>
                <cn> 1.34 </cn>
                <ci> species_37 </ci>
              </apply>
              <apply>
                <plus/>
                <ci> mwcd39d210_482b_47b5_b954_07cc17e31155 </ci>
                <ci> species_37 </ci>
              </apply>
            </apply>
          </math>
        </kineticLaw>
      </reaction>
      <reaction id="mw33f9b969_7c4e_45ca_9a58_1283f92cad68" name="PP_1 (RuBisCO)"
reversible="false" fast="false">
        <listOfReactants>
          <speciesReference species="species_3"/>
          <speciesReference species="species_1"/>
```

```
  <speciesReference species="species_6"/>
  <speciesReference species="species_2"/>
  <speciesReference species="species_14"/>
  <speciesReference species="species_8"/>
  <speciesReference species="species_12"/>
  <speciesReference species="species_5"/>
</listOfReactants>
<listOfProducts>
  <speciesReference species="species_25"/>
  <speciesReference species="species_2"/>
  <speciesReference species="species_6"/>
  <speciesReference species="species_14"/>
  <speciesReference species="species_8"/>
  <speciesReference species="species_12"/>
  <speciesReference species="species_5"/>
</listOfProducts>
<kineticLaw>
  <math xmlns="http://www.w3.org/1998/Math/MathML">
    <apply>
      <divide/>
      <apply>
        <times/>
        <ci> species_3 </ci>
        <apply>
          <divide/>
          <apply>
            <times/>
```

<ci> mw9f919ddc_7006_491f_b827_5b5094d66dd0 </ci>

<cn> 5.3 </cn>

<ci> species_1 </ci>

</apply>

<apply>

<plus/>

<ci> species_1 </ci>

<apply>

<times/>

<ci> mwd5269cf8_ccf6_4ec1_8ba7_8e8de3f91fd3 </ci>

<apply>

<plus/>

<cn type="integer"> 1 </cn>

<apply>

<divide/>

<ci> species_6 </ci>

<ci> mw58e11717_8712_4ffa_b588_c717796bdd73 </ci>

</apply>

</apply>

</apply>

</apply>

</apply>

</apply>

<apply>

<plus/>

<ci> species_3 </ci>

<apply>

<times/>
<ci> mwe084ba25_0a71_42ea_8ebb_20a6485bc610 </ci>
<apply>
<plus/>
<cn type="integer"> 1 </cn>
<apply>
<divide/>
<ci> species_2 </ci>
<ci> mwb7f4d7b4_05fc_4d66_9774_48dd6b62def8 </ci>
</apply>
<apply>
<divide/>
<ci> species_14 </ci>
<ci> mwbfaa378d_c0c2_49ba_960c_cb089dfe679a </ci>
</apply>
<apply>
<divide/>
<ci> species_8 </ci>
<ci> mw7ce6353b_b8ee_45d4_b210_882da23dc1a6 </ci>
</apply>
<apply>
<divide/>
<ci> species_12 </ci>
<ci> mw7c22b6fb_091f_473c_8ff6_62f0470aa853 </ci>
</apply>
<apply>
<divide/>

```xml
<ci> species_5 </ci>

<ci> mw6320b291_cd55_41c4_a212_0f802e1bd889 </ci>

</apply>

</apply>

</apply>

</apply>

</apply>

</math>

<listOfParameters>

<parameter id="mwbfaa378d_c0c2_49ba_960c_cb089dfe679a" name="KI12" value="0.08"/>

<parameter id="mwb7f4d7b4_05fc_4d66_9774_48dd6b62def8" name="KI11" value="0.84"/>

<parameter id="mw7c22b6fb_091f_473c_8ff6_62f0470aa853" name="KI14" value="0.9"/>

<parameter id="mw7ce6353b_b8ee_45d4_b210_882da23dc1a6" name="KI13" value="0.075"/>

<parameter id="mw6320b291_cd55_41c4_a212_0f802e1bd889" name="KI15" value="0.07"/>

<parameter id="mwd5269cf8_ccf6_4ec1_8ba7_8e8de3f91fd3" name="KM11" value="0.0115"/>

<parameter id="mw58e11717_8712_4ffa_b588_c717796bdd73" name="KM12" value="0.222"/>

<parameter id="mwe084ba25_0a71_42ea_8ebb_20a6485bc610" name="KM13" value="0.02"/>

</listOfParameters>

</kineticLaw>

</reaction>

<reaction id="mwfb52f808_34ac_44be_8317_544bfa615dbc" name="GL_1 alpha (phosphofructokinase)" reversible="false" fast="false">

<listOfReactants>

<speciesReference species="species_15"/>

</listOfReactants>
```

```
<listOfProducts>
  <speciesReference species="species_14"/>
</listOfProducts>
<kineticLaw>
  <math xmlns="http://www.w3.org/1998/Math/MathML">
    <apply>
      <divide/>
      <apply>
        <times/>
        <ci> mwfb9d0842_5ad1_42a2_b44c_12cfeaa36962 </ci>
        <cn> 0.87 </cn>
        <ci> species_15 </ci>
      </apply>
      <apply>
        <plus/>
        <ci> species_15 </ci>
        <ci> mwc59d3254_509c_411a_b656_9e80ac0a4d4a </ci>
      </apply>
    </apply>
  </math>
</kineticLaw>
</reaction>
<reaction id="mw888f46db_cf06_4f12_ad55_d96247e91c02" name="PP_2b (phosphoglycolate
phosphatase)" reversible="false" fast="false">
<listOfReactants>
  <speciesReference species="species_25"/>
</listOfReactants>
<listOfProducts>
```

```
  <speciesReference species="species_26"/>
  <speciesReference species="species_12"/>
</listOfProducts>
<kineticLaw>
  <math xmlns="http://www.w3.org/1998/Math/MathML">
    <apply>
      <divide/>
      <apply>
        <times/>
        <ci> mwa378ec6c_6ace_46c3_a515_4be6d27ac144 </ci>
        <cn> 0.78 </cn>
        <ci> species_25 </ci>
      </apply>
      <apply>
        <plus/>
        <ci> species_25 </ci>
        <apply>
          <times/>
          <ci> mw701ca18f_a968_4128_8415_d39acae3e377 </ci>
          <apply>
            <plus/>
            <cn type="integer"> 1 </cn>
            <apply>
              <divide/>
              <ci> species_26 </ci>
              <ci> mw6cacd0a2_9c0d_4e6c_ac36_efd63ce3bf57 </ci>
            </apply>
```

```
              </apply>
              <apply>
                <plus/>
                <cn type="integer"> 1 </cn>
                <apply>
                  <divide/>
                  <ci> species_12 </ci>
                  <ci> mw4ebf27da_6677_470e_a6ec_3d1e15922742 </ci>
                </apply>
              </apply>
            </apply>
          </apply>
        </apply>
      </math>
      <listOfParameters>
        <parameter id="mw6cacd0a2_9c0d_4e6c_ac36_efd63ce3bf57" name="KI1121" value="94"/>
        <parameter id="mw4ebf27da_6677_470e_a6ec_3d1e15922742" name="KI1122"
value="2.55"/>
      </listOfParameters>
    </kineticLaw>
  </reaction>
  <reaction id="mw8c050f3c_c600_4cba_8a38_619387f62796" name="GL_3a (phosphoglycerate
mutase)" fast="false">
    <listOfReactants>
      <speciesReference species="species_2"/>
    </listOfReactants>
    <listOfProducts>
      <speciesReference species="species_35"/>
```

</listOfProducts>
<kineticLaw>
<math xmlns="http://www.w3.org/1998/Math/MathML">
<apply>
<divide/>
<apply>
<times/>
<ci> mw4f571ae6_7040_40c6_b098_4ac7cf9ee24d </ci>
<cn> 1.12 </cn>
<apply>
<minus/>
<ci> species_2 </ci>
<apply>
<divide/>
<ci> species_35 </ci>
<ci> mw917c32b0_7a3f_4744_ac14_2e3d15da96f6 </ci>
</apply>
</apply>
</apply>
<apply>
<times/>
<ci> mw742fbec5_509e_42be_b7a8_12b9eeb49314 </ci>
<apply>
<plus/>
<cn type="integer"> 1 </cn>
<apply>
<divide/>

```
            <ci> species_2 </ci>
            <ci> mw742fbec5_509e_42be_b7a8_12b9eeb49314 </ci>
          </apply>
          <apply>
            <divide/>
            <ci> species_35 </ci>
            <ci> mw231dd6ca_4db0_4cdb_adc8_72349abdba07 </ci>
          </apply>
        </apply>
      </apply>
    </apply>
  </math>
 </kineticLaw>
</reaction>
<reaction id="mw13e13d10_557f_4f5e_9340_62df61395963" name="Sink GLY"
reversible="false" fast="false">
 <listOfReactants>
  <speciesReference species="species_28"/>
 </listOfReactants>
 <listOfProducts>
  <speciesReference species="species_24"/>
 </listOfProducts>
 <kineticLaw>
  <math xmlns="http://www.w3.org/1998/Math/MathML">
    <apply>
      <divide/>
      <apply>
        <times/>
```

```
            <ci> mw9c6e9e64_1ad3_462a_b257_61d666a10a15 </ci>
            <cn> 2.2 </cn>
            <ci> species_28 </ci>
          </apply>
          <apply>
            <plus/>
            <ci> mw64f1bfc2_b040_4495_98d5_84e5a2e9b1c0 </ci>
            <ci> species_28 </ci>
          </apply>
        </apply>
      </math>
      <listOfParameters>
        <parameter id="mw64f1bfc2_b040_4495_98d5_84e5a2e9b1c0" name="K_Sink_GLY" value="1"/>
      </listOfParameters>
    </kineticLaw>
  </reaction>
  <reaction id="mwc28b2370_81f7_45f3_a71d_18314992330e" name="Sink SER" reversible="false" fast="false">
    <listOfReactants>
      <speciesReference species="species_29"/>
    </listOfReactants>
    <listOfProducts>
      <speciesReference species="species_24"/>
    </listOfProducts>
    <kineticLaw>
      <math xmlns="http://www.w3.org/1998/Math/MathML">
        <apply>
```

```
<divide/>

<apply>

<times/>

<ci> mw108b366f_58cf_4f32_9f78_d3a14dd13026 </ci>

<cn> 0.35 </cn>

<ci> species_29 </ci>

</apply>

<apply>

<plus/>

<ci> mw7e38db19_97ad_43ac_a0b0_3a88f4184f72 </ci>

<ci> species_29 </ci>

</apply>

</apply>

</math>

<listOfParameters>

<parameter id="mw7e38db19_97ad_43ac_a0b0_3a88f4184f72" name="K_Sink_SER"
value="1"/>

</listOfParameters>

</kineticLaw>

</reaction>

<reaction id="mwe1c72296_a05e_459f_97dc_559333cc5eb6" name="GL_3b (phosphoglycerate
mutase)" fast="false">

<listOfReactants>

<speciesReference species="species_2"/>

</listOfReactants>

<listOfProducts>

<speciesReference species="species_35"/>

</listOfProducts>
```

```
<kineticLaw>
 <math xmlns="http://www.w3.org/1998/Math/MathML">
  <apply>
   <divide/>
   <apply>
    <times/>
    <ci> mw2ec1703a_729a_4b49_b4dd_4241b0d73907 </ci>
    <cn> 1.17 </cn>
    <apply>
     <minus/>
     <ci> species_2 </ci>
     <apply>
      <divide/>
      <ci> species_35 </ci>
      <ci> mw917c32b0_7a3f_4744_ac14_2e3d15da96f6 </ci>
     </apply>
    </apply>
   </apply>
   <apply>
    <times/>
    <ci> mwb99ebe97_7d91_406c_8b12_a56b2c939ff4 </ci>
    <apply>
     <plus/>
     <cn type="integer"> 1 </cn>
     <apply>
      <divide/>
      <ci> species_2 </ci>
```

<ci> mwb99ebe97_7d91_406c_8b12_a56b2c939ff4 </ci>

</apply>

<apply>

<divide/>

<ci> species_35 </ci>

<ci> mw23fae7dc_7d12_4fb0_98e2_c611e44007e1 </ci>

</apply>

</apply>

</apply>

</apply>

</math>

</kineticLaw>

</reaction>

<reaction id="mw63f09b77_c48f_4b1e_a2a6_7d5f6d48ea38" name="GL_3c (phosphoglycerate mutase)" fast="false">

<listOfReactants>

<speciesReference species="species_2"/>

</listOfReactants>

<listOfProducts>

<speciesReference species="species_35"/>

</listOfProducts>

<kineticLaw>

<math xmlns="http://www.w3.org/1998/Math/MathML">

<apply>

<divide/>

<apply>

<times/>

<ci> mwa1879047_3c26_45c1_8b2a_137433500cde </ci>

```
  <cn> 0.78 </cn>
  <apply>
    <minus/>
    <ci> species_2 </ci>
    <apply>
      <divide/>
      <ci> species_35 </ci>
      <ci> mw917c32b0_7a3f_4744_ac14_2e3d15da96f6 </ci>
    </apply>
  </apply>
</apply>
<apply>
  <times/>
  <ci> mw6205c3fd_ecf9_4b2b_98f1_dff68729cd03 </ci>
  <apply>
    <plus/>
    <cn type="integer"> 1 </cn>
    <apply>
      <divide/>
      <ci> species_2 </ci>
      <ci> mw6205c3fd_ecf9_4b2b_98f1_dff68729cd03 </ci>
    </apply>
    <apply>
      <divide/>
      <ci> species_35 </ci>
      <ci> mw23f5979c_8188_4521_9629_6469c95e42d6 </ci>
    </apply>
```

</apply>

</apply>

</apply>

</math>

</kineticLaw>

</reaction>

<reaction id="mw153159fa_5578_45d6_8d12_aacee3745c81" name="CO2 import" reversible="false" fast="false">

<listOfReactants>

<speciesReference species="mw733b668e_1dba_46ef_9777_e9331b0b590d"/>

</listOfReactants>

<listOfProducts>

<speciesReference species="species_1"/>

</listOfProducts>

<kineticLaw>

<math xmlns="http://www.w3.org/1998/Math/MathML">

<apply>

<times/>

<ci> mwaffea60c_8361_4bed_be10_814e0c0f7898 </ci>

<cn> 0.141 </cn>

<ci> mw733b668e_1dba_46ef_9777_e9331b0b590d </ci>

</apply>

</math>

</kineticLaw>

</reaction>

<reaction id="mwc0a63025_cc5a_48e0_8d5e_91b9f8bb5250" name="GL_2 (glyceraldehyde 3-phosphate dehydrogenase)" fast="false">

<listOfReactants>

```
  <speciesReference species="species_10"/>
  <speciesReference species="species_11"/>
</listOfReactants>
<listOfProducts>
  <speciesReference species="species_2"/>
  <speciesReference species="species_5"/>
</listOfProducts>
<kineticLaw>
  <math xmlns="http://www.w3.org/1998/Math/MathML">
    <apply>
      <divide/>
      <apply>
        <times/>
        <ci> mwd0f35f82_c657_4a53_9ac4_606e79cebb96 </ci>
        <cn> 0.79 </cn>
        <apply>
          <minus/>
          <apply>
            <times/>
            <ci> species_10 </ci>
            <ci> species_11 </ci>
          </apply>
          <apply>
            <times/>
            <ci> mw1a31f571_e225_4d2d_b672_bb6209080e70 </ci>
            <ci> species_2 </ci>
            <ci> species_5 </ci>
```

```
    </apply>
  </apply>
</apply>
<apply>
  <times/>
  <apply>
    <plus/>
    <cn type="integer"> 1 </cn>
    <apply>
      <divide/>
      <ci> species_10 </ci>
      <ci> mw2a519375_b634_461c_a4e7_2205e166b3ce </ci>
    </apply>
    <apply>
      <divide/>
      <ci> species_2 </ci>
      <ci> mw25dcc228_99f4_4d62_badc_00067a7db23a </ci>
    </apply>
  </apply>
  <apply>
    <plus/>
    <cn type="integer"> 1 </cn>
    <apply>
      <divide/>
      <ci> species_11 </ci>
      <ci> mw02659a3e_942b_4113_928b_885d34341771 </ci>
    </apply>
```

<apply>
<divide/>
<ci> species_5 </ci>
<ci> mw31fc107e_644a_475e_aeff_2b016202c816 </ci>
</apply>
</apply>
</apply>
</apply>
</math>
</kineticLaw>
</reaction>
<reaction id="mwd149e74a_6b61_4bd9_8b74_1d3187a49160" name="GSM_2 (glycine transaminase)" reversible="false" fast="false">
<listOfReactants>
<speciesReference species="species_27"/>
</listOfReactants>
<listOfProducts>
<speciesReference species="species_28"/>
</listOfProducts>
<kineticLaw>
<math xmlns="http://www.w3.org/1998/Math/MathML">
<apply>
<divide/>
<apply>
<times/>
<ci> mw016a3190_5878_41d4_8022_96365a5cde42 </ci>
<ci> species_27 </ci>
</apply>

```
          <apply>
            <plus/>
            <ci> mw1862dfa3_5857_4ef7_82ea_209a042715a4 </ci>
            <ci> species_27 </ci>
          </apply>
        </apply>
      </math>
    </kineticLaw>
  </reaction>
  <reaction id="mw04b20bf0_57cd_45c3_8316_a103dafa35fe" name="Sink G6P" reversible="false" fast="false">
    <listOfReactants>
      <speciesReference species="species_21"/>
    </listOfReactants>
    <listOfProducts>
      <speciesReference species="mw8dc354b7_d1b2_4cc9_bbf9_5d423d6013fb" stoichiometry="6"/>
    </listOfProducts>
    <kineticLaw>
      <math xmlns="http://www.w3.org/1998/Math/MathML">
        <apply>
          <divide/>
          <apply>
            <times/>
            <ci> mw2fcbcb1c_c103_48bb_86ed_9229a27de6ed </ci>
            <ci> species_21 </ci>
          </apply>
          <apply>
```

```
            <plus/>
            <ci> mw52505a99_c3c7_4c4c_8a62_b891bfab4dd8 </ci>
            <ci> species_21 </ci>
          </apply>
        </apply>
      </math>
    </kineticLaw>
  </reaction>
  <reaction id="mw92eee159_bf34_47ce_adc3_21834f561f8e" name="PKET alpha
(phosphoketolase)" reversible="false" fast="false">
    <listOfReactants>
      <speciesReference species="species_15"/>
    </listOfReactants>
    <listOfProducts>
      <speciesReference species="mwfe019cb4_4031_44c9_83f8_9ba5e8de4ed4"/>
      <speciesReference species="species_16"/>
    </listOfProducts>
    <kineticLaw>
      <math xmlns="http://www.w3.org/1998/Math/MathML">
        <apply>
          <divide/>
          <apply>
            <times/>
            <ci> mw72f442a2_e01a_44d3_b8bf_9d223245f350 </ci>
            <cn> 1.35 </cn>
            <ci> species_15 </ci>
          </apply>
          <apply>
```

```
            <plus/>
            <ci> species_15 </ci>
            <ci> mwa27b7723_ab20_4324_a886_f5c73588ac4a </ci>
          </apply>
        </apply>
      </math>
    </kineticLaw>
  </reaction>
  <reaction id="mw0891344d_8739_44c9_a4fb_b36347073110" name="Sink AceP"
reversible="false" fast="false">
    <listOfReactants>
      <speciesReference species="mwfe019cb4_4031_44c9_83f8_9ba5e8de4ed4"/>
    </listOfReactants>
    <listOfProducts>
      <speciesReference species="mwe556cd16_34e2_417a_b945_37061560bfe5"
stoichiometry="2"/>
    </listOfProducts>
    <kineticLaw>
      <math xmlns="http://www.w3.org/1998/Math/MathML">
        <apply>
          <divide/>
          <apply>
            <times/>
            <ci> mwd7c8b802_9d0c_4a81_9c65_1a50be7968bb </ci>
            <ci> mwfe019cb4_4031_44c9_83f8_9ba5e8de4ed4 </ci>
          </apply>
          <apply>
            <plus/>
```

```
            <ci> mwc1298b32_b9e2_4ab0_8087_3acdea483cdc </ci>
            <ci> mwfe019cb4_4031_44c9_83f8_9ba5e8de4ed4 </ci>
          </apply>
        </apply>
      </math>
    </kineticLaw>
  </reaction>
  <reaction id="mw26097e16_ab9d_4350_98c3_2e61cdf3ff9f" name="PKET beta
(phosphoketolase)" reversible="false" fast="false">
    <listOfReactants>
      <speciesReference species="species_15"/>
    </listOfReactants>
    <listOfProducts>
      <speciesReference species="mwfe019cb4_4031_44c9_83f8_9ba5e8de4ed4"/>
      <speciesReference species="species_16"/>
    </listOfProducts>
    <kineticLaw>
      <math xmlns="http://www.w3.org/1998/Math/MathML">
        <apply>
          <divide/>
          <apply>
            <times/>
            <ci> mw86416738_ea27_41b8_adf9_4d3b5a320d97 </ci>
            <cn> 1.1 </cn>
            <ci> species_15 </ci>
          </apply>
          <apply>
            <plus/>
```

```xml
            <ci> species_15 </ci>
            <ci> mw57949b29_e69c_444c_8d58_1ddda96d13c5 </ci>
          </apply>
        </apply>
      </math>
    </kineticLaw>
  </reaction>
  <reaction id="mwcb3cefb7_75fa_446c_9836_44386ea80892" name="GL_1 beta
(phosphofructokinase)" reversible="false" fast="false">
    <listOfReactants>
      <speciesReference species="species_15"/>
    </listOfReactants>
    <listOfProducts>
      <speciesReference species="species_14"/>
    </listOfProducts>
    <kineticLaw>
      <math xmlns="http://www.w3.org/1998/Math/MathML">
        <apply>
          <divide/>
          <apply>
            <times/>
            <ci> mw61b0fed3_e790_4af3_a1e7_65c1646248bf </ci>
            <cn> 1.11 </cn>
            <ci> species_15 </ci>
          </apply>
          <apply>
            <plus/>
            <ci> species_15 </ci>
```

```
            <ci> mw9d451500_8215_4269_83be_e237d42f0aab </ci>
          </apply>
        </apply>
      </math>
    </kineticLaw>
  </reaction>
  <reaction id="mw31133011_39d2_478f_bee1_c84fabc8343e" name="CC_6 beta (fructose-1,6-
bisphosphatase)" reversible="false" fast="false">
    <listOfReactants>
      <speciesReference species="species_14"/>
    </listOfReactants>
    <listOfProducts>
      <speciesReference species="species_15"/>
      <speciesReference species="species_12"/>
    </listOfProducts>
    <kineticLaw>
      <math xmlns="http://www.w3.org/1998/Math/MathML">
        <apply>
          <divide/>
          <apply>
            <times/>
            <ci> mw13a07b09_9a67_4561_b3db_8e39442e5d53 </ci>
            <cn> 0.87 </cn>
            <ci> species_14 </ci>
          </apply>
          <apply>
            <plus/>
            <ci> species_14 </ci>
```

<apply>

<times/>

<ci> mwdb1e1973_5df2_4a29_a277_64c677c902b4 </ci>

<apply>

<plus/>

<cn type="integer"> 1 </cn>

<apply>

<divide/>

<ci> species_15 </ci>

<ci> mwd2bb54b2_f070_41d0_a500_9b196833f57a </ci>

</apply>

<apply>

<divide/>

<ci> species_12 </ci>

<ci> mw9f468e68_fa4c_4260_a5af_3b6182abcb92 </ci>

</apply>

</apply>

</apply>

</apply>

</apply>

</math>

</kineticLaw>

</reaction>

<reaction id="mwe1c332df_bf6c_404f_b207_fa3e8ee321b4" name="C_11 beta (phosphopentose isomerase)" fast="false">

<listOfReactants>

<speciesReference species="species_18"/>

</listOfReactants>

```
<listOfProducts>
  <speciesReference species="species_20"/>
</listOfProducts>
<kineticLaw>
  <math xmlns="http://www.w3.org/1998/Math/MathML">
    <apply>
      <divide/>
      <apply>
        <times/>
        <ci> mw15a7ce92_eb88_4188_966c_aad7e9d8ea7e </ci>
        <cn> 0.63 </cn>
        <apply>
          <minus/>
          <ci> species_18 </ci>
          <apply>
            <divide/>
            <ci> species_20 </ci>
            <ci> mw94fab6bb_d1d8_4344_ac40_d9ea47173826 </ci>
          </apply>
        </apply>
      </apply>
      <apply>
        <times/>
        <ci> mw8da43b9c_22d4_4c48_bcc7_0bb2a83b77f6 </ci>
        <apply>
          <plus/>
          <cn type="integer"> 1 </cn>
```

```
<apply>
  <divide/>
  <ci> species_18 </ci>
  <ci> mw8da43b9c_22d4_4c48_bcc7_0bb2a83b77f6 </ci>
</apply>
<apply>
  <divide/>
  <ci> species_20 </ci>
  <ci> mw332683df_4959_4366_94ef_2b7108d780dc </ci>
</apply>
</apply>
</apply>
</apply>
</math>
</kineticLaw>
</reaction>
<reaction id="mw6ca245ce_5769_4e56_893d_f2a8e43f8119" name="PPP_1 (glucose-6-
phosphate dehydrogenase)" reversible="false" fast="false">
<listOfReactants>
  <speciesReference species="species_21"/>
</listOfReactants>
<listOfProducts>
  <speciesReference species="mw3447e887_c5e2_4271_9e5f_50c309bc8dc3"/>
</listOfProducts>
<kineticLaw>
  <math xmlns="http://www.w3.org/1998/Math/MathML">
    <apply>
      <divide/>
```

```
      <apply>
        <times/>
        <ci> mw84e3d5fb_d3f5_4a42_a963_2d0e4de0c9fa </ci>
        <cn> 0.08 </cn>
        <ci> species_21 </ci>
      </apply>
      <apply>
        <times/>
        <ci> mw212994b8_ca26_4fbc_a13e_bb054a312812 </ci>
        <apply>
          <plus/>
          <cn type="integer"> 1 </cn>
          <apply>
            <divide/>
            <ci> species_21 </ci>
            <ci> mw212994b8_ca26_4fbc_a13e_bb054a312812 </ci>
          </apply>
          <apply>
            <divide/>
            <ci> mw3447e887_c5e2_4271_9e5f_50c309bc8dc3 </ci>
            <ci> mw80012d61_bf17_467d_8029_8785e8b66833 </ci>
          </apply>
        </apply>
      </apply>
    </apply>
  </math>
</kineticLaw>
```

```
    </reaction>
    <reaction id="mw068859c7_1ee9_41f0_aaab_4cb88de606d7" name="PPP_2 (phosphogluconate dehydrogenase)" reversible="false" fast="false">
      <listOfReactants>
        <speciesReference species="mw3447e887_c5e2_4271_9e5f_50c309bc8dc3"/>
        <speciesReference species="species_11"/>
      </listOfReactants>
      <listOfProducts>
        <speciesReference species="species_20"/>
        <speciesReference species="species_5"/>
      </listOfProducts>
      <kineticLaw>
        <math xmlns="http://www.w3.org/1998/Math/MathML">
          <apply>
            <divide/>
            <apply>
              <times/>
              <ci> mwacee8ded_69a8_4141_87f2_2622f06daa29 </ci>
              <cn> 0.07 </cn>
              <ci> mw3447e887_c5e2_4271_9e5f_50c309bc8dc3 </ci>
              <ci> species_11 </ci>
            </apply>
            <apply>
              <times/>
              <apply>
                <plus/>
                <ci> mw3447e887_c5e2_4271_9e5f_50c309bc8dc3 </ci>
                <ci> mw9aad6b3a_ddc4_4aa7_af1d_9c6f98a72a80 </ci>
```

</apply>
<apply>
<plus/>
<ci> species_11 </ci>
<ci> mwbf2d484b_0c51_4597_84e7_a9d72a8ed607 </ci>
</apply>
</apply>
</apply>
</math>
</kineticLaw>
</reaction>
<reaction id="mw84530e4e_bd49_4c49_96fa_3753a80777df" name="PP_2c (phosphoglycolate phosphatase)" reversible="false" fast="false">
<listOfReactants>
<speciesReference species="species_25"/>
</listOfReactants>
<listOfProducts>
<speciesReference species="species_26"/>
<speciesReference species="species_12"/>
</listOfProducts>
<kineticLaw>
<math xmlns="http://www.w3.org/1998/Math/MathML">
<apply>
<divide/>
<apply>
<times/>
<ci> mwe7492e74_1fd5_412c_9bd7_c33659cbb9ae </ci>
<cn> 1.003 </cn>

```
  <ci> species_25 </ci>
</apply>
<apply>
  <plus/>
  <ci> species_25 </ci>
  <apply>
    <times/>
    <ci> mw2026f64b_36e7_4da9_8f99_4179a8aa5a0f </ci>
    <apply>
      <plus/>
      <cn type="integer"> 1 </cn>
      <apply>
        <divide/>
        <ci> species_26 </ci>
        <ci> mw42e7bfb9_db8f_4b73_bd5b_4f46a35e36a0 </ci>
      </apply>
    </apply>
    <apply>
      <plus/>
      <cn type="integer"> 1 </cn>
      <apply>
        <divide/>
        <ci> species_12 </ci>
        <ci> mw43a4fc8c_e159_44c4_aca5_0de4d39f2892 </ci>
      </apply>
    </apply>
  </apply>
```

</apply>
</apply>
</math>
</kineticLaw>
</reaction>
</listOfReactions>
</model>
</sbml>
